# Safety and immunogenicity of Vi-DT conjugate vaccine among 6-23-month-old children: Phase II, randomized, dose-scheduling, observer-blind Study

**DOI:** 10.1016/j.eclinm.2020.100540

**Published:** 2020-09-09

**Authors:** Maria Rosario Capeding, Arijit Sil, Birkneh Tilahun Tadesse, Tarun Saluja, Samuel Teshome, Edison Alberto, Deok Ryun Kim, Eun Lyeong Park, Ju Yeon Park, Jae Seung Yang, Suchada Chinaworapong, Jiwook Park, Sue-Kyoung Jo, Yun Chon, Seon-Young Yang, Ji Hwa Ryu, Inho Cheong, Kyu-Young Shim, Yoonyeong Lee, Hun Kim, Julia A. Lynch, Jerome H. Kim, Jean-Louis Excler, T. Anh Wartel, Sushant Sahastrabuddhe

**Affiliations:** 1 Research Institute for Tropical Medicine, Manila, The Philippines; 2 International Vaccine Institute, Seoul, Republic of Korea; 3 CORE Group Polio Project, Ethiopia; 4 SK bioscience, Seoul, Republic of Korea

**Keywords:** Infants and toddlers, Typhoid, typhoid conjugate vaccine, Vi-DT

## Abstract

**Background:**

Typhoid causes significant mortality among young children in resource-limited settings. Conjugate typhoid vaccines could significantly reduce typhoid-related child deaths, but only one WHO-prequalified typhoid conjugate vaccine exists for young children. To address this gap, we investigated the safety, immunogenicity and dose-scheduling of Vi-DT typhoid conjugate vaccine among children aged 6-23 months.

**Methods:**

In this single center, observer blind, phase II trial, participants were randomly assigned (2:2:1) to receive one or two doses of Vi-DT or comparator vaccine. Anti-Vi IgG titer and geometric mean titers (GMT) were determined at 0, 4, 24 and 28 weeks. Data were analyzed using per-protocol and immunogenicity (a subset of intention-to-treat analysis) sets. The trial is registered with ClinicalTrials.gov (NCT03527355).

**Findings:**

Between April and July 2018, 285 children were randomized; 114 received one or two doses of Vi-DT while 57 received comparator. 277 completed the study follow-up per protocol; 112 and 110 from single- and two-dose Vi-DT schedules, respectively and 55 from the placebo group were included in the per protocol analysis. Safety profile is satisfactory. Thirteen serious adverse events were reported during the 28-week follow-up, none of which were related to Vi-DT. The seroconversion rate four weeks after the first dose was 100% (95% CI 98·3-100) in Vi-DT recipients and 7·0% (95% CI 2·8-16·7) in comparator recipients (p<0·0001). Similarly, the seroconversion rate 4 weeks after the second dose was 98·2% (95% CI 93· 6-99·5) and 21·8% (95% CI 13·0-34·4) among Vi-DT and comparator groups, respectively (p<0·0001). Anti-Vi IgG GMT was significantly higher in Vi-DT than in control group at all post-vaccination visits (p<0·0001).

**Interpretation:**

Both single and two doses of Vi-DT vaccine are safe, well tolerated, and immunogenic for infants and toddlers in a moderately endemic setting.

**Research in context**

**Evidence before this study**

We reviewed the 2018 Cochrane report on typhoid vaccines and searched PubMed using “typhoid conjugate vaccine (TCV)”, “Vi-DT conjugate”, “typhoid” and “vaccine” with no language restrictions up to April 30, 2020. There were two phase I studies evaluating the safety and immunogenicity of Vi-DT vaccine among children and adults. The first phase I trial published in 2018 evaluated Vi-DT among 144 adults and 24 children among healthy Filipino adults and children. The second study published in 2019 included 118 participants, of whom 25 were children from 6 months to 12 years. Both studies showed that Vi-DT was safe and immunogenic. There were no phase II and beyond studies of Vi-DT among children less than 24 months of age.

**Added value of this study**

This is the first phase II trial to assess the safety and immunogenicity six months after vaccination of Vi-DT vaccine manufactured by SK bioscience among infants and toddlers. We show that both single and two doses of Vi-DT are safe and immunogenic in children 6-23 months of age, a group bearing significant typhoid morbidity and mortality. Data generated from this trial will be crucial to support licensure followed by WHO prequalification and introduction of Vi-DT vaccine in routine childhood immunization programs.

**Implications of all the available evidence**

Our findings confirm that Vi-DT is safe and immunogenic among children 6-23 months of age. TCVs are recommended by WHO for use in endemic settings and supported by Gavi for eligible countries. There is however only one WHO-prequalified TCV and demand currently exceeds supply. Findings from the current trial will be critical for licensure and WHO prequalification of Vi-DT, contributing to filling the gap in supply.

Alt-text: Unlabelled box

## 1 Introduction

Typhoid fever, an invasive infection caused by *Salmonella enterica serovar* Typhi (*S*. Typhi) is an important cause of morbidity and mortality across all age groups in resource limited settings [1,2]. Worldwide, over 10 million cases and around 115,000 deaths are attributed to *S.* Typhi infections annually [3]. A higher burden of disease has been reported among 6 to12-year-old children and toddlers [1,4, 5, 6, 7, 8, 9, 10]. Typhoid surveillance studies report that a quarter to more than half of cases with invasive *S.* Typhi disease are in the under-five age group [5,7].

Currently, antibiotics are the mainstay of treatment for cases of *S.* Typhi infections. However, multi and extensive drug resistant *S*. Typhi infections have been reported from several countries in Asia and Africa 11, 12, 13, 14, 15, 16, 17. The prevention of typhoid fever through immunization and other measures has the potential to decrease antibiotic use and limit the emergence of resistant S. Typhi strains. The emergence of antimicrobial resistant (AMR) *S.* Typhi infections contributed to the WHO guidelines for the introduction of typhoid fever vaccination in populations at high risk of infection [5,18, 19, 20, 21, 22, 23, 24].

Typhoid vaccines that can help reduce burden of disease are licensed [25]. Three or four doses of orally administered live-attenuated Ty21a vaccine provide about 50–70% protection for at least 7 years, licensed in capsule form from 5 years of age 26, 27, 28. The single-dose injectable Vi polysaccharide vaccine provides similar levels of protection for up to 3 years and is licensed from 2 years of age [29,30].

*S.* Typhi Vi polysaccharide vaccines are T-cell independent, lack affinity maturation, have poor antibody subclass switching and are unable to generate memory, which limits their use in children less than two years of age [31]. These limitations of Vi polysaccharide vaccines can be overcome by conjugation with a carrier protein that converts the immune response to T-cell dependent [32]. Improving the immunogenicity of typhoid conjugate vaccines in children under 2 years of age is an important advance given the significant burden of disease in young children and infants [5,7,33]. A first-in-human phase I trial conducted in the Philippines and another phase I trial conducted in Indonesia assessed the safety of Vi-DT conjugate vaccine compared to Vi polysaccharide (Typhim Vi®, Sanofi Pasteur) typhoid vaccine among healthy 2-45 years old adults and children [34,35]. No serious adverse events were reported in either group, and there was no difference in the frequency of solicited and unsolicited adverse events and medically significant events.

Following the successful completion of the phase I trial, further investigation on the safety and immunogenicity of Vi-DT conjugate vaccine among younger children was conducted in 6-23 month old subjects in a randomized, observer blinded, phase II clinical trial in the Philippines. In the phase I trial, the vaccine was shown to be safe and immunogenic four weeks post first dose. No increase in GMT was observed after the second dose of Vi-DT given 4 weeks later [34]. We here report the results of safety, immunogenicity and durability of immune responses of Vi-DT among children 6-23 months of age at 4 and 28 weeks after a single dose or at weeks 4 and 28 after a 0 and 24 week 2-dose regimen in children aged 6-23 months.

## 2 Methods

### 2.1 Study Design

The details of the trial design were recently published [36]. Briefly, the trial was a randomized, controlled, observer-blinded three-group phase II study (Clinicaltrials.gov: NCT03527355) with allocation ratio of 2:2:1 among healthy infants and toddlers 6-23 months of age. Groups A, B, and C represented children who received single dose, two doses or a comparator vaccine, respectively.

### 2.2 Participants

**Figure 1:**
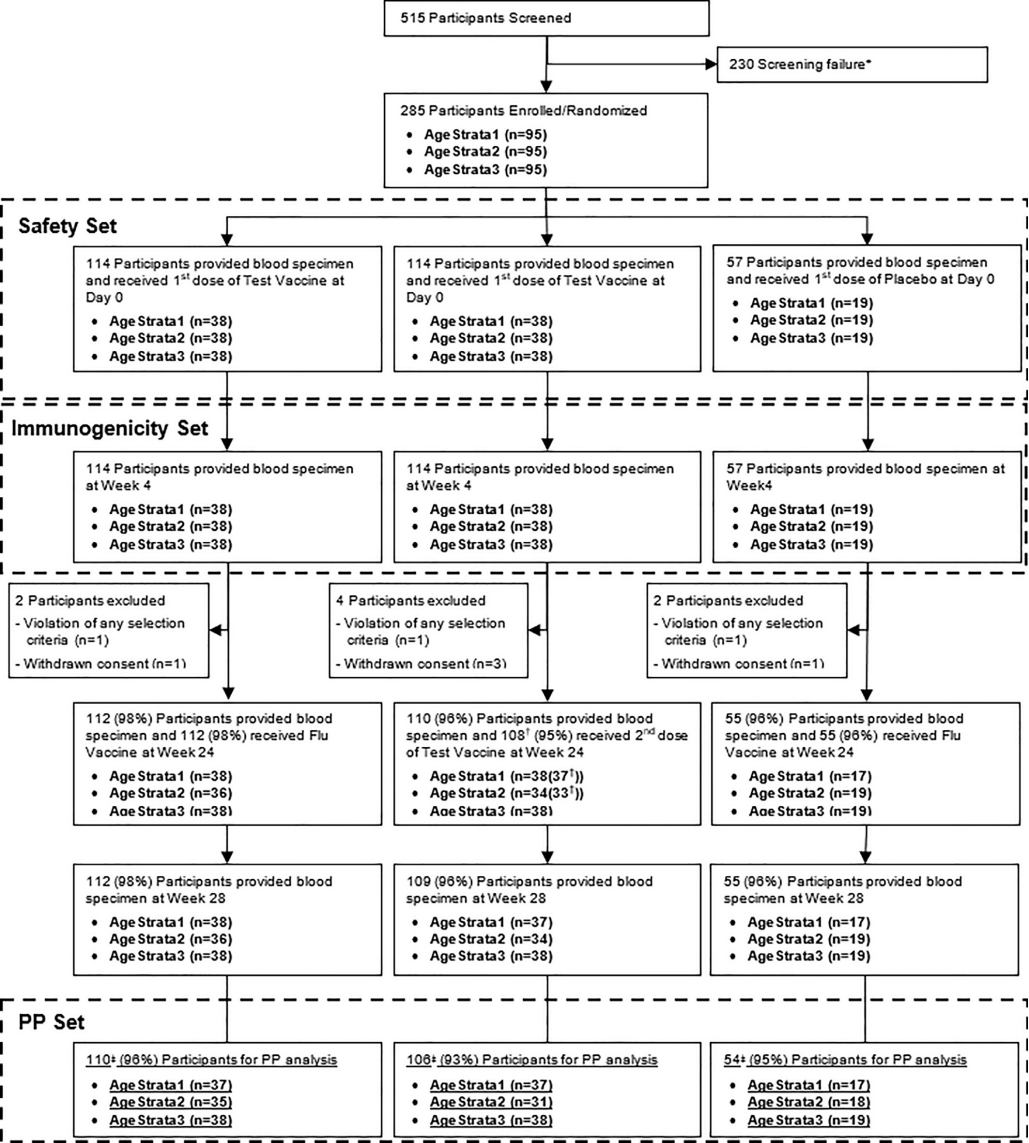
Flow diagram of participant disposition (CONSORT flow diagram). * Screen failure – included participants that had abnormal laboratory values on screening, which included abnormal hematological profile, liver enzymes, renal function test and others; † One participant in each age strata 1 and 2 did not receive the 2nd dose of Test Vaccine; ‡ 5 participants (2 in Vi-DT Single dose Group, 2 in Vi-DT Two-dose Group, 1 in Comparator Group) who had delayed the 2nd vaccination. Four participants did not receive the second dose of Vi-DT – once participant due to violation of the selection criteria (i.e., moved out of study area) and three due to withdrawal of consent.

A total of 515 participants aged 6 to 23 months were screened against predefined enrollment criteria (**Supplementary Table S1**), and 285 were included in the study. The study was conducted at the Research Institute for Tropical Medicine (RITM), Manila, Republic of the Philippines. A total of 285 participants, 114 in the single dose (Group A), 114 in the two-dose (Group B), and 57 in the comparator group (Group C) were enrolled (Figure 1). Parents and legal guardians visiting RITM or nearby health facilities for regular immunizations or medical check-up of their children were invited to participate. Enrolment was aligned with the Philippines routine immunization schedule where the third dose of pentavalent vaccine is given at 9 to 12 months of age along with measles, mumps, and rubella (MMR). Enrolment occurred between April and July 2018.

This clinical trial was conducted in accordance with ICH-GCP E6 (R2) Guidelines per the Declaration of Helsinki, Council for International Organizations of Medical Science (CIOMS) or local country's ethical requirements. The protocol was approved by the Philippines Food and Drug Administration (PFDA), the respective Institutional Review Boards (IRB) of RITM and the International Vaccine Institute (IVI). All parents or legal guardians of the infants and toddlers enrolled in the study participated voluntarily and signed an informed consent. A test of understanding was administered to the parents or legal guardians. Successful completion of the test of understanding was required before participant's parent or legal guardian could sign the informed consent. Investigators did not initiate study procedures before obtaining written informed consent. A copy of the informed consent document was given to the participants’ parent or legal guardian for their records. Confidentiality of all participants was maintained throughout the study.

### 2.3 Randomization and masking

The randomization list was generated by an independent statistician and included sequential numbers unique to each participant. The list for each age stratum (6 to less than 9 months, 9-12 months, and 13-23 months) was generated independently and block randomization was employed to ensure the effective balance between vaccine groups. Since the test and comparator vaccines used in this study had different packaging (presentation), a double-blind design was not possible. However, to avoid bias, an unblinded study staff member staying in a separate room administered the injections. The unblinded study nurse was not involved in the evaluation of vaccine safety and was not allowed to discuss vaccine administration with the investigator and clinical staff. The randomization number of the participant receiving the study vaccine was written on the empty vaccine vial, for records and reconciliation on the vaccine accountability log. Unblinding was permitted only in case of life-threatening condition or serious medical emergency when knowledge of vaccine allocation was judged to be relevant. The need for unblinding did not arise during the study period.

### 2.4 Procedures

Vaccine: The vaccination schedule for the three groups is summarized in Table 1. Briefly, the Vi-DT groups (Groups A and B) received one or two doses of Vi-DT vaccine which includes 25μg of purified Vi polysaccharide (*S.* Typhi C6524) and 37μg of diphtheria toxoid (*Corynebacterium diphtheriae* PW No.8). The stabilizers in the vaccine are 0.620 mg of disodium hydrogen phosphate, 0.152mg of sodium dihydrogen phosphate dihydrate and 4.25mg of sodium chloride. The test vaccine was manufactured, packaged, labelled (Vi-DT) by SK bioscience and stored at 2 - 8°C according to the manufacturer's specifications. The comparator group (Group C) received 0.5mL of 0.9% sodium chloride, packaged as 2- or 5-mL colorless ampoules. For the second Vi-DT dose, 0.25 mL of FluQuadri™, inactivated quadrivalent influenza vaccine (Sanofi Pasteur, France) was given to the comparator (Group C) and single dose groups (Group A).

**Table 1:** Vaccines administered at each vaccination time point and outcome assessment in the three study groups

Allocation	Follow up time following enrolment				
	Week 0	Week 1	Week 4	Week 24	Week 28

Group A (Single dose)	*Strata 1 and 3*: Vi-DT *Strata 2*: Vi-DT plus TRIMOVAX®† *All Strata*: Immediate safety	Safety assessment	Safety and immunogenicity assessment	*All Strata*: FluQuadri™ *All Strata*: Immediate safety	Safety and immunogenicity
Group B (Two dose)				*All Strata*: Vi-DT *All Strata*: Immediate safety a	
Group C (Comparator)	*Strata 1 and 3*: Placebo‡ *Strata 2*: TRIMOVAX®† *All Strata*: Immediate safety			*All Strata*: FluQuadri™ and Immediate safety	

Age Stratum 1 (6 to <9months); Age Stratum 2 (9 to 12 months); Age Stratum 3 (13-23 months).

† TRIMOVAX® – attenuated measles, mumps and rubella (MMR) vaccine was given to children between the ages of 9-12 months.

‡ Placebo for Age Strata 1 and 3 at enrolment was normal saline injection.

Eligible participants enrolled into the study were randomized into one of the three study groups within each age stratum: 6 to less than 9 months, 9-12 months, and 13-23 months. Participants were observed for 60 minutes after each vaccination for reactogenicity assessment. Solicited adverse events were recorded on a diary card during the seven days after each vaccination. Further, unsolicited adverse events (UAE) were recorded over the four weeks following each vaccination whereas serious adverse events (SAE) were monitored during the entire study period. A comprehensive list of relevant medical events was developed to assist in the classification of adverse events (**Supplementary Table S2 to S4**). The UAE were classified into System Organ Class (SOC) and preferred term (PT) using MedDRA (version 21.0) [37].

Blood samples were collected prior to vaccination at day 0 and 4, 24, and 28 weeks post first dose (the 28-week sample was 4 weeks post second dose in Group B) for immunogenicity assessment. An interim analysis was performed after all participants completed week 4 visit (i.e. 4 weeks post first Vi-DT dose, Group A and Group B combined) [36]. During the interim analysis, study personnel remained blinded. This primary analysis was performed when all participants completed week 28 (i.e., 4 weeks post second dose).

### 2.5 Outcomes

The safety and immunogenicity assessments are summarized in Table 1.

Safety and reactogenicity: The primary safety endpoints included local and systemic reactogenicity and adverse events including solicited, unsolicited and serious adverse events. Any event that was not clearly categorized under the classification of severe adverse events, but jeopardized participant safety and comfort was considered as a medically significant event (MSE).

Immunogenicity*:* Anti-Vi IgG was used as an indicator of immunogenicity of Vi-DT and measured by in-house ELISA as previously described [38]. Briefly, poly-L-lysine (1 μg/well) (Sigma, USA) in PBS was precoated prior to Vi coating (0.2 μg/well) (SK bioscience, South Korea) onto 96-well microplate (Thermo Scientific Nunc MaxiSorp, USA). Non-specific binding sites were blocked with 1% bovine serum albumin in PBS. Serially diluted serum samples were added and incubated for 1 h at 37°C. Diluted alkaline phosphatase conjugated mouse anti-human IgG Fc (Abcam, USA) (1:2,000) was added and incubated for 1h at 37°C. The 4-nitropheyl phosphate (Sigma, USA) was added to each well and plates were incubated for 1h at room temperature, followed by the addition of 3M NaOH stop solution. The plate was read at 405nm corrected with a reference wavelength at 490nm. Anti-Vi IgG titers (international unit, IU) were determined based on the international standard serum (NIBSC 16/138). Lower limit of detection for anti-Vi IgG was 0.14 IU/ml (internal validation, unpublished data).

The primary immunogenicity endpoint was the seroconversion rate defined as the proportion of participants with a 4-fold rise in the anti-Vi IgG titer at week 4 as compared to baseline value. The secondary immunogenicity endpoint was defined as the seroconversion rate at week 28 comparing the two-dose regimen of Vi-DT with comparator. GMT of anti-Vi IgG at week 4 post vaccination in the single dose regimen and at week 28 in the two-dose regimen were also compared.

### 2.6 Statistical Analysis

Sample Size: Assuming a 10% dropout rate, the sample size of 228 in the Vi-DT group (114 subjects each in single and two dose Group) versus 57 in the comparator vaccine group provided >99% power to detect superiority of seroconversion rate in the two Vi-DT regimens combined compared to comparator group. The seroconversion rate in the Vi-DT groups and comparator group was assumed to be 95% and 15%, respectively using a one-sided test at 0.0125 significance level. This sample size also provided 90% power for a non-inferiority test of GMT ratio of anti-Vi IgG between single-dose and two-dose regimens, using one-sided test at a 0.025 significance level (85% for significance level of 0.0125). The true GMT ratio was assumed to be 1, the coefficient of variation antibody titer was assumed as 3.0, and the non-inferiority margin of the ratio was assumed as 0.5 (WHO Technical Report Series 924). The seroconversion rate and coefficient of variation of GMT were assumed conservatively based on the phase I data [34].

The intention-to-treat (ITT) analysis included all participants randomized in the study. The safety analysis set was a subset of ITT analysis among those who received at least one dose of investigational vaccine. The immunogenicity analysis set was also a subset of the ITT analysis and included those who received at least one dose of investigational vaccines and had at least one post-baseline immunogenicity assessment.

The per-protocol (PP) analysis set included subjects who did not have any protocol deviations. The Vi-DT groups were assessed against comparator vaccine while comparison of Group A and Group B was a non-inferiority analysis. For superiority testing, significance was 2.5% (one-sided test). For non-inferiority testing, significance level was 2.5% with a one-sided tail and non-inferiority margin of the GMT ratio of 0.5. Analysis of covariance was performed using a Generalized Linear Model (GLM) and used to adjust for baseline titers and age. Imputation was not done for missing immunogenicity data.

The number and proportion of participants with immediate reactions, solicited adverse events at one week and unsolicited adverse events at four weeks after each dose of Vi-DT (Groups A and B combined) versus comparator were calculated for each age stratum. The proportion of participants with at least 4-fold rise of anti-Vi IgG antibody titer at week four compared to baseline was assessed using the Cochran-Mantel-Haenszel (CMH) test. Superiority was assessed using the two-sided 95% confidence interval (CI) for each vaccine group and p-value from the CMH test. The seroconversion rate at week 28 was compared between Group B and Group C using Chi-squared test. Further, the anti-Vi seroconversion rate four weeks after dose two in Group 2 was compared to the comparator vaccine using Chi-square test. The non-inferiority of anti-Vi GMT four weeks post single dose Vi-DT was compared to the GMT four weeks post second dose Vi-DT (at week 28) using the covariance model taking group and strata as covariates after log transformation, after which the data were approximately normally distributed. The GMT was calculated by multiplying all values and taking the *n^th^* root of the average, where n is the number of subjects with available data [1,25]. Vi-DT co-administration with measles, mumps, and rubella vaccines was assessed four weeks following vaccination in the 9-12-month age stratum.

### 2.7 Role of funding source

This study was funded by the Bill & Melinda Gates Foundation (OPP 1115556). The funder of the study did not have any role in data collection, analysis, interpretation, or writing of the manuscript. The corresponding author had full access to all the data in the study and had final responsibility for the decision to submit for publication.

## 3 Results

Among 515 potential participants screened, 285 were enrolled and randomized; most screening failures were due to abnormal laboratory parameters. All enrolled subjects received at least one dose of vaccine and provided one immunogenicity blood sample and were included in immunogenicity analysis set at week 28. A total of 15 participants were excluded from the PP analysis set at week 28: 4 participants from Vi-DT single dose group (Group A), 8 participants from the two-dose group (Group B) and 3 participants from comparator group (Group C). The reasons for exclusion included: missed the second vaccination (n=10) and delayed second vaccination (n=5). The disposition of study participants is described in Figure 1.

*Baseline Characteristics of Study Participants*: The median age of the participants at enrolment was 9 months (interquartile range (IQR): 8-15 months). More than half, 147(51·6%) were female. Age and gender distribution of study participants by age strata and treatment group as well as description of anthropometric assessment and vital signs at baseline and during follow-up are presented in Table 2 and Supplementary Figures S1 to S5. Weight, height, and vital signs of the participants were comparable at enrolment and during the study follow-up (**Supplementary Figures S1** to **S5**).

**Table 2:** Baseline characteristics of study subjects

**Characteristics**		**Vi-DTGroup**			**Comparator Group**	**Total**
		**Any dose**	**single dose**	**two-dose**		
**All ages**		**N=228**	**N=114**	**N=114**	**N=57**	**N=285**
Gender	Male (%)	109 (47.8)	57 (50.0)	52 (45.6)	29 (50.9)	138 (48.42)
	Female (%)	119 (52.2)	57 (50.0)	62 (54.4)	28 (49.1)	147 (51.58)
Age(months)	Mean (SD)	11.56 (5.45)	11.39 (5.27)	11.73 (5.64)	11.35 (5.14)	11.52 (5.38)
	Median (min, max)	9 (6, 23)	9 (6, 23)	9 (6, 23)	9 (6, 23)	9 (6, 23)
**6 to less than 9 months**		**N=76**	**N=38**	**N=38**	**N=19**	**N=95**
Gender	Male (%)	32 (42.1)	18 (47.4)	14 (36.8)	11 (57.9)	43 (45.26)
	Female (%)	44 (57.9)	20 (52.6)	24 (63.2)	8 (42.1)	52 (54.74)
Age(months)	Mean (SD)	6.83 (0.87)	6.76 (0.88)	6.89 (0.86)	6.79 (0.71)	6.82 (0.84)
	Median (min, max)	7 (6, 8)	6 (6, 8)	7 (6, 8)	7 (6, 8)	7 (6, 8)
**9 to 12 months**		**N=76**	**N=38**	**N=38**	**N=19**	**N=95**
Gender	Male (%)	39 (51.3)	21 (55.3)	18 (47.4)	9 (47.4)	48(50.53)
	Female (%)	37 (48.7)	17 (44.7)	20 (52.6)	10 (52.6)	47 (49.47)
Age(months)	Mean (SD)	9.25 (0.64)	9.26 (0.69)	9.24 (0.59)	9.42 (0.96)	9.28 (0.71)
	Median (min, max)	9 (8, 12)	9 (9, 12)	9 (8, 11)	9 (9, 12)	9 (8, 12)
**13 to 23 months**		**N=76**	**N=38**	**N=38**	**N=19**	**N=95**
Gender	Male (%)	38 (50.0)	18 (47.4)	20 (52.6)	9 (47.4)	47 (49.47)
	Female (%)	38 (50.0)	20 (52.6)	18 (47.4)	10 (52.6)	48 (50.53)
Age(months)	Mean (SD)	18.59 (3.24)	18.13 (3.21)	19.05 (3.25)	17.84 (3.24)	18.44 (3.24)
	Median (min, max)	19 (13, 23)	19 (13, 23)	20 (13, 23)	19 (13, 23)	19 (13, 23)

SD – Standard Deviation; min- minimum; max – maximum

*Serious adverse events*: A total of 13 SAEs were reported within the 28-week follow-up period – 10 in the Vi-DT groups (5 in each of Group A and Group B) and 3 in the comparator group. Only one of the SAEs occurred within four weeks of the first dose of Vi-DT, a febrile convulsion which occurred in Group A within AgeStrata1 (6 to less than 9 months). There were no SAEs in Groups B and C within the four weeks of first dose (**Supplementary Table S5**).

Two SAEs occurred within four weeks following second dose of Vi-DT among Groups B and C within AgeStrata2 (9-12 months) – a case of gastroenteritis in Group B and a febrile convulsion in Group C. No SAE was reported from Group A within the four weeks post dose two of Vi-DT.

The remaining 10 SAEs occurred within 4-24 weeks of the first dose of Vi-DT – 4 cases of pneumonia (1 in Group A, AgeStrata1; 1 in Group A, AgeStrata2; and 1 each in Group A and C, AgeStrata3); 2 cases of febrile convulsion (1 each in Group B, AgeStrata1; and Group B, AgeStrata3); 3 cases of gastroenteritis (1 in Group A, AgeStrata2; 1 in Group B, AgeStrata1; and 1 in Group C, AgeStrata3); and 1 case of frontal abscess in Group B, AgeStrata1. None of the SAEs were judged to be associated with the investigational product (IP).

*Immediate reactions*: One participant from Group B, 9-12 months age stratum, experienced multiple immediate reactions (erythema, fever, and hypersensitivity) post first dose of Vi-DT, which led to omission of the second dose of Vi-DT and exclusion from the PP analysis. The reaction was of mild severity and resolved without any sequelae. No immediate reactions were reported post second dose in any group. The immediate reactions did not differ significantly in frequency between groups (p=0·47) and were not considered clinically significant either since most presented mild local irritation (Supplementary Table S6).

*Solicited Adverse Events*: A detailed description of solicited AEs is presented in Table 4 and Supplementary Tables S7 to S9. Seven days post first dose, 25·9% (28·1% in Group A and 23·7% in Group B) had solicited AEs in the Vi-DT groups as compared to 19·3% in the comparator group (p =0·43). The most frequent solicited systemic AE reported across age strata within the Vi-DT group was ‘fever’ while ‘pain/tenderness’ the most frequent local AE. In the comparator group, ‘diarrhea’ was most frequent solicited systemic AE. Solicited AEs were also assessed seven days post second dose. In the Vi-DT group, 11·4% (16·1% in Group A and 6·5% in Group B) had solicited AEs while 9·1% in the comparator group had AEs; the differences did not reach statistical significance, neither were they considered as clinically significant differences.

*Unsolicited Adverse events*: There was no difference between groups in the frequency of unsolicited AEs within the 4 weeks post first dose: 61·4% in the Vi-DT group and 68·4% in the comparator group had unsolicited AE (p=0·34). Even though the incidence of UAE was not statistically different within groups, the 6 to less than 9 months age stratum in the Vi-DT group had a significantly lower rate of unsolicited AE (57·9% vs. 63·2% in the other age strata) .

Within four weeks post second dose, the frequency of unsolicited AE was not significantly different: 32·7% in the Vi-DT group and 27·3% in the comparator group (p=0·51). Details of the proportion and distribution of unsolicited AEs is presented in Table 5 and Supplementary Tables S10 and S12.

*Medically Significant Events*: Overall, a total of 52 children (22.8%) had a medically significant event (MSE). A higher occurrence of MSEs was observed in the comparator group, which was marginally significant (p=0·05). Detailed description of MSE is provided in Supplementary Tables S13 and S14.

**Figure 2:**
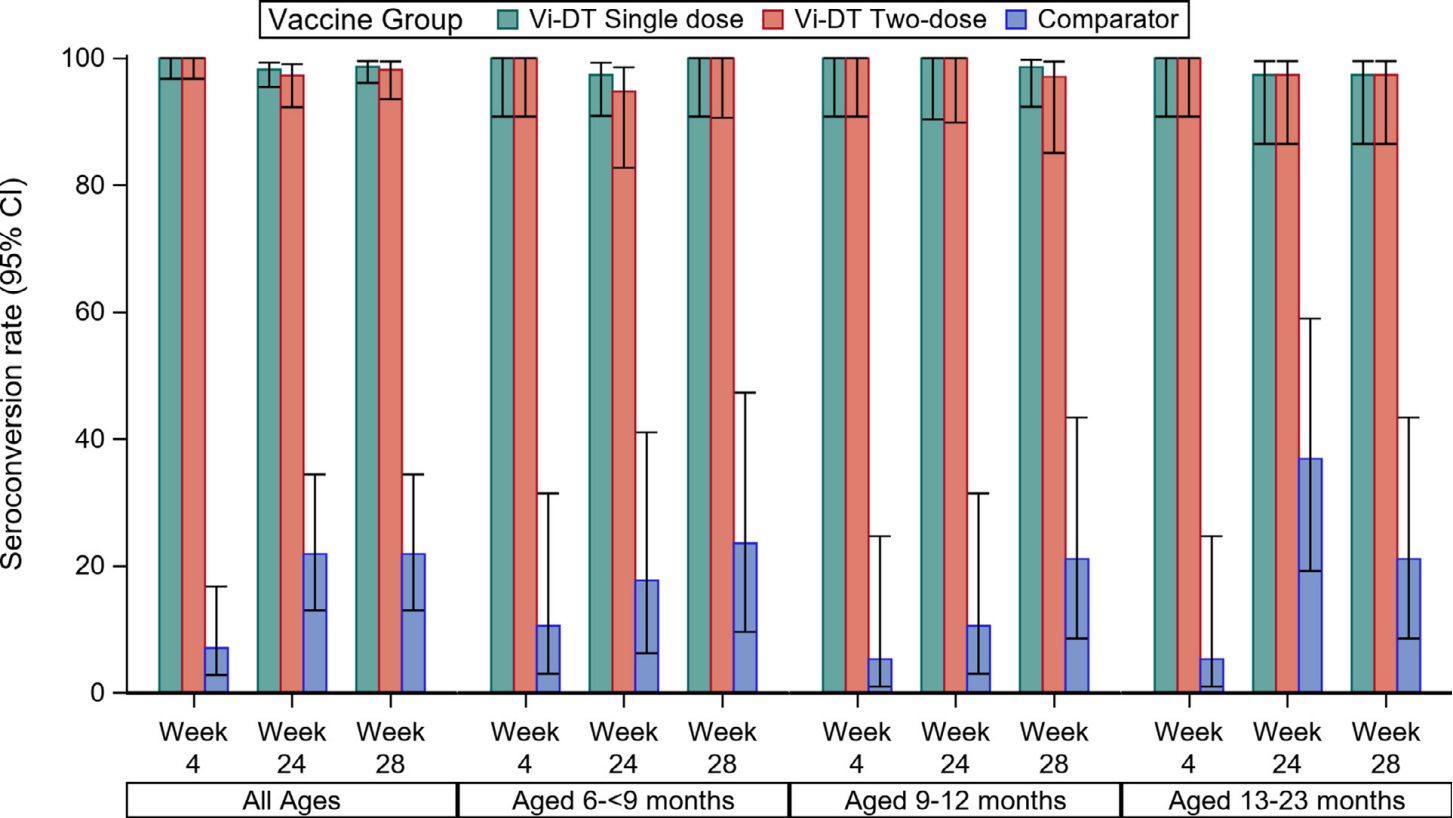
Seroconversion rate by vaccine group, age strata and follow-up time point – Immunogenicity Analysis Set. Seroconversion was defined as 4-fold rise in the anti-Vi IgG titer at week 4 as compared to baseline value. The bars represent the seroconversion rate by age group at different follow up time points; the error bars represent the 95% confidence interval for the point estimate of seroconversion.

*Seroconversion using Immunogenicity Analysis Set*: The seroconversion rate was higher in the Vi-DT groups than in comparator across all age strata. Four weeks post first dose, 100% (95%CI 98·3-100·0) of subjects in Vi-DT Groups combined showed a 4-fold rise in anti-Vi IgG titer versus baseline, while 7% (95%CI 2·8-16·7) of comparator recipients had a 4-fold rise (p < 0.0001). Four weeks post second dose (28 weeks post first dose), seroconversion rates in Groups A and B were 99·1%(95%CI 95·1-99·8) and 97·3% (95%CI 92·3-99·1) respectively versus 21·8% (95%CI 13·0-34·4) in the comparator. Comparing single- and two-dose Vi-DT regimens, there was no difference in the seroconversion rate 28 weeks after first dose (p=0·55). Seroconversion rates four weeks post dose one and four-weeks post dose two were not significantly different between Groups A and Group B (p=0·15) (Figure 2 and Supplementary Tables S15 and S16).

*Seroconversion using Per Protocol Population*: Similar to the findings from the immunogenicity analysis set, in the PP set, almost all participants in the Vi-DT groups seroconverted at four weeks post each dose, which was significantly higher than that in the comparator group (p <0·0001) (Supplementary Tables S17 and S18 and Figure S6).

**Figure 3:**
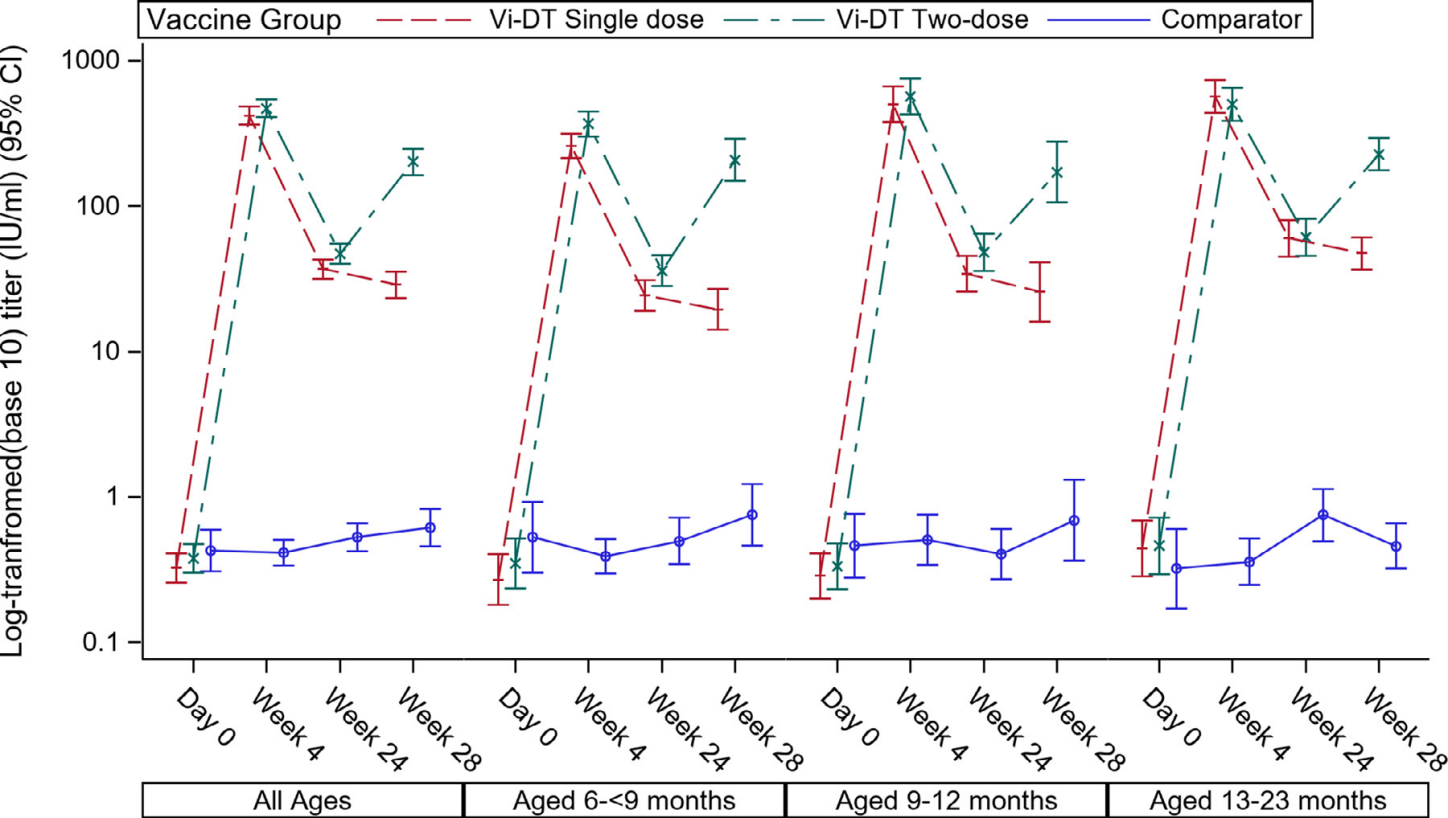
GMT of anti-Vi IgG response by vaccine group, age strata and follow-up time point– Immunogenicity set. The line represents the GMT (IU/mL) values at each follow up time points and the error bars represent the 95% Confidence Interval.

*Geometric mean titers using Immunogenicity Analysis Set*: Pooling first dose Vi-DT titers for both the single- and two-dose Vi-DT groups yields a GMT post-first dose of 444.4 (95%CI 401·7-491·6). In the single dose group, by week 28 this had decreased to a GMT of 28·7 (95%CI 23·4-35·3), while in the two-dose group, 4 weeks after the second dose, the GMT was 201.3 (95%CI 163·4-247·9). The GMT four weeks post single dose Vi-DT was non-inferior to the GMT four weeks post second dose (Table 3). The GMT values by age strata and follow-up time points are shown in Figure 3, Table 3, and Supplementary Table S19.

**Table 3:** GMT of Anti-Vi IgG response for overall age strata (Immunogenicity Set)

**Vi-DT dose**	**Time point**	**Vi-DT Group**											**Comparator Group**			**P-value**†
		**Any dose**			**Single dose**				**two-dose**							
		N	GMT^a^ (95% CI)	SD	N	GMT^a^ (95% CI)	SD	N		GMT^a^ (95% CI)	SD	N		GMT^a^ (95% CI)	SD	
First Dose	Day 0	228	0.35 (0.30, 0.41)	1.20	114	0.32 (0.26, 0.41)	1.12	114		0.38 (0.30, 0.47)	1.28	57		0.43 (0.31, 0.59)	1.42	–
	Week 4	228	444.38 (401.70, 491.60)	0.68	114	420.03 (364.16,484.49)	0.69	114		470.14 (407.60,542.29)	0.67	57		0.41 (0.34, 0.50)	1.16	–
Second Dose	Week 24	222	41.54 (37.13,46.48)	0.77	112	36.82 (31.47,43.08)	0.80	110		46.99 (40.10,55.06)	0.72	55		0.53 (0.42, 0.66)	1.27	–
	Week 28	221	74.98 (62.21,90.38)	1.36	112	28.70 (23.37,35.25)	0.86	109		201.28 (163.41,247.92)	1.04	55		0.61 (0.46, 0.82)	1.60	<.0001^[^^1^^]^

N – total number tested; 95% CI – 95% confidence interval; SD – Standard deviation

a Geometric Mean Titers (unit: IU/ml); [1] GMT of Anti-Vi IgG ELISA Response at Week 28 (Single-dose vs• Two-dose);

† P-values for comparison of GMTs was adjusted for age strata in the model; The ratio (95% CI) of GMT of Anti-Vi IgG ELISA Response at Week 4 of single-dose group vs. Week 28 of two-dose group is 2•08 (1•66, 2•61)

**Table 4:** Proportion of subjects with solicited AE by severity (Safety Analysis Set)

**Solicited AE occurrence**		**Vi-DT Group**						**Comparator Group** **(N=57)**		
		**Any dose(N=228)**		**single dose(N=114)**		**two-dose(N=114)**				
		**Number of AEs**	**Number of Participants (%)**	**Number of AEs**	**Number of Participants (%)**	**Number of AEs**	**Number of Participants (%)**	**Number of AEs**	**Number of Participants (%)**	**P-value**†
**Solicited AE** (day 0 to day 7)		**158**	**59 (25.88)**	**64**	**32 (28.07)**	**94**	**27 (23.68)**	**32**	**11 (19.30)**	
Severity:	Mild	131	54 (23.68)	55	28 (24.56)	76	26 (22.81)	28	11 (19.30)	0.7323
	Moderate	26	19 (8.33)	9	9 (7.89)	17	10 (8.77)	3	2 (3.51)	0.4484
	Severe	1	1 (0.44)	0	0 (0.00)	1	1 (0.88)	1	1 (1.75)	0.4130
	Potentially life threatening	0	0 (0.00)	0	0 (0.00)	0	0 (0.00)	0	0 (0.00)	-
**Solicited AE** (day 168 to day 175)		**55**	**25 (11.36)**	**40**	**18 (16.07)**	**15**	**7 (6.48)**	**15**	**5 (9.09)**	
Severity:	Mild	43	20 (9.09)	31	14 (12.50)	12	6 (5.56)	14	4 (7.27)	0.1700
	Moderate	10	9 (4.09)	7	7 (6.25)	3	2 (1.85)	0	0 (0.00)	0.0583
	Severe	2	2 (0.91)	2	2 (1.79)	0	0 (0.00)	1	1 (1.82)	0.3793
	Potentially life threatening	0	0 (0.00)	0	0 (0.00)	0	0 (0.00)	0	0 (0.00)	-
**Solicited AE** (within 7 days after each vaccination)		**213**	**77 (33.77)**	**104**	**45 (39.47)**	**109**	**32 (28.07)**	**47**	**13 (22.81)**	
Severity:	Mild	174	67 (29.39)	86	37 (32.46)	88	30 (26.32)	42	12 (21.05)	0.2532
	Moderate	36	27 (11.84)	16	16 (14.04)	20	11 (9.65)	3	2 (3.51)	0.0994
	Severe	3	3 (1.32)	2	2 (1.75)	1	1 (0.88)	2	2 (3.51)	0.4678
	Potentially life threatening	0	0 (0.00)	0	0 (0.00)	0	0 (0.00)	0	0 (0.00)	-

AE – Adverse event

† P-values for all ages have been derived using stratified Chi-square (Cochran-Mantel-Haenszel) test stratified by age. (Vi-DT single vs. two-dose Group vs. Comparator Group). However, P-values may not demonstrate the effect of treatment due to the lack of power.

**Table 5:** Proportion of subjects with unsolicited AE by severity (Safety Analysis Set)

**Unsolicited AE occurrence**		**Vi-DT Group**							**Comparator Group** **(N=57)**		
		**Any dose(N=228)**		**single dose(N=114)**		**two-dose(N=114)**					
		**Number of AEs**	**Number of Participants (%)**	**Number of AEs**	**Number of Participants (%)**	**Number of AEs**	**Number of Participants (%)**		**Number of AEs**	**Number of Participants (%)**	**P-value**†
**Within 4 weeks after first dose**		**211**	**140 (61·40)**	**115**	**74 (64·91)**	**96**	**66 (57·89)**		**74**	**39 (68·42)**	
Severity:	Mild	203	135 (59·21)	109	70 (61·40)	94	65 (57·02)		71	39 (68·42)	0.3544
	Moderate	8	8 (3·51)	6	6 (5·26)	2	2 (1·75)		3	3 (5·26)	0.3244
	Severe	0	0 (0·00)	0	0 (0·00)	0	0 (0·00)		0	0 (0·00)	-
	Potentially life threatening	0	0 (0·00)	0	0 (0·00)	0	0 (0·00)		0	0 (0·00)	-
**Within 4 weeks after second dose**		**90**	**72 (32·73)**	**50**	**39 (34·82)**	**40**	**33 (30·56)**	**24**		**15 (27·27)**	
Severity:	Mild	89	72 (32·73)	50	39 (34·82)	39	33 (30·56)	23		14 (25·45)	0.4706
	Moderate	1	1 (0·45)	0	0 (0·00)	1	1 (0·93)	1		1 (1·82)	0.4342
	Severe	0	0 (0·00)	0	0 (0·00)	0	0 (0·00)	0		0 (0·00)	-
	Potentially life threatening	0	0 (0·00)	0	0 (0·00)	0	0 (0·00)	0		0 (0·00)	-

AE – Adverse event

† P-values for all ages have been derived using stratified Chi-square (Cochran-Mantel-Haenszel) test stratified by age. (Vi-DT single vs. two-dose Group vs. Comparator Group). However, P-values may not demonstrate the effect of treatment due to the lack of power

*Geometric mean titers using Per Protocol Population*: The per protocol analysis results were similar to the immunogenicity analysis results (Supplementary Tables S20 and S21, and Figure S7).

*Impact on measles, mumps, and rubella co-vaccination*: We further assessed the impact of Vi-DT on the seroconversion patterns for measles, mumps, and rubella vaccines four weeks following vaccination in the 9-12 months age stratum (n=95)· The seroconversion rate for measles in the Vi-DT group was 94·7(95%CI 87·2-97·9) while it was 100 (95%CI 83·2-100) in the comparator group, which was not statistically different between the groups . Seroconversion for mumps was also comparable [86·8 (95%CI 77·5-92·7)] in the Vi-DT groups versus [89·5 (95%CI 68·6-97·1)] in the comparator group. Similarly, seroconversion rate for rubella was comparable between the Vi-DT groups [98·7 (95%CI 93·0-99·8)] and the comparator group [94·7 (95%CI 75·4-99·1)] (p=0.54) (**Supplementary Table S22**).

## 4 Discussion

We report the results of the first Phase II trial investigating the safety and immunogenicity of the SK bioscience Vi-DT conjugate vaccine in 6 to 23-month-old infants and toddlers with a 28-week follow-up. A phase I study of Vi-DT in 2 to 45-year-old participants conducted in the Philippines was published previously [34]. Single dose Vi-DT and Vi-DT given at 0- and 24-weeks vaccination time points were safe, well tolerated, and immunogenic over 28 weeks of follow-up. Moreover, the immune response to concomitantly administered MMR vaccine was not affected by the Vi-DT administration [36]. In 6 to 23-month-old infants and toddlers we found high levels of seroconversion (100%) after the first dose and persistent seroconversion at week 28 in the 1-dose group. The recipients of a second dose had a corresponding increase in titer, but the second dose did not result in a higher titer than that was seen after the first Vi-DT dose; which is consistent with Phase I where boosting was not seen when the two doses were given 4 weeks apart and with other typhoid conjugate vaccines [25,34]. There was no difference in the occurrence of SAEs between the vaccine and comparator groups. The overall high rate of unsolicited AE (61·4%) observed in the current study was similar to that reported in the Phase I study among 2 to 5 year-old subjects (54·2%), conducted at the same site in the Philippines [34]. The findings pave the way for the phase III study conduct.

Several studies reported the safety and immunogenicity of Vi-DT and other typhoid conjugate vaccines (TCV) among participants older than two years [27,35,39, 40, 41]. However, there are no data on the safety and immunogenicity of Vi-DT in children younger than two years, except for the recently published study of Vi-DT from Indonesia [39,42]. Thus far, three Vi polysaccharide vaccines conjugated to tetanus toxoid and one conjugated to CRM_197_ as a carrier protein have been licensed in India for use in infants [25,43]. However, PedaTyphi® and Zyvac® have not undergone the WHO prequalification process thereby limiting their use in settings outside of India. The other vaccine, Typbar-TCV® is a WHO-prequalified vaccine and is available for vaccination of persons aged 6 months to 45 years. Even though comparison of our results with other studies is difficult due to differences in laboratory standards and measurements, the high seroconversion rates observed in our study are comparable to findings for Vi-TT and Vi-CRM_197_ typhoid conjugate vaccines in the same age group [8,44]. TCVs including Vi-DT confer limited protection against *S*. Paratyphi, whose clinical features may be indistinguishable from that of *S*. Typhi.

Our intention is to develop an additional, effective typhoid conjugate vaccine for infants and young children, which is consistent with the Strategic Advisory Group of Experts (SAGE) recommendations (17-19 October 2017) [45]. The ideal time for introduction of typhoid vaccine in infants should align with the routine EPI schedule [39]. A single dose regimen is preferable and remains the goal of this vaccine development project [46]. Our findings demonstrate that a single dose of Vi-DT vaccine provided anti-Vi seroconversion rates similar to the two-dose regimen in children under 24 months of age followed for 28 weeks. It will be important to continue to follow this cohort to observe the persistence of anti-Vi responses in order to demonstrate that Vi-DT conjugate vaccine might be considered as an addition to the EPI schedule among young children at risk of typhoid fever [39].

Intriguingly, we observed a modest rise in the seroconversion rate of anti-VI IgG in the comparator group at weeks 24 and 28 (21·8%) as compared to the seroconversion rates at week 4 (7%). While the rise in seroconversion rate in the absence of exposure to Vi-DT could be attributed to the high burden and seasonality of typhoid fever in the Philippines,[47] the finding needs further investigation to fully understand the seasonality of anti-Vi IgG titer levels and potential association with unrecognized *S*. Typhi infection in the Philippines.

In conclusion, our findings show that conjugated Vi-DT vaccine is safe and immunogenic in infants and toddlers. Furthermore, we found the persistence of anti-Vi seroconversion at 28 weeks in the single dose group, and follow-up is ongoing for 2 years between single-dose and two-dose regimens of Vi-DT vaccines. A booster dose at two years is planned for the single-dose group. Large-scale phase 3 studies with the single-dose of Vi-DT have started with the objective of achieving WHO prequalification and improving the supply of TCV, an important vaccine for global health.

## Declaration of Competing Interest

Authors, Dr. Capeding reports grants from International Vaccine Institute, during the conduct of the study., Seon-Young Yang, Ji Hwa Ryu, Inho Cheong, Kyu-Young Shim, Yoonyeong Lee, and Hun Kim are employees of SK BioScience. All other authors declare no conflict to interest.

## Appendix Supplementary materials

Image, application 1

Image, application 2
