# Supplementary material for: Safety and immunogenicity of Vi-DT conjugate vaccine among 6-23-month-old children: Phase II, randomized, dose-scheduling, observer-blind Study
Source: eClinicalMedicine. 2020 Sep 9;27:100540. doi: 10.1016/j.eclinm.2020.100540 (PMC7599314; doi:10.1016/j.eclinm.2020.100540)
Supplement: Supplementary file 1 [file mmc1.pdf]

A Phase II, Randomized, Dose-scheduling, Observer-Blinded Study to Assess the Safety, Reactogenicity and Immunogenicity of Vi-DT Conjugate Vaccine in 6-23-Month old Healthy Filipino Infants and Toddlers

**Protocol Number:**	**IVI T002**
**Principal Investigator:**	Dr. Maria Rosario Capeding<br>Head, Department of Microbiology<br>Research Institute for Tropical Medicine<br>Research Drive, Filinvest Corporate City<br>Muntinlupa City, Metro Manila,<br>The Philippines 1718<br>[redacted]
**Sponsor:**	International Vaccine Institute<br>SNU Research Park, 1, Gwanak-ro,<br>Gwanak-gu, Seoul 08826, Republic of Korea
**Co-sponsor**	SK bioscience<br>310 Pangyo-ro, Bundang-gu,<br>Seongnam-si, Gyeonggi-do, 13494,<br>Seoul, Republic of Korea
**Funding Agency:**	Bill and Melinda Gates Foundation<br>Seattle, WA, USA
**Version Number, Date:**	Version 6.0 29OCT2019

FOR OFFICIAL USE ONLY
Information and data included in this document contain privileged and/or proprietary information, which is the property of the International Vaccine Institute, SK bioscience, and the Research Institute of Tropical Medicine, Manila, The Philippines, and may not be reproduced, published or disclosed to others without written authorization. These restrictions on disclosure will apply equally to all future information, which is indicated as privileged or proprietary.

KEY ROLES

1. Principal Investigator
   Dr. Maria Rosario Capeding
   Head, Department of Microbiology
   Research Institute for Tropical Medicine
   Research Drive, Filinvest Corporate City
   Muntinlupa City, Metro Manila, The Philippines 1718
   [redacted]

2. Senior Sub-Investigator
   Dr. Edison Alberto
   Clinical Reseach Investigator
   [redacted]m

3. Sub-Investigators
   Dr. Katherine B. Buluran
   [redacted]

   Dr. Rachel B. Lawenko
   [redacted]

   Dr. Loudhie Cyd M. Phee
   [redacted]

   Dr. Kathleen Ann Hemedez Perez
   [redacted]

   Clinical Reseach Investigator
   Research Institute for Tropical Medicine
   Research Drive, Filinvest Corporate City
   Muntinlupa City, Metro Manila, The Philippines 1718
   [redacted]

4. Contract Research Organization
   [redacted]

5. Safety Laboratory
   Research Institute for Tropical Medicine

Research Drive, Filinvest Corporate City
Muntinlupa City, Metro Manila, The Philippines 1718

6. IVI Personnel
   i. IVI Medical Monitor
      Dr. Birkneh Tilahun Tadesse
      [redacted]

   ii. Head of Clinical Development & Regulatory
      Dr. Anh Wartel
      [redacted]

   iii. Acting Head of Data Management and Biostatistics
      Deok Ryun Kim
      [redacted]

   iv. Clinical Operations Manager
      Suchada Chinaworapong
      [redacted]

   v. Research Laboratory
      Dr. Jae Seung Yang
      [redacted]

   vi. Head of Quality Management
      Tobin Guarnacci
      [redacted]

TABLE OF CONTENTS

Key Roles....2
List of Abbreviations....7
Statement of Compliance....9
1 SYNOPSIS....10
2 INTRODUCTION....18
2.1 Background....18
3 Vi-DT CONJUGATE VACCINE....23
3.1 Preclinical Data....24
3.2 Clinical Data....24
3.3 Study Rationale....25
3.4 Potential Risks and Benefits....26
3.4.1 Known Potential Risks....26
3.4.2 Known Potential Benefits....27
4 OBJECTIVES....27
5 STUDY DESIGN....28
5.1 Study Endpoints....28
5.2 Overall Design....29
5.3 Measures to Minimize Bias....34
5.3.1 Randomization/Masking Procedures....34
5.3.2 Unblinding of Participants....35
6 STUDY AGENT....35
6.1 Study Agent(s) and Control Description....35
6.1.1 Acquisition....35
6.1.2 Formulation, Appearance, Packaging, and Labeling....35
6.1.3 Preparation....39
6.1.4 Dosing and Route of Administration....39
6.1.5 Dose Adjustments/Modifications/Delays....40
6.1.6 Tracking of Dose....41
6.2 Study Agent Accountability Procedures....41
6.3 Standard of Care....41
6.4 Concomitant Medications, Treatments, and Procedures....41
7 STUDY POPULATION....41
7.1 Strategies for Recruitment and Retention....42
7.2 Consent Procedures and Documentation....42
7.3 Compensation for Participation....43
7.4 Participant Inclusion Criteria....43
7.5 Participant Exclusion Criteria....43
7.6 Study Procedures....44
7.6.1 Screening....44
7.6.2 Enrollment....45
7.6.3 Follow-up Procedures and Visits....46
7.7 Participant Withdrawal or Termination....51
7.7.1 Reasons for Withdrawal or Termination....51
7.7.2 Handling of Participant Discontinuation or Termination....52
7.8 Lost to Follow-Up....53

7.9 Protocol Deviations 53
7.10 Protocol Amendments 54
7.11 Premature Termination or Suspension of Study 54
7.12 End of Study 55
8 LABORATORY PROCEDURES/EVALUATIONS 55
8.1 Clinical Laboratory Evaluations 55
8.2 Specimen Processing, Handling, and Storage 55
8.3 Specimen Shipment 55
8.4 Assessment of Immunogenicity 56
9 ASSESSMENT OF SAFETY 56
9.1 Safety Assessment 57
9.1.1 Definition of Adverse Events (AE) 57
9.1.2 Definition of Serious Adverse Events (SAE) 58
9.1.3 Definition of Unanticipated Problems (UP) 58
9.2 Classification of an Adverse Event 59
9.2.1 Severity of Event 59
9.2.2 Relationship to Investigational Product 61
9.2.3 Expectedness 62
9.3 Time Period and Frequency for Event Assessment and Follow-Up 63
9.4 Reporting Procedures 64
9.4.1 Adverse Event Recording and Reporting 64
9.4.2 Serious Adverse Event Reporting 64
9.4.3 Safety Oversight 65
10 STUDY MONITORING 66
11 STATISTICAL CONSIDERATIONS 67
11.1 Sample Size 67
11.2 Statistical Analysis Plan 67
11.3 Statistical Hypotheses 67
11.4 Analysis Datasets 69
11.5 Description of Statistical Methods 70
11.5.1 General Approach 70
11.5.2 Baseline Descriptive Statistics 70
11.5.3 Safety Analysis 70
11.5.4 Analysis of the Primary Immunogenicity Endpoint(s) 71
11.5.5 Analysis of the Secondary Immunogenicity Endpoint(s) 71
11.5.6 Adherence and Retention Analyses 72
11.5.7 Planned Interim Analysis 72
11.5.8 Additional Sub-Group Analysis 73
11.5.9 Multiple Comparison/Multiplicity 73
11.5.10 Exploratory Analyses 73
12 SOURCE DOCUMENTS AND ACCESS TO SOURCE DOCUMENTS 74
13 DATA HANDLING AND RECORD KEEPING 74
13.1 Data Collection and Management Responsibilities 74
13.2 Study Records Retention 75
13.3 Publication and Data Sharing Policy 76
14 QUALITY ASSURANCE AND QUALITY CONTROL 76
15 ETHICS/PROTECTION OF HUMAN SUBJECTS 77

15.1 Regulatory and Ethical Compliance 77
15.2 Participant and Data Confidentiality 77
15.3. Research Use of Stored Human Samples or Specimes 78
15.4 Future Use of Stored Specimens 78
16 REFERENCES 79
17 APPENDICES 82

LIST OF ABBREVIATIONS

AE	Adverse Event
AEFI	Adverse Event Following Immunization
ALT	Alanine Aminotransferase
AST	Aspartate Aminotransferase
BMGF	Bill and Melinda Gates Foundation
BSA	Bovine Serum Albumin
°C	Degree Celsius
CI	Confidence Interval
CIOMS	Council for International Organizations of Medical Sciences
CMH	Cochran-Mantel-Haenszel test
CRF	Case Report Form
DT	Diphtheria Toxoid
eCRF	Electronic Case Report Form
EDTA	Ethylenediaminetetraacetic Acid
ELISA	Enzyme Linked Immunosorbent Assay
EPI	Expanded Program on Immunization
FAS	Full Analysis Set
GCP	Good Clinical Practices
GCLP	Good Clinical Laboratory Practices
GMP	Good Manufacturing Practice
GMR	Geometric Mean Ratio
GMT	Geometric Mean Titer
HIV	Human Immunodeficiency Virus
IB	Investigator's Brochure
ICF	Informed Consent Form
ICH	International Council for Harmonization
IgG	Immunoglobulin G
IL	Interleukin
IRB	Institutional Review Board
IVI	International Vaccine Institute
MOP	Manual of Procedures
MMR	Measles-Mumps-Rubella
μg	Microgram
N	Number
nm	Nanometer
PBS	Phosphate Buffered Saline
PFDA	Philippines Food and Drug Administration
PHP	Philippine Peso
PI	Principal Investigator
PP	Per Protocol
QC	Quality Control
RITM	Research Institute for Tropical Medicine

SAE	Serious Adverse Event
SAP	Statistical Analysis Plan
SBA	Serum Bactericidal Antibody
SMC	Safety Monitoring Committee
SOE	Schedule of Events
SUSAR	Suspected Unexpected Serious Adverse Reaction
Vi	*Salmonella* Typhi Capsular Polysaccharide
Vi-DT	Diptheria Toxoid Conjugated Vi-Polysaccharide Vaccine
Vi-PS	*Salmonella* Typhi Capsular Polysaccharide Vaccine
Vi-rEPA	*Pseudomonas aeruginosa* exotoxin A Conjugated Vi-Polysaccharide Vaccine
Vi-TT	Tetanus Toxoid Conjugated Vi-Polysaccharide Vaccine
WHO	World Health Organization

STATEMENT OF COMPLIANCE

The study will be conducted according to the protocol and in compliance with International Council for Harmonization (ICH) Good Clinical Practice (GCP), Belmont Principles, CIOMS guidelines, Declaration of Helsinki and other applicable regulations in the Republic of the Philippines (Food and Drug Administration of the Republic of the Philippines) and sponsor requirement. The Principal Investigator will ensure that no deviation from, or changes to the protocol will take place without prior agreement from the sponsor and documented approval from the Institutional Review Boards (IRBs), except where necessary to eliminate an immediate hazard(s) to the trial participants. The protocol, informed consent form(s), recruitment materials, and all participant materials will be submitted to the IRBs for review and approval. All identified study personnel will be trained to perform their roles and will carry out their responsibilities in accordance with ICH GCP guideline and clinic site SOPs. Roles and responsibilities of study staff are presented in the Manual of Procedures.

I agree to ensure that all staff members involved in the conduct of this study are informed about their obligations in meeting the above commitments.

Principal Investigator: ______________________________
Print/Type Name

Signed: ______________________________ Date: ____________________
Signature

1 SYNOPSIS

**Name of the Sponsor:** International Vaccine Institute (IVI)
**Name of Investigational Product:** Vi-DT
**Name of Active Ingredients:** Diphtheria Toxoid Conjugated Vi-Polysaccharide Typhoid Vaccine
**Title of Study:** A Phase 2, Randomized, Dose-scheduling, Observer-Blinded Study to Assess the Safety, Reactogenicity and Immunogenicity of Vi-DT Conjugate Vaccine in 6-23-Month old Healthy Infants and Toddlers.
**Protocol Number:** IVI T002
**Study Center(s):** Research Institute for Tropical Medicine (RITM), Alabang, Muntinlupa City, Manila, The Philippines
**Principal Investigator:** Dr. Maria Rosario Capeding, RITM<br><br>**Senior Sub-Investigator:** Dr. Edison Alberto, RITM<br><br>**Data Management and Biostatistics Scientist:** Dr.Yun Chon, IVI<br><br>**Research Laboratory Scientist :** Jae Seung Yang, IVI<br><br>**Study Medical Monitor**: Dr. Arijit Sil

**Study Period (years/months)**<br><br>Estimated date first participant enrolled: April 2018<br>Estimated date last participant enrolled: July 2018<br>Estimated duration of the trial: 30 months	**Phase of development:** 2
**Study Hypothesis**<br><br>We seek to establish immunogenicity of the single dose and two-dose regimens by testing that the single dose and two-dose regimens are superior to a group receiving EPI vaccines or placebo and to compare the single dose and two-dose regimens by testing that the single dose regimen is non-inferior to the two-dose regimen. We will assess immunogenicity for all groups at weeks 4, 24, and 28, and at weeks 60, 96 and 100 for single dose and weeks 60 and 96 weeks for two-dose recipients. The sample size calculation is based on the hypotheses of the immunogenicity endpoints. We will also assess the safety and reactogenicity of Vi-DT by analyzing safety data after each vaccination and between first and second doses.	

Objectives

Primary

- Assess and describe the safety and reactogenicity of Vi-DT
- Assess and compare anti-Vi seroconversion rate 4 weeks post dose one [of combined one and two-dose regimens] of Vi-DT to comparator group

Secondary

- Assess and compare immunogenicity 4 weeks post dose 2 of the two-dose regimen to comparator group
- Assess and compare immunogenicity between week 4 (post dose 1) in single dose regimen and week 28 (4 weeks post dose 2) in the two-dose regimen
- Assess and describe immunogenicity of the two regimens at week 28, corresponding to 4 weeks post dose 2 of the two-dose regimen and 28 weeks post dose 1 of the single dose regimen
- Assess and describe immunogenicity between week 4 (post dose 1) and week 28 (4 weeks post dose 2) in the two-dose regimen
- Assess and describe immunogenicity between week 24 (dose 2) and week 28 (4 weeks post dose 2) of the two-dose regimen
- Assess and describe immunogenicity at week 60 in single dose and two dose regimen
- Assess and describe immunogenicity between boost time point (week 96) and 4 weeks post boost (week 100) in the single dose regimen.
- Assess and describe immunogenicity between week 96 in the single dose regimen comparatively to the two-dose regimen.

Exploratory

- Assess and describe serum bactericidal assay (SBA) titers and seroconversion rates 4 weeks post last Vi-DT dose of each regimen (single vs. two doses), and at weeks 96 and 100 of the single dose regimen and at week 96 in the two-dose regimen.
- Assess and describe possible immunological interference of Vi-DT with MMR vaccine in children of 9-12 months of age.

Methodology

This is a randomized, observer-blinded Phase 2 study in healthy infants and children 6-23 months of age at the time of the first vaccine dose. The purpose of this study is to assess the safety and immunogenicity of the Vi-DT in this age group. The Vi-DT vaccine will be administered at 25 µg either as a single dose, or two doses 6 months apart.

Eligible participants enrolled into the study will be randomized into one of the three study groups within each age strata of 6 to less than 9 months, 9 to 12 months and 13 to 23 months. Subjects will be excluded from the study if they present abnormal lab values at screening. Participants will be observed at the study site for 60 minutes after each vaccination and safety assessment

recorded. Solicited adverse events will be recorded on a diary card during 7 days after each vaccination. Unsolicited adverse events will be recorded during the 4 weeks after each vaccination and between the two-doses study visits. Serious adverse events will be recorded during the entire study period. With the exception of designated study site personnel responsible for vaccine administration, study investigators, study nurse, and those assessing clinical outcomes, and data analysts will be blinded to vaccine allocation until all participant samples at week 28 will have been analyzed for immunogenicity.

Blood samples will be collected at baseline prior to vaccination and at 4, 24 and 28 weeks for immunogenicity assessment. An interim analysis will be performed after all participants complete week 4 visit post first Vi-DT dose (single dose and two-dose regimen combined) in order to help expediting the Phase 3 planning. This interim analysis will be done in a way so that the study and study personnel remain blinded to the allocation of vaccine and placebo. The primary analysis will be performed when all participants complete week 28 visit. Immunogenicity and safety data up to week 28 will be included in the primary analysis.

After all study groups will be unblinded after week 36, single dose and two-dose Vi-DT recipients will continue to be followed up. At week 60, blood samples will be collected for immunogenicity. At week 96 (month 24), the single dose group (Group A) will be given a booster dose of Vi-DT. Blood samples will be collected for immunogenicity (prior vaccination) at week 96 and 4 weeks post vaccination at week 100. The two-dose recipients will also have blood samples collected at week 96. The final analysis will be perfomed after all partcipants complete their scheduled study visits at week 100 or early if discontinued from the study.

Estimated Number of participants to Enroll

A total of 285 participants aged 6 to 23 months will be enrolled in this study, 114, 114 and 57 participants will be randomized to either the single dose, two-dose Vi-DT regimens or placebo/comparator group, respectively within age strata. Three age strata will be 6 to less than 9 months, 9 to 12 months and 13 to 23 months. In each age strata, a minimum number of 80 participants will be enrolled. We will allow the 9-12 months old children to receive Measles-Mumps-Rubella (MMR) vaccine concomitantly with Vi-DT vaccine and descriptive analysis of immune response to MMR only and to MMR and Vi-DT vaccines will be performed to assess the possible immunological interference with MMR vaccine.

Criteria for Inclusion/Exclusion

Inclusion Criteria

In order to be eligible to participate in this study, any individual must meet the following criteria:

- Healthy infants and children 6-23 months of age at enrollment
- Birth weight ≥ 2500 g
- ≥ 37 weeks of pregnancy or judged to be full-term by the midwife or birth attendant

- Parents aged 18 years and above and legal guardians aged 21 years and above as per

the legal authorization in the Philippines, who have voluntarily given informed consent

- Parents/Legal Guardians willing to follow the study procedures of the study and available for the entire duration of the study

Exclusion Criteria

An individual who meets any of the following criteria will be excluded from participation in this study:

- Child with a congenital abnormality
- Subject with abnormal routine biological values at screening
- Subject concomitantly enrolled or scheduled to be enrolled in another trial
- Acute illness, in particular infectious disease or fever (axillary temperature ≥37.5°C), within three days prior to enrolment and vaccination
- Known history of immune function disorders including immunodeficiency diseases, or chronic use of systemic steroids (>20 mg/day prednisone equivalent for periods exceeding 10 days), cytotoxic or other immunosuppressive drugs
- Child with a previously ascertained or suspected disease caused by *S.* typhi
- Child who have had household contact with/and or intimate exposure to an individual with laboratory-confirmed *S*. typhi.
- Known history or allergy to vaccines or other medications
- Know history of allergy to eggs, chicken protein, neomycin and formaldehyde
- History of uncontrolled coagulopathy or blood disorders
- Mother has known HIV infection or other immune function disorders
- Any abnormality or chronic disease which in the opinion of the investigator might be detrimental for the safety of the subject and interfere with the assessment of the study objectives.
- Child whose parents or legal guardian planning to move from the study area before the end of study period

Investigational Product, Dosage and Mode of Administration

Test Vaccine

Vi polysaccharide typhoid vaccine conjugated with Diphtheria toxoid proteins (Vi-DT), manufactured by SK bioscience (Republic of Korea).

- Dose formulation: 25 µg/0.5 mL, presented in single dose glass vials
- Mode of Administration: First dose 0.5 mL by intramuscular injection in the left anterolateral thigh; Second dose 0.5mL by intramuscular injection in the left deltoid.
- Storage Conditions: +2-8°C

Comparator Vaccines

Placebo (0.9% sodium chloride isotonic solution)

- Manufacturer: EuroMed Inc,
- Dose: 0.5 mL, intramuscular injection in the anterolateral aspect of the left thigh

- Storage condition; protected from light, +2-8°C without freezing
- Please refer to package insert: Appendix 1

FluQuadri™ (Split viron, inactivated quadrivalent Influenza vaccine)

- Manufacturer: Sanofi Pasteur
- Dose: Single injection, 0.25 mL (half dose of adult presentation), intramuscular injection in the left deltoid. Participants, who have not been vaccinated for flu before, will receive a second dose of FluQuadri™ at least 4 weeks post first dose (as per manufacturer's recommendation).
- Storage condition: protected from light, +2-8°C
- Please refer to package insert: Appendix 2

TRIMOVAX[$] (Attenuated measles-mumps-rubella vaccine)

- Manufacturer: Sanofi Pasteur
- Dose: 0.5mL, subcutaneous injection in the deltoid
- Storage condition: protected from light, +2-8°C
- Please refer to package insert: Appendix 3

[$]In case of unavailability of Trimovax from Sanofi Pasteur, a similar vaccine from alternative manufacturer will be used (e.g., Priorix from GSK)

Immunization schedule

Group	Number of Vaccinees	Vaccination Schedule (Weeks)		
		0	24	96***
**A**	114	Vi-DT** 25 µg 0.5 mL	FluQuadri™ * 0.25 mL	Vi-DT 25 µg 0.5 mL
**B********	114	Vi-DT** 25 µg 0.5 mL	Vi-DT 25 µg[†] 0.5 mL	No Vi-DT boost
**C**	57	Placebo**	FluQuadri™ * 0.25 mL	N/A

* A second dose will be administered at least 4 weeks later as per manufacturer's recommendation
** Children aged 9 to 12 months will receive MMR vaccination as per the recommendations in the Philippines.
*** The booster dose will be administered open-labeled to Group A recipients only.
**** Two doses of 0.25mL Fluquadri™ will be provided to Group B participants after unblinding.
[†] 4th dose of DTaP vaccine recommended for age 12-18 months in the Philippines will be administered one month after the second dose of Vi-DT.

Reference Therapy, Dosage, Schedule, and Mode of Administration

Intramuscular route in the left anterolateral muscle of the thigh will be used for first dose of Vi-DT. The second dose of Vi-DT will be given in the left deltoid.

Criteria for Evaluation

Safety Endpoints

- Frequency of local and systemic solicited adverse events during the 7 days after each dose
- Solicited local reactions at the site of injection: Pain, tenderness, erythema/redness, swelling/ induration, pruritus
- Solicited systemic reactions: Fever, lethargy, irritability, vomiting, diarrhea, drowsiness, loss of appetite, and persistent crying, rash, nasopharyngitis.
- Frequency of unsolicited adverse events during 4 weeks after each dose and between the two doses at 0 and 24 weeks
- Frequency of Serious Adverse Events during the entire study period

Immunogenicity Endpoints

- Seroconversion from baseline (day 0), defined as four-fold increase of anti-Vi IgG titer
- Geometric Mean Titers (GMT) of anti-Vi IgG

Exploratory Endpoints

- Seroconversion rate from baseline (defined as four-fold increase) of serum bactericidal antibody assay (SBA) 4 weeks post Vi-DT vaccination (week 4 for single dose and week 28 for two-dose recipients) and at week 96 of the single dose and two-dose regimens
- Serum bactericidal antibody titers 4 weeks post Vi-DT vaccination (week 4 for single dose and week 28 for two-dose recipients) and at week 96 of the single dose and two-dose regimens
- Measles, mumps, and rubella ELISA antibody titers 4 weeks after MMR vaccination

Statistical Considerations

The statistical analysis will focus on comparisons of single dose and two doses immunogenicity at time points specified in the Schedule of Events compared to comparator as well as between the two regimens.

In addition, to evaluate the safety and reactogenicity of Vi-DT, a descriptive analysis of the safety endpoints including solicited local and systemic reactions, unsolicited AEs and SAEs, for each regimen after each vaccination and between first and second doses will be performed.

Assuming a 10% dropout rate, the sample size of n=114 for Vi-DT groups vs. n=57 placebo/comparator vaccine would provide >99% power to detect the superiority of seroconversion rate in two Vi-DT regimens compared to comparator vaccine where the

seroconversion rate of two Vi-DT regimens and comparator vaccine are assumed as 95% for each Vi-DT regimen, and 15%, respectively using one-sided test at 0.0125 significance level. This sample size will also provide 90% power for a non-inferiority test of GMT ratio of anti-Vi IgG between single-dose and two-dose regimens, using one-sided test at a 0.025 significance level (85% for significant level of 0.0125), the true GMT ratio is assumed as 1, the coefficient of variation on titer of immunogenicity is assumed as 3.0, and the non-inferiority margin of ratio was assumed as 0.5. The seroconversion rate and coefficient of variation of GMT were assumed conservatively based on Phase I data.

Primary Comparison

To demonstrate superiority of single dose (at week 4) of Vi-DT vaccine compared to comparator vaccine

- Anti-Vi seroconversion rate 4 weeks post single dose (at week 4) of Vi-DT vaccine (25 µg) is superior to 4 weeks post comparator group (at week 4)

Secondary Comparisons

- Anti-Vi seroconversion rate 4 weeks post two doses (at week 28) of Vi-DT vaccine (25 µg) is superior to 4 weeks post comparator vaccine (at week 28)

To demonstrate the single dose Vi-DT regimen is non-inferior to the two-dose Vi-DT regimen:

- Anti-Vi GMT 4 weeks post single dose of Vi-DT vaccine (week 4) is non-inferior to 4 weeks post two dose (at week 28) of Vi-DT vaccine using non-inferiority margin of GMR of 0.5

To control studywise type 1 error rate of 0.025 using one sided test, the primary endpoint will be tested using alpha=0.0125. The first secondary endpoint will be tested using alpha=0.0125. The comparison of single dose vs. two-dose regimens would be tested sequentially using alpha=0.025 or 0.0125 depending on the significance of above two hypotheses.

The following secondary endpoints will be tested without adjusting for multiple testing. Since the following secondary endpoints would not be powered in the study, these assessments and descriptions will not be used as decision-making criteria but will contribute to a more detailed knowledge of the immune response induced by the Vi-DT vaccine.

- Seroconversion rate 4 weeks post single dose (week 4) and 4 weeks post two dose (week 28)

- Anti-Vi GMT and seroconversion rates 28 weeks post first vaccination between single dose (week 28) and two doses (week 4 after the last dose)

- Anti-Vi GMT between weeks 4 and 28 for the two-dose regimen

- Anti-Vi GMT and seroconversion rates at week 24 and week 28 of the two-dose regimen

- Anti-Vi GMT and seroconversion rates at week 60 in the single dose and two dose regimens

- Anti-Vi GMT and seroconversion rates at weeks 96 and 100 in the single dose group

- Anti-Vi GMT and seroconversion rates at weeks 96 in the single dose and two-dose regimens

Exploratory Comparisons

- Serum bactericidal GMT and seroconversion rates 4 weeks post Vi-DT between single dose (week 4) and two-dose (weeks 28) regimens and at weeks 60, 96 and 100 of the single dose and weeks 60 and 96 of the two-dose regimens

- Possible immunological interference of Vi-DT to MMR vaccine will be assessed descriptively at week 4.

2 INTRODUCTION

2.1 BACKGROUND

The global burden of typhoid fever is estimated at 21 million cases and 222 000 typhoid-related deaths per year [1] . Typhoid fever is more common in children and young adults than in older people [2]. Worldwide, typhoid fever is most prevalent in impoverished areas that are overcrowded with poor access to sanitation. Incidence estimates suggest that south-central Asia, Southeast Asia, and southern Africa are regions with high incidence of *S*. Typhi infection (more than 100 cases per 100,000 person years) [3,4,5]. Other regions of Asia and Africa, Latin America, the Caribbean, and Oceania have a medium incidence of 10 to 100 cases per 100,000 person years. These estimates, however, are limited by lack of consistent reporting from all areas of the world and are based on extrapolation of data across regions and age groups. Recent data from Africa have revealed that several countries in Eastern and West Africa have rates >100 per 100,000 [6].

Between 1 January and 13 November 2013, 28,224 cases of suspected or clinically diagnosed typhoid fever were recorded in the Philippines with case fatality rate of 0.27%. During the same time period in Regions 6, 7 and 8 and the National Capital Region there were 5,637 suspected or clinically diagnosed and 60 laboratory confirmed cases [7]. Typhoid fever continue to be reported from the Philippines, and during the first 6 months of 2014 there were 10,597 cases of typhoid fever [8]. A total of 27,106 suspected typhoid cases were reported in 2016, which is 9.9% lower than in 2015. Age of cases ranged from less than one month to 98 years. Most of the cases were aged between 5 to 14 years. There were 38 reported deaths (CFR of 0.14%) [9].

The incidence of Tyhoid fever in children under 2 year of age is reported from several endemic countries. In a population-based prospective study conducted to evaluate incidence of typhoid bacterimia in infants and young children in southern coastal Pakistan the incidence of typhoid bacterimia in children less than 2 years of age was 443 cases per 100,000 child years (95%CI 193.8-876.5) and in infants under 12 months of age was 506.4 cases per 100,000 child years (95%CI 160.9-1222.0)[10]. Typhoid fever incidence among children varied by age with significant proportion in under 2 years old. In a study conducted among preschool children from urban slums in Bangladesh, 4% of typhoid fever cases were seen in the first year of life and 27% in 1 to 1.9 years old [11].

In a study conducted in Ghana which assessed variation of salmonella infections by population size, the typhoid fever in infants adjusted incidence rate per 100,000 Person year of observation was 168 (CI 48-590) in urban and 93 (CI26-325) in rural setup [12].

Very recent data (publication in preparation) from the IVI Department of Epidemiology and their large Typhoid surveillance program in Africa show that the incidence in children less than 1 year of age is 5.4 /100,000 PY and 39.4 /100,000 PY for children aged 1 to less than 2 years. Unfortunately, the typhoid surveillance system in the Philippines does not allow to provide such breakdown in younger age groups.

The Strategic Advisory Group of Experts (SAGE) on Immunization1 in a meeting held on 17-19 October 2017 in Geneva, Switzerland recommended the introduction of typhoid conjugate vaccine (TCV) for infants and children over 6 months of age as a single dose in typhoid endemic countries [13].

Etiological agent

Typhoid fever is caused by *Salmonella enterica* serovar Typhi (*S*. Typhi). It is a rod-shaped gram-negative facultative anaerobe bacterium belonging to the Enterobacteriaceae family. Among more than 2,300 closely-related Salmonella serovars recognized, *Salmonella enteritica* serotype Typhi and *Salmonella enterica* serotype Paratyphi A, B & C are pathogenic exclusively for humans. Infection therefore implies contact with infected person or use of contaminated food or water. Non typhoidal salmonella (such as *Salmonella enteritidis* and *Salmonella typhimurium* may also cause sever illness consistent with typhoid fever [14]. Salmonella possesses a flagellar antigen (H), somatic (O) and a surface antigen (Vi). The Vi capsular is superficial antigen overlying antigen. It is present in few serovar, the most important is *Salmonella enteritica* serotype Typhi but also present in *Salmonella enteritica* serotype Paratyphi C and *Salmonella dublin*.

Clinical presentation

Typhoid fever is one of the most common causes of bacteremia in many developing countries [15]. The clinical feature of typhoid fever is that of a sub-acute systemic infection. Classic reports describe the characteristic stages of typhoid fever in untreated individuals with rising fever and bacteremia. However presentation is variable ranging from mild fever to more severe forms such as toxic shock. General symptoms include high grade fever (40^0C) lasting for more

than 3 days, profuse sweating, chills, abdominal pain, altered bowel functions, malaise, myalgia, anorexia, intestinal bleeding and perforation [16]. In a small percentage of cases, the bacteria may also colonize the gall bladder, leading to a chronic carrier state. Although the disease is known widely, typhoid fever is still often confused with other acute febrile illnesses such as malaria, typhus and dengue fever, even with the use of laboratory diagnosis.

Pathogenesis

Susceptible human hosts are infected upon consumption of food or water contaminated with *S*. Typhi. Inside the small intestine, *S.* Typhi attach to epithelial cells, penetrates the mucosal epithelium to reach the lamina propria through enterocytes and M cells, the domelike epithelial cells that cover Peyer's patches. In the lamina propria, *S*. Typhi triggers an influx of macrophages and dendritic cells that ingest the bacilli but do not generally kill them. Some remain within macrophages of the small intestinal lymphoid tissue. Other typhoid bacilli are drained into mesenteric lymph nodes where there is further multiplication and ingestion by the macrophages. Eventually, there is a release of tumor necrosis factor-alpha, interleukin-2 (IL-2), IL-6, and other inflammatory cytokines by the mononuclear cells. After reaching the blood circulation via the thoracic duct, the bacteria are filtered from the circulation and sequestered inside the phagocytic cells of the liver, spleen, and bone marrow [17]. Replication within the endothelial system is the hallmark of typhoid fever and is responsible for the clinical finding of prostration, generalized sepsis and hepato-splenomegaly. Some individuals will contain the orgamnism within the gastrointestinal system and do not become systematically ill but have persistent *S.* Typhi carriage [18].

Typhoid control and prevention

Most of the typhoid cases are effectively treated with antibiotics, although the case fatality rate remains at about 1%. Improvement in sanitary infrastructures and implementation of hygienic practices can reduce the typhoid disease burden as seen in most developed countries. However, the development of adequate infrastructures for improved water and sanitation requires large and long-term investments, and is therefore a distant goal for the impoverished populations. Instead, increased population and limited opportunities in rural areas has resulted in urbanization and increased population density, the major risk factor for typhoid transmission. Basic health education such as hand washing and proper food handling is also known to be effective in reducing typhoid fever. Although typhoid fever can be effectively treated with

antibiotics, growing rates of antibiotic resistance in many countries are making this treatment option increasingly more difficult and costly.

Though a vaccine against typhoid fever was developed and used in the early 20th century, typhoid vaccine development received attention in the early 1960s, when *S.* Typhi strains resistant to chloramphenicol were isolated. As a result, among many candidates, two vaccines, one oral, and one injectable, were licensed in 1990. Today, there are enough evidences that typhoid fever vaccines are efficacious, effective under public health conditions, and have an impact on the incidence for the benefit of larger population. WHO has recommended that countries consider the use of typhoid vaccines for high-risk groups and populations, and for outbreak control [1]. It is therefore essential to consider a comprehensive approach that combines targeted vaccination of high-risk populations as a short- to medium-term prevention measure, along with longer term solutions of water and sanitation improvements and improved living standards [19]. In endemic countries, control of typhoid would require implementing immunization for young children and incorporating typhoid vaccine in the EPI.

There are currently two WHO-recommended vaccines for protection againist *S.* Typhi: live oral vaccine strain Ty21a and parentral Vi polysaccharide vaccines.

Live, Oral Ty21a Vaccine

The Ty21a vaccine consists of a mutant strain of Salmonella Typhi Ty2 that was isolated after chemical mutagenesis and has a galE- and Vi-negative phenotype. It is supplied in enteric -coated capsules, or Liquid suspension (lyophilized vaccine + buffer mixed with water upon use). Immune response to the vaccine starts 14 days after vaccination, which is mediated by mucosal (IgA), serum (IgG), and cell-mediated antibodies. The vaccine has showed no booster effect. It has shelf life of 14 days at +25°C.

The overall protective efficacy for a three-dose regimen ranged between 67% and 80% in large-scale efficacy trials, conducted in 1980s in Chile [20]. The most common adverse events reported with Ty21a were mild and transient gastrointestinal disturbances, followed by general symptoms such as fever. This vaccine is licensed for use in persons 2 years and above for the liquid formulation and 5 years and older for the capsule formulation.

Parenteral Vi Polysaccharide Vaccine

The parenteral subunit Vi polysaccharide vaccine (ViPS) was developed from wild type *S.* Typhi strain Ty2 on the basis of non-denatured purification of the Vi polysaccharide at the National Institute of Health (US). The ViPS vaccine is given as a single dose and was found to confer, overall, 64–72% protection for 17–21 months and 55% over 3 years [21]. The ViPS vaccine is well tolerated and safe. The most common side effects are pain, redness and induration at injection site, and fever. The Vi capsular polysaccharide synthesized by *S*. Typhi is an important virulence determinant and the ability to produce antibodies against Vi is a critical component in the host's defense against infection by *S*. Typhi. ViPS vaccine was found to be poorly immunogenic in children 2-5 years and not immunogenic in children < 2 years of age. This vaccine continues to be the most common vaccine in use in high endemic countries and was systematically used in routine public health programs in China, Vietnam and Nepal, The vaccine is widely available in the private market in China, India, Pakistan and many other endemic countries. Few counties such as Sri Lanka use this vaccine in their public health program through targeted approach; otherwise, no other country adopted the vaccine in their immunization program.

Typhoid conjugate vaccines

The scientists at the US National Institute of Child Health and Disease (NICHD) have developed the method that used the heterobifunctional cross-linking reagent, N-succinimidyl-3-(2-pyridyldithio)-propionate (SPDP) or adipic acid dihydrazide (ADH) as linkers to bind Vi to proteins. Using a nontoxic recombinant protein that is antigenically identical to *Pseudomonas aeruginosa* exotoxin A as a carrier protein, the resultant conjugates (Vi-rEPA) were more immunogenic in mice and juvenile Rhesus monkeys than the Vi alone. In contrast to the T-independent properties of the Vi alone, conjugates of this polysaccharide with several medically relevant proteins induced booster responses in mice and in juvenile Rhesus monkeys. This synthetic scheme was reproducible, provided high yields of Vi-protein conjugates, and was applicable to several medically relevant proteins such as diphtheria and tetanus toxoids [22]. In a randomized, vaccine-controlled study of infants in Vietnam, Vi-rEPA was safe, elicited protective levels of IgG anti-Vi, and was compatible with EPI vaccines. These data show that Vi-rEPA can be added to the routine immunization of infants in countries where typhoid fever is prevalent [23].

The Novartis Vaccines Institute for Global Health, Siena, Italy, is developing a typhoid conjugate vaccine (Vi-CRM197) using Vi from *Citrobacter freundii* WR7011 conjugated to the, CRM197, a non-toxic mutant of the diphtheria toxin [24]. Phase I and II clinical trials were conducted in European adults. In the phase I trial, single dose of Vi-CRM197 was compared with Typherix® in 50 European volunteers between 18 to 40 years of age. Phase II trial was a dose-ranging design (12.5, 5.0 or 1.25 µg) with 88 European participants between 18 to 40 years of age in which all Vi-CRM197 doses were at least as immunogenic as unconjugated Vi [25]. Recently, phase II studies have been completed in India, Pakistan, and The Philippines in four different age-groups: 18 to 45 years; 24 to 59 months; 9 to 12 months; and infants aged 6 weeks with each group having 40 participants. Novartis Vaccines Institute for Global Health (NVGH, now Scalvo Behring Vaccines Institute for Global Health, a GSK company) since then has transferred the technology to Biological E, Hyderabad, India.

With the technology initially transferred from US NIH, Biomed Pvt Ltd. in India developed a conjugate vaccine using Tetanus Toxoid as the carrier protein. This product was tested in a clinical trial in 169 participants > 12 weeks with a comparison group (Vi) of 37 children > 2 years[26] . The results from this study were compared with the NIH study in Vietnam and it was reported that there was four fold or greater rise in antibody titer of each group on ELISA which was statistically equivalent to Vi-rEPA. Based on the results of this study, this product was submitted for licensure and was licensed for more than 3 months of age in 2008 in India.

Similarly, Bharat Biotech in Hyderabad, India also developed typhoid conjugate vaccine using Tetanus Toxoid as the carrier protein with Vi polysaccharide. This vaccine was tested in children (2 to 17 years) for safety, immunogenicity and dose ranging (15 µg versus 25 µg/0.5 mL). There was no significant difference between two doses of 25 µg/ 0.5 mL and two doses of 15 µg/0.5 mL. In the next clinical trial, comparative assessment of the immunogenicity of Vi-TT versus the polysaccharide vaccine was done in 981 participants (6 months to 45 years old). The investigators found 4-fold seroconversion rates in each treatment arm at 6 weeks post vaccination [27]. After 2 years of follow-up, the anti-Vi titers were in the study arm as compared to comparator arm. Based on these results, Bharat Biotech received marketing authorization for Typbar-TCV in India in 2013 as a single dose indication for all aged 6 months and above

3 VI-DT CONJUGATE VACCINE

The Vi-DT Vaccine is a conjugate typhoid vaccine in which purified Vi polysaccharide derived from Salmonella Typhi C6524 is conjugated to Diphtheria Toxoid (DT) as the carrier protein. The Vi-DT vaccine to be used in this trial contains 25 µg of Typhoid antigen (Vi) in the form of Vi-Diphtheria Toxoid conjugate, provided in 0.5 mL in a single dose container.

3.1 PRECLINICAL DATA

Pre-clinical immunogenicity and toxicity studies were conducted to assess immunogenicity and ensure safety of Vi-DT in animal models. A first immunogenicity study of Vi-DT was conducted in mice. Typhoid Kovax (Vi-polysaccharide typhoid vaccine) marketed by Korea Vaccine Co., Ltd. was used as a comparator. All animals that received Vi-DT showed higher immune responses than those elicited by the comparator. Serum antibody titers at week 6 were higher than at week 2, demonstrating a booster effect. The antibody titers were maintained up to week 10. SK bioscience also used guinea pigs and rabbits for immunogenicity testing. The result showed that Vi-DT elicited higher immune responses than those elicited by the comparator in both animal species.

Toxicology studies for Vi-DT with single and repeat dose in mice, respiratory system toxicity in mice and cardiovascular toxicity in female beagle dogs were done at MPI Research (Mattawan, MI, USA). Study results indicated that both single and four repeat intramuscular doses (days 1, 15, 29 and 43) of Vi-DT to mice did not result in mortality, clinical or macroscopic observations, or elicit any changes in body weight, food consumption, neurobehavioral measures, or respiratory function. Also respiratory system toxicity and cardiovascular toxicity studies in mice and beagle dogs did not indicate any toxicity with Vi-DT.

3.2 CLINICAL DATA

A first-in-human Phase 1 trial was conducted in the Philippines to assess the safety and immunogenicity of Vi-DT Conjugate Vaccine compared to Vi-Polysaccharide (Typhim Vi®, Sanofi Pasteur) Typhoid Vaccine in healthy Filipino adults and children. The protocol was approved by the RITM and IVI IRBs and by the Philippines FDA. Informed consent was obtained from all participants.

A total of 144 participants were recruited in Manila, The Philippines, and randomized equally (N=72 in each group) to Test (Vi-DT) and Comparator (Typhim Vi®) group within each stratum of adults, adolescents and young children (N=48 in each strata). There was no significant difference in age and gender among test and comparator group. Male and female participants were 66% and 34%, respectively. The median age was 26 years (18-45) in adults, 11 years (6-16) in adolescents and 4 years (2-5) in children.

No SAE was reported in either group. No participant was discontinued from the study due to AE. All solicited and unsolicited AEs are mild or moderate in both arms with the exception of a 4-year old girl, in Test group with grade 3 fever that resolved without sequel. The proportions of participants with solicited AEs in Test and Comparator groups were respectively 54.17% and 50% in adults, 37.5% and 45.8% in adolescents and 25% and 25% in children. The proportions of participants with unsolicited AEs in Test and Comparator groups were respectively 50% and 45.8% in adults, 37.5% and 45.8% in adolescents, and 79.17% and 70.83% in children. The majority of solicited AEs in adults were pain, tenderness and headache; in adolescents pain and tenderness; and in children pain and fever.

All participants in Test group showed seroconversion (defined as 4-fold increase of anti-Vi IgG titer) after the first and second doses while 97% of participants in Comparator group. Test group showed about 4-fold higher GMT than in the Comparator group. SBA servoconversion rates were significantly higher in the Test group than in the Comparator group post first and second doses (71% vs. 52.17% and 70.4% vs. 51.39%, respectively). SBA GMT were also significantly higher in the Test group than in the Comparator group post first and second doses (526.56 vs. 271.26 and 586.5 vs. 222.97, respectively). Anti-DT responses were significantly higher in the Test group than in the Comparator group with a 26-fold rise post first dose compared to baseline values in the Test group while a 0.93-rise was observed in the Comparator group.

3.3 STUDY RATIONALE

Vaccination with Vi polysaccharide has been shown to protect individuals from typhoid fever but Vi vaccine has several limitations. Vi is poorly immunogenic and revaccination does not elicit an anamnestic response [28]. There is increasing evidence of *S*. Typhi infection in younger children supporting the need for vaccinating children against typhoid in the first year of life [29]. However, the response to Vi polysaccharide in children under two years of age is poor and consequently

Vi vaccines are not licensed for use in this at risk age group. Currently available live-attenuated vaccine Ty21a is only available in capsule format that can be administered to children 5 years and above, requires multiple doses, and must be stored in strict cold chain [30]. The limitations of Vi vaccines can be overcome by conjugation of the Vi to a carrier protein. Immune responses to bacterial capsular polysaccharides are generally T-cell independent and lack affinity maturation, poor antibody subclass switching and the inability to generate memory. Conjugation of the polysaccharide to a protein carrier converts the immune response to T-cell dependent, which is characterized by affinity maturation, subclass switching and induction of memory [31].

Vi-DT typhoid conjugate vaccine, Vi-Polysaccharide conjugated to Diphtheria Toxoid (DT), is designed to obtain license for use in children 6 months of age and above, followed by WHO prequalification for procurement by Gavi in different regions of the world, and for use in older children and adults.

The results of first-in-human Phase I trial of Vi-DT show that the vaccine is safe and immunogenic in age groups 2-45 years. These promising results pave the way in pursuing the clinical development of Vi-DT in younger children of 6 to 23 months on age.

While a single dose of vaccine would be highly preferable and remains the ultimate goal, we will compare regimens of single dose and two doses administered 6 months apart. This interval would allow the administration of Vi-DT contemporarily to EPI vaccines recommended in the country and avoid additional vaccination visits of parents and children to the health centers. This study will address the duration of the immune response of the single dose regimen at week 96 (Vi-DT booster dose) and at week 100 and of the two regimens at week 96.

3.4 POTENTIAL RISKS AND BENEFITS

3.4.1 KNOWN POTENTIAL RISKS

Vi conjugate vaccines already licensed in India are found to be safe, and without safety concern [27]. Also the Vi-DT vaccine components, Vi Polysaccharide and DT, are licensed and in use for a long time with established safety profile. In the course of the Phase 1 trial conducted in the Philippines at RITM, Vi-DT was not associated with any severe adverse event or serious adverse event. Any vaccine could cause an anaphylactic reaction, though such reactions are rare. Expected local and systemic reactions include injection site pain, tenderness, erythema, redness, induration, swelling, pruritus, and systemic reactions such as fever, lethargy, irritability,

vomiting, diarrhea, drowsiness, loss of appetite, and persistent crying. The MMR vaccine may also induce rash and naso-pharyngitis. These side effects are expected to be mild or moderate and transient and resolving spontaneously without sequelae.

3.4.2 KNOWN POTENTIAL BENEFITS

Study participants may receive no direct benefit from study participation. They will however have access to their medical records. Findings of medical concern will be referred for appropriate care and treatment. Compared with polysaccharide vaccines, conjugate vaccines are usually more immunogenic and better at inducing long term memory responses, especially in young children < 2 years of age. The potential benefits to vaccinated participants are substantial, since typhoid is endemic in several parts of the Philippines. Typhoid affects infants and young children and can lead to death if not treated promptly with appropriate antibiotics. The use of an effective vaccine in this age group will contribute to the prevention and control of typhoid and prevent death from typhoid fever.

Knowledge of vaccine-induced immune responses in infants and toddlers in a population with endemic typhoid will be useful for future use of this vaccine administered with EPI vacines to prevent and control typhoid.

4 OBJECTIVES

Primary

- Assess and describe the safety and reactogenicity of Vi-DT
- Assess and compare anti-Vi seroconversion rate 4 weeks post dose one [of combined one and two-dose regimens] of Vi-DT to comparator group

Secondary

- Assess and compare immunogenicity 4 weeks post dose 2 of the two-dose regimen to comparator group
- Assess and compare immunogenicity between week 4 (post dose 1) in single dose regimen and week 28 (4 weeks post dose 2) in the two-dose regimen
- Assess and describe immunogenicity of the two regimens at week 28, corresponding to 4 weeks post dose 2 of the two-dose regimen and 28 weeks post dose 1 of the single dose regimen

- Assess and describe immunogenicity between week 4 (post dose 1) and week 28 (4 weeks post dose 2) in the two-dose regimen
- Assess and describe immunogenicity between week 24 (dose 2) and week 28 (4 weeks post dose 2) of the two-dose regimen
- Assess and describe immunogenicity at week 60 in single dose and two dose regimen
- Assess and describe immunogenicity between boost time point (week 96) and 4 weeks post boost (week 100) in the single dose regimen.
- Assess and describe immunogenicity between week 96 in the single dose regimen comparatively to the two-dose regimen.

Exploratory

- Assess and describe serum bactericidal assay (SBA) titers and seroconversion rates 4 weeks post last Vi-DT dose of each regimen (single vs. two doses), and at weeks 96 and 100 of the single dose regimen and at week 96 in the two-dose regimen.
- Assess and describe possible immunological interference of Vi-DT with MMR vaccine in children of 9-12 months of age.

5 STUDY DESIGN

5.1 STUDY ENDPOINTS

Safety Endpoints

- Frequency of local and systemic solicited adverse events during the 7 days after each dose
    - Solicited local reactions at the site of injection: Pain, tenderness, erythema/redness, swelling/ induration, pruritus
    - Solicited systemic reactions: Fever, lethargy, irritability, vomiting, diarrhea, drowsiness, loss of appetite, persistent crying, rash and nasopharyngitis
- Frequency of unsolicited adverse events during 4 weeks after each dose and between the two doses at 0 and 24 weeks
- Frequency of Serious Adverse Events during the entire study period

Immunogenicity Endpoints

- Seroconversion from baseline (day 0), defined as four-fold increase of anti-Vi IgG titer

- Geometric Mean Titers (GMT) of anti-Vi IgG

Exploratory Endpoints

- Seroconversion rate from baseline (defined as four-fold increase) of serum bactericidal antibody assay (SBA) 4 weeks post Vi-DT vaccination (week 4 for single dose and week 28 for two-dose recipients) and at week 96 of the single dose and two-dose regimens
- Serum bactericidal antibody titers 4 weeks post Vi-DT vaccination (week 4 for single dose and week 28 for two-dose recipients) and at week 96 of the single dose and two-dose regimens.
- Measles, mumps and rubella ELISA antibody titers

5.2 OVERALL DESIGN

The trial design is illustrated in Tables 1, 2, and 3, and detailed below. This is a randomized, observer-blinded, Phase 2 study in healthy infants and toddlers 6-23 months of age at the time of the first vaccine dose. The purpose of this study is to assess the safety and immunogenicity of the Vi-DT in this age group. The Vi-DT vaccine will be administered intramuscularly at 25 µg either as a single dose, or two doses 6 months apart.

Participants will be evaluated at screening for general health, medical history, and undergo a physical examination and laboratory evaluations. Eligible participants enrolled into the study will be randomized into one of the three study groups within each age strata of 6 to less than 9 months, 9 to 12 months and 13 to 23 months.

The Vi-DT vaccine will be administered at 25 µg either as a single dose, or two doses 6 months apart. Single dose group (Group A) will receive Vi-DT at Day 0 and FluQuadri™ at week 24. The two-dose group (Group B) will receive Vi-DT at Day 0 and week 24. The comparator group (Group C) will receive placebo at Day 0 and FluQuadri™ at week 24. Children aged 9 months to 12 months will receive Vi-DT or placebo alongside MMR vaccine, which is the recommended EPI vaccine in the Philippines for this age group.

Safety and tolerability will be assessed with clinical monitoring. Participants will be observed at the study site for 60 minutes after each vaccination and safety assessment recorded. Adverse events including vaccine-related reactions will be solicited with the aid of diary cards and

interview with parents for 7 days after each vaccination. The information gained from the review of the diary card and the interview of parents/legal guardian will be recorded in the CRF. In addition adverse events will be documented at each clinical encounter after the first vaccination through weeks 28 or Visit 8. Adverse events will be continued to be documented at week 36 (visit 9), week 60 (visit 10) and week 96 (visit 11). For Group A, a booster dose of Vi-DT will be given at week 96. Adverse events will be recorded 7 day post booster dose at week 97 (visit 12) using diary card and at week 100 (visit 13). Serious adverse events will be recorded during the entire study period.

Blood samples will be collected at intervals specified in the schedule of events to assess immunogenicity, serum bacteriocidal assay (SBA) and the possible Vi-DT MMR vaccine interaction. Blood samples will also be collected to assess boosting for the single dose group at week 96 and 4 weeks post vaccination at week 100. The two-dose recipients will also have blood samples collected at week 96.

With the exception of designated study site personnel responsible for vaccine administration, study investigators, study nurse, and those assessing clinical outcomes, and data analysts will be blinded to vaccine allocation.

An interim analysis will be performed after all participants complete week 4 visit post first Vi-DT dose (single dose and two-dose regimen combined). Week 4 safety and immunogenicity data will help expedite the phase 3 planning. This interim analysis will be done in a way to keep study and study personnel blinded to the allocation of vaccine or placebo.

The primary analysis will be performed when all participants complete week 28 visit. Immunogenicity and safety data up to week 28 will be included in the primary analysis.

Single dose and two-dose Vi-DT recipients will continue to be followed up. At week 60, blood samples will be collected for immunogenicity. At week 96 (month 24), the single dose group will be given a booster dose of Vi-DT. Blood samples will be collected for immunogenicity (prior vaccination) at week 96 and 4 weeks post vaccination at week 100. The two-dose recipients will also have blood samples collected at week 96. The final analysis will be perfomed after all participant complete their scheduled study visits at week 100 or if early discontinued from the study.

Rationale for use of Placebo

We seek to demonstrate superiority of Vi-DT to comparator vaccine. In the comparator arm (Group C), placebo will be used for the first vaccination at week 0 for two reasons: 1/ unavailability of other vaccine recommended in the Philippines for certain age groups, e.g., 6 to less than 9 months; 2/ in conformity with study design, a second injection is needed 6 months later to respect the blinding and the number of injections administered at a single time point. There is no recommended vaccine in the Philippines that can be administered 6 months apart for this age group. The use of placebo will allow the simultaneous administration of routine EPI vaccines that children are entitled to receive at a given age.

Justification for the dose of Vi-DT

The dose of Vi-DT to be tested in the Phase 2 study is 25 µg/0.5 mL of Vi polysaccharide. This dose was found to be safe and immunogenic in participants aged 2 years and above in a Phase 1 (IVI T001) conducted in the Philippines.

Previous studies conducted with other typhoid conjugate vaccines concur to use 25 µg/0.5 mL of Vi polysaccharide. Immune response in infants aged 6-8 weeks was less than older age groups when low dose (5 µg) of conjugated typhoid vaccine was used [32]. Single dose and two doses of 25 µg/0.5 mL, and two doses of 15 µg/0.5 mL were tested in a Phase 2b study in age group 2-17 years. Single dose of 25 µg/0.5 mL of Vi-TT conjugated vaccine showed excellent immune response (100% seroconversion) and was found to be safe in infants and young children aged 6-23 months. Based on these results Bharat Biotech carried out a Phase III clinical trial with single dose 25 µg/0.5 mL [27]. The Bharat Vi-TT vaccine is now licensed in India and several other countries and submitted for WHO prequalification.

Similarly, in a dose ranging study, 25 µg, 12.5 µg and 5 µg of conjugate typhoid vaccine Vi-rEPA (Vi polysaccharide conjugated to exoprotein of *Pseuomonas aeruginosa*) conducted in children 2-5 years of age in Vietnam, 25 µg was found to be safe with higher immunogenicity [33]. Further 25 µg Vi-rEPA vaccine safety and immunogenicity study was conducted in infants aged 2, 4, 6 and 12 months along routine EPI vaccines and was found to be safe and immunogenic [23].

Table 1. Immunization Schedule

Group	Number of	Vaccination Schedule (Weeks)

	Vaccinees	0	24	96***
**A**	114	Vi-DT** 25 µg 0.5 mL	FluQuadri™ * 0.25 mL	Vi-DT 25 µg 0.5 mL
**B******	114	Vi-DT** 25 µg 0.5 mL	Vi-DT 25 µg† 0.5 mL	No Vi-DT boost
**C**	57	Placebo**	FluQuadri™ * 0.25 mL	N/A

* A second dose will be administered at least 4 weeks later as per manufacturer's recommendation
** Children aged 9 to 12 months will receive MMR vaccination as per recommendations in the Philippines.
*** Booster dose at week 96 will be administered open-labeled to Group A recipients only.
**** Fluquadri™ 0.25mL will be provided to Group B participants after unblinding after week36
† 4th dose of DTaP vaccine recommended for age group 12-18 months in the Philippines will be administered after one month after the second dose of Vi-DT

After administration of FluQuadri® at the 24th week, the 2018 batches were expired, therefore in the subsequent year (in 2019) vaccination against influenza was provided with two doses of FluQuadri® outside of protocol approved defined window to all enrolled subjects after unblinding post week 36 (i.e. influenza vaccination was administrered during Visit 10 and one month after visit 10 through an unscheduled visit). (please refer to MFR No. 10 dated 26OCT2018) Since week 36 was the last visit of group C subjects, they were provided with FluQuadri® without documentation in the source document as they were no longer part of the study at that point of time.

A number of subjects could not be vaccinated during scheduled visits (during Visit 6) with FluQuadri® due to unresolved AEs and due to the expiration of the FluQuadri® vaccine batch/lot which occurred on 12JAN2019, Delayed subject vaccination was remediated by use of an alternate vaccine e.g. Varivax® . Approval for use of the Varivax® vaccine was granted by IVI IRB on 11MAR2019 and RITM IRB on 13MAR2019 prior to its use. (please refer to MFR No. 14 dated 22 MAR 2019)

Table 2. Schedule of Events

Visit Number	V1	V2	V3	V4	V5	V6	V7	V8	V9

Visit Day	D-7 - 0	D0	D7	D28	D84	D168	D175	D196	D252
**Visit Week**	-1	0	1	4	12	24	25	28	36
**Visit Window**		±1D	±1D	±3D	±3D	±3D	±1D	±3D	±7D
**Vaccination**		**X**				**X**			
Screening	X								
Informed Consent	X								
Inclusion & Exclusion Criteria	X	X							
Verification of eligibility						X			
Medical History	X	X							
Vital Signs	X	X	X	X	X	X	X	X	X
Physical Examination	X	X	X	X	X	X	X	X	X
Enrollment & Randomization		X							
Vaccination		X				X			
Post Vaccination 60 min F/U		X				X			
Solicited AE		X	X			X	X		
Unsolicited AE		X	X	X	X	X	X	X	X
SAE		X	X	X	X	X	X	X	X
Diary Card Provided		X				X			
Diary Card Checked			X	X			X	X	
Concomitant Medications		X	X	X	X	X	X	X	X
Screening Blood Volume	5 mL								
Immunogenicity Blood Volume		3 mL		3 mL		3 mL		3 mL	
Cumulative Blood Volume	5 mL	8 mL		11 mL		14 mL		17 mL	

Table 3. Schedule of Events for late Vi-DT boost

Visit Number		V10	V11	V12*	V13*
**Visit Day**		D420	D672	D679	D700
**Visit Week**		60	96	97	100
**Visit Window**		±7D	±14D	±1D	±7D
**Vaccination**	**Group A**		**X**		
	**Group B**		**No boost**		
Verification of eligibility			X*		
Medical History		X	X		X
Vital Signs		X	X	X	X
Physical Examination		X	X	X	X
Post Vaccination 60 min F/U			X*		
Solicited AE			X*	X	

Unsolicited AE	X	X	X	X
SAE	X	X	X	X
Diary Card Provided		X*		
Diary Card Checked			X	X
Concomitant Medications	X	X	X	X
Immunogenicity Blood Volume	3 mL	3 mL		3 mL
Cumulative Blood Volume	3 mL	6 mL		9 mL

*Only for Group A

5.3 MEASURES TO MINIMIZE BIAS

5.3.1 RANDOMIZATION/MASKING PROCEDURES

The randomization list will be generated by a independent statistician who is not part of the study at IVI. Eligible participants will be assigned to receive single dose, or two doses of Vi-DT Vaccine or comparator vaccine in a 2:2:1 ratio. For the single dose group, flu vaccine will be administrated as second dose for blinding purpose. For the comparator group, placebo and flu vaccine will be administrated as first and second vaccines, respectively. The randomization list will contain sequential numbers unique to each participant and the block randomization process will be employed to ensure an effective balance between the interventions.

Two types of randomization list, one with randomization number only, second with randomization number and vaccine allocation will be prepared. Participants in the study will be randomized into three treatment groups within each age strata: 6 to less than 9 months, 9 to 12 months, and 13 to 23 months. Randomization list “without the treatment allocation” with only numbers will be given to the blinded trial staff, for enrolling the trial participants and assigning them the randomization number. The randomization list “with the vaccine allocation” will be given to the unblinded vaccine administrator (nurse) in a sealed envelope.

Upon enrolment, in order to receive the study vaccine, participants will be sent to the vaccine administrator with their randomization number. The unblinded study nurse located in a different room will administer vaccine(s) to the participant according to the randomization list. The randomization number of the participant receiving the study vaccine will be written on the empty vaccine vial, for record and reconcilliation and on the vaccine accountability log.
Trial staff other than the unblinded study staff will remain blinded to vaccine administration. The unblinded study nurse will not be involved in the evaluation of vaccine safety and will not discuss with the investigator and clinical staff about vaccines administered.

5.3.2 UNBLINDING OF PARTICIPANTS

Unblinding will be considered on a case-by-case basis and only in the case of a health-threatening condition or serious medical emergency when the vaccine allocation is judged relevant for the safety of the participants. The Principal investigator will be provided with the randomization individual envelop with treatment allocation of Test or Comparator vaccine in a sealed evelope. If the Principal Investigator considers necessary to unblind a participant for safety reasons, the PI can break the blind code, confirm the relevant vaccine from the randomization individual envelop, and take actions accordingly. If an accidental unblinding occurs, the unblinding should be documented and recorded. The investigator will inform the sponsor by email or fax.

Study participants will be unblinded after database lock. The database will be locked for the primary analysis after safety data is cleaned and all immunogenicity data at week 28 is made available. After unblinding single dose Vi-DT recipients will be called for further follow-up visits until week 100 and two-dose Vi-DT recipients until week 96.

6 STUDY AGENT

6.1 STUDY AGENT(S) AND CONTROL DESCRIPTION

6.1.1 ACQUISITION

Test vaccine (Vi-DT, NBP618) manufactured by SK bioscience in the Republic of Korea will be shipped to study site in Manila, the Philippines. Placebo saline (0.9% sodium chloride) manufacture by Euro-Med will be purchased through local distributor. RITM will procure TRIMOVAX[$], the MMR vaccine (Sanofi Pasteur), placebo and FluQuadri™ (Sanofi Pasteur). Other EPI vaccines recommended for the age of the child will also be administered at the study site. The unblinded nurse will receive the study agents and will be responsible for accounting, storage, handling and administration of the vaccines.

[$]In case of unavailability of Trimovax from Sanofi Pasteur, a similar vaccine from alternative manufacturer will be used (e.g., Priorix from GSK)

6.1.2 FORMULATION, APPEARANCE, PACKAGING, AND LABELING

Test Vaccine

1) Code name: NBP618

2) Manufacturer: SK bioscience Co., Ltd.
3) Ingredient: Purified Vi-polysaccharide conjugated to diphtheria toxoid
4) Appearance: Clear, colorless liquid
5) Dose: 0.5 mL/Vial

Table 4. Test Vaccine Content

Function	Component	Reference	Quantity
Active ingredient	Purified Vi polysaccharide (*Salmonella* Typhi C6524)	In-house	25 μg
Active ingredient	Diphtheria toxoid (*Corynebacterium diphtheria* PW No.8)	In-house	37 μg
Stabilizer	Di Sodium hydrogen phosphate	EP	0.620 mg
Stabilizer	Sodium Dihydrogen Phosphate dihydrate	EP	0.152 mg
Stabilizer	Sodium chloride	EP	4.25 mg

6) Packaging: will be manufactured and packaged by SK bioscience and supplied to the study pharmacy nurse at the clinical site. The Test Vaccine will be labelled by SK bioscience as Test Vaccine (Vi-DT).
7) Labeling: will be labeled as follows, according to Article 75, Paragraph 6 of the Enforcement Regulations of the Pharmaceutical Affairs Act. Test vaccine will be labelled as detailed in Section 5 of Part B: Investigational Medicinal Product Dossier, the template of vial and carton label are as follows:
8) Storage condition: hermetic container, protected from light, +2°C to 8°C without freezing
9) Retest period: 24 months from date of manufacturing

Test Vaccine vial label**:**

Vial label "For Clinical Trial Use Only"<br>Code: NBP618<br>Lot No. : OOOOOO<br>Retest Date: 24 months from the date of manufacture<br>(Mfd. Date: YYYY.MM.DD)<br>Manufacturer: SK bioscience Co., Ltd., L House<br>Address: 150, Saneopdanji-gil, Pungsan-eup, Andong-si,

Gyeongsangbuk-do, Republic of Korea

Box Label:

"For Clinical Trial Use Only"<br>Code: NBP618<br>Lot No. : OOOOOO<br>Storage Condition: Store in hermetic container at 2-8°C; protected from light<br>Manufacturer: SK bioscience Co., Ltd., L House<br>Address: 150, Saneopdanji-gil, Pungsan-eup, Andong-si, Gyeongsangbuk-do, Republic of Korea

Comparator vaccine (first dose at week 0)

Placebo

1) Product name: 0.9% Sodium Chloride
2) Manufacturer: Euro-Med
3) Ingredient: NaCl
4) Appearance: Clear, colorless liquid
5) Dose: 0.5 mL
6) Packaging: 2 mL or 5 mL/ampoule
7) Storage condition: protect from light, keep in the refrigerator, and store at +2°C to 8°C without freezing
8) Retest period: Not applicable

TRIMOVAX$ (Attenuated measles, mumps and Rubella vaccine)

1) Product Name: TRIMOVAX
2) Manufacturer: Sanofi Pasteur
3) Ingredient: Live attenuated measles virus(schwarz strain), mumps virus and rubella virus containing human albumin
4) Appearance: reconstituted vaccine has clear, yellow to purple red appearance
5) Dose: 0.5mL, subcutaneous injection
6) Packaging: Powder, single dose vial
7) Labeling: Keep original label as registered in the Philippines
8) Storage condition: protected from light, stored at +2°C to 8°C
9) Retest: Reconstituted vaccine should be used immediately

[$]In case of unavailability of Trimovax from Sanofi Pasteur, a similar vaccine from alternative manufacturer will be used (e.g., Priorix from GSK)

Comparator vaccine (second dose at week 24)

FluQuadri™

1) Product name: FluQuadri™ (Split viron, inactivated quadrivalent Influenza vaccine)
2) Manufacturer: Sanofi Pasteur
3) Ingredients:
4) Active Ingredients: contains 4 killed influenza virus strains: (Southern hemisphere strains selected by WHO for the 2018-2019 season)
5) Other Ingredients: contains less than 0.5 µg ovalbumin (egg protein) per dose; includes sodium chloride, sodium phosphate dibasic anhydrous, sodium phosphate monobasic anhydrous, water for injection, and traces of formaldehyde and octoxinol-9.
6) Appearance: Clear and slightly opalescent in colour
7) Dose: A single 0.5 mL/syringe,. Children 6-23 months of age will receive half dose (0,25 mL) of the adult dose as per manufacturer's recommendation
8) *Participants who have not been vaccinated for flu before, will receive a second dose of FluQuadri™ at least 4 weeks post first dose (as per manufacturer's recommendation).
9) Packaging: Prefilled syringe (pink syringe plunger rod), 0.5 mL
10) Labeling: keep original label as registered in the Philippines
11) Storage condition: protected from light, keep it in the refrigerator, store at +2°C to 8°C without freezing
12) Retest period: Life time is 12 Months

Varivax®

1. Product name: Varivax®
2. Manufacturer: Merck
3. Ingredients: Varicella Virus Vaccine Live
4. Appearance: When reconstituted clear, colorless to pale yellow liquid
5. Dose: 0.5 mL after reconstitution and is administered by subcutaneous injection
6. Packaging: lyophilized vaccine (package A) and Diluent (package B)
7. Storage: Before reconstitution, store the lyophilized vaccine in a freezer at a temperature between (−50°C and −15°C) and Vaccine stored at 2°C to 8°C which is not used within 72 hours of removal from (−15°C) storage should be discarded.

6.1.3 PREPARATION

The preparation and administration of the vaccines to subjects enrolled into the study will only be done by the unblinded Study Nurse according to the procedures stipulated in this study protocol. The unblinded Study Nurse responsible for vaccine administration is qualified to perform this task under applicable local laws and regulations for the Philippines.

The licensed vaccines and placebo will be prepared before use according to the package insert. For further details please refer to the MOP.

6.1.4 DOSING AND ROUTE OF ADMINISTRATION

Vi-DT manufactured by SK bioscience, Republic of Korea, is formulated to contain purified vi-polysaccharide 25 µg in 0.5 mL per dose. For all children from all groups, the first dose of Vi-DT at week 0 will be administered intramuscularly at 25 µg (0.5 mL) in the antero-lateral aspect of the left thigh and the second dose of Vi-DT at week 24, 25 µg (0.5 mL) intramuscularly in the left deltoid.

FluQadri™ manufactured by Sanofi Pateur and distributed in the Philippines is a split virion, inactivated quadrivalent influenza vaccine which is formulated to contain 4 killed influenza virus strains. The vaccine is supplied as sterile 0.5 mL solution in prefilled syringes. FluQadri™ is administered intramuscularly 0.25 mL per dose on the left antero-lateral aspect of the left thigh for children aged 6 to 12 months and 0.25 mL per dose intramuscularly in the left deltoid muscle for children aged >12 to 23 months as recommended by the manufacturer. A second dose of Fluquadri™ will be given at least 4 weeks apart for participants in group A and C who never received Fluquadri™ vaccine before as per the manufacturer's recommendationand after unblinding after week 36. Participants from Group B will receive Fluquadri™ as per the manufacturers recommendation given at least 4 weeks apart after unblinding after week 36.

Placebo used for this trial is a 0.9% sodium chloride solution, a product of Euro-Med registered in the Philippines. The placebo will be administered in a volume of 0.25 mL intramuscular on the left antero-lateral thigh.
In case the left thigh cannot be used for vaccination due to infection, eczema or injury, the right thigh will be used for vaccination instead. If the left deltoid cannot be used for vaccine administration, the right deltoid will be used instead.

TRIMOVAX$ (Sanofi Pasteur) is administered 0.5 mL subcutaneously in the deltoid for children 9 to 12 months of age as recommended in the Philippines. A booster dose will be given at 12 months for children who received the first MMR vaccination at 9 months and at 15 months for those who received the first vaccination at 10, 11 or 12 months of age. The MMR vaccine will be given concomittently with Vi-DT or placebo for participants aged 9 to 12 months in all study groups. If a participant in age group 9 to 12 months presents to the study site after receiving MMR vaccine elsewhere, the participant will be enrolled but will not be included in the analysis for Vi-DT possible immunological interference with MMR vaccine.

$In case of unavailability of Trimovax from Sanofi Pasteur, a similar vaccine from alternative manufacturer will be administered as per their package insert (e.g., Priorix from GSK)

The tables below summarizes the vaccination administration per group, age range and route of administration.

**Age at enrollment**	**Study group**	**First vaccination**<br><br>**Week 0**	**Route and Administration site**	**MMR route and site**	**Age at second vaccination**	**Second vaccination**<br><br>**Week 24**	**Route and Administration site**
**6<9 months**	A	Vi-DT	IM, left thigh		**12<15 months**	FluQuadri	IM, left deltoid
	B	Vi-DT	IM, left thigh			Vi-DT	IM, left deltoid
	C	Placebo	IM, left thigh			FluQuadri	IM, left deltoid
**9-12 months**	A	Vi-DT	IM, left thigh	SC, deltoid	**15-18 months**	FluQuadri	IM, left deltoid
	B	Vi-DT	IM, left thigh	SC, deltoid		Vi-DT	IM, left deltoid
	C	Placebo	IM, left thigh	SC, deltoid		FluQuadri	IM, left deltoid
**>12-23 months**	A	Vi-DT	IM, left thigh		**>18-29 months**	FluQuadri	IM, left deltoid
	B	Vi-DT	IM, left thigh			Vi-DT	IM, left deltoid
	C	Placebo	IM, left thigh			FluQuadri	IM, left deltoid

**Study group**	**Booster vaccination**<br><br>**Week 96**	**Route and Administration site**
A	Vi-DT	IM, left deltoid

6.1.5 DOSE ADJUSTMENTS/MODIFICATIONS/DELAYS

There is no plan of Vi-DT dose adjustment and modification during the course of study. Fluquadri™ is supplied in prefilled syringe of 0.5 mL. We will use half (0.25 mL) of the 0.5 ML

Fluquadri™ as per the recommendation of the manufacturer for this age group. All vaccines will be administered within the specified window period in the SOE. TRIMOVAX[$] is lyophilized and supplied in vials of single dose. The vaccine will be reconstituted using diluent provided by the manufacturer and administered subcutaneously in the deltoid in a volume of 0.5 mL.

[$]In case of unavailability of Trimovax from Sanofi Pasteur, a similar vaccine from alternative manufacturer will be administered as per their package insert (e.g., Priorix from GSK)

6.1.6 TRACKING OF DOSE

All study agents and MMR and other EPI vaccines administered will be registered in the vaccine administration logbook and CRF by the unblinded Nurse. The unblinded study monitor will ensure timeline adherance for administration of study agent.

6.2 STUDY AGENT ACCOUNTABILITY PROCEDURES

Unused Vi-DT wil be disposed or returned as per the guidance from the manufacturer. All expired vaccines will be disposed as per the site SOP for disposal (See details in the MOP).

6.3 STANDARD OF CARE

- Appropriate medical care and treatment to participants in need during the trial will be provided as per standard of care practice in the Philippines.
- The PI will conduct appropriate medical investigations deemed necessary to evaluate any medical conditions that might arise during the course of the study and ensure that participants receive appropriate care and are referred to appropriate health services as needed.

6.4 CONCOMITANT MEDICATIONS, TREATMENTS, AND PROCEDURES

A prescription medication is defined as a medication that can be prescribed only by an authorized/licensed physician. Medications to be reported in the Case Report Form (CRF) are concomitant prescription medications, over-the-counter medications and supplements.

7 STUDY POPULATION

7.1 STRATEGIES FOR RECRUITMENT AND RETENTION

The targeted sample size is 285: 114 for the single dose Group A, 114 two-dose Group B and 57 for the placebo/ Comparator Group C of children aged 6-23 months. Study staffs will approach parents and legal guardians of children aged 6-23 months between Days -7 to 0 at the health facility. The enrollment venue is the clinical trial site at RITM. Parents with children aged 6 to 23 months visiting health centers for regular immunizations or medical check-up who may be interested in having their child participate in the study, will be asked to go to RITM during the recruitment period. Posters will be utilized as passive information dissemination strategy and will be posted at health centers. Parents /legal Guardians in age catagories: 6 to less than 9 months (infants coming for the third pentavalent vaccine dose), 9 to 12 months (Infants coming for MMR vaccine), and 13 to 23 months will be asked for consent. A minimum number of 80 participants will be enrolled in each age strata. The study will require long-term participation therefore, telephone follow-up reminder calls at Day 3, and every month after week 4 until week 28 for all study groups and until week 96 for single dose and two dose groups and week 100 for the single dose group (Group A) will be conducted (Details are provided in the MOP.)

7.2 CONSENT PROCEDURES AND DOCUMENTATION

Informed consent is a process that is initiated prior to the individual's agreeing to participate in the study and continues throughout the individual's study participation. Extensive discussion of risks and possible benefits of participation will be provided to the parents or legal guardians of the child (called here 'participants'). Consent forms will be IRB-approved prior to their use and the participant will be asked to read and review the document.

The investigator or designated study team member will explain the study to the parents or legal guardians of potential participants and answer any questions that may arise. All participants will receive a verbal explanation in terms suited to their comprehension of the purposes, procedures, and potential risks of the study and of their rights as research participants. The parents or legal guardians of potential participants will have the opportunity to carefully review the written consent form and ask questions prior to signing. Parent or legal guardian of potential participant may wish to discuss the study with family or friends before making any decision as to whether or not to participate in the study and come back later to inform the Principal Investigator or designee of his/her decision. For those individuals who express interest in continuing with the consent process, the Principal Investigator or designee will review the consent form privately in

detail with the parents or legal guardians of potential participants and answer any questions. Before signing the consent, parents or legal guardians of potential participants will be asked to undergo an informed consent process validation to ensure that they fully understand the purpose of the study, procedures, potential risks and their rights in this study.

The participants' parent or legal guardian will sign the informed consent document prior to any procedures being done specifically for the study. The participants' parent or legal guardian may withdraw consent at any time throughout the course of the trial. A copy of the informed consent document will be given to the participants' parent or legal guardian for their records. The rights and welfare of the participants will be protected by emphasizing to parent or legal guardian that the quality of their medical care will not be adversely affected if they decline to participate in this study.

7.3 COMPENSATION FOR PARTICIPATION

Participants will not be compensated for participation in the study. However a prorated reimbursement to the parent or legal guardian for travel expenses and time lost from work will be given. The reimbursement will be a total of PHP 1000 (if from within Muntinlupa City) or PHP 1500 (if from out of Muntinlupa City) for each scheduled visit.

7.4 PARTICIPANT INCLUSION CRITERIA

In order to be eligible to participate in this study, any individual must meet the following criteria:

- Healthy infants and children 6-23 months of age at enrollment as determined by medical history, physical examination and clinical jugment of the investigator
- Birth weight ≥ 2500 g
- ≥ 37 weeks of pregnancy or judge to be full-term by the midwife or birth attendant
- Parents aged 18 years and above and legal guardians aged 21 years and above as per the legal authorization in the Philippines, who have voluntarily given informed consent
- Parents/ legal guardians willing to follow the study procedures of the study and available for the entire duration of the study

7.5 PARTICIPANT EXCLUSION CRITERIA

An individual who meets any of the following criteria will be excluded from participation in this study:

- Child with a congenital abnormality
- Subject with abnormal routine biological values at screening
- Subject concomitantly enrolled or scheduled to be enrolled in another trial
- Acute illness, in particular infectious disease or fever (axillary temperature ≥37.5°C), within three days prior to enrolment and vaccination
- Known history of immune function disorders including immunodeficiency diseases, or chronic use of systemic steroids (>20 mg/day prednisone equivalent for periods exceeding 10 days), cytotoxic or other immunosuppressive drugs
- Child with a previously ascertained or suspected disease caused by *S*. typhi
- Child who have had household contact with/and or intimate exposure to an individual with laboratory-confirmed *S*. typhi
- Known history or allergy to vaccines or other medications
- Know history of allergy to eggs, chicken protein, neomycin and formaldehyde
- History of uncontrolled coagulopathy or blood disorders
- Mother has known HIV infection or other immune function disorders
- Any abnormality or chronic disease which in the opinion of the investigator might be detrimental for the safety of the subject and interfere with the assessment of the study objectives
- Child whose parents or legal guardian planning to move from the study area before the end of study period

7.6 STUDY PROCEDURES

7.6.1 SCREENING

Informed Consent Process

The informed consent process will be conducted as described above. The date of signature of the informed consent will be entered on the CRF.

The screening process will take place during the week prior to enrollment (Days -7 to 0). After written consent is obtained, screening procedures including blood draw will be performed as described in the SOE and MOP. The study staff will motivate the screened eligible child parents/ legal guardian to visit trial site on Day 0 for vaccination. A participant will be enrolled if he/she

meets all inclusion and exclusion criteria and his/her health status is deemed acceptable as determined by medical history, physical examination, laboratory values, and medical judgement of the Principal Investigator. Laboratory screening values as described below under clinical laboratory evaluations will be completed after informed consent is obtained. A study ID card will be provided to each enrolled participant.

Details of visit procedures during screening are as follows:

Visit 1 - Screening visit (Days -7 to 0)

1) Explain study objectives and procedures, risk and benefits to the participant parent or legal guardian.
2) Perform informed consent process validation
3) Obtain written informed consent from the participant parent or legal guardian. Written Informed consent will be obtained prior to performance of any study-specific screening or tests or evaluations.
4) Perform screening procedures within 0 to 7 days prior to first vaccination.
5) Collect blood sample for laboratory evaluation (including hematology and biochemistry).
6) Collect demographics, medical history, vital signs, inclusion and exclusion criteria
7) Schedule participant for enrolment and vaccination visit (V2) at the study center within 7 days after the screening visit. If an eligible participant parent or legal guardian does not come back for V2 within the 7 days after the screening visit, screening procedures must be repeated.
8) Children who do not meet the criteria for participation in this trial because of fever or acute illness that has resolved may be rescreened. Rescreened participants will be assigned the same participant number as for the initial screening.

7.6.2 ENROLLMENT

Informed consent form will be verified by study staff and inclusion and exclusion criteriea will be reviewed. Participant will be randomized to the single dose or two-dose ViDT or placebo/comparator groups into three age stratas of age 6 to less than 9 months, 9 to 12 months, and 13 to 23 months. Participants will be attributed a study number. After randomization, medical history, medication history will be obtained. Vital signs and results from physical examination, growth and development evaluations will be recorded. Blood for Vi ELISA

IgG and serum bactericidal antibody (SBA) measurements will be obtained prior IP administration.

Details of visit procedures during enrollment are described below and in the SOE and MOP:

Visit 2 - Enrollment and First Vaccination (Day 0)

1) Review lab results and collect medical history, perform clinical examination and check concomitant therapies and record in the CRF.
2) Confirm eligibility of participant
3) Perform enrolment, randomization and attribute study number to participant.
4) Perform blood draw for assessment of baseline immune status before vaccination.
5) Perform first vaccination as per instructions of the Manual of Procedures.
6) Monitor participant for 1 hour following vaccination as follows:
   - Local examination of injection site
   - Clinical examination including vital signs, general physical examination before leaving the study center 60 (±5) min post vaccination
   - Record solicited and unsolicited adverse reactions (if any occurring)
7) Schedule next visit to the study center and remind participant parent or legal guardian to bring the diary card at the next visit
8) Participant parent or legal guardian will be instructed to evaluate local and systemic reactions at home for 7 days post immunization (from Day 0 to Day 6 post vaccination day). Diary card will be issued to record adverse events and participant parent or legal guardian will be instructed how to fill in the diary card. A thermometer will be given along with diary card to record fever. Next clinic visit after 7 days will be scheduled. Participant parent or legal guardian will also be instructed to contact investigator/ study staff if needed.

7.6.3 FOLLOW-UP PROCEDURES AND VISITS

Days 0-6

The study staff will contact the participant parent or legal guardian by telephone to remind them and provide assisstance to record local and systemic reactions with the use of the diary card and to remind the date of the next visit day (at the call on Day 3 post first vaccination).

Visit 3 – Safety follow-up visit (Day 7 ± 1 day)

1) Record solicited and unsolicited adverse events since last visit in CRF
2) Verify that the participant parent or legal guardian fills in the diary card correctly
3) Perform clinical examination (including vital signs and general physical exam) and record in CRF
4) Schedule next visit V4 and remind participant parent or legal guardian to contact investigator/ study staff if needed.

Visit 4 – Follow-up visit (Week 4, Day 28 ± 3 days)

1) Check Diary Card and confirm with participant parent or legal guardian before recording observations in the CRF
2) Record any unsolicited AE, SAE and concomitant medications in CRF
3) Perform clinical examination including vital signs, general physical examination
4) Perform blood draw for assessment of immune responses post first vaccination
5) Schedule next visit V5 and remind participant parent or legal guardian to bring the diary card at the next visit

Visit 5 – Follow-up visit (Week 12, Day 84 ± 3 days)

1) Record medically significant unsolicited AE, SAE and concomitant medications in CRF
2) Perform clinical examination including vital signs, general physical examination
3) Schedule next visit to the study center

Visit 6 - Second vaccination visit (Week 24, Day 168 ± 3 days)

1) Verify eligibility of participant using inclusion and exclusion criteria
2) Provide Diary Card and confirm with participant’s parent or legal guardian their understanding of use.
3) Record any medically significant unsolicited AE, SAE and concomitant medications in the CRF
4) Perform clinical examination including vital signs, general physical examination.
5) Perform blood draw for assessment of immune responses post vaccination.
6) Perform second vaccination as per instructions in the MOP
7) Monitor participant for 60 minutes following vaccination as follows:
    - Local examination of injection site

- Clinical examination including vital signs, general physical examination before leaving the study center 60 (+5) min post vaccination
- Record solicited and unsolicited adverse reactions (if any occurring).

8) Participant parent or legal guardian will be instructed to evaluate local and systemic reactions for 7 days post-immunization (from day 0 to 6 post vaccination day). A thermometer will be given to participant parent or legal guardian if the previously given (Visit 2) is not available for any reason. Next clinic visit after 7 days will be scheduled. Participant parent or legal guardian will be instructed about his subsequent appointment. Participant parent or legal guardian will also be instructed to contact investigator/ study staff if needed
9) Remind participant parent or legal guardian to bring the diary card at the next visit

<u>Additional Guidence to site for the subjects who has already used 3 days of window period in Visit 6 (Refer MFR No. 11 dated 28JAN2019)</u>

Study requires 7 days solicited AE information and 28 days unsolicited AE data following each vaccination. Considering this and vaccination is an important event, here even 3 days of window had been used by the subject at Visit 6, we will consider the second vaccination as Day 0 again (hypothetically) and go ahead for capturing solicited and unsolicited adverse events. Thus Visit 6, visit 7 or visit 8 might face minor deviations which will be reported to ethical committee but subjects data will be kept within per protocol set for analysis. However study site should try to readjust other subsequent visits (i.e. Visit 9-D252, Visit 10-D420), so that subsequent deviations will not occur in the study.

Similar situation will arise during Visit 11, when booster vaccination will be administered (only to Group A) and we have to consider that day (hypothetically) as Day 0 and will capture 7 days solicited AE data and 28 days unsolicited adverse events at Visit 12 and Visit 13, respectively.

Visit Number	V1	V2	V3	V4	V5	V6	V7	V8	V9	V10	V11	V12	V13
Visit Day	D -7~0	D0	D7	D28	D84	D168	D175	D196	D252	D420	D672	679	D700
Visit Week	-1	0	1	4	12	24	25	28	36	60	96	97	100
Visit Window		±1D	±1D	±3D	±3D	±3D	±1D	±3D	±7D	±7D	±14D	±1D	±7D
Hypothetically V6 is considered as D0 and we have to go ahead						D0	D7 ±1D	D28± 3D	D252[#]				

Visit Number	V1	V2	V3	V4	V5	V6	V7	V8	V9	V10	V11	V12	V13
**Hypothetically V11 is considered as D0 and we have to go ahead**											D0	D7 ±1D	D28±7 D$

back to initial schedule

$ i.e. close to D700 as much as possible

Days 168-175

The study staff will contact the participant parent or legal guardian by telephone to provide assisstance to record local and systemic reactions with the use of the diary card and to remind the date of the next visit day (at the call on Day 3 post first vaccination).

Visit 7 - Safety follow-up visit (Week 25, Day 175 ± 1 day)

1) Verify that the participant parent or legal guardian fills in the diary card correctly
2) Record solicited and unsolicited adverse events and concomitant medication since last visit in CRF
3) Perform clinical examination (including vital signs, local exam at the site of injection and general physical exam) and record in CRF
4) Schedule next visit V8 and remind participant parent or legal guardian to contact investigator/ study staff if needed.
5) Schedule next visit to the study center and remind participant parent or legal guardian to bring the diary card at the next visit

Visit 8 - (Week 28, Day 196 ± 3 days)

1) Collect Diary Card and check with participant parent or legal guardian before recording observations in the CRF
2) Record any unsolicited AE, SAE and concomitant medications in CRF
3) Perform clinical examination including vital signs, general physical examination
4) Perform blood draw for assessment of immune responses post vaccination

Visit 9 - (Week 36, Day 252 ± 7 days)

1) Perform clinical examination including vital signs, general physical examination
2) Record any medically significant unsolicited AEs and SAE in CRF
3) Ensure CRF is duely completed before the participant parent or legal guardian leave the study center

4) After week 36 visit and after database lock participants will be unblinded and notified of further follow-up if they belong to Group A or Group B or that they have completed the study if they belong to Group C
5) Schedule for the second dose of FluQuadri™ for the single dose (Group A) and Comparator group (Group C). Two dose group (GroupB) will also be scheduled to recieve Fluquadri™ after week 36 visit and unblinding.

Visit 10 - Single dose and two-dose groups follow-up visit (Week 60, Day 420 ± 7 days)

1) Record medically significant unsolicited AE and SAE in eCRF
2) Perform clinical examination including vital signs, general physical examination
3) Perform blood draw for assessment of immune responses post vaccination for single dose and two dose recipients
4) Schedule next visit to the study center

Visit 11 - Single dose and two-dose groups follow-up visit (Week 96, Day 672 ± 14 days)

1) Verify eligibility of participant using inclusion and exclusion criteria
2) Perform blood draw for assessment of immunogenicity (prior vaccination for Group A)
3) Perform booster vaccination for single dose recipients as per instructions of the MOP.
4) Monitor participant for 60 minutes following vaccination (Group A participants only) as follows:
    - Local examination of injection site
    - Clinical examination including vital signs, general physical examination before leaving the study center 60 (±5) min post vaccination
    - Record solicited and unsolicited adverse reactions (if any occurring)
5) Schedule next visit V12 for booster(single) dose reciepients to the study center and remind participant parent or legal guardian to bring the diary card at the next visit
6) This is final study visit for two dose reciepient group and parents/legal guardian for the two dose groups will be notified accordingly.

Participant parent or legal guardian will be instructed to evaluate local and systemic reactions at home for 7 days post-immunization (from Day 0 to Day 6 post vaccination day). Diary card will be issued to record adverse events and participant parent or legal guardian will be instructed regarding filling of the diary card. A thermometer will be given along with diary card to record

fever. Next clinic visit after 7 days will be scheduled. Participant parent or legal guardian will also be instructed to contact investigator/ study staff if needed.

Visit 12 - Safety follow-up visit for single dose group who received booster dose (Week 97, Day 679 ± 1 day)

1) Record solicited and unsolicited adverse events since last visit in CRF
2) Verify that the participant parent or legal guardian fills in the diary card correctly
3) Perform clinical examination (including vital signs and general physical exam) and record in CRF
4) Schedule next visit V13 and remind participant parent or legal guardian to contact investigator/ study staff if needed.

Visit 13 - Study completion visit (Week 100, Day 700 ± 7 days)

This is the final visit of the study.

1) Collect Diary Card and check with participant parent or legal guardian before recording observations in the CRF
2) Record any unsolicited AE, SAE and concomitant medications in CRF
3) Perform clinical examination including vital signs, general physical examination
4) Perform blood draw for assessment of immune responses
5) Ensure CRF is duely completed before the participant parent or legal guardian leave the study center

Participant will be continued to be monitored after study ends for unresolved AE.

7.7 PARTICIPANT WITHDRAWAL OR TERMINATION

7.7.1 REASONS FOR WITHDRAWAL OR TERMINATION

Participant parent or legal guardians are free to withdraw their child from participation in the study at any time upon request, without justification and without prejudice. The Principal Investigator may also decide to discontinue participation of a child from study interventions in the following cases:

1) An acute reaction (allergy, hypersensitivity reaction, etc.) to the investigational product.
2) Occurrence of an illness or serious adverse event or adverse event that in the judgment of the investigator may be detrimental for the participant's safety.

3) A study participant’s parent or legal guardian withdrawal of informed consent.
4) A study participant’s medical condition or use of medication that in the judgment of the investigator may compromise the participant’s safety and/or the scientific integrity of the study
5) Violation of the inclusion/exclusion criteria by the participant.
6) A study participant’s no-show for a scheduled visit without notices, unable to contact, and lost to follow-up.
7) Any other reason of study discontinuation as per the judgment of the Principal Investigator

7.7.2 HANDLING OF PARTICIPANT DISCONTINUATION OR TERMINATION

Discontinuation from study intervention does not mean discontinuation from the study, and remaining study procedures will be completed as indicated by the study protocol. If a clinically significant finding is identified after enrollment, the PI will determine if any change in participant management is needed. Any new clinically relevant finding will be reported as an adverse event.

Study team will encourage parent or legal guardian of withdrawn or terminated participant to continue in the study for safety follow-up. If she/he declines, this will end the participant’s interaction with the study team for this protocol. The study team will engage in no further communication with the volunteer except as directed by an IRB with regard to participant safety information. Protocol-specified safety follow-up procedures will be discussed with the participant parent or legal guardian to capture AEs, serious adverse events (SAEs), and unanticipated problems (UPs). The reason for participant discontinuation or withdrawal from the study will be recorded on the study follow-up Case Report Form (CRF). Only data and samples already collected will be analyzed according to protocol. The study team will not utilize samples or data from this volunteer for any future use and will discard residual samples when the study is completed. Counseling about any issue will be provided if he/she decides to discontinue participation in the study. Medical advice will also be provided in the best interest of the participant.

Participants whose parents or legal guardian have signed the informed consent form and who are randomized but do not receive the study intervention will be replaced. Participants who receive the study intervention and subsequently withdraw, or are withdrawn or discontinued from the study will not be replaced.

In the event of early termination of a participant:

1) Reason for early termination of the participant will be recorded in the CRF.
2) Any unsolicited AE, SAE and concomitant medications will be recorded in CRF
3) Clinical examination including vital signs, general physical examination will be performed if participant's parent/legal guardian is willing.

7.8 LOST TO FOLLOW-UP

A participant will be considered lost to follow-up if he or she fails to return for any of scheduled visits and remains unreachable to study site staff.

The following actions will be taken if a participant fails to return to the clinic for a required study visit:

- The site staff will attempt to contact the participant parent/legal guardian and counsel on the importance of maintaining the assigned visit schedule and ascertain if the participant parent/legal guardian wishes to and/or should continue in the study.
- Before a participant is deemed lost to follow-up, the investigator or designee will make every effort to regain contact with the participant parent/legal guardian (will make 3 telephone calls). These contact attempts should be documented in the participant's medical record or study file.

Should the participant continue to be unreachable, he/she will be considered to have withdrawn from the study with a primary reason of lost to follow-up.

7.9 PROTOCOL DEVIATIONS

A protocol deviation is any noncompliance with the clinical trial protocol or GCP by the participant, the investigator, or the study site staff. It is the responsibility of the PI to use continuous vigilance to identify and report all protocol deviations within 10 working days of identification of the protocol deviation to RITM IRB and to sponsor. The PI is responsible for knowing and adhering to the RITM requirements. The IVI Study Medical Monitor will report all protocol deviations to IVI IRB.

Major deviations are defined as those jeopardise the safety or rights of the participant or the scientific integrity of the study which is applicable to cases listed below.

- Violation of inclusion and exclusion criteria
- Vaccination with wrong vaccine as defined in the protocol
- Vaccination not following the immunization schedule defined in protocol
- Visit outside window for the immunogenicity assessment after discussion with the study medical monitor.
- Missed samples for immunogenicity

Major protocol deviations thought to affect the scientific integrity of the study will be reported and discussed with investigator, monitor, sponsor, and statistician for their exclusion from the per protocol analysis. For minor protocol deviations considered not to affect the scientific integrity of the study, the extent of deviation or delay as well as reason will be accurately documented.

7.10 PROTOCOL AMENDMENTS

Any amendment of the approved protocol shall be submitted to all IRBs (IVI and RITM) and to the National Regulatory Authority (PFDA) for approval before use.

7.11 PREMATURE TERMINATION OR SUSPENSION OF STUDY

This study may be temporarily suspended or prematurely terminated if there is sufficient reasonable cause as per PI recommendation after consultation with sponsor. IRBs and PFDA may also require termination of the study. Written notification, documenting the reason for study suspension or termination, will be provided by the suspending or terminating party to investigator, the sponsor, the regulatory authorities, and IRBs.

Circumstances that may warrant termination or suspension are:

- Determination of unexpected, significant, or unacceptable risk to participant as recommended by the PI
- Poor protocol compliance

Study may resume once concerns about safety, protocol compliance, data quality are addressed and satisfy the sponsor, IRBs and/or PFDA.

7.12 END OF STUDY

A participant is considered to have completed the study if he or she has completed all phases of the study including the last visit or the last scheduled procedure shown in the Schedule of Events (SOE) accodding to group allocation.

8 LABORATORY PROCEDURES/EVALUATIONS

8.1 CLINICAL LABORATORY EVALUATIONS

Within 7 days prior to enrollment, routine biological parameters will be measured at the RITM Hospital Laboratory to screen study participants. All participants with abnormal values as per lab value ranges specified below will be considered as screen failures and will not be enrolled in the study:

- White Blood Cell count <2.5 X10^{9} cells/L
- Platelets < 124.999 X10^{9} cells/L
- Hemoglobin ≤ 95 g/L
- Creatinine ≥ 1.1X upper limit of normal value
- Aspartate amino-transferase (AST), Alanine amino-transferase (ALT) ≥ 1.25 x upper limit of normal value

Harriet Lane Handbook, Nelson's Textbook of Paediatrics, 20th edition and RITM laboratory normal ranges also served as additional guidance. (Refer to MFR No. 8 dated 07SEP2018)

8.2 SPECIMEN PROCESSING, HANDLING, AND STORAGE

Venous blood will be collected from participants for screening biological parameters and immunogenicity assessments. For immunogenicity assessment, whole blood will be centrifuged, and sera will be aliquoted and stored at between -60°C to -80°C until shipment to IVI and use. Pre-print study labels provided in advance will be attached on each of the serum aliquots.

8.3 SPECIMEN SHIPMENT

Aliquoted blood samples for immunogenicity assessment will be shipped from RITM to the International Vaccine Institute, Seoul, Republic of Korea, where they will be stored at -70°C for analysis and storage for 5 years after study completion.

8.4 ASSESSMENT OF IMMUNOGENICITY

Anti-Vi IgG ELISA

The assay is used to measure anti-Vi specific antibodies of the IgG isotype in human sera. Poly-L-lysine is pre-coated and purified Vi antigen at a concentration of 2 µg/mL is absorbed onto 96-well microtiter plates. Non-specific binding sites are blocked with BSA in PBS, diluted human sera is then added to the first wells in the plate then serially diluted across the plate. Antibodies specific to Vi will bind to the Vi coated to the plate. The bound IgG is detected using alkaline phosphatase labelled goat anti-human IgG. Addition of 4-nitrophenyl phosphate substrate causes a colour change proportional to the amount of human anti-Vi IgG antibody present in the serum. Optical Densities of the wells are measured at 405 nm. The level of the specific anti-Vi IgG in ELISA units for each serum sample is determined by comparison to a reference serum.

Serum Bactericidal Antibody (SBA)

The serum bactericidal assay measures functional antibody level in test serum, which is capable of killing a *S*. Typhi Ty2 in the presence of exogenous complements. Complements in the sera are inactivated by heating them at 56°C for 30 min before the assay. *S*. Typhi Ty2 strain is cultured in a Luria-Bertani (LB) broth for 15 to 16 hours at 37°C, with shaking incubator. The bacteria are harvested by centrifugation and resuspended the cells in sterile normal PBS. Prepare a mixture containing the cultured bacteria and baby rabbit complements. Add the bacterial mixture to a 96-well microtiter plate containing properly diluted test serum samples in PBS. After incubation for 60 min at 37°C, each well is plated onto square LB agar plate and incubated overnight at 37°C. Number of survived colony on LB plate is counted. Serum bactericidal titer is defined as the highest dilution of serum that gives 50% of inhibition of colony formation of *S*. Typhi.

Measles, Mumps and Rubella Antibody Assays

Measles, mumps and rubella antibody titers will be measured by ELISA using a commercial kit.

9 ASSESSMENT OF SAFETY

9.1 SAFETY ASSESSMENT

The following procedures will be performed to monitor safety as listed in the SOE:

- ***Demographic and medical history*** *(age, gender, baseline medical history of participants)*
- ***Physical examination*** *(height and weight, organ systems, growth and development and motor assessments)*
- ***Vital signs*** *(temperature, pulse, respirations)*
- **Diary card** will be used for participant's parents/legal guardian reported outcomes.

At each study visit, the investigator will inquire about the occurrence of AE/SAEs since the last visit.

9.1.1 DEFINITION OF ADVERSE EVENTS (AE)

Adverse events (AE) are defined as any untoward medical occurrence which follows immunization and which does not necessarily have a causal relationship with the administration of the vaccine. An AE may be any unfavorable or unintended sign, symptom, abnormal laboratory finding or disease.

Solicited AEs are predetermined events, identified in the Investigator's Brochure, which may reflect safety concerns related to the investigational product. AEs that will be solicited by the participant's parent or legal guardian and recorded in the diary card and reviewed by a blinded observer during the 7 days after each dose for this study include:

- Local reactions at the site of injection: Pain/tenderness, erythema/redness, induration/swelling, pruritus
- Systemic reactions: Fever, lethargy, irritability, vomiting, diarrhea, drowsiness, loss of appetite, and persistent crying. Rash and nasopharyngitis are described as possible MMR vaccine-specific adverse events and will be collected following the vaccine administration at week 0 for children aged 9 to less than 12 months.

Unsolicited AEs are all other adverse events (those that do not fall under the categories of solicited Averse Reactions) that are identified by site staff, the PI and the Medical Research Monitors. These unsolicited AEs will be documented in the participant's clinic records and

entered in the study eCRFs. They will be recorded during the 4 weeks after each dose and between the two doses at weeks 0 and 24. At weeks 12, 24, 36, 60, 96 and 100, AEs that are “medically significant” events, defined as requiring multiple visits (two or more) to a physician for the same condition, or that result in hospitalization or an emergency room visit, will be captured.

Results will be expressed as frequency of the AEs and individual descriptions will be tabulated according to MedDRA organ class system.

9.1.2 DEFINITION OF SERIOUS ADVERSE EVENTS (SAE)

An AE or suspected adverse reaction is considered "serious" if, in the view of either the investigator or sponsor, it results in any of the following outcomes:

- Death,
- Life-threatening event
- In-patient hospitalization > 24 hours or prolongation of existing hospitalization
- Persistent or significant incapacity or substantial disruption of the ability to conduct normal life functions
- Congenital anomaly/birth defect.
- Important medical events that may not result in death, be life-threatening, or require hospitalization may be considered serious when, based upon appropriate medical judgment, they may jeopardize the participant and may require medical or surgical intervention to prevent one of the outcomes listed in this definition.

9.1.3 DEFINITION OF UNANTICIPATED PROBLEMS (UP)

Unanticipated problems include, any incident, experience, or outcome that meets **all** of the following criteria:

- Unexpected in terms of nature, severity, or frequency of what is stated under adverse events in the protocol informed consent and Investigator’s Brochure (IB).
- Related or possibly related to vaccination
- Suggests that participants or others will be at a greater risk of harm (including physical, psychological, economic, or social harm) than was previously known or recognized.

Suspected unexpected serious adverse event (SUSAR) is defined as a serious adverse reaction whose nature or severity is not consistent with the applicable product information, VI-DT Investigator’s Brochure or the summary of product characterisitcs of an authorized product.

9.2 CLASSIFICATION OF AN ADVERSE EVENT

9.2.1 SEVERITY OF EVENT

All solicited AEs in the study will be graded for their severity and recorded in the eCRF as described in below table. All other AEs will be assessed by the study clinician using the National Institute of Allergy and Infectious Diseases (NIAID) Division of AIDS Table for grading the severity of Adult and Pediatric Adverse events [34] .

Table 5. Solicited systemic adverse reaction severity grading

Systemic (General)	Mild (Grade1)	Moderate (Grade2)	Severe (Grade3)	Potentially Life Threatening (Grade 4)
Fever*	38.0 – 38.5°C	38.6 – 39.2°C	39.3 – 39.9°C	$\geq$ 40°C
Irritability	Require minimal or no treatment	Results in low level of inconvenience or concern	Interupt daily activity and require drug therapy	ER visit or hospitalization
Vomiting	No interference with routine activity	Some interference with routine activity	Significant; prevents routine daily activity	ER visit or hospitalization
Diarrhea	No Interference with routine activity 1-2 episodes/24	Some Interference with routine activity > 2 episodes/24	Prevents daily activity requires outpatient IV hydration	ER visit or hospitalization for hypotensive shock
Drowsiness	No interference with routine activity	Some interference with routine activity	Significant; prevents daily routine activity	ER visit or hospitalization
Loss of appetite	Require minimal or no treatment	Results in low level of inconvenience or concern	Require drug therapy	ER visit or hospitalization
Persistent crying	Require minimal or no treatment	Results in low level of inconvenience or concern	Interupt daily activity and require drug therapy	ER visit or hospitalization

Rash	Localized Rash	Diffuse rash OR Target lesions	Diffuse rash and vesicles or limited number of bullae or superficial ulceration of mucus membranes limited to one side	Extensive or generalized bulleous lesion OR ulceration of mucus memberane involving two or more distinct mucosal sites OR Stevens Johnson syndrome
Nasopharyngits	Require minimal or no treatment	Results in low level of inconvenience or concern	Interupt daily activity and require drug therapy	ER visit or hospitalization

* Axillary temperature will be recorded.

Table 6. Solicited local adverse reaction severity grading

Local Reaction to Injectable Product	Mild (Grade 1)	Moderate (Grade 2)	Severe (Grade 3)	Potentially Life Threatening (Grade 4)
Pain /Tenderness	Does not interfere with routine activity	Interferes with routine activity or repeated use of non-narcotic pain reliever	Prevents routine daily activity or repeated use of narcotic pain reliever	Emergency room (ER) visit or hospitalization
Erythema/Redness	Affected area ≤ 2.5 cm in diameter	Affected area > 2.5 cm in diameter, but less than 50% of extrimity segment is affected	> 50% of extremity segment is affected or ulceration or phlebitis or secondary infection or sterile abcess	Exfoliative Dermatitis, Necrosis involving dermis and deeper tissue

Swelling/Induration	Affected area ≤ 2.5 cm in diameter	Affected area > 2.5 cm in diameter, but < 50% of extremity segment is affected	More than 50% of extremity segment is affected or ulceration or phlebitis or secondary infection or sterile abcess	Exfoliative Dermatitis, Necrosis involving dermis and deeper tissue
Pruritis associated with injection	Itching localized to injection site AND Relieved spontaneously or with < 48 hours treatment	Itching beyond the injection site but not generalized OR Itching localized to injection site requiring ≥ 48 hours treatment	Generalized itching causing inability to perform usual social & functional activities	Not Applicable

Signs and symptoms of unsolicited AEs will be recorded from the date of first vaccination until week 28 post first vaccination for all study groups, and until week 100 for Groups A and until week 96 for Group B.

All unsolicited adverse events observed by investigator and/or reported by parent/legally acceptable representative after discussing with the investigator will be recorded in the CRFs with their severity grading and relatedness to the study vaccine.

All SAEs irrespective of their causal association will also be graded for their severity.

9.2.2 RELATIONSHIP TO INVESTIGATIONAL PRODUCT

For all collected AEs, the clinician who examines and evaluates the participant will determine the relationship of each AE with the investigational product based on plausible biologic mechanism, temporal relationship of occurrence after administration of the investigational product, identification of possible alternative ethiologies including underlying disease, concurrent illness or concommittent medication, and his/her clinical judgment. The relationship of vaccination to advere event (AE) will be determined based on the definitions below.

- **Definitely Related –** There is clear evidence to suggest a causal relationship, and other possible contributing factors can be ruled out. The clinical event, including an abnormal laboratory test result, occurs in a plausible time relationship to vaccine administration and cannot be explained by concurrent disease or other drugs or chemicals. The response to withdrawal of the Vaccine (dechallenge) should be clinically plausible.
- **Probably Related –** There is evidence to suggest a causal relationship, and the influence of other factors is unlikely. The clinical event, including an abnormal laboratory test result, occurs within a reasonable time after administration of vaccine, is unlikely to be attributed to concurrent disease or other drugs or chemicals, and follows a clinically reasonable response on withdrawal (dechallenge).
- **Possibly Related –** There is some evidence to suggest a causal relationship (e.g., the event occurred within a reasonable time after administration of vaccine). However, other factors may have contributed to the event (e.g., the participant clinical condition, other concomitant events). Although an AE may rate only as “possibly related” soon after discovery, it can be flagged as requiring more information and later be upgraded to “probably related” or“definitely related,” as appropriate.
- **Unlikely to be related** – A clinical event, including an abnormal laboratory test result, whose temporal relationship to vaccine administration makes a causal relationship improbable (e.g., theevent did not occur within a reasonable time after administration of vaccine) and inwhich other drugs or chemicals or underlying disease provides plausible explanations (e.g., theparticipant clinical condition, other concomitant treatments).
- **Not Related** – The AE is completely independent of vaccine administration, and/or evidence exists that the event is definitely related to another etiology. There must be an alternative,definitive etiology documented by the clinician.

9.2.3 EXPECTEDNESS

The Study Medical Monitor will be responsible for determining whether an AE is expected or unexpected. An Adverse Reaction will be considered unexpected if the nature, severity, or frequency of the event is not consistent with the risk information previously described for the study agent.

9.3 TIME PERIOD AND FREQUENCY FOR EVENT ASSESSMENT AND FOLLOW-UP

All participants will be observed for immediate local and systemic reactions for 60 minutes after each vaccination. For 7 consecutive days (Days 0-6) after each dose of study vaccine, the participant's parent or legal guardian will be asked to record solicited local and systemic symptoms in the diary card. The study staff will remind participant parent or legal guardian's of the importance of properly filling the diary card and to return the card at the next scheduled study visit. If they did not fill up or lost their card, the parent or legal guardian will be interviewed for recall of symptoms with trained study staff during clinic visit on Day 7 after each vaccination.

The occurrence of an adverse event (AE) will come to the attention of study personnel during study visits and interviews of a study participant presenting for medical care, or upon review by a study monitor.

All AEs including local and systemic reactions not meeting the criteria for SAEs will be captured on the appropriate case report form (CRF). Information to be collected includes event description, time of onset, symptoms and physical examination findings, clinician's assessment of severity, relationship to study product (assessed by the PI), medications given and time of resolution/stabilization of the event. All AEs occurring while on study will be documented appropriately regardless of relationship.

Any medical condition that is present at screening will be considered as baseline and not reported as an AE. However, if the study participant's condition deteriorates or if frequency of intermittent condition increases at any time during the study, it will be recorded as an AE.

Changes in the severity of an AE will be documented to allow an assessment of the duration of the event at each level of severity to be performed. AEs characterized as intermittent require documentation of onset and duration of each episode.

The investigator will record all reportable events with start dates occurring any time after informed consent is obtained until the last day of study participation. At each study visit, the investigator will inquire about the occurrence of AE/SAEs since the last visit. Events will be followed for outcome information until resolution or stabilization.

9.4 REPORTING PROCEDURES

9.4.1 ADVERSE EVENT RECORDING AND REPORTING

Adverse events, solicited AEs, and SAEs will be assesed at all study visits, documented in the source record, and recorded in the eCRF using accepted medical terms and/or the diagnosis that accurately characterize the event. When the diagnosis is known the AE term recorded in the eCRF will be the diagnosis rather than constellation of symptoms. The PI will assess all AEs for seriousness, relationship to investigational product, severity, and other possible causes.

The timeframe for the collection of AEs and SAEs begins at the first administration of investigational product through to the end of the trial. All adverse events occuring through Visit 8 for all study groups and Visits 9 to 13 for Groups A and Visits 9 to 11 for Group B will be collected and recorded in the source document and eCRF.

When an AE has not resolved by the next visit it will be documented in the eCRF as ongoing. Documentation will include date of onset, detailed description of the event and relevant history and physical examination, severity, attribution of the AE, treatment given and date the AE improved or resolved. The medical monitor will review the AEs reported regularly and clearify with PI if there are queries. The data manager will review all AEs for consistency and provide summary of AEs to the medical monitor periodically. Non-clinically significant AEs still ongoing as the end of the study will be listed as continuing. SAEs continuing at the end of the study will be followed to resolution or stabilization. Details of AE reporting are included in the MOP.

The PI, sub-investigators, and site staff will exercise due deligence in ascertaining, accurately recording and promptely entering data on the eCRF for all AEs of all study participants. As data becomes availabe from the participant, the clinic and laboratories, adverse events should be recorded and entered by the site staff on regular basis. site investigators will review, in a timely manner, the AE source data and determine the severity of the event and relation to the study agent. Site investigators will contact the study medical monitor for consultation of AEs as required.

9.4.2 SERIOUS ADVERSE EVENT REPORTING

The PI will complete a SAE Form within the following time frame:

- All SAEs will be recorded on the SAE Form and submitted to the sponsor within 24 hours of initial receipt of the information (weekends and holidays are not included) and addressed to:

Email: *Birkneh.Tadesse@ivi.int*

Dr. Birkneh Tilahun Tadesse
Study Medical Monitor
International Vaccine Institute
SNU Research Park,
1 Gwanak-ro, Gwanak-gu, Seoul 08826 Republic of Korea,
Phone: +82-2-881-1231, +82-2-872-2801 (Ext:231)
Fax: +82-2-881-1164
Mobile :

- PFDA to be notified within 7 calendar days by the sponsor with complete report due within 8 additional calendar days of the first information.
- The RITM IRB will be notified within fifteen (15) days after knowledge of the occurrence of the event OR within 10 working days after the occurrence of the event OR 48 hours after the causality of the event was established by the principal investigator.

All information (which may include special investigations and treatment received) will be recorded on the SAE Form and submitted to PFDA and RITM IRB. All SAEs will be followed until satisfactory resolution or until the site investigator deems the event to be chronic or to be stable. Other supporting documentation of the event may be requested by the sponsor and should be provided as soon as possible. SAE reporting to IVI IRB is not mandatory and will be reported with the annual renewal report.

- The sponsor will be responsible for notifying the IVI IRB of Suspected Unexpected Serious Adverse Reactions (SUSAR) within 24 hours of initial receipt of the information (weekends and holidays are not included).

The SUSAR report will include the following information:

- Protocol information: protocol number and date
- A detailed description of the event, incident, experience, or outcome
- An explanation of the basis for determining that the event, incident, experience, or outcome represents an unexpected problem

9.4.3 SAFETY OVERSIGHT

An internal Safety Monitoring Committee (SMC) will be responsible to oversee the vaccine safety patterns during the course of the clinical trial. The SMC will be composed of individuals with appropriate expertise, including at least one medically qualified person (Refer to MFR No. 15 dated 17APR2019). The SMC will review blinded safety data on a regular basis according to the guidelines of the SMC charter. The SMC will send a summary of safety review findings to the PI and the Study Medical Monitor.

10 STUDY MONITORING

Study monitoring and auditing will be performed in accordance with the sponsor's procedures, GCP guidelines and any other applicable regulatory requirements.

Upon successful approval of the protocol and establishment of the Regulatory File, the clinical monitor will establish a clinical monitoring plan. To ensure that the investigator and the study staff understand and accept their defined responsibilities, the clinical monitor will maintain regular correspondence with the site and may be present during the course of the study to verify the acceptability of the facilities, compliance with the investigational plan and relevant regulations, and the maintenance of complete records.

Investigators and/or their study staff will be trained on the study protocol and all applicable study procedures prior to study initiation. Electronic CRFs supplied by the sponsor must be completed for each enrolled participant. The data entries as well as study related documents will be checked by the sponsor and/or trained delegates of the sponsor.

Study progress will be monitored by IVI study team or representative (e.g., a contract research organization) as frequently as necessary to ensure the rights and well-being of study participants are protected; to verify adequate, accurate and complete data collection; protocol compliance and to determine that the study is being conducted in conformance with applicable regulatory requirements. Arrangements for monitoring visits will be made in advance in accordance with the monitoring plan, except in case of emergency.

11 STATISTICAL CONSIDERATIONS

11.1 SAMPLE SIZE

Assuming a 10% dropout rate, the sample size of n=114 for Vi-DT groups vs. n=57 placebo/comparator vaccine would provide >99% power to detect the superiority of seroconversion rate in two Vi-DT regimens compared to comparator group where the seroconversion rate of two Vi-DT regimens and comparator group are assumed as 95% for each Vi-DT regimen, and 15%, respectively using one-sided test at 0.0125 significance level. This sample size will also provide 90% power for a non-inferiority test of GMT ratio of anti-Vi IgG between single-dose and two-dose regimens, using one-sided test at a 0.025 significance level (85% for significant level of 0.0125), the true GMT ratio is assumed as 1, the coefficient of variation on titer of immunogenicity is assumed as 3.0, and the non-inferiority margin of ratio was assumed as 0.5 [WHO TRS 924]. The seroconversion rate and CV of GMT were assumed conservatively based on phase 1 data.

A total of 285 participants aged 6 to 23 months will be enrolled in this study, 114, 114 and 57 participants will be randomized to either the single dose, two-dose Vi-DT regimens or placebo/comparator group, respectively within age strata. Three age strata will be 6 to less than 9 months, 9 to 12 months and 13 to 23 months. In each age strata, a minimum number of 80 participants will be enrolled. We will allow the 9-12 months old children to receive measles, mumps and rubella (MMR) vaccine concomitantly with Vi-DT vaccine and descriptive analysis of immune response to MMR only and to MMR and Vi-DT vaccines will be performed to assess the possible immunological interference with MMR vaccine.

11.2 STATISTICAL ANALYSIS PLAN

The statistical analysis will focus on comparisons of single dose and two doses immunogenicity at time points specified in the Schedule of Events (SOE) compared to comparator as well as between the two regimens. Additional statistical analysis details will be described in the statistical analysis plan (SAP) (which will be finalised prior to database lock) and any deviation(s) from the original SAP will be described and justified in the final study report.

11.3 STATISTICAL HYPOTHESES

The comparison for the primary objective is seroconversion rate in anti-Vi IgG ELISA antibody titer at 4 week after single dose Vi-DT (combined one and two-dose regimens) vs. Comparator vaccine at week 4. The primary hypothesis is:

- Seroconversion rate of single dose of Vi-DT vaccine is superior to comparator vaccine at week 4.

The primary hypothesis will be tested with type 1 error of 0.0125 using one-sided test at the interim analysis.

The two main comparisons for the secondary objectives are:

- Seroconversion rate in anti-Vi IgG ELISA antibody titer at 4 weeks after two dose Vi-DT (week 28) vs. Placebo/Comparator vaccine at 4 weeks after second vaccine administration (week 28), and
- Geometric mean titer (GMT) of anti-Vi IgG ELISA antibody at 4 weeks after single dose Vi-DT regimen (week 4) vs. two dose Vi-DT regimens (week 28).

The secondary hypotheses are:

- Seroconversion rate of two doses of Vi-DT vaccine is superior to comparator vaccine at week 28
- GMT of anti-Vi at 4 weeks post single dose (at week 4) of Vi-DT vaccine is non-inferior to GMT of anti-Vi 4 weeks post two doses (at week 28) of Vi-DT vaccine

The seroconversion rate in anti-Vi IgG ELISA antibody titer 4 weeks after two doses of Vi-DT (week 28) vs. Placebo/Comparator vaccine 4 weeks after second vaccine administration (week 28), (the first of the secondary comparisons) will be tested with rest of type 1 error rate of 0.0125.

Once, either the primary endpoint or the first secondary endpoint are statistically significant, then Geometric mean titer (GMT) of anti-Vi IgG ELISA antibody at 4 weeks after single dose regimen (week 4) vs. two dose Vi-DT regimens (week 28) (the 2nd secondary endpoint) will be tested sequentially using type 1 error of 0.025 or 0.0125 dependings on statistical significance of both or one hypothesis, respectively.

The following secondary comparisons will be tested without adjusting for multiple testing. Since the following secondary comparisons would not be powered in the study, these assessments

and descriptions will not be used as decision-making criteria but will contribute to a more detailed knowledge of the immune response induced by the Vi-DT vaccine.

- Seroconversion rate 4 weeks post single dose (week 4) regimen and 4 weeks post two dose (week 28) regimen
- Anti-Vi GMT and seroconversion rates 28 weeks post first vaccination between single dose regimen (week 28) and two-dose regimen (4 weeks post second dose)
- Anti-Vi GMT between weeks 4 and 28 for the two-dose regimen
- Anti-Vi GMT and seroconversion rates at weeks 24 and 28 of the two-dose regimen
- Anti-Vi GMT and seroconversion rates at week 60 of the single dose and two-dose regimens
- Anti-Vi GMT and seroconversion rates at weeks 96 and 100 in the single dose regimen
- Anti-Vi GMT and seroconversion rates at week 96 of the single dose and two-dose regimens

11.4 ANALYSIS DATASETS

The intention-to-treat (ITT) analysis set will include all participants randomized in the study. This data set will be used for demographic information.

The safety analysis set is a subset of ITT analysis set of those who received at least one dose of investigational vaccines.

The immunogenicity analysis set is a subset of ITT analysis set of those who received at least one dose of investigational vaccines and have at least one post-baseline immunogenicity data available.

The per-protocol (PP) analysis set will be a subset of the immunogenecity analysis set who do not have protocol violations (defined as major deviation from the protocol compromising the scientific integrity of the study) with regards to the inclusion/exclusion criteria, are compliant with study procedures, completed all visits as scheduled and received the correct vaccinations.

The immunogenicity analysis set will be used for the primary analysis of interest for the immunogenicity endpoints. A secondary analysis using the PP analysis sets will be conducted for the secondary immunogenicity endpoints.

11.5 DESCRIPTION OF STATISTICAL METHODS

11.5.1 GENERAL APPROACH

This study is a randomized, observer-blinded phase II study in healthy infants 6-23 months of age at the time of the first vaccine dose to assess and compare immunogenicity between single dose and two dose(s) of Vi-DT with Placebo/Comparator vaccine by superiority test, and single dose and two-dose regimen by non-inferiority test.

Unless specified, for superiority test significance level is 2.5% with one-sided test. For non-inferiority test for GMT ratio of Vi-DT regimens, significance level is 2.5% (or 1.25 %) with one-sided tail and non-inferiority margin of the GMT ratio is 0.5.

Analysis of covariance will be used to adjust for baseline titers, stratification and imbalances in baseline characteristics if necessary.

Missing immunogenicity data will not be imputed for the analysis.

11.5.2 BASELINE DESCRIPTIVE STATISTICS

Demographic characteristics and other baseline data of participants enrolled will be tabulated by vaccine group and overall. Continuous variables such as age, height and weight will be summarized by number of participants, mean, standard deviation, median, minimum and maximum. Categorical variables such as sex will be summarized by frequency and percentage in each vaccine.

11.5.3 SAFETY ANALYSIS

Number and proportion of participants with solicited AE 7 days post single dose and two doses of Vi-DT or placebo/comparator vaccine will be calculated and the 95% confidence interval of the proportion will be presented for each group within each age strata as well as overall strata.

Number and proportion of participants with unsolicited AEs single dose and two doses of Vi-DT or placebo/comparator vaccine through to 4 weeks following each dose will be provided within age strata as well as overall strata.

Occurance of any SAE during the study period will be also summarized and listed.

11.5.4 ANALYSIS OF THE PRIMARY IMMUNOGENICITY ENDPOINT(S)

The primary immunogenicity endpoint will be measured as seroconversion rate at week 4 (Placebo/Comparator vaccine and first dose of Vi-DT from single dose and two-dose groups combined) for comparison. For assessment of seroconversion rate, the proportion of participants with at least 4-fold rise anti-Vi IgG ELISA antibody titer at week 4 as compared to prior to the investigational product dosing (Day 0) will be calculated. The primary analysis of the primary endpoint will be done by Cochran-Mantel-Haenszel (CMH) test.

The two-sided 95% CI for each vaccine group will be provided with a estimate of seroconversion rate and superiority will be confirmed from the results and p-value of the CMH test. This endpoint will be tested at the interim analysis with type 1 error rate of 0.0125 (one-sided test from CMH test). An analysis of covariance model will be performed as a sensitivity analysis to adjust baseline characteristics.

11.5.5 ANALYSIS OF THE SECONDARY IMMUNOGENICITY ENDPOINT(S)

The first of the secondary immunogenicity endpoints will be measured as seroconversion rate at week 28 to compare two-dose regimen of Vi-DT with placebo/comparator. Superiority of anti-Vi seroconversion rate 4 weeks post two dose of Vi-DT compared to comparator vaccine at week 28 will be analyzed using the same approach as used for the primary immunogenicity endpoint.

The other secondary endpoint is GMT at week 4 in the single dose regimen and at week 28 in the two-dose regimen. The non-inferiority of anti-Vi GMT 4 weeks post single dose of Vi-DT vaccine (week 4) compared to 4 weeks post two dose (at week 28) of Vi-DT vaccine will be analyzed using an analysis of covariance model with group and strata as covariates after log transformation. The non-inferiority of one-dose regimen will be confirmed if the lower limit of one-tailed 97.5% (or one-tailed 98.75%) confidence interval of the ratio of GMT of single dose to two doses of Vi-DT is equal to or greater than the non-inferiority margin of 0.5.

The above two secondary comparisons would be tested sequentially in order to preserve the study wise type 1 error of 0.025 using one-sided test as decribed in Section 11.3.

The following secondary endpoints will be tested without adjusting for multiple testing. Since the following secondary endpoints would not be powered in the study, these assessments and

descriptions will not be used as decision-making criteria but will contribute to a more detailed knowledge of the immune response induced by the Vi-DT vaccine.

- Seroconversion rate 4 weeks post single dose regimen (week 4) and 4 weeks post two-dose regimen (week 28) of Vi-DT vaccine
- Anti-Vi GMT and seroconversion rates 28 weeks post first vaccination between single dose (week 28) and two-dose regimens (week 4 post second dose).
- Anti-Vi GMT between weeks 4 and 28 for the two-dose regimen
- Anti-Vi GMT and seroconversion rates at week 24 and week 28 of the two-dose regimen
- Anti-Vi GMT and seroconversion rates at week 60 in the single dose and two-dose regimens
- Anti-Vi GMT and seroconversion rates at weeks 96 and 100 in the single dose group
- Anti-Vi GMT and seroconversion rates at weeks 96 between the single dose and two-dose regimens of Vi-DT vaccine

GMT will be analyzed using an analysis of covariance model with group and strata as covariates after log transformation. Seroconversion rate will be analyzed by CMH test. For each vaccine group, two sided 95% CI will be also provided for GMT and seroconversion rates. Details of analysis method for these endpoints will be described in the SAP.

11.5.6 ADHERENCE AND RETENTION ANALYSES

Summaries of Participants Disposition will be based on all participants who provide informed consent/assent in the study. A flow diagram of participant disposition (CONSORT flow diagram) will illustrate the progress of participants through the study duration from initial screening for eligibility to the completion of the final primary outcome assessment. Number and percentage by vaccine group will be given for participants in the ITT, safety analysis set, immunogenicity analysis set and PP analysis sets, and reasons for study discontinuation.

11.5.7 PLANNED INTERIM ANALYSIS

An interim analysis will be performed after all participants complete week 4 visit post first Vi-DT dose (single dose and two-dose regimens combined). The purpose of this interim analysis is to help early Phase 3 planning. The primary endpoint and safety information within 4 weeks after the dose one of investigational vaccine will be cleaned, locked, summarzied and reported to the

funding agency in unblinded manner. For the interim analysis, 0.0125 of type 1 error (with one-side test) will be used to control overall studywise type 1 error rate of 0.025. Rest of 0.0125 will be used for the superiority testing of primary endpoint at the primary analysis. This interim analysis will be performed by independent statistician who is not involved in the study directly. The study and study personnel will remain blinded to allocation of groups and interventions.

11.5.8 ADDITIONAL SUB-GROUP ANALYSIS

Potential difference in safety and immunogenicity by sex, age group may be investigated.
The immunogenicity of Vi-DT alone or concomitantly with MMR vaccine will be described.

11.5.9 MULTIPLE COMPARISON/MULTIPLICITY

Type 1 error of 0.0125 (one-sided test) will be used to test the primary endpoint at interim analysis. The rest of 0.0125 will be used for the one-sided superiority test of the 1st secondary endpoint at primary analysis. If both primary and 1st secondary endpoints are significant, the 2nd secondary endpoint will be tested sequentially in order to preserve the study wise type 1 error of 0.025 using one sided test as described in Section 11.3.

11.5.10 EXPLORATORY ANALYSES

- Serum bactericidal GMT and seroconversion rates 4 weeks post Vi-DT between single dose (week 4) and two-dose (weeks 28) regimens and at weeks 60 and 96 of the single dose and two-dose regimens and at week 100 of single dose regimen will be described at each assessment timepoint using the same approach for other secondary endpoints as decribed in Section 11.5.5.
- For participants who received MMR vaccine comcomitantly with Vi-DT or placebo at week 0, immunogenicity (GMT of measles, mumps and rubella antibodies and number of participants with titer above thereshhold) at week 4 will be summarized for Vi-DT (single dose and two dose regimens combined) vs. placebo/comparator to assess the possible immunological interference of Vi-DT on MMR vaccine.

12 SOURCE DOCUMENTS AND ACCESS TO SOURCE DOCUMENTS

Data recorded on the eCRF will be verified by checking the eCRF entries against source documents (i.e., all original records, laboratory reports, medical records, participants diaries, memory aids) in order to ensure data completeness and accuracy as required by study protocol. Source documents will be stored at the clinical site in a secured place under lock and key. The investigator and/or site staff must make eCRFs and source documents of participants enrolled in this study available for inspection by IVI data managment team or its representative at the time of each monitoring visit.

At a minimum, source documentation must be available to substantiate participant identification, eligibility and participation, proper informed consent procedures, dates of visits, adherence to protocol procedures, adequate reporting and follow-up of adverse events, administration of concomitant medication, study vaccine receipt/dispensing/return records, study vaccine administration information, and date of completion and reason. Specific items required as source documents will be reviewed with the investigator before the study.

The source documents must also be available for inspection, verification and copying, as required by regulations, by officials of the regulatory health authorities (e.g., PFDA, others) and/or IRBs and for possible audit by BMGF. The investigator and study site staff must comply with applicable privacy, data protection and medical confidentiality laws for use and disclosure of information related to the study and enrolled participants.

The participant must also allow access to her medical records. Each participant should be informed of this prior to the start of the study.

Each participant will have a complete source documentation of records including study log books, ICF, lab reports and test results for the entire study period. Appropriate source documents will be prepared by study staffs. These records must be available to the IVI and regulatory authories upon request for review.

13 DATA HANDLING AND RECORD KEEPING

13.1 DATA COLLECTION AND MANAGEMENT RESPONSIBILITIES

Electronic Case Report Forms (eCRF) will be used for recording data for each participant enrolled in the study. The Prinicipal investigator is responsible to ensure the accuracy, completeness, legibility and timeliness of the data captured in eCRF. Data captured in the eCRF derived from source documents will be consistent with the source documents. In case of discrepancies, data will be clarified and corrected. The IVI will provide guidance to investigator on making corrections to the eCRF.

Study staff will extract all data collected in source documents and workbooks for computerization into the electronic Case Report Form (CRF). Data will be double-entered into computer in a dedicated area located using data entry programs specially created for the study. These programs will utilize custom-made software. All programs will incorporate identification of the key punching errors, range and consistency checks with data entry.

This data managment system will provide error reports and summary reports for each activity. The data will be automatically backed up systematically onto IVI server. In addition, the software will provide for an audit trail of all sequential changes made. Data security for this data management system will be augmented by automatic computer virus scanning at start-up of data entry and data management session, and password protection for accessing data and data management software. Data entry and cleaning will be conducted at the sites. Final data cleaning, data freezing and data analysis will be performed at the IVI. Unblinding of study vaccines will be carried out after database lock.

13.2 STUDY RECORDS RETENTION

The Principal Investigator will retain all study records required by sponsor and by the applicable regulations in a secure and safe facility. The PI will consult IVI representative before disposal of any study records, and will notify the sponsor of any change in the location, disposition, or custody of the study files.These documents should be retained for not less than 15 years (ICH E6, 4.9.5). The IVI will inform the PI as to when these documents no longer need to be retained (ICH E6, 5.5.12).

13.3 PUBLICATION AND DATA SHARING POLICY

IVI assures that the key design elements of this protocol will be posted in a publicly accessible database such as Clinicaltrials.gov. All data collected during this study will be used to support this vaccine development plan until licensure and WHO prequalification. All individual data will stay strictly confidential. Analyzed data may be presented in scientific conferences, and published in peer-reviewed scientific journals. Anyone wishing to publish or present data obtained during and/or after completion of the study will conform to study site policies and then forward the publication for review and approval to IVI and SK bioscience.

14 QUALITY ASSURANCE AND QUALITY CONTROL

As required by ICH E6 (R2) GCP, Quality Assurance and Quality Control (QC), the sponsor will implement a system to manage quality throughout all stages of the trial process and is responsible for implementing and maintaining quality assurance and quality control systems with written SOPs to ensure that trials are conducted and data are generated, documented (recorded), and reported in compliance with the protocol, GCP, and the applicable regulatory requirement(s).

Following written SOPs, the monitors will verify that the clinical trial is conducted and data are generated, documented (recorded), and reported in compliance with the protocol, GCP, and the applicable regulatory requirements (e.g., Good Clinical Laboratory Practices (GCLP), Good Manufacturing Practices (GMP)).

In addition, Quality Management (QM) will provide objective oversight during study planning, conduct and closue to include conduct of audits to support both vendor selection and compliance and Investigator Site compliance.

The investigational site will provide direct access to all trial related sites, source data/documents, and reports for the purpose of monitoring and auditing by the sponsor, and inspection by local and regulatory authorities.

15 ETHICS/PROTECTION OF HUMAN SUBJECTS

15.1 REGULATORY AND ETHICAL COMPLIANCE

The investigators will ensure that this study is conducted in full conformity with the ICH E6 GCP Guidelines, Council for International Organizations of Medical Science (CIOMS), local country's ethical policy statement or the Declaration of Helsinki, whichever provides the most protection to human subjects.

15.2 PARTICIPANT AND DATA CONFIDENTIALITY

Participant confidentiality is strictly held in trust by the participating investigators, their staff, and the sponsor and their agents. This confidentiality is extended to cover testing of biological samples and genetic tests in addition to the clinical information relating to participants. Therefore, the study protocol, documentation, data, and all other information generated will be held in strict confidence. No information concerning the study or the data will be released to any unauthorized third party without prior written approval of the sponsor.

The study monitor, other authorized representatives of the sponsor, representatives of the IRB or pharmaceutical company supplying study product may inspect all documents and records required to be maintained by the investigator, including but not limited to, medical records (office, clinic, or hospital) and pharmacy records for the participants in this study. The clinical study site will permit access to such records.

The study participant's contact information will be securely stored at clinical site for internal use during the study. At the end of the study, all records will continue to be kept in a secure location for as long a period as dictated by local IRB and Institutional regulations.

Individual participants and their research data will be identified by a unique study identification number. The study data entry and study management systems used by clinical site and by IVI Data Management will be secured and password-protected.

No personal identifier will be used in any publication or communication used to support this research study. The participant's identification number will be used in the event it becomes necessary to identify data specific to a single participant.

15.3. RESEARCH USE OF STORED HUMAN SAMPLES OR SPECIMES

- Intended Use: Samples and data collected under this protocol may be used to study immune responses to the vaccines administered and for safety purpose if deemed necessary per medical judgement of the PI or special request from the sponsor or IRBs. No genetic testing will be performed.
- Storage: Samples and data will be stored at RITM and IVI using codes assigned by the investigators. Data will be kept in password-protected computers. Only investigators will have access to the stored samples and data.
- Disposition at the completion of the study: All stored samples will be sent to IVI for long term storage. Study participants who request destruction of samples will be notified of compliance with such request and all supporting details will be maintained for tracking.

15.4 FUTURE USE OF STORED SPECIMENS

With the participant's approval (consent form) and as approved by RITM and IVI IRBs, the identified biological samples will be stored at the immunology lab in IVI for future use. The immunology lab at IVI will be attributed a code that will allow linking the biological specimens with the phenotypic data from each participant, maintaining the masking of the identity of the participant.

During the conduct of the study, an individual participant can choose to withdraw consent to have biological specimens stored for future research. However, withdrawal of consent with regard to biosample storage will not be possible after the study is completed.

The stored samples may be used for additional assessment of immunogenicity, study of possible immune correlates of protection, validation of assays, testing of new assays, and for safety purpose.

16 REFERENCES

1. WHO (2008) Weekly Epidemiological Records:Typhoid. WHO position paper 8: 49-59.
2. John J, Van Aart CJ, Grassly NC (2016) The Burden of Typhoid and Paratyphoid in India: Systematic Review and Meta-analysis. PLoS Negl Trop Dis 10: e0004616.
3. Buckle GC, Walker CL, Black RE (2012) Typhoid fever and paratyphoid fever: Systematic review to estimate global morbidity and mortality for 2010. J Glob Health 2: 010401.
4. Crump JA, Luby SP, Mintz ED (2004) The global burden of typhoid fever. Bull World Health Organ 82: 346-353.
5. Mogasale V, Maskery B, Ochiai RL, Lee JS, Mogasale VV, et al. (2014) Burden of typhoid fever in low-income and middle-income countries: a systematic, literature-based update with risk-factor adjustment. Lancet Glob Health 2: e570-580.
6. Marks F, von Kalckreuth V, Aaby P, Adu-Sarkodie Y, El Tayeb MA, et al. (2017) Incidence of invasive salmonella disease in sub-Saharan Africa: a multicentre population-based surveillance study. Lancet Glob Health 5: e310-e323.
7. World Health Organization (2014) EWARN weekly summary.
8. Herriman R (2015) The Philippines reportable diseases update for 2015. Outbreak News Today.
9. Department of Health, Epidemiology Bureau, Public Health Surveillance Division, Republic of the Philippines (2016) Typhoid. Morbidity Week 48 (January 1- December 3, 2016).
10. Owais A, Sultana S, Zaman U, Rizvi A, Zaidi AK (2010) Incidence of typhoid bacteremia in infants and young children in southern coastal Pakistan. Pediatr Infect Dis J 29: 1035-1039.
11. Brooks WA, Hossain A, Goswami D, Nahar K, Alam K, et al (2005) Bacteremic Typhoid Fever in Children in an Urban Slum, Bangladesh. Emerg Infect Dis 11:326-329.
12. Cruz Espinoza LM, Nichols C, Adu-Sarkodie Y, Al-Emran HM, Baker S, et al. (2016) Variations of Invasive Salmonella Infections by Population Size in Asante Akim North Municipal, Ghana. Clin Infect Dis 62 Suppl 1: S17-22.
13. WHO Strategic Advisory Group of Experts (SAGE) on Immunization (2017) *http://www.who.int/imunization/sage/en/index.html* accessed: October 2017.
14. McClelland M, Sanderson KE, Spieth J, Clifton SW, Latreille P, et al. (2001) Complete genome sequence of Salmonella enterica serovar Typhimurium LT2. Nature 413: 852-856.
15. Crump JA, Luby SP, Mintz ED (2004) The global burden of typhoid fever. Bull World Health Organ 82: 346-353.
16. Bhan MK, Bahl R, Bhatnagar S (2005) Typhoid and paratyphoid fever. Lancet 366: 749-762.
17. Hornick RB, Greisman SE, Woodward TE, DuPont HL, Dawkins AT, et al. (1970) Typhoid fever: pathogenesis and immunologic control. 2. N Engl J Med 283: 739-746.

18. Hornick RB, Greisman SE, Woodward TE, DuPont HL, Dawkins AT, et al. (1970) Typhoid fever: pathogenesis and immunologic control. N Engl J Med 283: 686-691.

19. DeRoeck D, Jodar L, Clemens J (2007) Putting typhoid vaccination on the global health agenda. N Engl J Med 357: 1069-1071.

20. Levine MM, Ferreccio C, Black RE, Germanier R (1987) Large-scale field trial of Ty21a live oral typhoid vaccine in enteric-coated capsule formulation. Lancet 1: 1049-1052.

21. Klugman KP, Koornhof HJ, Robbins JB, Le Cam NN (1996) Immunogenicity, efficacy and serological correlate of protection of Salmonella typhi Vi capsular polysaccharide vaccine three years after immunization. Vaccine 14: 435-438.

22. Szu SC TD, Trofa AC, et al (1994) Laboratory and preliminary clinical characterization of Vi capsular polysaccharide-protein conjugate vaccines. Infection and Immunity 62:4440-4.

23. Thiem VD, Lin FY, Canh DG, Son NH, Anh DD, et al. (2011) The Vi conjugate typhoid vaccine is safe, elicits protective levels of IgG anti-Vi, and is compatible with routine infant vaccines. Clin Vaccine Immunol 18: 730-735.

24. Micoli F RS, Pisoni I, et al (2011) Vi-CRM 197 as a new conjugate vaccine against Salmonella Typhi. Vaccine 29:712-20.

25. van Damme P KF, Anemona A, et al (2011) Safety, immunogenicity and dose ranging of a new Vi-CRM(1)(9)(7) conjugate vaccine against typhoid fever: randomized clinical testing in healthy adults. PLos One 6:e25398.

26. Monjori Mitra AG, Suparna Chatterjee, Iqbal Kaur, Nisha Bhattacharya, Suparna Basu (2016) Efficacy and safety of vi-tetanus toxoid conjugated typhoid vaccine (PedaTyph™) in Indian children: School based cluster randomized study. Human Vaccin Immunother 12:939-45.

27. Mohan VK, Varanasi V, Singh A, Pasetti MF, Levine MM, et al. (2015) Safety and immunogenicity of a Vi polysaccharide-tetanus toxoid conjugate vaccine (Typbar-TCV) in healthy infants, children, and adults in typhoid endemic areas: a multicenter, 2-cohort, open-label, double-blind, randomized controlled phase 3 study. Clin Infect Dis 61: 393-402.

28. Cadoz M (1998) Potential and limitations of polysaccharide vaccines in infancy. Vaccine 16:1391-5.

29. Kanungo S Dutta S SD (2008) Epidemiology of typhoid and paratyphoid fever in India. J Infect Dev Ctries 2:454-60

30. Amicizia D, Arata L. Zangrillo F, Panatto D, Gasparini R (2017) Overview of the impact of Typhoid and Paratyphoid fever. Utility of Ty21a vaccine (Vivotif®). J Prev Med Hyg 58:E1-E33.

31. Stein KE (1992) Thymus-independent and thymus-dependent responses to polysaccharide antigens. J Infect Dis 165 Suppl 1: S49-52.

32. Bhutta ZA, Capeding MR, Bavdekar A, Marchetti E, Ariff S, et al. (2014) Immunogenicity and safety of the Vi-CRM197 conjugate vaccine against typhoid fever in adults, children, and infants in south and southeast Asia: results from two randomised, observer-blind, age de-escalation, phase 2 trials. Lancet Infect Dis 14: 119-129.

33. Canh DG, Lin FY, Thiem VD, Trach DD, Trong ND, et al. (2004) Effect of dosage on immunogenicity of a Vi conjugate vaccine injected twice into 2- to 5-year-old Vietnamese children. Infect Immun 72: 6586-6588.

34. Division of AIDS/NIAID/NIH (November 2014) Table for Grading the severity of Adult and Pediatric Adverse Events. Version 2.0.

17 APPENDICES

1. SALINE 0.9% SOLUTION PACKAGE INSERT
2. FLUQUADRI™ PACKAGE INSERT
3. TRIMOVAX® PACKAGE INSERT
4. PRIORIX® PACKAGE INSERT
5. VARIVAX® PACKAGE INSERT
