# Supplementary material for: Safety and immunogenicity of Vi-DT conjugate vaccine among 6-23-month-old children: Phase II, randomized, dose-scheduling, observer-blind Study
Source: eClinicalMedicine. 2020 Sep 9;27:100540. doi: 10.1016/j.eclinm.2020.100540 (PMC7599314; doi:10.1016/j.eclinm.2020.100540)
Supplement: Supplementary file 2 [file mmc2.docx]

***Supplementary File***

T[able S1 Inclusion and Exclusion Criteria defined for the study participants 2](#_Toc44662382)

[Table S2 Adverse Event Assessment Methodology 2](#_Toc44662383)

[Table S3 Solicited local adverse reaction severity grading 3](#_Toc44662384)

[Table S4 Solicited systemic adverse reaction severity grading 3](#_Toc44662385)

[Table S5 Distribution of Serious Adverse Events 8](#_Toc44662386)

[Table S6 Distribution of immediate reactions 10](#_Toc44662387)

[Table S7 Distribution of solicited adverse events 14](#_Toc44662388)

[Table S8 Proportion of subjects with solicited AE within 7 days after each vaccination (Safety Analysis Set) 20](#_Toc44662389)

[Table S9 Summary of Solicited Adverse Events [All ages] by relatedness 21](#_Toc44662390)

[Table S10 Summary of Unsolicited Adverse Events [All ages] by severity, relatedness 21](#_Toc44662391)

[Table S11 Distribution of unsolicited AEs 4 weeks after each vaccination 22](#_Toc44662392)

[Table S 12 Proportion of subjects with unsolicited AE within 4 weeks after each vaccination (Safety Analysis Set) 33](#_Toc44662393)

[Table S13 Summary of proportion of subject with medically significant unsolicited AE (except 4 weeks after each vaccination) 34](#_Toc44662394)

[Table S14 Distribution of medically significant unsolicited AE (except 4 weeks after each vaccination) 34](#_Toc44662395)

[Table S15 Seroconversion of Anti-Vi IgG Response for all ages – immunogenicity set 37](#_Toc44662396)

[Table S16 Seroconversion of Anti-Vi IgG Response as per age strata – Immunogenicity set 37](#_Toc44662397)

[Table S17 Seroconversion of Anti-Vi IgG Response for all ages – per protocol set 38](#_Toc44662398)

[Table S18 Seroconversion of Anti-Vi IgG Response by age strata – per protocol set 38](#_Toc44662399)

[Table S19 GMT and GMF rise of Anti-Vi IgG Response by age strata- Immunogenicity set 39](#_Toc44662400)

[Table S20 GMT of Anti-Vi IgG Response, all ages – Per Protocol Set 40](#_Toc44662401)

[Table S21 GMT of Anti-Vi IgG Response by age strata – Per Protocol Set 42](#_Toc44662402)

[Table S22 Seroconversion among children for whom measles, mumps and rubella vaccines were co-administered. 44](#_Toc44662403)

[Figure S1 Distribution of Height (cm) among participants in the Vi-DT trial 4](https://ivionline-my.sharepoint.com/personal/eunlyeong_park_ivi_int/Documents/01.Project/01.IVI%20T002/06.Anlysis%20Result/0.Manuscript/Vi-DT%20Phase%20II%20Supplementary%20File_25JUN2020_Redlines_ELP.docx#_Toc44333402)

[Figure S2 Overall distribution of weight (kg) among the participants in the Vi-DT trial 4](https://ivionline-my.sharepoint.com/personal/eunlyeong_park_ivi_int/Documents/01.Project/01.IVI%20T002/06.Anlysis%20Result/0.Manuscript/Vi-DT%20Phase%20II%20Supplementary%20File_25JUN2020_Redlines_ELP.docx#_Toc44333403)

[Figure S3 Heart Rate distribution (overall) 5](https://ivionline-my.sharepoint.com/personal/eunlyeong_park_ivi_int/Documents/01.Project/01.IVI%20T002/06.Anlysis%20Result/0.Manuscript/Vi-DT%20Phase%20II%20Supplementary%20File_25JUN2020_Redlines_ELP.docx#_Toc44333404)

[Figure S4 Respiratory rate (Overall) 6](https://ivionline-my.sharepoint.com/personal/eunlyeong_park_ivi_int/Documents/01.Project/01.IVI%20T002/06.Anlysis%20Result/0.Manuscript/Vi-DT%20Phase%20II%20Supplementary%20File_25JUN2020_Redlines_ELP.docx#_Toc44333405)

[Figure S5 Body Temperature (Overall) 7](https://ivionline-my.sharepoint.com/personal/eunlyeong_park_ivi_int/Documents/01.Project/01.IVI%20T002/06.Anlysis%20Result/0.Manuscript/Vi-DT%20Phase%20II%20Supplementary%20File_25JUN2020_Redlines_ELP.docx#_Toc44333406)

[Figure S6 Seroconversion of Anti-Vi IgG ELISA Response – Per Protocol set 38](https://ivionline-my.sharepoint.com/personal/eunlyeong_park_ivi_int/Documents/01.Project/01.IVI%20T002/06.Anlysis%20Result/0.Manuscript/Vi-DT%20Phase%20II%20Supplementary%20File_25JUN2020_Redlines_ELP.docx#_Toc44333407)

[Figure S7 GMT of Anti-Vi IgG ELISA Response – Per Protocol Set 41](#_Toc44333408)

Table S1 Inclusion and Exclusion Criteria defined for the study participants

| **Inclusion criteria** |
| --- |
| - Healthy infants and children 6-23 months of age at enrollment - Birth weight ≥ 2500 g - ≥ 37 weeks of pregnancy or judged to be full-term by the midwife or birth attendant - Parents aged 18 years and above and legal guardians aged 21 years and above as per the legal authorization in the Philippines, who had voluntarily given informed consent - Parents/Legal Guardians willing to follow the study procedures of the study and available for the entire duration of the study |
| **Exclusion criteria** |
| - Subject with abnormal routine biological values at screening - Subject concomitantly enrolled or scheduled to be enrolled in another trial - Acute illness, infectious disease or fever (axillary temperature ≥37.5°C), within three days prior to enrolment and vaccination - Known history of immune function disorders including immunodeficiency diseases, or chronic use of systemic steroids (>20 mg/day prednisone equivalent for periods exceeding 10 days), cytotoxic or other immunosuppressive drugs - Child with a previously ascertained or suspected disease caused by *S*. Typhi - Child who have had household contact with/and or intimate exposure to an individual with laboratory-confirmed *S*. Typhi - Known history or allergy to vaccines or other medications - Know history of allergy to eggs, chicken protein, neomycin and formaldehyde - History of uncontrolled coagulopathy or blood disorders - Mother has known HIV infection or other immune function disorders - Child whose parents or legal guardian planning to move from the study area before the end of study period |

Table S2 Adverse Event Assessment Methodology

| **Assessment Methodology** | |
| --- | --- |
| **Solicited General Adverse Events (Days 0 ~ 6)** | |
| Pain/tenderness | General observation/interview |
| Erythema/redness |  |
| Induration/swelling |  |
| Pruritus, |  |
| Lethargy |  |
| Irritability |  |
| Vomiting |  |
| Diarrhea |  |
| Drowsiness |  |
| Loss of appetite |  |
| Persistent crying |  |
| Rash and nasopharyngitis (only for 9-12-month stratum) * |  |
| Fever | Thermometer/interview |
| **Unsolicited Adverse Events (Days 0 ~ 180)** | |
| Any other symptoms | General observation/interview |

*nasopharyngitis was assessed for those who received MMR only.

Table S3 Solicited local adverse reaction severity grading

| **Local Reaction to Injectable Product** | **Mild**  **(Grade 1)** | **Moderate**  **(Grade 2)** | **Severe**  **(Grade 3)** | **Potentially Life Threatening (Grade 4)** |
| --- | --- | --- | --- | --- |
| Pain /Tenderness | Does not interfere with routine activity | Interferes with routine activity or repeated use of non-narcotic pain reliever | Prevents routine daily activity or repeated use of narcotic pain reliever | Emergency room (ER) visit or hospitalization |
| Erythema/Redness | Affected area ≤ 2·5 cm in diameter | Affected area > 2·5 cm in diameter, but less than 50% of extremity segment is affected | > 50% of extremity segment is affected or ulceration or phlebitis or secondary infection or sterile abscess | Exfoliative Dermatitis, Necrosis involving dermis and deeper tissue |
| Swelling/Induration | Affected area ≤ 2·5 cm in diameter | Affected area > 2·5 cm in diameter, but < 50% of extremity segment is affected | More than 50% of extremity segment is affected or ulceration or phlebitis or secondary infection or sterile abscess | Exfoliative Dermatitis, Necrosis involving dermis and deeper tissue |
| Pruritus associated  with injection | Itching localized to injection site AND  Relieved spontaneously or with < 48 hours treatment | Itching beyond the injection site but not generalized OR Itching localized to injection site requiring ≥ 48 hours treatment | Generalized itching  causing inability to perform usual social & functional activities | Not Applicable |

Table S4 Solicited systemic adverse reaction severity grading

| **Systemic (General)** | **Mild (Grade1)** | **Moderate (Grade2)** | **Severe (Grade3)** | **Potentially Life Threatening (Grade 4)** |
| --- | --- | --- | --- | --- |
| Fever* | 38·0 – 38·5°C | 38·6 – 39·2°C | 39·3 – 39·9°C | > 40°C |
| Lethargy | Changes causing no or minimal interference with usual social and functional activities | Mild lethargy or somnolence causing greater than minimal interference with usual social and functional activities | Confusion, memory impairment, lethargy or somnolence causing inability to perform usual social and functional activities | Dilirium OR optunded OR Coma |
| Irritability | Require minimal or no treatment | Results in low level of inconvenience or concern | Interrupt daily activity and require drug therapy | ER visit or hospitalization |
| Vomiting | No interference with routine activity | Some interference with routine activity | Significant; prevents routine daily activity | ER visit or hospitalization |
| Diarrhea | No Interference with routine activity 1-2 episodes/24 | Some Interference with routine activity > 2 episodes/24 | Prevents daily activity requires outpatient IV hydration | ER visit or hospitalization for hypotensive shock |
| Drowsiness | No interference with routine activity | Some interference with routine activity | Significant; prevents daily routine activity | ER visit or hospitalization |
| Loss of appetite | Require minimal or no treatment | Results in low level of inconvenience or concern | Require drug therapy | ER visit or hospitalization |
| Persistent crying | Require minimal or no treatment | Results in low level of inconvenience or concern | Interrupt daily activity and require drug therapy | ER visit or hospitalization |
| Rash | Localized Rash | Diffuse rash OR Target lesions | Diffuse rash and vesicles or limited number of bullae or superficial ulceration of mucus membranes limited to one side | Extensive or generalized bullous lesion OR ulceration of mucus membrane involving two or more distinct mucosal sites OR Stevens Johnson syndrome |
| Nasopharyngitis | Require minimal or no treatment | Results in low level of inconvenience or concern | Interrupt daily activity and require drug therapy | ER visit or hospitalization |

Figure S1 Distribution of Height (cm) among participants in the Vi-DT trial


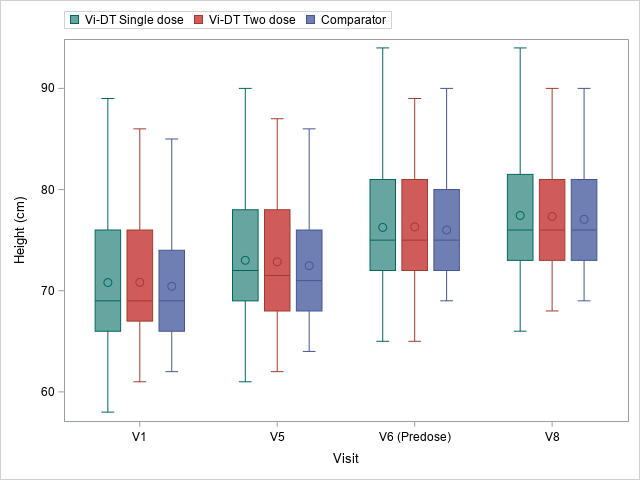


The middle box represents median (the middle horizontal line) and the open circles represent the mean height (cm) while the bar represents the interquartile range of the height (cm). The whiskers represent the ±1.5 * Inter Quartile Range (IQR) of the height (cm).


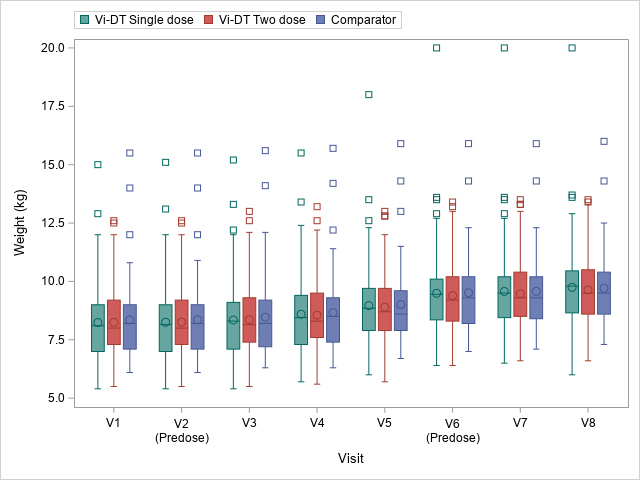


Figure S2 Overall distribution of weight (kg) among the participants in the Vi-DT trial

The middle box represents median (the middle horizontal line) and the open circles represent the mean weight (kg) while the bar represents the interquartile range (IQR) of the weight (kg). The whiskers represent ±1.5 * Inter Quartile Range (IQR) of the weight (km). The squares represent an outlier which is lower or greater than 1.5*IQR.


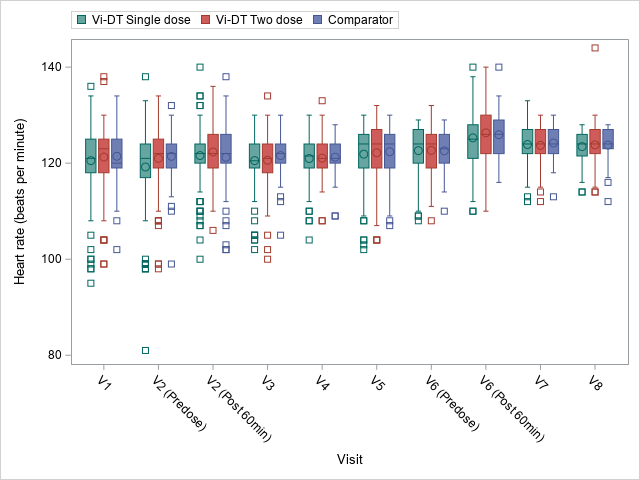


Figure S3 Heart Rate distribution (overall)

The middle box represents median (the middle horizontal line) and the open circles represent the mean heart rate (beats per minute) while the bar represents the interquartile range (IQR) of the heart rate (beats per minute). The whiskers represent ±1.5 * Inter Quartile Range (IQR) of the heart rate (beats per minute). The squares represent an outlier which is lower or greater than 1.5IQR.


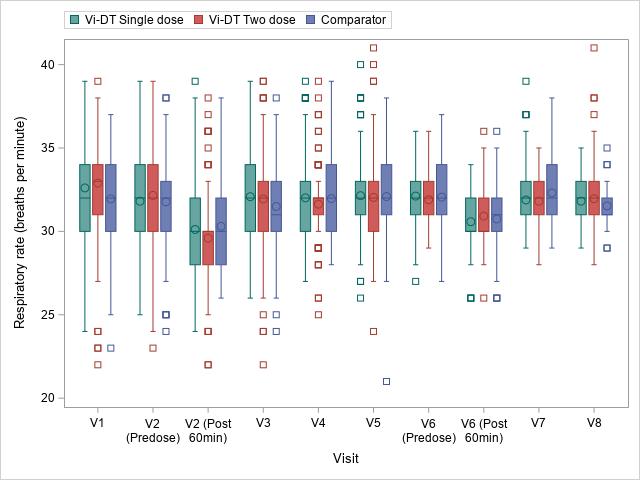


Figure S4 Respiratory rate (Overall)

The middle box represents median (the middle horizontal line) and the open circles represent the mean respiratory rate (breathes per minute) while the bar represents the interquartile range (IQR) of the respiratory rate (breaths per minute). The whiskers represent ±1.5 * IQR of the respiratory rate (breaths per minute). The squares represent an outlier which is lower or greater than 1.5IQR.

The middle box represents median (the middle horizontal line) and the open circles represent the mean body temperature (Celsius) while the bar represents the interquartile range of the body temperature (Celsius). The whiskers represent ±1.5 * Inter Quartile Range (IQR) of the body temperature (Celsius).


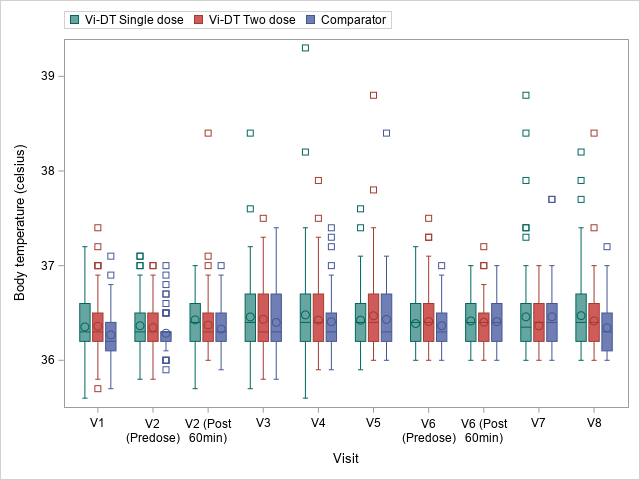


Figure S5 Body Temperature (Overall)

Table S5 Distribution of Serious Adverse Events

| **SOC\PT** | **Vi-DT Group**  **(N=228)** | |  |  |  |  | **Comparator Group (N=57)** | |
| --- | --- | --- | --- | --- | --- | --- | --- | --- |
|  |  |  | **Single dose**  **(N=114)** | | **two-dose**  **(N=114)** | |  |  |
| **Within 4 weeks after first dose** | **# of AEs** | **Number of participants (%)** | **# of AEs** | **Number of participants (%)** | **# of AEs** | **Number of participants (%)** | **# of AEs** | **Number of participants (%)** |
| **All ages** | **1** | **1 (0·44)** | **1** | **1 (0·88)** | **0** | **0 (0·00)** | **0** | **0 (0·00)** |
| **Nervous system disorders** | 1 | 1 (0·44) | 1 | 1 (0·88) | 0 | 0 (0·00) | 0 | 0 (0·00) |
| Febrile convulsion | 1 | 1 (0·44) | 1 | 1 (0·88) | 0 | 0 (0·00) | 0 | 0 (0·00) |
| **AgeStrata1** | **1** | **1 (1·32)** | **1** | **1 (2·63)** | **0** | **0 (0·00)** | **0** | **0 (0·00)** |
| **Nervous system disorders** | 1 | 1 (1·32) | 1 | 1 (2·63) | 0 | 0 (0·00) | 0 | 0 (0·00) |
| Febrile convulsion | 1 | 1 (1·32) | 1 | 1 (2·63) | 0 | 0 (0·00) | 0 | 0 (0·00) |
| **AgeStrata2** | **0** | **0 (0·00)** | **0** | **0 (0·00)** | **0** | **0 (0·00)** | **0** | **0 (0·00)** |
| **AgeStrata3** | **0** | **0 (0·00)** | **0** | **0 (0·00)** | **0** | **0 (0·00)** | **0** | **0 (0·00)** |
|  | **Vi-DT Group**  **(N=220)** | |  |  |  |  | **Comparator Group (N=55)** | |
|  |  |  | **Single dose**  **(N=112)** | | **two-dose**  **(N=108)** | |  |  |
| **Within 4 weeks after second dose** | **# of AEs** | **Number of participants (%)** | **# of AEs** | **Number of participants (%)** | **# of AEs** | **Number of participants (%)** | **# of AEs** | **Number of participants (%)** |
| **All ages** | **1** | **1 (0·45)** | **0** | **0 (0·00)** | **1** | **1 (0·93)** | **1** | **1 (1·82)** |
| **Gastrointestinal disorder** | 1 | 1 (0·45) | 0 | 0 (0·00) | 1 | 1 (0·93) | 0 | 0 (0·00) |
| Gastroenteritis | 1 | 1 (0·45) | 0 | 0 (0·00) | 1 | 1 (0·93) | 0 | 0 (0·00) |
| **Nervous system disorders** | 0 | 0 (0·00) | 0 | 0 (0·00) | 0 | 0 (0·00) | 1 | 1 (1·82) |
| Febrile convulsion | 0 | 0 (0·00) | 0 | 0 (0·00) | 0 | 0 (0·00) | 1 | 1 (1·82) |
| **AgeStrata1** | **0** | **0 (0·00)** | **0** | **0 (0·00)** | **0** | **0 (0·00)** | **0** | **0 (0·00)** |
| **AgeStrata2** | **1** | **1 (1·45)** | **0** | **0 (0·00)** | **1** | **1 (3·03)** | **1** | **1 (5·26)** |
| **Gastrointestinal disorder** | 1 | 1 (1·45) | 0 | 0 (0·00) | 1 | 1 (3·03) | 0 | 0 (0·00) |
| Gastroenteritis | 1 | 1 (1·45) | 0 | 0 (0·00) | 1 | 1 (3·03) | 0 | 0 (0·00) |
| **Nervous system disorders** | 0 | 0 (0·00) | 0 | 0 (0·00) | 0 | 0 (0·00) | 1 | 1 (5·26) |
| Febrile convulsion | 0 | 0 (0·00) | 0 | 0 (0·00) | 0 | 0 (0·00) | 1 | 1 (5·26) |
| **AgeStrata3** | **0** | **0 (0·00)** | **0** | **0 (0·00)** | **0** | **0 (0·00)** | **0** | **0 (0·00)** |
|  | **Vi-DT Group**  **(N=228)** | |  |  |  |  | **Comparator Group (N=57)** | |
|  |  |  | **Single dose**  **(N=114)** | | **two-dose**  **(N=114)** | |  |  |
| **Within 4 weeks after any dose** | **# of AEs** | **Number of participants (%)** | **# of AEs** | **Number of participants (%)** | **# of AEs** | **Number of participants (%)** | **# of AEs** | **Number of participants (%)** |
| **All ages** | **2** | **2 (0·88)** | **1** | **1 (0·88)** | **1** | **1 (0·88)** | **1** | **1 (1·75)** |
| **Gastrointestinal disorder** | 1 | 1 (0·44) | 0 | 0 (0·00) | 1 | 1 (0·88) | 0 | 0 (0·00) |
| Gastroenteritis | 1 | 1 (0·44) | 0 | 0 (0·00) | 1 | 1 (0·88) | 0 | 0 (0·00) |
| **Nervous system disorders** | 1 | 1 (0·44) | 1 | 1 (0·88) | 0 | 0 (0·00) | 1 | 1 (1·75) |
| Febrile convulsion* | 1 | 1 (0·44) | 1 | 1 (0·88) | 0 | 0 (0·00) | 1 | 1 (1·75) |
| **AgeStrata1** | **1** | **1 (1·32)** | **1** | **1 (2·63)** | **0** | **0 (0·00)** | **0** | **0 (0·00)** |
| **Nervous system disorders** | 1 | 1 (1·32) | 1 | 1 (2·63) | 0 | 0 (0·00) | 0 | 0 (0·00) |
| Febrile convulsion | 1 | 1 (1·32) | 1 | 1 (2·63) | 0 | 0 (0·00) | 0 | 0 (0·00) |
| **AgeStrata2** | **1** | **1 (1·32)** | **0** | **0 (0·00)** | **1** | **1 (2·63)** | **1** | **1 (5·26)** |
| **Gastrointestinal disorder** | 1 | 1 (1·32) | 0 | 0 (0·00) | 1 | 1 (2·63) | 0 | 0 (0·00) |
| Gastroenteritis | 1 | 1 (1·32) | 0 | 0 (0·00) | 1 | 1 (2·63) | 0 | 0 (0·00) |
| **Nervous system disorders** | 0 | 0 (0·00) | 0 | 0 (0·00) | 0 | 0 (0·00) | 1 | 1 (5·26) |
| Febrile convulsion | 0 | 0 (0·00) | 0 | 0 (0·00) | 0 | 0 (0·00) | 1 | 1 (5·26) |
| **AgeStrata3** | **0** | **0 (0·00)** | **0** | **0 (0·00)** | **0** | **0 (0·00)** | **0** | **0 (0·00)** |
|  | **Vi-DT Group**  **(N=228)** | |  |  |  |  | **Comparator Group (N=57)** | |
|  |  |  | **Single dose**  **(N=114)** | | **two-dose**  **(N=114)** | |  |  |
| **Entire study period** | **# of AEs** | **Number of participants (%)** | **# of AEs** | **Number of participants (%)** | **# of AEs** | **Number of participants (%)** | **# of AEs** | **Number of participants (%)** |
| **All ages** | **10** | **10 (4·39)** | **5** | **5 (4·39)** | **5** | **5 (4·39)** | **3** | **3 (5·26)** |
| **Gastrointestinal disorder** | 3 | 3 (1·32) | 1 | 1 (0·88) | 2 | 2 (1·75) | 1 | 1 (1·75) |
| Gastroenteritis | 3 | 3 (1·32) | 1 | 1 (0·88) | 2 | 2 (1·75) | 1 | 1 (1·75) |
| **Infections and infestation** | 4 | 4 (1·75) | 3 | 3 (2·63) | 1 | 1 (0·88) | 1 | 1 (1·75) |
| Infections NEC | 1 | 1 (0·44) | 0 | 0 (0·00) | 1 | 1 (0·88) | 0 | 0 (0·00) |
| Pneumonia | 3 | 3 (1·32) | 3 | 3 (2·63) | 0 | 0 (0·00) | 1 | 1 (1·75) |
| **Nervous system disorders** | 3 | 3 (1·32) | 1 | 1 (0·88) | 2 | 2 (1·75) | 1 | 1 (1·75) |
| Febrile convulsion | 3 | 3 (1·32) | 1 | 1 (0·88) | 2 | 2 (1·75) | 1 | 1 (1·75) |
| **AgeStrata1** | **5** | **5 (6·58)** | **2** | **2 (5·26)** | **3** | **3 (7·89)** | **0** | **0 (0·00)** |
| **Gastrointestinal disorder** | 1 | 1 (1·32) | 0 | 0 (0·00) | 1 | 1 (2·63) | 0 | 0 (0·00) |
| Gastroenteritis | 1 | 1 (1·32) | 0 | 0 (0·00) | 1 | 1 (2·63) | 0 | 0 (0·00) |
| **Infections and infestations** | 2 | 2 (2·63) | 1 | 1 (2·63) | 1 | 1 (2·63) | 0 | 0 (0·00) |
| Infections NEC | 1 | 1 (1·32) | 0 | 0 (0·00) | 1 | 1 (2·63) | 0 | 0 (0·00) |
| Pneumonia | 1 | 1 (1·32) | 1 | 1 (2·63) | 0 | 0 (0·00) | 0 | 0 (0·00) |
| **Nervous system disorders** | 2 | 2 (2·63) | 1 | 1 (2·63) | 1 | 1 (2·63) | 0 | 0 (0·00) |
| Febrile convulsion | 2 | 2 (2·63) | 1 | 1 (2·63) | 1 | 1 (2·63) | 0 | 0 (0·00) |
| **AgeStrata2** | **3** | **3 (3·95)** | **2** | **2 (5·26)** | **1** | **1 (2·63)** | **1** | **1 (5·26)** |
| **Gastrointestinal disorder** | 2 | 2 (2·63) | 1 | 1 (2·63) | 1 | 1 (2·63) | 0 | 0 (0·00) |
| Gastroenteritis | 2 | 2 (2·63) | 1 | 1 (2·63) | 1 | 1 (2·63) | 0 | 0 (0·00) |
| **Infections and infestations** | 1 | 1 (1·32) | 1 | 1 (2·63) | 0 | 0 (0·00) | 0 | 0 (0·00) |
| Pneumonia | 1 | 1 (1·32) | 1 | 1 (2·63) | 0 | 0 (0·00) | 0 | 0 (0·00) |
| **Nervous system disorders** | 0 | 0 (0·00) | 0 | 0 (0·00) | 0 | 0 (0·00) | 1 | 1 (5·26) |
| Febrile convulsion | 0 | 0 (0·00) | 0 | 0 (0·00) | 0 | 0 (0·00) | 1 | 1 (5·26) |
| **AgeStrata3** | **2** | **2 (2·63)** | **1** | **1 (2·63)** | **1** | **1 (2·63)** | **2** | **2 (10·53)** |
| **Gastrointestinal disorder** | 0 | 0 (0·00) | 0 | 0 (0·00) | 0 | 0 (0·00) | 1 | 1 (5·26) |
| Gastroenteritis | 0 | 0 (0·00) | 0 | 0 (0·00) | 0 | 0 (0·00) | 1 | 1 (5·26) |
| **Infections and infestation** | 1 | 1 (1·32) | 1 | 1 (2·63) | 0 | 0 (0·00) | 1 | 1 (5·26) |
| Pneumonia | 1 | 1 (1·32) | 1 | 1 (2·63) | 0 | 0 (0·00) | 1 | 1 (5·26) |
| **Nervous system disorders** | 1 | 1 (1·32) | 0 | 0 (0·00) | 1 | 1 (2·63) | 0 | 0 (0·00) |
| Febrile convulsion | 1 | 1 (1·32) | 0 | 0 (0·00) | 1 | 1 (2·63) | 0 | 0 (0·00) |

*Three cases of Convulsion occurred in the Vi-DT group – 15, 40 and 127 days after first dose and all were assessed as not related. One convulsion in the placebo group occurred 20 days following second dose

Table S6 Distribution of immediate reactions

|  | **Vi-DT Group**  **(N=228)** | |  |  |  |  | **Comparator Group (N=57)** | | |
| --- | --- | --- | --- | --- | --- | --- | --- | --- | --- |
|  |  |  | **Single dose**  **(N=114)** | | **two-dose**  **(N=114)** | |  |  |  |
| **After first dose** | **# of AEs** | **Number of participants (%)** | **# of AEs** | **Number of participants (%)** | **# of AEs** | **Number of participants (%)** | **# of AEs** | **Number of participants (%)** | |
| **All ages** | **3** | **1 (0.44)** | **0** | **0 (0.00)** | **3** | **1 (0.88)** | **0** | **0 (0.00)** | |
| **Local AE** | **1** | **1 (0.44)** | **0** | **0 (0.00)** | **1** | **1 (0.88)** | **0** | **0 (0.00)** | |
| Pain/Tenderness | 0 | 0 (0.00) | 0 | 0 (0.00) | 0 | 0 (0.00) | 0 | 0 (0.00) | |
| Erythema/Redness | 1 | 1 (0.44) | 0 | 0 (0.00) | 1 | 1 (0.88) | 0 | 0 (0.00) | |
| Swelling/Induration | 0 | 0 (0.00) | 0 | 0 (0.00) | 0 | 0 (0.00) | 0 | 0 (0.00) | |
| Pruritus | 0 | 0 (0.00) | 0 | 0 (0.00) | 0 | 0 (0.00) | 0 | | 0 (0.00) |
| **Systemic AE** | **1** | **1 (0.44)** | **0** | **0 (0.00)** | **1** | **1 (0.88)** | **0** | | **0 (0.00)** |
| Fever | 1 | 1 (0.44) | 0 | 0 (0.00) | 1 | 1 (0.88) | 0 | | 0 (0.00) |
| Lethargy | 0 | 0 (0.00) | 0 | 0 (0.00) | 0 | 0 (0.00) | 0 | | 0 (0.00) |
| Irritability | 0 | 0 (0.00) | 0 | 0 (0.00) | 0 | 0 (0.00) | 0 | | 0 (0.00) |
| Vomiting | 0 | 0 (0.00) | 0 | 0 (0.00) | 0 | 0 (0.00) | 0 | | 0 (0.00) |
| Diarrhea | 0 | 0 (0.00) | 0 | 0 (0.00) | 0 | 0 (0.00) | 0 | | 0 (0.00) |
| Drowsiness | 0 | 0 (0.00) | 0 | 0 (0.00) | 0 | 0 (0.00) | 0 | | 0 (0.00) |
| Loss of appetite | 0 | 0 (0.00) | 0 | 0 (0.00) | 0 | 0 (0.00) | 0 | | 0 (0.00) |
| Persistent crying | 0 | 0 (0.00) | 0 | 0 (0.00) | 0 | 0 (0.00) | 0 | | 0 (0.00) |
| Rash^*^ | 0 | 0 (0.00) | 0 | 0 (0.00) | 0 | 0 (0.00) | 0 | | 0 (0.00) |
| Nasopharyngitis^*^ | 0 | 0 (0.00) | 0 | 0 (0.00) | 0 | 0 (0.00) | 0 | | 0 (0.00) |
| **Other AE** | **1** | **1 (0.44)** | **0** | **0 (0.00)** | **1** | **1 (0.88)** | **0** | | **0 (0.00)** |
| T/C HYPERSENSITIVITY REACTION PROBABLY SECONDARY TO IP | 1 | 1 (0.44) | 0 | 0 (0.00) | 1 | 1 (0.88) | 0 | | 0 (0.00) |
| **Age Strata 1** | **0** | **0 (0.00)** | **0** | **0 (0.00)** | **0** | **0 (0.00)** | **0** | | **0 (0.00)** |
| **Local AE** | **0** | **0 (0.00)** | **0** | **0 (0.00)** | **0** | **0 (0.00)** | **0** | | **0 (0.00)** |
| Pain/Tenderness | 0 | 0 (0.00) | 0 | 0 (0.00) | 0 | 0 (0.00) | 0 | | 0 (0.00) |
| Erythema/Redness | 0 | 0 (0.00) | 0 | 0 (0.00) | 0 | 0 (0.00) | 0 | | 0 (0.00) |
| Swelling/Induration | 0 | 0 (0.00) | 0 | 0 (0.00) | 0 | 0 (0.00) | 0 | | 0 (0.00) |
| Pruritus | 0 | 0 (0.00) | 0 | 0 (0.00) | 0 | 0 (0.00) | 0 | | 0 (0.00) |
| **Systemic AE** | **0** | **0 (0.00)** | **0** | **0 (0.00)** | **0** | **0 (0.00)** | **0** | | **0 (0.00)** |
| Fever | 0 | 0 (0.00) | 0 | 0 (0.00) | 0 | 0 (0.00) | 0 | | 0 (0.00) |
| Lethargy | 0 | 0 (0.00) | 0 | 0 (0.00) | 0 | 0 (0.00) | 0 | | 0 (0.00) |
| Irritability | 0 | 0 (0.00) | 0 | 0 (0.00) | 0 | 0 (0.00) | 0 | | 0 (0.00) |
| Vomiting | 0 | 0 (0.00) | 0 | 0 (0.00) | 0 | 0 (0.00) | 0 | | 0 (0.00) |
| Diarrhea | 0 | 0 (0.00) | 0 | 0 (0.00) | 0 | 0 (0.00) | 0 | | 0 (0.00) |
| Drowsiness | 0 | 0 (0.00) | 0 | 0 (0.00) | 0 | 0 (0.00) | 0 | | 0 (0.00) |
| Loss of appetite | 0 | 0 (0.00) | 0 | 0 (0.00) | 0 | 0 (0.00) | 0 | | 0 (0.00) |
| Persistent crying | 0 | 0 (0.00) | 0 | 0 (0.00) | 0 | 0 (0.00) | 0 | | 0 (0.00) |
| **Age Strata 2** | **3** | **1 (1.32)** | **0** | **0 (0.00)** | **3** | **1 (2.63)** | **0** | | **0 (0.00)** |
| **Local AE** | **1** | **1 (1.32)** | **0** | **0 (0.00)** | **1** | **1 (2.63)** | **0** | | **0 (0.00)** |
| Pain/Tenderness | 0 | 0 (0.00) | 0 | 0 (0.00) | 0 | 0 (0.00) | 0 | | 0 (0.00) |
| Erythema/Redness | 1 | 1 (1.32) | 0 | 0 (0.00) | 1 | 1 (2.63) | 0 | | 0 (0.00) |
| Swelling/Induration | 0 | 0 (0.00) | 0 | 0 (0.00) | 0 | 0 (0.00) | 0 | | 0 (0.00) |
| Pruritus | 0 | 0 (0.00) | 0 | 0 (0.00) | 0 | 0 (0.00) | 0 | | 0 (0.00) |
| **Systemic AE** | **1** | **1 (1.32)** | **0** | **0 (0.00)** | **1** | **1 (2.63)** | **0** | | **0 (0.00)** |
| Fever | 1 | 1 (1.32) | 0 | 0 (0.00) | 1 | 1 (2.63) | 0 | | 0 (0.00) |
| Lethargy | 0 | 0 (0.00) | 0 | 0 (0.00) | 0 | 0 (0.00) | 0 | | 0 (0.00) |
| Irritability | 0 | 0 (0.00) | 0 | 0 (0.00) | 0 | 0 (0.00) | 0 | | 0 (0.00) |
| Vomiting | 0 | 0 (0.00) | 0 | 0 (0.00) | 0 | 0 (0.00) | 0 | | 0 (0.00) |
| Diarrhea | 0 | 0 (0.00) | 0 | 0 (0.00) | 0 | 0 (0.00) | 0 | | 0 (0.00) |
| Drowsiness | 0 | 0 (0.00) | 0 | 0 (0.00) | 0 | 0 (0.00) | 0 | | 0 (0.00) |
| Loss of appetite | 0 | 0 (0.00) | 0 | 0 (0.00) | 0 | 0 (0.00) | 0 | | 0 (0.00) |
| Persistent crying | 0 | 0 (0.00) | 0 | 0 (0.00) | 0 | 0 (0.00) | 0 | | 0 (0.00) |
| Rash^*^ | 0 | 0 (0.00) | 0 | 0 (0.00) | 0 | 0 (0.00) | 0 | | 0 (0.00) |
| Nasopharyngitis^*^ | 0 | 0 (0.00) | 0 | 0 (0.00) | 0 | 0 (0.00) | 0 | | 0 (0.00) |
| **Other AE** | **1** | **1 (1.32)** | **0** | **0 (0.00)** | **1** | **1 (2.63)** | **0** | | **0 (0.00)** |
| T/C HYPERSENSITIVITY REACTION PROBABLY SECONDARY TO IP | 1 | 1 (1.32) | 0 | 0 (0.00) | 1 | 1 (2.63) | 0 | | 0 (0.00) |
| **Age Strata 3** | **0** | **0 (0.00)** | **0** | **0 (0.00)** | **0** | **0 (0.00)** | **0** | | **0 (0.00)** |
| **Local AE** | **0** | **0 (0.00)** | **0** | **0 (0.00)** | **0** | **0 (0.00)** | **0** | | **0 (0.00)** |
| Pain/Tenderness | 0 | 0 (0.00) | 0 | 0 (0.00) | 0 | 0 (0.00) | 0 | | 0 (0.00) |
| Erythema/Redness | 0 | 0 (0.00) | 0 | 0 (0.00) | 0 | 0 (0.00) | 0 | | 0 (0.00) |
| Swelling/Induration | 0 | 0 (0.00) | 0 | 0 (0.00) | 0 | 0 (0.00) | 0 | | 0 (0.00) |
| Pruritus | 0 | 0 (0.00) | 0 | 0 (0.00) | 0 | 0 (0.00) | 0 | | 0 (0.00) |
| **Systemic AE** | **0** | **0 (0.00)** | **0** | **0 (0.00)** | **0** | **0 (0.00)** | **0** | | **0 (0.00)** |
| Fever | 0 | 0 (0.00) | 0 | 0 (0.00) | 0 | 0 (0.00) | 0 | | 0 (0.00) |
| Lethargy | 0 | 0 (0.00) | 0 | 0 (0.00) | 0 | 0 (0.00) | 0 | | 0 (0.00) |
| Irritability | 0 | 0 (0.00) | 0 | 0 (0.00) | 0 | 0 (0.00) | 0 | | 0 (0.00) |
| Vomiting | 0 | 0 (0.00) | 0 | 0 (0.00) | 0 | 0 (0.00) | 0 | | 0 (0.00) |
| Diarrhea | 0 | 0 (0.00) | 0 | 0 (0.00) | 0 | 0 (0.00) | 0 | | 0 (0.00) |
| Drowsiness | 0 | 0 (0.00) | 0 | 0 (0.00) | 0 | 0 (0.00) | 0 | | 0 (0.00) |
| Loss of appetite | 0 | 0 (0.00) | 0 | 0 (0.00) | 0 | 0 (0.00) | 0 | | 0 (0.00) |
| Persistent crying | 0 | 0 (0.00) | 0 | 0 (0.00) | 0 | 0 (0.00) | 0 | | 0 (0.00) |
|  | **Vi-DT Group**  **(N=220)** | |  |  |  |  | **Comparator Group (N=55)** | | |
|  |  |  | **Single dose**  **(N=112)** | | **two-dose**  **(N=108)** | |  |  |  |
| **After second dose** | **# of AEs** | **Number of participants (%)** | **# of AEs** | **Number of participants (%)** | **# of AEs** | **Number of participants (%)** | **# of AEs** | | **Number of participants (%)** |
| **All ages** | 0 | 0 (0.00) | 0 | 0 (0.00) | 0 | 0 (0.00) | 0 | | 0 (0.00) |
| **Local AE** | 0 | 0 (0.00) | 0 | 0 (0.00) | 0 | 0 (0.00) | 0 | | 0 (0.00) |
| Pain/Tenderness | 0 | 0 (0.00) | 0 | 0 (0.00) | 0 | 0 (0.00) | 0 | | 0 (0.00) |
| Erythema/Redness | 0 | 0 (0.00) | 0 | 0 (0.00) | 0 | 0 (0.00) | 0 | | 0 (0.00) |
| Swelling/Induration | 0 | 0 (0.00) | 0 | 0 (0.00) | 0 | 0 (0.00) | 0 | | 0 (0.00) |
| Pruritus | 0 | 0 (0.00) | 0 | 0 (0.00) | 0 | 0 (0.00) | 0 | | 0 (0.00) |
| **Systemic AE** | **0** | **0 (0.00)** | **0** | **0 (0.00)** | **0** | **0 (0.00)** | **0** | | **0 (0.00)** |
| Fever | 0 | 0 (0.00) | 0 | 0 (0.00) | 0 | 0 (0.00) | 0 | | 0 (0.00) |
| Lethargy | 0 | 0 (0.00) | 0 | 0 (0.00) | 0 | 0 (0.00) | 0 | | 0 (0.00) |
| Irritability | 0 | 0 (0.00) | 0 | 0 (0.00) | 0 | 0 (0.00) | 0 | | 0 (0.00) |
| Vomiting | 0 | 0 (0.00) | 0 | 0 (0.00) | 0 | 0 (0.00) | 0 | | 0 (0.00) |
| Diarrhea | 0 | 0 (0.00) | 0 | 0 (0.00) | 0 | 0 (0.00) | 0 | | 0 (0.00) |
| Drowsiness | 0 | 0 (0.00) | 0 | 0 (0.00) | 0 | 0 (0.00) | 0 | | 0 (0.00) |
| Loss of appetite | 0 | 0 (0.00) | 0 | 0 (0.00) | 0 | 0 (0.00) | 0 | | 0 (0.00) |
| Persistent crying | 0 | 0 (0.00) | 0 | 0 (0.00) | 0 | 0 (0.00) | 0 | | 0 (0.00) |
| **Age Strata 1** | **0** | **0 (0.00)** | **0** | **0 (0.00)** | **0** | **0 (0.00)** | **0** | | **0 (0.00)** |
| **Local AE** | **0** | **0 (0.00)** | **0** | **0 (0.00)** | **0** | **0 (0.00)** | **0** | | **0 (0.00)** |
| Pain/Tenderness | 0 | 0 (0.00) | 0 | 0 (0.00) | 0 | 0 (0.00) | 0 | | 0 (0.00) |
| Erythema/Redness | 0 | 0 (0.00) | 0 | 0 (0.00) | 0 | 0 (0.00) | 0 | | 0 (0.00) |
| Swelling/Induration | 0 | 0 (0.00) | 0 | 0 (0.00) | 0 | 0 (0.00) | 0 | | 0 (0.00) |
| Pruritus | 0 | 0 (0.00) | 0 | 0 (0.00) | 0 | 0 (0.00) | 0 | | 0 (0.00) |
| **Systemic AE** | **0** | **0 (0.00)** | **0** | **0 (0.00)** | **0** | **0 (0.00)** | **0** | | **0 (0.00)** |
| Fever | 0 | 0 (0.00) | 0 | 0 (0.00) | 0 | 0 (0.00) | 0 | | 0 (0.00) |
| Lethargy | 0 | 0 (0.00) | 0 | 0 (0.00) | 0 | 0 (0.00) | 0 | | 0 (0.00) |
| Irritability | 0 | 0 (0.00) | 0 | 0 (0.00) | 0 | 0 (0.00) | 0 | | 0 (0.00) |
| Vomiting | 0 | 0 (0.00) | 0 | 0 (0.00) | 0 | 0 (0.00) | 0 | | 0 (0.00) |
| Diarrhea | 0 | 0 (0.00) | 0 | 0 (0.00) | 0 | 0 (0.00) | 0 | | 0 (0.00) |
| Drowsiness | 0 | 0 (0.00) | 0 | 0 (0.00) | 0 | 0 (0.00) | 0 | | 0 (0.00) |
| Loss of appetite | 0 | 0 (0.00) | 0 | 0 (0.00) | 0 | 0 (0.00) | 0 | | 0 (0.00) |
| Persistent crying | 0 | 0 (0.00) | 0 | 0 (0.00) | 0 | 0 (0.00) | 0 | | 0 (0.00) |
| **Age Strata 2** | **0** | **0 (0.00)** | **0** | **0 (0.00)** | **0** | **0 (0.00)** | **0** | | **0 (0.00)** |
| **Local AE** | **0** | **0 (0.00)** | **0** | **0 (0.00)** | **0** | **0 (0.00)** | **0** | | **0 (0.00)** |
| Pain/Tenderness | 0 | 0 (0.00) | 0 | 0 (0.00) | 0 | 0 (0.00) | 0 | | 0 (0.00) |
| Erythema/Redness | 0 | 0 (0.00) | 0 | 0 (0.00) | 0 | 0 (0.00) | 0 | | 0 (0.00) |
| Swelling/Induration | 0 | 0 (0.00) | 0 | 0 (0.00) | 0 | 0 (0.00) | 0 | | 0 (0.00) |
| Pruritus | 0 | 0 (0.00) | 0 | 0 (0.00) | 0 | 0 (0.00) | 0 | | 0 (0.00) |
| Systemic AE | 0 | 0 (0.00) | 0 | 0 (0.00) | 0 | 0 (0.00) | 0 | | 0 (0.00) |
| Fever | 0 | 0 (0.00) | 0 | 0 (0.00) | 0 | 0 (0.00) | 0 | | 0 (0.00) |
| Lethargy | 0 | 0 (0.00) | 0 | 0 (0.00) | 0 | 0 (0.00) | 0 | | 0 (0.00) |
| Irritability | 0 | 0 (0.00) | 0 | 0 (0.00) | 0 | 0 (0.00) | 0 | | 0 (0.00) |
| Vomiting | 0 | 0 (0.00) | 0 | 0 (0.00) | 0 | 0 (0.00) | 0 | | 0 (0.00) |
| Diarrhea | 0 | 0 (0.00) | 0 | 0 (0.00) | 0 | 0 (0.00) | 0 | | 0 (0.00) |
| Drowsiness | 0 | 0 (0.00) | 0 | 0 (0.00) | 0 | 0 (0.00) | 0 | | 0 (0.00) |
| Loss of appetite | 0 | 0 (0.00) | 0 | 0 (0.00) | 0 | 0 (0.00) | 0 | | 0 (0.00) |
| Persistent crying | 0 | 0 (0.00) | 0 | 0 (0.00) | 0 | 0 (0.00) | 0 | | 0 (0.00) |
| **Age Strata 3** | **0** | **0 (0.00)** | **0** | **0 (0.00)** | **0** | **0 (0.00)** | **0** | | **0 (0.00)** |
| **Local AE** | **0** | **0 (0.00)** | **0** | **0 (0.00)** | **0** | **0 (0.00)** | **0** | | **0 (0.00)** |
| Pain/Tenderness | 0 | 0 (0.00) | 0 | 0 (0.00) | 0 | 0 (0.00) | 0 | | 0 (0.00) |
| Erythema/Redness | 0 | 0 (0.00) | 0 | 0 (0.00) | 0 | 0 (0.00) | 0 | | 0 (0.00) |
| Swelling/Induration | 0 | 0 (0.00) | 0 | 0 (0.00) | 0 | 0 (0.00) | 0 | | 0 (0.00) |
| Pruritus | 0 | 0 (0.00) | 0 | 0 (0.00) | 0 | 0 (0.00) | 0 | | 0 (0.00) |
| **Systemic AE** | **0** | **0 (0.00)** | **0** | **0 (0.00)** | **0** | **0 (0.00)** | **0** | | **0 (0.00)** |
| Fever | 0 | 0 (0.00) | 0 | 0 (0.00) | 0 | 0 (0.00) | 0 | | 0 (0.00) |
| Lethargy | 0 | 0 (0.00) | 0 | 0 (0.00) | 0 | 0 (0.00) | 0 | | 0 (0.00) |
| Irritability | 0 | 0 (0.00) | 0 | 0 (0.00) | 0 | 0 (0.00) | 0 | | 0 (0.00) |
| Vomiting | 0 | 0 (0.00) | 0 | 0 (0.00) | 0 | 0 (0.00) | 0 | | 0 (0.00) |
| Diarrhea | 0 | 0 (0.00) | 0 | 0 (0.00) | 0 | 0 (0.00) | 0 | | 0 (0.00) |
| Drowsiness | 0 | 0 (0.00) | 0 | 0 (0.00) | 0 | 0 (0.00) | 0 | | 0 (0.00) |
| Loss of appetite | 0 | 0 (0.00) | 0 | 0 (0.00) | 0 | 0 (0.00) | 0 | | 0 (0.00) |
| Persistent crying | 0 | 0 (0.00) | 0 | 0 (0.00) | 0 | 0 (0.00) | 0 | | 0 (0.00) |
|  | **Vi-DT Group**  **(N=228)** | |  |  |  |  | **Comparator Group (N=57)** | | |
|  |  |  | **Single dose**  **(N=114)** | | **two-dose**  **(N=114)** | |  |  |  |
| **After any dose** | **# of AEs** | **Number of participants (%)** | **# of AEs** | **Number of participants (%)** | **# of AEs** | **Number of participants (%)** | **# of AEs** | | **Number of participants (%)** |
| **All ages** | **3** | **1 (0.44)** | **0** | **0 (0.00)** | **3** | **1 (0.88)** | **0** | | **0 (0.00)** |
| **Local AE** | **1** | **1 (0.44)** | **0** | **0 (0.00)** | **1** | **1 (0.88)** | **0** | | **0 (0.00)** |
| Pain/Tenderness | 0 | 0 (0.00) | 0 | 0 (0.00) | 0 | 0 (0.00) | 0 | | 0 (0.00) |
| Erythema/Redness | 1 | 1 (0.44) | 0 | 0 (0.00) | 1 | 1 (0.88) | 0 | | 0 (0.00) |
| Swelling/Induration | 0 | 0 (0.00) | 0 | 0 (0.00) | 0 | 0 (0.00) | 0 | | 0 (0.00) |
| Pruritus | 0 | 0 (0.00) | 0 | 0 (0.00) | 0 | 0 (0.00) | 0 | | 0 (0.00) |
| **Systemic AE** | **1** | **1 (0.44)** | **0** | **0 (0.00)** | **1** | **0 (0.88)** | **0** | | **0 (0.00)** |
| Fever | 1 | 1 (0.44) | 0 | 0 (0.00) | 1 | 1 (0.88) | 0 | | 0 (0.00) |
| Lethargy | 0 | 0 (0.00) | 0 | 0 (0.00) | 0 | 0 (0.00) | 0 | | 0 (0.00) |
| Irritability | 0 | 0 (0.00) | 0 | 0 (0.00) | 0 | 0 (0.00) | 0 | | 0 (0.00) |
| Vomiting | 0 | 0 (0.00) | 0 | 0 (0.00) | 0 | 0 (0.00) | 0 | | 0 (0.00) |
| Diarrhea | 0 | 0 (0.00) | 0 | 0 (0.00) | 0 | 0 (0.00) | 0 | | 0 (0.00) |
| Drowsiness | 0 | 0 (0.00) | 0 | 0 (0.00) | 0 | 0 (0.00) | 0 | | 0 (0.00) |
| Loss of appetite | 0 | 0 (0.00) | 0 | 0 (0.00) | 0 | 0 (0.00) | 0 | | 0 (0.00) |
| Persistent crying | 0 | 0 (0.00) | 0 | 0 (0.00) | 0 | 0 (0.00) | 0 | | 0 (0.00) |
| Rash^*^ | 0 | 0 (0.00) | 0 | 0 (0.00) | 0 | 0 (0.00) | 0 | | 0 (0.00) |
| Nasopharyngitis^*^ | 0 | 0 (0.00) | 0 | 0 (0.00) | 0 | 0 (0.00) | 0 | | 0 (0.00) |
| **Other AE** | **1** | **1 (0.44)** | **0** | **0 (0.00)** | **1** | **1 (0.88)** | **0** | | **0 (0.00)** |
| T/C HYPERSENSITIVITY REACTION PROBABLY SECONDARY TO IP | 1 | 1 (0.44) | 0 | 0 (0.00) | 1 | 1 (0.88) | 0 | | 0 (0.00) |
| **Age Strata 1** | **0** | **0 (0.00)** | **0** | **0 (0.00)** | **0** | **0 (0.00)** | **0** | | **0 (0.00)** |
| **Local AE** | **0** | **0 (0.00)** | **0** | **0 (0.00)** | **0** | **0 (0.00)** | **0** | | **0 (0.00)** |
| Pain/Tenderness | 0 | 0 (0.00) | 0 | 0 (0.00) | 0 | 0 (0.00) | 0 | | 0 (0.00) |
| Erythema/Redness | 0 | 0 (0.00) | 0 | 0 (0.00) | 0 | 0 (0.00) | 0 | | 0 (0.00) |
| Swelling/Induration | 0 | 0 (0.00) | 0 | 0 (0.00) | 0 | 0 (0.00) | 0 | | 0 (0.00) |
| Pruritus | 0 | 0 (0.00) | 0 | 0 (0.00) | 0 | 0 (0.00) | 0 | | 0 (0.00) |
| **Systemic AE** | **0** | **0 (0.00)** | **0** | **0 (0.00)** | **0** | **0 (0.00)** | **0** | | **0 (0.00)** |
| Fever | 0 | 0 (0.00) | 0 | 0 (0.00) | 0 | 0 (0.00) | 0 | | 0 (0.00) |
| Lethargy | 0 | 0 (0.00) | 0 | 0 (0.00) | 0 | 0 (0.00) | 0 | | 0 (0.00) |
| Irritability | 0 | 0 (0.00) | 0 | 0 (0.00) | 0 | 0 (0.00) | 0 | | 0 (0.00) |
| Vomiting | 0 | 0 (0.00) | 0 | 0 (0.00) | 0 | 0 (0.00) | 0 | | 0 (0.00) |
| Diarrhea | 0 | 0 (0.00) | 0 | 0 (0.00) | 0 | 0 (0.00) | 0 | | 0 (0.00) |
| Drowsiness | 0 | 0 (0.00) | 0 | 0 (0.00) | 0 | 0 (0.00) | 0 | | 0 (0.00) |
| Loss of appetite | 0 | 0 (0.00) | 0 | 0 (0.00) | 0 | 0 (0.00) | 0 | | 0 (0.00) |
| Persistent crying | 0 | 0 (0.00) | 0 | 0 (0.00) | 0 | 0 (0.00) | 0 | | 0 (0.00) |
| **Age Strata 2** | **3** | **1 (1.32)** | **0** | **0 (0.00)** | **3** | **1 (2.63)** | **0** | | **0 (0.00)** |
| **Local AE** | **3** | **1 (1.32)** | **0** | **0 (0.00)** | **3** | **1 (2.63)** | **0** | | **0 (0.00)** |
| Pain/Tenderness | 0 | 0 (0.00) | 0 | 0 (0.00) | 0 | 0 (0.00) | 0 | | 0 (0.00) |
| Erythema/Redness | 1 | 1 (1.32) | 0 | 0 (0.00) | 1 | 1 (2.63) | 0 | | 0 (0.00) |
| Swelling/Induration | 0 | 0 (0.00) | 0 | 0 (0.00) | 0 | 0 (0.00) | 0 | | 0 (0.00) |
| Pruritus | 0 | 0 (0.00) | 0 | 0 (0.00) | 0 | 0 (0.00) | 0 | | 0 (0.00) |
| **Systemic AE** | **1** | **1 (1.32)** | **0** | **0 (0.00)** | **1** | **1 (2.63)** | **0** | | **0 (0.00)** |
| Fever | 1 | 1 (1.32) | 0 | 0 (0.00) | 1 | 1 (2.63) | 0 | | 0 (0.00) |
| Lethargy | 0 | 0 (0.00) | 0 | 0 (0.00) | 0 | 0 (0.00) | 0 | | 0 (0.00) |
| Irritability | 0 | 0 (0.00) | 0 | 0 (0.00) | 0 | 0 (0.00) | 0 | | 0 (0.00) |
| Vomiting | 0 | 0 (0.00) | 0 | 0 (0.00) | 0 | 0 (0.00) | 0 | | 0 (0.00) |
| Diarrhea | 0 | 0 (0.00) | 0 | 0 (0.00) | 0 | 0 (0.00) | 0 | | 0 (0.00) |
| Drowsiness | 0 | 0 (0.00) | 0 | 0 (0.00) | 0 | 0 (0.00) | 0 | | 0 (0.00) |
| Loss of appetite | 0 | 0 (0.00) | 0 | 0 (0.00) | 0 | 0 (0.00) | 0 | | 0 (0.00) |
| Persistent crying | 0 | 0 (0.00) | 0 | 0 (0.00) | 0 | 0 (0.00) | 0 | | 0 (0.00) |
| Rash^*^ | 0 | 0 (0.00) | 0 | 0 (0.00) | 0 | 0 (0.00) | 0 | | 0 (0.00) |
| Nasopharyngitis^*^ | 0 | 0 (0.00) | 0 | 0 (0.00) | 0 | 0 (0.00) | 0 | | 0 (0.00) |
| **Other AE** | **1** | **1 (1.32)** | **0** | **0 (0.00)** | **1** | **1 (2.63)** | **0** | | **0 (0.00)** |
| T/C HYPERSENSITIVITY REACTION PROBABLY SECONDARY TO IP | 1 | 1 (1.32) | 0 | 0 (0.00) | 1 | 1 (2.63) | 0 | | 0 (0.00) |
| **Age Strata 3** | **0** | **0 (0.00)** | **0** | **0 (0.00)** | **0** | **0 (0.00)** | **0** | | **0 (0.00)** |
| **Local AE** | **0** | **0 (0.00)** | **0** | **0 (0.00)** | **0** | **0 (0.00)** | **0** | | **0 (0.00)** |
| Pain/Tenderness | 0 | 0 (0.00) | 0 | 0 (0.00) | 0 | 0 (0.00) | 0 | | 0 (0.00) |
| Erythema/Redness | 0 | 0 (0.00) | 0 | 0 (0.00) | 0 | 0 (0.00) | 0 | | 0 (0.00) |
| Swelling/Induration | 0 | 0 (0.00) | 0 | 0 (0.00) | 0 | 0 (0.00) | 0 | | 0 (0.00) |
| Pruritus | 0 | 0 (0.00) | 0 | 0 (0.00) | 0 | 0 (0.00) | 0 | | 0 (0.00) |
| **Systemic AE** | **0** | **0 (0.00)** | **0** | **0 (0.00)** | **0** | **0 (0.00)** | **0** | | **0 (0.00)** |
| Fever | 0 | 0 (0.00) | 0 | 0 (0.00) | 0 | 0 (0.00) | 0 | | 0 (0.00) |
| Lethargy | 0 | 0 (0.00) | 0 | 0 (0.00) | 0 | 0 (0.00) | 0 | | 0 (0.00) |
| Irritability | 0 | 0 (0.00) | 0 | 0 (0.00) | 0 | 0 (0.00) | 0 | | 0 (0.00) |
| Vomiting | 0 | 0 (0.00) | 0 | 0 (0.00) | 0 | 0 (0.00) | 0 | | 0 (0.00) |
| Diarrhea | 0 | 0 (0.00) | 0 | 0 (0.00) | 0 | 0 (0.00) | 0 | | 0 (0.00) |
| Drowsiness | 0 | 0 (0.00) | 0 | 0 (0.00) | 0 | 0 (0.00) | 0 | | 0 (0.00) |
| Loss of appetite | 0 | 0 (0.00) | 0 | 0 (0.00) | 0 | 0 (0.00) | 0 | | 0 (0.00) |
| Persistent crying | 0 | 0 (0.00) | 0 | 0 (0.00) | 0 | 0 (0.00) | 0 | | 0 (0.00) |

* Only for 2^nd^ age strata who got MMR vaccination

Table S7 Distribution of solicited adverse events

|  | **Vi-DT Group**  **(N=228)** | |  |  |  |  | **Comparator Group (N=57)** | |
| --- | --- | --- | --- | --- | --- | --- | --- | --- |
|  |  |  | **Single dose**  **(N=114)** | | **two-dose**  **(N=114)** | |  |  |
| **Within 7 days after first dose** | **# of AEs** | **Number of participants (%)** | **# of AEs** | **Number of participants (%)** | **# of AEs** | **Number of participants (%)** | **# of AEs** | **Number of participants (%)** |
| **All ages** | **158** | **59 (25.88)** | **64** | **32 (28.07)** | **94** | **27 (23.68)** | **32** | **11 (19.30)** |
| **Local AE** | **25** | **15 (6.58)** | **11** | **6 (5.26)** | **14** | **9 (7.89)** | **2** | **2 (3.51)** |
| Pain/Tenderness | 10 | 10 (4.39) | 5 | 5 (4.39) | 5 | 5 (4.39) | 1 | 1 (1.75) |
| Erythema/Redness | 7 | 7 (3.07) | 3 | 3 (2.63) | 4 | 4 (3.51) | 0 | 0 (0.00) |
| Swelling/Induration | 5 | 5 (2.19) | 2 | 2 (1.75) | 3 | 3 (2.63) | 0 | 0 (0.00) |
| Pruritus | 3 | 3 (1.32) | 1 | 1 (0.88) | 2 | 2 (1.75) | 1 | 1 (1.75) |
| **Systemic AE** | **133** | **54 (23.68)** | **53** | **29 (25.44)** | **80** | **25 (21.93)** | **30** | **10 (17.54)** |
| Fever | 24 | 24 (10.53) | 11 | 11 (9.65) | 13 | 13 (11.40) | 3 | 3 (5.26) |
| Lethargy | 15 | 13 (5.70) | 7 | 7 (6.14) | 8 | 6 (5.26) | 4 | 4 (7.02) |
| Irritability | 15 | 13 (5.70) | 6 | 6 (5.26) | 9 | 7 (6.14) | 3 | 3 (5.26) |
| Vomiting | 8 | 8 (3.51) | 3 | 3 (2.63) | 5 | 5 (4.39) | 3 | 3 (5.26) |
| Diarrhea | 19 | 19 (8.33) | 9 | 9 (7.89) | 10 | 10 (8.77) | 6 | 6 (10.53) |
| Drowsiness | 5 | 5 (2.19) | 1 | 1 (0.88) | 4 | 4 (3.51) | 0 | 0 (0.00) |
| Loss of appetite | 13 | 12 (5.26) | 3 | 3 (2.63) | 10 | 9 (7.89) | 3 | 3 (5.26) |
| Persistent crying | 11 | 8 (3.51) | 2 | 2 (1.75) | 9 | 6 (5.26) | 3 | 3 (5.26) |
| Rash^*^ | 3 | 3 (3.95) | 2 | 2 (5.26) | 1 | 1 (2.63) | 0 | 0 (0.00) |
| Nasopharyngitis^*^ | 20 | 18 (23.68) | 9 | 9 (23.68) | 11 | 9 (23.68) | 5 | 4 (21.05) |
| **Age Strata 1** | **43** | **17 (22.37)** | **19** | **10 (26.32)** | **24** | **7 (18.42)** | **10** | **4 (21.05)** |
| **Local AE** | **8** | **3 (3.95)** | **4** | **2 (5.26)** | **4** | **1 (2.63)** | **1** | **1 (5.26)** |
| Pain/Tenderness | 3 | 3 (3.95) | 2 | 2 (5.26) | 1 | 1 (2.63) | 1 | 1 (5.26) |
| Erythema/Redness | 2 | 2 (2.63) | 1 | 1 (2.63) | 1 | 1 (2.63) | 0 | 0 (0.00) |
| Swelling/Induration | 2 | 2 (2.63) | 1 | 1 (2.63) | 1 | 1 (2.63) | 0 | 0 (0.00) |
| Pruritus | 1 | 1 (1.32) | 0 | 0 (0.00) | 1 | 1 (2.63) | 0 | 0 (0.00) |
| **Systemic AE** | **35** | **15 (19.74)** | **15** | **9 (23.68)** | **20** | **6 (15.79)** | **9** | **3 (15.79)** |
| Fever | 8 | 8 (10.53) | 5 | 5 (13.16) | 3 | 3 (7.89) | 1 | 1 (5.26) |
| Lethargy | 6 | 5 (6.58) | 4 | 4 (10.53) | 2 | 1 (2.63) | 1 | 1 (5.26) |
| Irritability | 3 | 3 (3.95) | 1 | 1 (2.63) | 2 | 2 (5.26) | 1 | 1 (5.26) |
| Vomiting | 3 | 3 (3.95) | 1 | 1 (2.63) | 2 | 2 (5.26) | 1 | 1 (5.26) |
| Diarrhea | 7 | 7 (9.21) | 3 | 3 (7.89) | 4 | 4 (10.53) | 2 | 2 (10.53) |
| Drowsiness | 2 | 2 (2.63) | 0 | 0 (0.00) | 2 | 2 (5.26) | 0 | 0 (0.00) |
| Loss of appetite | 3 | 3 (3.95) | 1 | 1 (2.63) | 2 | 2 (5.26) | 2 | 2 (10.53) |
| Persistent crying | 3 | 2 (2.63) | 0 | 0 (0.00) | 3 | 2 (5.26) | 1 | 1 (5.26) |
| **Age Strata 2** | **88** | **29 (38.16)** | **32** | **14 (36.84)** | **56** | **15 (39.47)** | **12** | **5 (26.32)** |
| **Local AE** | **9** | **7 (9.21)** | **1** | **1 (2.63)** | **8** | **6 (15.79)** | **0** | **0 (0.00)** |
| Pain/Tenderness | 4 | 4 (5.26) | 0 | 0 (0.00) | 4 | 4 (10.53) | 0 | 0 (0.00) |
| Erythema/Redness | 3 | 3 (3.95) | 1 | 1 (2.63) | 2 | 2 (5.26) | 0 | 0 (0.00) |
| Swelling/Induration | 1 | 1 (1.32) | 0 | 0 (0.00) | 1 | 1 (2.63) | 0 | 0 (0.00) |
| Pruritus | 1 | 1 (1.32) | 0 | 0 (0.00) | 1 | 1 (2.63) | 0 | 0 (0.00) |
| **Systemic AE** | **79** | **28 (36.84)** | **31** | **14 (36.84)** | **48** | **14 (36.84)** | **12** | **5 (26.32)** |
| Fever | 10 | 10 (13.16) | 3 | 3 (7.89) | 7 | 7 (18.42) | 0 | 0 (0.00) |
| Lethargy | 8 | 7 (9.21) | 3 | 3 (7.89) | 5 | 4 (10.53) | 2 | 2 (10.53) |
| Irritability | 10 | 8 (10.53) | 4 | 4 (10.53) | 6 | 4 (10.53) | 1 | 1 (5.26) |
| Vomiting | 4 | 4 (5.26) | 1 | 1 (2.63) | 3 | 3 (7.89) | 1 | 1 (5.26) |
| Diarrhea | 7 | 7 (9.21) | 4 | 4 (10.53) | 3 | 3 (7.89) | 2 | 2 (10.53) |
| Drowsiness | 3 | 3 (3.95) | 1 | 1 (2.63) | 2 | 2 (5.26) | 0 | 0 (0.00) |
| Loss of appetite | 7 | 6 (7.89) | 2 | 2 (5.26) | 5 | 4 (10.53) | 0 | 0 (0.00) |
| Persistent crying | 7 | 5 (6.58) | 2 | 2 (5.26) | 5 | 3 (7.89) | 1 | 1 (5.26) |
| Rash^*^ | 3 | 3 (3.95) | 2 | 2 (5.26) | 1 | 1 (2.63) | 0 | 0 (0.00) |
| Nasopharyngitis^*^ | 20 | 18 (23.68) | 9 | 9 (23.68) | 11 | 9 (23.68) | 5 | 4 (21.05) |
| **Age Strata 3** | **27** | **13 (17.11)** | **13** | **8 (21.05)** | **14** | **5 (13.16)** | **10** | **2 (10.53)** |
| **Local AE** | **8** | **5 (6.58)** | **6** | **3 (7.89)** | **2** | **2 (5.26)** | **1** | **1 (5.26)** |
| Pain/Tenderness | 3 | 3 (3.95) | 3 | 3 (7.89) | 0 | 0 (0.00) | 0 | 0 (0.00) |
| Erythema/Redness | 2 | 2 (2.63) | 1 | 1 (2.63) | 1 | 1 (2.63) | 0 | 0 (0.00) |
| Swelling/Induration | 2 | 2 (2.63) | 1 | 1 (2.63) | 1 | 1 (2.63) | 0 | 0 (0.00) |
| Pruritus | 1 | 1 (1.32) | 1 | 1 (2.63) | 0 | 0 (0.00) | 1 | 1 (5.26) |
| **Systemic AE** | **19** | **11 (14.47)** | **7** | **6 (15.79)** | **12** | **5 (13.16)** | **9** | **2 (10.53)** |
| Fever | 6 | 6 (7.89) | 3 | 3 (7.89) | 3 | 3 (7.89) | 2 | 2 (10.53) |
| Lethargy | 1 | 1 (1.32) | 0 | 0 (0.00) | 1 | 1 (2.63) | 1 | 1 (5.26) |
| Irritability | 2 | 2 (2.63) | 1 | 1 (2.63) | 1 | 1 (2.63) | 1 | 1 (5.26) |
| Vomiting | 1 | 1 (1.32) | 1 | 1 (2.63) | 0 | 0 (0.00) | 1 | 1 (5.26) |
| Diarrhea | 5 | 5 (6.58) | 2 | 2 (5.26) | 3 | 3 (7.89) | 2 | 2 (10.53) |
| Drowsiness | 0 | 0 (0.00) | 0 | 0 (0.00) | 0 | 0 (0.00) | 0 | 0 (0.00) |
| Loss of appetite | 3 | 3 (3.95) | 0 | 0 (0.00) | 3 | 3 (7.89) | 1 | 1 (5.26) |
| Persistent crying | 1 | 1 (1.32) | 0 | 0 (0.00) | 1 | 1 (2.63) | 1 | 1 (5.26) |
|  | **Vi-DT Group**  **(N=220)** | |  |  |  |  | **Comparator Group (N=55)** | |
|  |  |  | **Single dose**  **(N=112)** | | **two-dose**  **(N=108)** | |  |  |
| **Within 7 days after second dose** | **# of AEs** | **Number of participants (%)** | **# of AEs** | **Number of participants (%)** | **# of AEs** | **Number of participants (%)** | **# of AEs** | **Number of participants (%)** |
| **All ages** | **55** | **25 (11.36)** | **40** | **18 (16.07)** | **15** | **7 (6.48)** | **15** | **5 (9.09)** |
| **Local AE** | **8** | **4 (1.82)** | **6** | **2 (1.79)** | **2** | **2 (1.85)** | **4** | **1 (1.82)** |
| Pain/Tenderness | 3 | 3 (1.36) | 1 | 1 (0.89) | 2 | 2 (1.85) | 1 | 1 (1.82) |
| Erythema/Redness | 2 | 2 (0.91) | 2 | 2 (1.79) | 0 | 0 (0.00) | 1 | 1 (1.82) |
| Swelling/Induration | 1 | 1 (0.45) | 1 | 1 (0.89) | 0 | 0 (0.00) | 1 | 1 (1.82) |
| Pruritus | 2 | 2 (0.91) | 2 | 2 (1.79) | 0 | 0 (0.00) | 1 | 1 (1.82) |
| **Systemic AE** | **47** | **24 (10.91)** | **34** | **18 (16.07)** | **13** | **6 (5.56)** | **11** | **5 (9.09)** |
| Fever | 19 | 19 (8.64) | 15 | 15 (13.39) | 4 | 4 (3.70) | 2 | 2 (3.64) |
| Lethargy | 4 | 4 (1.82) | 2 | 2 (1.79) | 2 | 2 (1.85) | 1 | 1 (1.82) |
| Irritability | 2 | 2 (0.91) | 1 | 1 (0.89) | 1 | 1 (0.93) | 2 | 2 (3.64) |
| Vomiting | 5 | 5 (2.27) | 3 | 3 (2.68) | 2 | 2 (1.85) | 1 | 1 (1.82) |
| Diarrhea | 5 | 5 (2.27) | 5 | 5 (4.46) | 0 | 0 (0.00) | 1 | 1 (1.82) |
| Drowsiness | 3 | 3 (1.36) | 2 | 2 (1.79) | 1 | 1 (0.93) | 1 | 1 (1.82) |
| Loss of appetite | 5 | 5 (2.27) | 4 | 4 (3.57) | 1 | 1 (0.93) | 2 | 2 (3.64) |
| Persistent crying | 4 | 4 (1.82) | 2 | 2 (1.79) | 2 | 2 (1.85) | 1 | 1 (1.82) |
| **Age Strata 1** | **7** | **5 (6.67)** | **7** | **5 (13.16)** | **0** | **0 (0.00)** | **9** | **2 (11.76)** |
| **Local AE** | **0** | **0 (0.00)** | **0** | **0 (0.00)** | **0** | **0 (0.00)** | **4** | **1 (5.88)** |
| Pain/Tenderness | 0 | 0 (0.00) | 0 | 0 (0.00) | 0 | 0 (0.00) | 1 | 1 (5.88) |
| Erythema/Redness | 0 | 0 (0.00) | 0 | 0 (0.00) | 0 | 0 (0.00) | 1 | 1 (5.88) |
| Swelling/Induration | 0 | 0 (0.00) | 0 | 0 (0.00) | 0 | 0 (0.00) | 1 | 1 (5.88) |
| Pruritus | 0 | 0 (0.00) | 0 | 0 (0.00) | 0 | 0 (0.00) | 1 | 1 (5.88) |
| **Systemic AE** | **7** | **5 (6.67)** | **7** | **5 (13.16)** | **0** | **0 (0.00)** | **5** | **2 (11.76)** |
| Fever | 5 | 5 (6.67) | 5 | 5 (13.16) | 0 | 0 (0.00) | 0 | 0 (0.00) |
| Lethargy | 0 | 0 (0.00) | 0 | 0 (0.00) | 0 | 0 (0.00) | 0 | 0 (0.00) |
| Irritability | 0 | 0 (0.00) | 0 | 0 (0.00) | 0 | 0 (0.00) | 1 | 1 (5.88) |
| Vomiting | 0 | 0 (0.00) | 0 | 0 (0.00) | 0 | 0 (0.00) | 1 | 1 (5.88) |
| Diarrhea | 1 | 1 (1.33) | 1 | 1 (2.63) | 0 | 0 (0.00) | 1 | 1 (5.88) |
| Drowsiness | 0 | 0 (0.00) | 0 | 0 (0.00) | 0 | 0 (0.00) | 0 | 0 (0.00) |
| Loss of appetite | 1 | 1 (1.33) | 1 | 1 (2.63) | 0 | 0 (0.00) | 1 | 1 (5.88) |
| Persistent crying | 0 | 0 (0.00) | 0 | 0 (0.00) | 0 | 0 (0.00) | 1 | 1 (5.88) |
| **Age Strata 2** | **29** | **9 (13.04)** | **22** | **7 (19.44)** | **7** | **2 (6.06)** | **4** | **1 (5.26)** |
| **Local AE** | **7** | **3 (4.35)** | **6** | **2 (5.56)** | **1** | **1 (3.03)** | **0** | **0 (0.00)** |
| Pain/Tenderness | 2 | 2 (2.90) | 1 | 1 (2.78) | 1 | 1 (3.03) | 0 | 0 (0.00) |
| Erythema/Redness | 2 | 2 (2.90) | 2 | 2 (5.56) | 0 | 0 (0.00) | 0 | 0 (0.00) |
| Swelling/Induration | 1 | 1 (1.45) | 1 | 1 (2.78) | 0 | 0 (0.00) | 0 | 0 (0.00) |
| Pruritus | 2 | 2 (2.90) | 2 | 2 (5.56) | 0 | 0 (0.00) | 0 | 0 (0.00) |
| **Systemic AE** | **22** | **9 (13.04)** | **16** | **7 (19.44)** | **6** | **2 (6.06)** | **4** | **1 (5.26)** |
| Fever | 6 | 6 (8.70) | 5 | 5 (13.89) | 1 | 1 (3.03) | 0 | 0 (0.00) |
| Lethargy | 2 | 2 (2.90) | 1 | 1 (2.78) | 1 | 1 (3.03) | 1 | 1 (5.26) |
| Irritability | 2 | 2 (2.90) | 1 | 1 (2.78) | 1 | 1 (3.03) | 1 | 1 (5.26) |
| Vomiting | 2 | 2 (2.90) | 1 | 1 (2.78) | 1 | 1 (3.03) | 0 | 0 (0.00) |
| Diarrhea | 3 | 3 (4.35) | 3 | 3 (8.33) | 0 | 0 (0.00) | 0 | 0 (0.00) |
| Drowsiness | 3 | 3 (4.35) | 2 | 2 (5.56) | 1 | 1 (3.03) | 1 | 1 (5.26) |
| Loss of appetite | 1 | 1 (1.45) | 1 | 1 (2.78) | 0 | 0 (0.00) | 1 | 1 (5.26) |
| Persistent crying | 3 | 3 (4.35) | 2 | 2 (5.56) | 1 | 1 (3.03) | 0 | 0 (0.00) |
| **Age Strata 3** | **19** | **11 (14.47)** | **11** | **6 (15.79)** | **8** | **5 (13.16)** | **2** | **2 (10.53)** |
| **Local AE** | **1** | **1 (1.32)** | **0** | **0 (0.00)** | **1** | **1 (2.63)** | **0** | **0 (0.00)** |
| Pain/Tenderness | 1 | 1 (1.32) | 0 | 0 (0.00) | 1 | 1 (2.63) | 0 | 0 (0.00) |
| Erythema/Redness | 0 | 0 (0.00) | 0 | 0 (0.00) | 0 | 0 (0.00) | 0 | 0 (0.00) |
| Swelling/Induration | 0 | 0 (0.00) | 0 | 0 (0.00) | 0 | 0 (0.00) | 0 | 0 (0.00) |
| Pruritus | 0 | 0 (0.00) | 0 | 0 (0.00) | 0 | 0 (0.00) | 0 | 0 (0.00) |
| **Systemic AE** | **18** | **10 (13.16)** | **11** | **6 (15.79)** | **7** | **4 (10.53)** | **2** | **2 (10.53)** |
| Fever | 8 | 8 (10.53) | 5 | 5 (13.16) | 3 | 3 (7.89) | 2 | 2 (10.53) |
| Lethargy | 2 | 2 (2.63) | 1 | 1 (2.63) | 1 | 1 (2.63) | 0 | 0 (0.00) |
| Irritability | 0 | 0 (0.00) | 0 | 0 (0.00) | 0 | 0 (0.00) | 0 | 0 (0.00) |
| Vomiting | 3 | 3 (3.95) | 2 | 2 (5.26) | 1 | 1 (2.63) | 0 | 0 (0.00) |
| Diarrhea | 1 | 1 (1.32) | 1 | 1 (2.63) | 0 | 0 (0.00) | 0 | 0 (0.00) |
| Drowsiness | 0 | 0 (0.00) | 0 | 0 (0.00) | 0 | 0 (0.00) | 0 | 0 (0.00) |
| Loss of appetite | 3 | 3 (3.95) | 2 | 2 (5.26) | 1 | 1 (2.63) | 0 | 0 (0.00) |
| Persistent crying | 1 | 1 (1.32) | 0 | 0 (0.00) | 1 | 1 (2.63) | 0 | 0 (0.00) |
|  | **Vi-DT Group**  **(N=228)** | |  |  |  |  | **Comparator Group (N=57)** | |
|  |  |  | **Single dose**  **(N=114)** | | **two-dose**  **(N=114)** | |  |  |
| **Within 7 days after any dose** | **# of AEs** | **Number of participants (%)** | **# of AEs** | **Number of participants (%)** | **# of AEs** | **Number of participants (%)** | **# of AEs** | **Number of participants (%)** |
| **All ages** | **213** | **77 (33.77)** | **104** | **45 (39.47)** | **109** | **32 (28.07)** | **47** | **13 (22.81)** |
| **Local AE** | **33** | **18 (7.89)** | **17** | **7 (6.14)** | **16** | **11 (9.65)** | **6** | **3 (5.26)** |
| Pain/Tenderness | 13 | 13 (5.70) | 6 | 6 (5.26) | 7 | 7 (6.14) | 2 | 2 (3.51) |
| Erythema/Redness | 9 | 8 (3.51) | 5 | 4 (3.51) | 4 | 4 (3.51) | 1 | 1 (1.75) |
| Swelling/Induration | 6 | 6 (2.63) | 3 | 3 (2.63) | 3 | 3 (2.63) | 1 | 1 (1.75) |
| Pruritus | 5 | 5 (2.19) | 3 | 3 (2.63) | 2 | 2 (1.75) | 2 | 2 (3.51) |
| **Systemic AE** | **180** | **71 (31.14)** | **87** | **42 (36.84)** | **93** | **29 (25.44)** | **41** | **12 (21.05)** |
| Fever | 43 | 41 (17.98) | 26 | 25 (21.93) | 17 | 16 (14.04) | 5 | 4 (7.02) |
| Lethargy | 19 | 15 (6.58) | 9 | 8 (7.02) | 10 | 7 (6.14) | 5 | 5 (8.77) |
| Irritability | 17 | 13 (5.70) | 7 | 6 (5.26) | 10 | 7 (6.14) | 5 | 5 (8.77) |
| Vomiting | 13 | 13 (5.70) | 6 | 6 (5.26) | 7 | 7 (6.14) | 4 | 4 (7.02) |
| Diarrhea | 24 | 23 (10.09) | 14 | 13 (11.40) | 10 | 10 (8.77) | 7 | 6 (10.53) |
| Drowsiness | 8 | 7 (3.07) | 3 | 3 (2.63) | 5 | 4 (3.51) | 1 | 1 (1.75) |
| Loss of appetite | 18 | 17 (7.46) | 7 | 7 (6.14) | 11 | 10 (8.77) | 5 | 4 (7.02) |
| Persistent crying | 15 | 10 (4.39) | 4 | 3 (2.63) | 11 | 7 (6.14) | 4 | 4 (7.02) |
| Rash^*^ | 3 | 3 (3.95) | 2 | 2 (5.26) | 1 | 1 (2.63) | 0 | 0 (0.00) |
| Nasopharyngitis^*^ | 20 | 18 (23.68) | 9 | 9 (23.68) | 11 | 9 (23.68) | 5 | 4 (21.05) |
| **Age Strata 1** | **50** | **21 (27.63)** | **26** | **14 (36.84)** | **24** | **7 (18.42)** | **19** | **5 (26.32)** |
| **Local AE** | **8** | **3 (3.95)** | **4** | **2 (5.26)** | **4** | **1 (2.63)** | **5** | **2 (10.53)** |
| Pain/Tenderness | 3 | 3 (3.95) | 2 | 2 (5.26) | 1 | 1 (2.63) | 2 | 2 (10.53) |
| Erythema/Redness | 2 | 2 (2.63) | 1 | 1 (2.63) | 1 | 1 (2.63) | 1 | 1 (5.26) |
| Swelling/Induration | 2 | 2 (2.63) | 1 | 1 (2.63) | 1 | 1 (2.63) | 1 | 1 (5.26) |
| Pruritus | 1 | 1 (1.32) | 0 | 0 (0.00) | 1 | 1 (2.63) | 1 | 1 (5.26) |
| **Systemic AE** | **42** | **19 (25.00)** | **22** | **13 (34.21)** | **20** | **6 (15.79)** | **14** | **4 (21.05)** |
| Fever | 13 | 12 (15.79) | 10 | 9 (23.68) | 3 | 3 (7.89) | 1 | 1 (5.26) |
| Lethargy | 6 | 5 (6.58) | 4 | 4 (10.53) | 2 | 1 (2.63) | 1 | 1 (5.26) |
| Irritability | 3 | 3 (3.95) | 1 | 1 (2.63) | 2 | 2 (5.26) | 2 | 2 (10.53) |
| Vomiting | 3 | 3 (3.95) | 1 | 1 (2.63) | 2 | 2 (5.26) | 2 | 2 (10.53) |
| Diarrhea | 8 | 8 (10.53) | 4 | 4 (10.53) | 4 | 4 (10.53) | 3 | 2 (10.53) |
| Drowsiness | 2 | 2 (2.63) | 0 | 0 (0.00) | 2 | 2 (5.26) | 0 | 0 (0.00) |
| Loss of appetite | 4 | 4 (5.26) | 2 | 2 (5.26) | 2 | 2 (5.26) | 3 | 2 (10.53) |
| Persistent crying | 3 | 2 (2.63) | 0 | 0 (0.00) | 3 | 2 (5.26) | 2 | 2 (10.53) |
| **Age Strata 2** | **117** | **34 (44.74)** | **54** | **18 (47.37)** | **63** | **16 (42.11)** | **16** | **5 (26.32)** |
| **Local AE** | **16** | **9 (11.84)** | **7** | **2 (5.26)** | **9** | **7 (18.42)** | **0** | **0 (0.00)** |
| Pain/Tenderness | 6 | 6 (7.89) | 1 | 1 (2.63) | 5 | 5 (13.16) | 0 | 0 (0.00) |
| Erythema/Redness | 5 | 4 (5.26) | 3 | 2 (5.26) | 2 | 2 (5.26) | 0 | 0 (0.00) |
| Swelling/Induration | 2 | 2 (2.63) | 1 | 1 (2.63) | 1 | 1 (2.63) | 0 | 0 (0.00) |
| Pruritus | 3 | 3 (3.95) | 2 | 2 (5.26) | 1 | 1 (2.63) | 0 | 0 (0.00) |
| **Systemic AE** | **101** | **33 (43.42)** | **47** | **18 (47.37)** | **54** | **15 (39.47)** | **16** | **5 (26.32)** |
| Fever | 16 | 16 (21.05) | 8 | 8 (21.05) | 8 | 8 (21.05) | 0 | 0 (0.00) |
| Lethargy | 10 | 7 (9.21) | 4 | 3 (7.89) | 6 | 4 (10.53) | 3 | 3 (15.79) |
| Irritability | 12 | 8 (10.53) | 5 | 4 (10.53) | 7 | 4 (10.53) | 2 | 2 (10.53) |
| Vomiting | 6 | 6 (7.89) | 2 | 2 (5.26) | 4 | 4 (10.53) | 1 | 1 (5.26) |
| Diarrhea | 10 | 9 (11.84) | 7 | 6 (15.79) | 3 | 3 (7.89) | 2 | 2 (10.53) |
| Drowsiness | 6 | 5 (6.58) | 3 | 3 (7.89) | 3 | 2 (5.26) | 1 | 1 (5.26) |
| Loss of appetite | 8 | 7 (9.21) | 3 | 3 (7.89) | 5 | 4 (10.53) | 1 | 1 (5.26) |
| Persistent crying | 10 | 6 (7.89) | 4 | 3 (7.89) | 6 | 3 (7.89) | 1 | 1 (5.26) |
| Rash^*^ | 3 | 3 (3.95) | 2 | 2 (5.26) | 1 | 1 (2.63) | 0 | 0 (0.00) |
| Nasopharyngitis^*^ | 20 | 18 (23.68) | 9 | 9 (23.68) | 11 | 9 (23.68) | 5 | 4 (21.05) |
| **Age Strata 3** | **46** | **22 (28.95)** | **24** | **13 (34.21)** | **22** | **9 (23.68)** | **12** | **3 (15.79)** |
| **Local AE** | **9** | **6 (7.89)** | **6** | **3 (7.89)** | **3** | **3 (7.89)** | **1** | **1 (5.26)** |
| Pain/Tenderness | 4 | 4 (5.26) | 3 | 3 (7.89) | 1 | 1 (2.63) | 0 | 0 (0.00) |
| Erythema/Redness | 2 | 2 (2.63) | 1 | 1 (2.63) | 1 | 1 (2.63) | 0 | 0 (0.00) |
| Swelling/Induration | 2 | 2 (2.63) | 1 | 1 (2.63) | 1 | 1 (2.63) | 0 | 0 (0.00) |
| Pruritus | 1 | 1 (1.32) | 1 | 1 (2.63) | 0 | 0 (0.00) | 1 | 1 (5.26) |
| **Systemic AE** | **37** | **19 (25.00)** | **18** | **11 (28.95)** | **19** | **8 (21.05)** | **11** | **3 (15.79)** |
| Fever | 14 | 13 (17.11) | 8 | 8 (21.05) | 6 | 5 (13.16) | 4 | 3 (15.79) |
| Lethargy | 3 | 3 (3.95) | 1 | 1 (2.63) | 2 | 2 (5.26) | 1 | 1 (5.26) |
| Irritability | 2 | 2 (2.63) | 1 | 1 (2.63) | 1 | 1 (2.63) | 1 | 1 (5.26) |
| Vomiting | 4 | 4 (5.26) | 3 | 3 (7.89) | 1 | 1 (2.63) | 1 | 1 (5.26) |
| Diarrhea | 6 | 6 (7.89) | 3 | 3 (7.89) | 3 | 3 (7.89) | 2 | 2 (10.53) |
| Drowsiness | 0 | 0 (0.00) | 0 | 0 (0.00) | 0 | 0 (0.00) | 0 | 0 (0.00) |
| Loss of appetite | 6 | 6 (7.89) | 2 | 2 (5.26) | 4 | 4 (10.53) | 1 | 1 (5.26) |
| Persistent crying | 2 | 2 (2.63) | 0 | 0 (0.00) | 2 | 2 (5.26) | 1 | 1 (5.26) |

* Only for 2^nd^ age strata who got MMR vaccination

Table S8 Proportion of subjects with solicited AE within 7 days after each vaccination (Safety Analysis Set)

|  | **Vi-DT Group** | | | | | | | | | | **Group-C:**  **Comparator Group** | | |  |
| --- | --- | --- | --- | --- | --- | --- | --- | --- | --- | --- | --- | --- | --- | --- |
|  | **Total** | | | **Group-A: Single dose** | | | | **Group-B: Two-dose** | | |  |  |  |  |
| **Within 7 days after first dose** | **N** | **Number of participants (%)** | **95% CI** | **N** | **Number of participants (%)** | **95% CI** | **N** | | **Number of participants (%)** | **95% CI** | **N** | **Number of participants (%)** | **95% CI** | **P-value**† |
| All ages | 228 | 59 (25·88) | (20·63, 31·93) | 114 | 32 (28·07) | (20·64, 36·93) | 114 | | 27 (23·68) | (16·82, 32·27) | 57 | 11 (19·30) | (11·13, 31·34) | 0·4266 |
| AgeStrata1 | 76 | 17 (22·37) | (14·46, 32·93) | 38 | 10 (26·32) | (14·97, 42·01) | 38 | | 7 (18·42) | (9·22, 33·42) | 19 | 4 (21·05) | (8·51, 43·33) | -- |
| AgeStrata2 | 76 | 29 (38·16) | (28·06, 49·40) | 38 | 14 (36·84) | (23·38, 52·72) | 38 | | 15 (39·47) | (25·60, 55·28) | 19 | 5 (26·32) | (11·81, 48·79) | -- |
| AgeStrata3 | 76 | 13 (17·11) | (10·28, 27·10) | 38 | 8 (21·05) | (11·07, 36·35) | 38 | | 5 (13·16) | (5·75, 27·33) | 19 | 2 (10·53) | (2·94, 31·39) | -- |
| **Within 7 days after second dose** | **N** | **Number of participants (%)** | **95% CI** | **N** | **Number of participants (%)** | **95% CI** | **N** | | **Number of participants (%)** | **95% CI** | **N** | **Number of participants (%)** | **95% CI** | **P-value**† |
| All ages | 220 | 25 (11·36) | (7·82, 16·24) | 112 | 18 (16·07) | (10·41, 23·98) | 108 | | 7 (6·48) | (3·17, 12·78) | 55 | 5 (9·09) | (3·95, 19·58) | 0·0537 |
| AgeStrata1 | 75 | 5 (6·67) | (2·88, 14·68) | 38 | 5 (13·16) | (5·75, 27·33) | 37 | | 0 (0·00) | - | 17 | 2 (11·76) | (3·29, 34·34) | -- |
| AgeStrata2 | 69 | 9 (13·04) | (7·02, 22·97) | 36 | 7 (19·44) | (9·75, 35·03) | 33 | | 2 (6·06) | (1·68, 19·61) | 19 | 1 (5·26) | (0·94, 24·64) | -- |
| AgeStrata3 | 76 | 11 (14·47) | (8·28, 24·09) | 38 | 6 (15·79) | (7·44, 30·42) | 38 | | 5 (13·16) | (5·75, 27·33) | 19 | 2 (10·53) | (2·94, 31·39) | -- |
| **Within 7 days after any dose** | **N** | **Number of participants (%)** | **95% CI** | **N** | **Number of participants (%)** | **95% CI** | **N** | | **Number of participants (%)** | **95% CI** | **N** | **Number of participants (%)** | **95% CI** | **P-value**† |
| All ages | 228 | 77 (33·77) | (27·95, 40·13) | 114 | 45 (39·47) | (30·98, 48·65) | 114 | | 32 (28·07) | (20·64, 36·93) | 57 | 13 (22·81) | (13·84, 35·21) | 0·0490 |
| AgeStrata1 | 76 | 21 (27·63) | (18·84, 38·58) | 38 | 14 (36·84) | (23·38, 52·72) | 38 | | 7 (18·42) | (9·22, 33·42) | 19 | 5 (26·32) | (11·81, 48·79) | -- |
| AgeStrata2 | 76 | 34 (44·74) | (34·08, 55·90) | 38 | 18 (47·37) | (32·48, 62·74) | 38 | | 16 (42·11) | (27·85, 57·81) | 19 | 5 (26·32) | (11·81, 48·79) | -- |
| AgeStrata3 | 76 | 22 (28·95) | (19·96, 39·96) | 38 | 13 (34·21) | (21·21, 50·11) | 38 | | 9 (23·68) | (12·99, 39·21) | 19 | 3 (15·79) | (5·52, 37·57) | -- |

Table S9 Summary of Solicited Adverse Events [All ages] by relatedness

| **Within 4 weeks after first dose** | | **Vi-DT Group (N=228)** | |  | |  | | | **Comparator Group (N=57)** | | | |  |
| --- | --- | --- | --- | --- | --- | --- | --- | --- | --- | --- | --- | --- | --- |
|  |  |  |  | **single dose**  **(N=114)** | | **two-dose**  **(N=114)** | | |  |  |  |  |  |
|  |  | **Number of AEs** | **Number of Participants (%)** | **Number of AEs** | **Number of Participants (%)** | **Number of AEs** | **Number of Participants (%)** | | **Number of AEs** | | **Number of Participants (%)** | | **P-value**^†^ |
| **Solicited AE** (day 0 to day 7) | | **158** | **59 (25.88)** | **64** | **32 (28.07)** | **94** | **27 (23.68)** | | **32** | | **11 (19.30)** | |  |
| Relatedness: | Definitely Related | 17 | 10 (4.39) | 5 | 4 (3.51) | 12 | 6 (5.26) | | 0 | | 0 (0.00) | | 0.2109 |
|  | Probably Related | 64 | 31 (13.60) | 30 | 17 (14.91) | 34 | 14 (12.28) | | 11 | | 5 (8.77) | | 0.5199 |
|  | Possibly related | 77 | 29 (12.72) | 29 | 15 (13.16) | 48 | 14 (12.28) | | 21 | | 9 (15.79) | | 0.8075 |
|  | Unlikely related | 0 | 0 (0.00) | 0 | 0 (0.00) | 0 | 0 (0.00) | | 0 | | 0 (0.00) | | - |
|  | Not Related | 0 | 0 (0.00) | 0 | 0 (0.00) | 0 | 0 (0.00) | | 0 | | 0 (0.00) | | - |
| **Within 4 weeks after second dose** | | **Vi-DT Group (N=220)** | |  | |  | | **Comparator Group (N=55)** | | | | |  |
|  |  |  |  | **single dose (N=112)** | | **two-dose (N=108)** | |  |  |  |  |  |  |
|  |  | **Number of AEs** | **Number of Participants (%)** | **Number of AEs** | **Number of Participants (%)** | **Number of AEs** | **Number of Participants (%)** | **Number of AEs** | | **Number of Participants (%)** | | | **P-value**^†^ |
| **Solicited AE** | (day 168 to day 175) | **55** | **25 (11.36)** | **40** | **18 (16.07)** | **15** | **7 (6.48)** | **15** | | **5 (9.09)** | | |  |
| Relatedness: | Definitely Related | 6 | 4 (1.82) | 4 | 2 (1.79) | 2 | 2 (1.85) | 0 | | 0 (0.00) | | | 0.5872 |
|  | Probably Related | 13 | 7 (3.18) | 6 | 3 (2.68) | 7 | 4 (3.70) | 0 | | 0 (0.00) | | | 0.3391 |
|  | Possibly related | 36 | 19 (8.64) | 30 | 16 (14.29) | 6 | 3 (2.78) | 15 | | 5 (9.09) | | | 0.0107 |
|  | Unlikely related | 0 | 0 (0.00) | 0 | 0 (0.00) | 0 | 0 (0.00) | 0 | | 0 (0.00) | | | - |
|  | Not Related | 0 | 0 (0.00) | 0 | 0 (0.00) | 0 | 0 (0.00) | 0 | | 0 (0.00) | | | - |
| **Entire study period (day 0 to day 196)** | | **Vi-DT Group (N=228)** | |  | |  | | **Comparator Group (N=57)** | | | |  | |
|  |  |  |  | **single dose (N=114)** | | **two-dose (N=114)** | |  |  |  |  |  |  |
|  |  | **Number of AEs** | **Number of Participants (%)** | **Number of AEs** | **Number of Participants (%)** | **Number of AEs** | **Number of Participants (%)** | **Number of AEs** | | **Number of Participants (%)** | | **P-value**^†^ | |
| **Solicited AE** | (within 7 days after each vaccination) | **213** | **77 (33.77)** | **104** | **45 (39.47)** | **109** | **32 (28.07)** | **47** | | **13 (22.81)** | |  | |
| Relatedness: | Definitely Related | 23 | 13 (5.70) | 9 | 5 (4.39) | 14 | 8 (7.02) | 0 | | 0 (0.00) | | 0.1157 | |
|  | Probably Related | 77 | 37 (16.23) | 36 | 19 (16.67) | 41 | 18 (15.79) | 11 | | 5 (8.77) | | 0.3616 | |
|  | Possibly related | 113 | 45 (19.74) | 59 | 29 (25.44) | 54 | 16 (14.04) | 36 | | 12 (21.05) | | 0.0907 | |
|  | Unlikely related | 0 | 0 (0.00) | 0 | 0 (0.00) | 0 | 0 (0.00) | 0 | | 0 (0.00) | | - | |
|  | Not Related | 0 | 0 (0.00) | 0 | 0 (0.00) | 0 | 0 (0.00) | 0 | | 0 (0.00) | | - | |

^†^P-values for all ages have been derived using stratified Chi-square (Cochran-Mantel-Haenszel) test stratified by age. (Vi-DT single vs. two-dose Group vs. Comparator Group). However, P-value may not demonstrate the effect of treatment due to the lack of power.

Table S10 Summary of Unsolicited Adverse Events [All ages] by severity, relatedness

| **Within 4 weeks after first dose** | | **Vi-DT Group (N=228)** | |  | |  | | | **Comparator Group (N=57)** | |  |
| --- | --- | --- | --- | --- | --- | --- | --- | --- | --- | --- | --- |
|  |  |  |  | **single dose**  **(N=114)** | | **two-dose**  **(N=114)** | | |  |  |  |
|  |  | **Number of AEs** | **Number of Participants (%)** | **Number of AEs** | **Number of Participants (%)** | **Number of AEs** | **Number of Participants (%)** | | **Number of AEs** | **Number of Participants (%)** | **P-value**^†^ |
| **Unsolicited AE** | | **211** | **140 (61·40)** | **115** | **74 (64·91)** | **96** | **66 (57·89)** | | **74** | **39 (68·42)** |  |
| Relatedness: | Definitely Related | 1 | 1 (0·44) | 0 | 0 (0·00) | 1 | 1 (0·88) | | 0 | 0 (0·00) | 0.4724 |
|  | Probably Related | 17 | 15 (6·58) | 7 | 6 (5·26) | 10 | 9 (7·89) | | 1 | 1 (1·75) | 0.2537 |
|  | Possibly related | 39 | 37 (16·23) | 20 | 19 (16·67) | 19 | 18 (15·79) | | 12 | 10 (17·54) | 0.9558 |
|  | Unlikely related | 26 | 23 (10·09) | 17 | 14 (12·28) | 9 | 9 (7·89) | | 12 | 10 (17·54) | 0.1703 |
|  | Not Related | 128 | 94 (41·23) | 71 | 48 (42·11) | 57 | 46 (40·35) | | 49 | 31 (54·39) | 0.1877 |
| **Within 4 weeks after second dose** | | **Vi-DT Group (N=220)** | |  | |  | | **Comparator Group (N=55)** | | |  |
|  |  |  |  | **single dose (N=112)** | | **two-dose (N=108)** | |  |  |  |  |
|  |  | **Number of AEs** | **Number of Participants (%)** | **Number of AEs** | **Number of Participants (%)** | **Number of AEs** | **Number of Participants (%)** | **Number of AEs** | | **Number of Participants (%)** | **P-value**^†^ |
| **Unsolicited AE** | | **90** | **72 (32·73)** | **50** | **39 (34·82)** | **40** | **33 (30·56)** | **24** | | **15 (27·27)** |  |
| Relatedness: | Definitely Related | 0 | 0 (0·00) | 0 | 0 (0·00) | 0 | 0 (0·00) | 0 | | 0 (0·00) | - |
|  | Probably Related | 0 | 0 (0·00) | 0 | 0 (0·00) | 0 | 0 (0·00) | 0 | | 0 (0·00) | - |
|  | Possibly related | 12 | 12 (5·45) | 10 | 10 (8·93) | 2 | 2 (1·85) | 4 | | 4 (7·27) | 0.0688 |
|  | Unlikely related | 7 | 6 (2·73) | 2 | 2 (1·79) | 5 | 4 (3·70) | 2 | | 2 (3·64) | 0.6546 |
|  | Not Related | 71 | 58 (26·36) | 38 | 29 (25·89) | 33 | 29 (26·85) | 18 | | 11 (20·00) | 0.6399 |

^†^P-values for all ages have been derived using stratified Chi-square (Cochran-Mantel-Haenszel) test stratified by age. (Vi-DT single vs. two-dose Group vs. Comparator Group). However, P-value may not demonstrate the effect of treatment due to the lack of power

Table S11 Distribution of unsolicited AEs 4 weeks after each vaccination

| **SOC\PT** | **Vi-DT Group**  **(N=228)** | |  |  |  |  | **Comparator Group (N=57)** | |
| --- | --- | --- | --- | --- | --- | --- | --- | --- |
|  |  |  | **Single dose**  **(N=114)** | | **two-dose**  **(N=114)** | |  |  |
| **Within 4 weeks after first dose** | **# of AEs** | **Number of participants (%)** | **# of AEs** | **Number of participants (%)** | **# of AEs** | **Number of participants (%)** | **# of AEs** | **Number of participants (%)** |
| **All ages** | **211** | **140(61.40)** | **115** | **74 (64.91)** | **96** | **66 (57.89)** | **74** | **39 (68.42)** |
| **Gastrointestinal disorders** | 36 | 33 (14.47) | 19 | 17 (14.91) | 17 | 16 (14.04) | 19 | 18 (31.58) |
| Aphthous ulcer | 2 | 2 (0.88) | 1 | 1 (0.88) | 1 | 1 (0.88) | 0 | 0 (0.00) |
| Epigastric discomfort | 1 | 1 (0.44) | 0 | 0 (0.00) | 1 | 1 (0.88) | 0 | 0 (0.00) |
| Gastroenteritis | 32 | 31 (13.60) | 18 | 17 (14.91) | 14 | 14 (12.28) | 17 | 16 (28.07) |
| Toothache | 1 | 1 (0.44) | 0 | 0 (0.00) | 1 | 1 (0.88) | 2 | 2 (3.51) |
| **General disorders and administration site conditions** | 6 | 6 (2.63) | 3 | 3 (2.63) | 3 | 3 (2.63) | 3 | 3 (5.26) |
| Crying | 0 | 0 (0.00) | 0 | 0 (0.00) | 0 | 0 (0.00) | 1 | 1 (1.75) |
| Inflammation | 0 | 0 (0.00) | 0 | 0 (0.00) | 0 | 0 (0.00) | 1 | 1 (1.75) |
| Pyrexia | 6 | 6 (2.63) | 3 | 3 (2.63) | 3 | 3 (2.63) | 1 | 1 (1.75) |
| **Immune system disorders** | 1 | 1 (0.44) | 0 | 0 (0.00) | 1 | 1 (0.88) | 0 | 0 (0.00) |
| Hypersensitivity | 1 | 1 (0.44) | 0 | 0 (0.00) | 1 | 1 (0.88) | 0 | 0 (0.00) |
| **Infections and infestations** | 111 | 92 (40.35) | 61 | 51 (44.74) | 50 | 41 (35.96) | 34 | 26 (45.61) |
| Carbuncle | 2 | 1 (0.44) | 2 | 1 (0.88) | 0 | 0 (0.00) | 2 | 2 (3.51) |
| Conjunctivitis viral | 3 | 3 (1.32) | 2 | 2 (1.75) | 1 | 1 (0.88) | 0 | 0 (0.00) |
| Exanthema subitum | 1 | 1 (0.44) | 1 | 1 (0.88) | 0 | 0 (0.00) | 0 | 0 (0.00) |
| Herpangina | 2 | 2 (0.88) | 1 | 1 (0.88) | 1 | 1 (0.88) | 0 | 0 (0.00) |
| Impetigo | 4 | 4 (1.75) | 1 | 1 (0.88) | 3 | 3 (2.63) | 1 | 1 (1.75) |
| Nasopharyngitis | 1 | 1 (0.44) | 0 | 0 (0.00) | 1 | 1 (0.88) | 0 | 0 (0.00) |
| Otitis media | 1 | 1 (0.44) | 1 | 1 (0.88) | 0 | 0 (0.00) | 0 | 0 (0.00) |
| Otitis media acute | 1 | 1 (0.44) | 0 | 0 (0.00) | 1 | 1 (0.88) | 0 | 0 (0.00) |
| Perichondritis | 0 | 0 (0.00) | 0 | 0 (0.00) | 0 | 0 (0.00) | 1 | 1 (1.75) |
| Pneumonia | 2 | 2 (0.88) | 2 | 2 (1.75) | 0 | 0 (0.00) | 3 | 3 (5.26) |
| Rhinitis | 18 | 18 (7.89) | 11 | 11 (9.65) | 7 | 7 (6.14) | 5 | 5 (8.77) |
| Upper respiratory tract infection | 66 | 60 (26.32) | 34 | 32 (28.07) | 32 | 28 (24.56) | 15 | 13 (22.81) |
| Urinary tract infection | 4 | 4 (1.75) | 2 | 2 (1.75) | 2 | 2 (1.75) | 2 | 2 (3.51) |
| Varicella | 0 | 0 (0.00) | 0 | 0 (0.00) | 0 | 0 (0.00) | 1 | 1 (1.75) |
| Viral infection | 4 | 4 (1.75) | 2 | 2 (1.75) | 2 | 2 (1.75) | 4 | 4 (7.02) |
| Viral rash | 2 | 2 (0.88) | 2 | 2 (1.75) | 0 | 0 (0.00) | 0 | 0 (0.00) |
| **Injury, poisoning and procedural complications** | 6 | 6 (2.63) | 5 | 5 (4.39) | 1 | 1 (0.88) | 1 | 1 (1.75) |
| Animal bite | 2 | 2 (0.88) | 2 | 2 (1.75) | 0 | 0 (0.00) | 0 | 0 (0.00) |
| Animal scratch | 1 | 1 (0.44) | 1 | 1 (0.88) | 0 | 0 (0.00) | 0 | 0 (0.00) |
| Joint injury | 1 | 1 (0.44) | 1 | 1 (0.88) | 0 | 0 (0.00) | 1 | 1 (1.75) |
| Traumatic haematoma | 1 | 1 (0.44) | 1 | 1 (0.88) | 0 | 0 (0.00) | 0 | 0 (0.00) |
| Wound | 1 | 1 (0.44) | 0 | 0 (0.00) | 1 | 1 (0.88) | 0 | 0 (0.00) |
| **Metabolism and nutrition disorders** | 0 | 0 (0.00) | 0 | 0 (0.00) | 0 | 0 (0.00) | 1 | 1 (1.75) |
| Food intolerance | 0 | 0 (0.00) | 0 | 0 (0.00) | 0 | 0 (0.00) | 1 | 1 (1.75) |
| **Nervous system disorders** | 1 | 1 (0.44) | 1 | 1 (0.88) | 0 | 0 (0.00) | 0 | 0 (0.00) |
| Febrile convulsion | 1 | 1 (0.44) | 1 | 1 (0.88) | 0 | 0 (0.00) | 0 | 0 (0.00) |
| **Psychiatric disorders** | 0 | 0 (0.00) | 0 | 0 (0.00) | 0 | 0 (0.00) | 1 | 1 (1.75) |
| Irritability | 0 | 0 (0.00) | 0 | 0 (0.00) | 0 | 0 (0.00) | 1 | 1 (1.75) |
| **Respiratory, thoracic and mediastinal disorders** | 36 | 35 (15.35) | 21 | 20 (17.54) | 15 | 15 (13.16) | 11 | 11 (19.30) |
| Bronchiolitis | 0 | 0 (0.00) | 0 | 0 (0.00) | 0 | 0 (0.00) | 1 | 1 (1.75) |
| Bronchitis | 17 | 17 (7.46) | 11 | 11 (9.65) | 6 | 6 (5.26) | 4 | 4 (7.02) |
| Cough | 1 | 1 (0.44) | 1 | 1 (0.88) | 0 | 0 (0.00) | 0 | 0 (0.00) |
| Nasopharyngitis | 17 | 17 (7.46) | 9 | 9 (7.89) | 8 | 8 (7.02) | 5 | 5 (8.77) |
| Pharyngotonsillitis | 1 | 1 (0.44) | 0 | 0 (0.00) | 1 | 1 (0.88) | 0 | 0 (0.00) |
| Upper respiratory tract infection | 0 | 0 (0.00) | 0 | 0 (0.00) | 0 | 0 (0.00) | 1 | 1 (1.75) |
| **Skin and subcutaneous tissue disorders** | 14 | 14 (6.14) | 5 | 5 (4.39) | 9 | 9 (7.89) | 4 | 4 (7.02) |
| Dermatitis | 0 | 0 (0.00) | 0 | 0 (0.00) | 0 | 0 (0.00) | 1 | 1 (1.75) |
| Dermatitis contact | 1 | 1 (0.44) | 0 | 0 (0.00) | 1 | 1 (0.88) | 1 | 1 (1.75) |
| Dermatitis diaper | 4 | 4 (1.75) | 2 | 2 (1.75) | 2 | 2 (1.75) | 1 | 1 (1.75) |
| Dermatitis infected | 1 | 1 (0.44) | 1 | 1 (0.88) | 0 | 0 (0.00) | 0 | 0 (0.00) |
| Folliculitis | 5 | 5 (2.19) | 0 | 0 (0.00) | 5 | 5 (4.39) | 0 | 0 (0.00) |
| Miliaria | 3 | 3 (1.32) | 2 | 2 (1.75) | 1 | 1 (0.88) | 1 | 1 (1.75) |
| **Age Strata1** | **68** | **44 (57.89)** | **33** | **23 (60.53)** | **35** | **21 (55.26)** | **29** | **16 (84.21)** |
| **Gastrointestinal disorders** | 11 | 10 (13.16) | 5 | 5 (13.16) | 6 | 5 (13.16) | 8 | 8 (42.11) |
| Epigastric discomfort | 1 | 1 (1.32) | 0 | 0 (0.00) | 1 | 1 (2.63) | 0 | 0 (0.00) |
| Gastroenteritis | 10 | 10 (13.16) | 5 | 5 (13.16) | 5 | 5 (13.16) | 8 | 8 (42.11) |
| **General disorders and** administration **site conditions** | 2 | 2 (2.63) | 0 | 0 (0.00) | 2 | 2 (5.26) | 2 | 2 (10.53) |
| Crying | 0 | 0 (0.00) | 0 | 0 (0.00) | 0 | 0 (0.00) | 1 | 1 (5.26) |
| Inflammation | 0 | 0 (0.00) | 0 | 0 (0.00) | 0 | 0 (0.00) | 1 | 1 (5.26) |
| Pyrexia | 2 | 2 (2.63) | 0 | 0 (0.00) | 2 | 2 (5.26) | 0 | 0 (0.00) |
| **Infections and infestations** | 39 | 33 (43.42) | 20 | 18 (47.37) | 19 | 15 (39.47) | 12 | 10 (52.63) |
| Conjunctivitis viral | 1 | 1 (1.32) | 1 | 1 (2.63) | 0 | 0 (0.00) | 0 | 0 (0.00) |
| Impetigo | 1 | 1 (1.32) | 0 | 0 (0.00) | 1 | 1 (2.63) | 0 | 0 (0.00) |
| Otitis media acute | 1 | 1 (1.32) | 0 | 0 (0.00) | 1 | 1 (2.63) | 0 | 0 (0.00) |
| Perichondritis | 0 | 0 (0.00) | 0 | 0 (0.00) | 0 | 0 (0.00) | 1 | 1 (5.26) |
| Pneumonia | 1 | 1 (1.32) | 1 | 1 (2.63) | 0 | 0 (0.00) | 0 | 0 (0.00) |
| Rhinitis | 8 | 8 (10.53) | 3 | 3 (7.89) | 5 | 5 (13.16) | 2 | 2 (10.53) |
| Upper respiratory tract infection | 23 | 20 (26.32) | 13 | 12 (31.58) | 10 | 8 (21.05) | 7 | 6 (31.58) |
| Urinary tract infection | 2 | 2 (2.63) | 1 | 1 (2.63) | 1 | 1 (2.63) | 1 | 1 (5.26) |
| Viral infection | 1 | 1 (1.32) | 0 | 0 (0.00) | 1 | 1 (2.63) | 1 | 1 (5.26) |
| Viral rash | 1 | 1 (1.32) | 1 | 1 (2.63) | 0 | 0 (0.00) | 0 | 0 (0.00) |
| **Nervous system disorders** | 1 | 1 (1.32) | 1 | 1 (2.63) | 0 | 0 (0.00) | 0 | 0 (0.00) |
| Febrile convulsion | 1 | 1 (1.32) | 1 | 1 (2.63) | 0 | 0 (0.00) | 0 | 0 (0.00) |
| **Psychiatric disorders** | 0 | 0 (0.00) | 0 | 0 (0.00) | 0 | 0 (0.00) | 1 | 1 (5.26) |
| Irritability | 0 | 0 (0.00) | 0 | 0 (0.00) | 0 | 0 (0.00) | 1 | 1 (5.26) |
| **Respiratory, thoracic and mediastinal disorders** | 13 | 13 (17.11) | 7 | 7 (18.42) | 6 | 6 (15.79) | 4 | 4 (21.05) |
| Bronchitis | 7 | 7 (9.21) | 4 | 4 (10.53) | 3 | 3 (7.89) | 1 | 1 (5.26) |
| Nasopharyngitis | 6 | 6 (7.89) | 3 | 3 (7.89) | 3 | 3 (7.89) | 3 | 3 (15.79) |
| **Skin and subcutaneous tissue disorders** | 2 | 2 (2.63) | 0 | 0 (0.00) | 2 | 2 (5.26) | 2 | 2 (10.53) |
| Dermatitis | 0 | 0 (0.00) | 0 | 0 (0.00) | 0 | 0 (0.00) | 1 | 1 (5.26) |
| Dermatitis diaper | 2 | 2 (2.63) | 0 | 0 (0.00) | 2 | 2 (5.26) | 1 | 1 (5.26) |
| **Age Strata2** | **77** | **50 (65.79)** | **42** | **25 (65.79)** | **35** | **25 (65.79)** | **24** | **12 (63.16)** |
| **Gastrointestinal disorders** | 13 | 11 (14.47) | 8 | 6 (15.79) | 5 | 5 (13.16) | 5 | 4 (21.05) |
| Aphthous ulcer | 1 | 1 (1.32) | 1 | 1 (2.63) | 0 | 0 (0.00) | 0 | 0 (0.00) |
| Gastroenteritis | 11 | 10 (13.16) | 7 | 6 (15.79) | 4 | 4 (10.53) | 4 | 3 (15.79) |
| Toothache | 1 | 1 (1.32) | 0 | 0 (0.00) | 1 | 1 (2.63) | 1 | 1 (5.26) |
| **General disorders and** administration **site conditions** | 4 | 4 (5.26) | 3 | 3 (7.89) | 1 | 1 (2.63) | 0 | 0 (0.00) |
| Pyrexia | 4 | 4 (5.26) | 3 | 3 (7.89) | 1 | 1 (2.63) | 0 | 0 (0.00) |
| **Immune system disorders** | 1 | 1 (1.32) | 0 | 0 (0.00) | 1 | 1 (2.63) | 0 | 0 (0.00) |
| Hypersensitivity | 1 | 1 (1.32) | 0 | 0 (0.00) | 1 | 1 (2.63) | 0 | 0 (0.00) |
| **Infections and infestations** | 39 | 31 (40.79) | 20 | 16 (42.11) | 19 | 15 (39.47) | 11 | 8 (42.11) |
| Carbuncle | 2 | 1 (1.32) | 2 | 1 (2.63) | 0 | 0 (0.00) | 1 | 1 (5.26) |
| Exanthema subitum | 1 | 1 (1.32) | 1 | 1 (2.63) | 0 | 0 (0.00) | 0 | 0 (0.00) |
| Impetigo | 1 | 1 (1.32) | 0 | 0 (0.00) | 1 | 1 (2.63) | 0 | 0 (0.00) |
| Pneumonia | 1 | 1 (1.32) | 1 | 1 (2.63) | 0 | 0 (0.00) | 2 | 2 (10.53) |
| Rhinitis | 3 | 3 (3.95) | 3 | 3 (7.89) | 0 | 0 (0.00) | 1 | 1 (5.26) |
| Upper respiratory tract infection | 27 | 25 (32.89) | 11 | 11 (28.95) | 16 | 14 (36.84) | 6 | 6 (31.58) |
| Urinary tract infection | 2 | 2 (2.63) | 1 | 1 (2.63) | 1 | 1 (2.63) | 1 | 1 (5.26) |
| Viral infection | 2 | 2 (2.63) | 1 | 1 (2.63) | 1 | 1 (2.63) | 0 | 0 (0.00) |
| **Injury, poisoning and procedural complications** | 1 | 1 (1.32) | 1 | 1 (2.63) | 0 | 0 (0.00) | 0 | 0 (0.00) |
| Traumatic haematoma | 1 | 1 (1.32) | 1 | 1 (2.63) | 0 | 0 (0.00) | 0 | 0 (0.00) |
| **Metabolism and nutrition disorders** | 0 | 0 (0.00) | 0 | 0 (0.00) | 0 | 0 (0.00) | 1 | 1 (5.26) |
| Food intolerance | 0 | 0 (0.00) | 0 | 0 (0.00) | 0 | 0 (0.00) | 1 | 1 (5.26) |
| **Respiratory, thoracic and mediastinal disorders** | 12 | 12 (15.79) | 8 | 8 (21.05) | 4 | 4 (10.53) | 6 | 6 (31.58) |
| Bronchiolitis | 0 | 0 (0.00) | 0 | 0 (0.00) | 0 | 0 (0.00) | 1 | 1 (5.26) |
| Bronchitis | 4 | 4 (5.26) | 4 | 4 (10.53) | 0 | 0 (0.00) | 3 | 3 (15.79) |
| Nasopharyngitis | 8 | 8 (10.53) | 4 | 4 (10.53) | 4 | 4 (10.53) | 2 | 2 (10.53) |
| **Skin and subcutaneous tissue disorders** | 7 | 7 (9.21) | 2 | 2 (5.26) | 5 | 5 (13.16) | 1 | 1 (5.26) |
| Dermatitis contact | 1 | 1 (1.32) | 0 | 0 (0.00) | 1 | 1 (2.63) | 1 | 1 (5.26) |
| Dermatitis diaper | 2 | 2 (2.63) | 2 | 2 (5.26) | 0 | 0 (0.00) | 0 | 0 (0.00) |
| Folliculitis | 3 | 3 (3.95) | 0 | 0 (0.00) | 3 | 3 (7.89) | 0 | 0 (0.00) |
| Miliaria | 1 | 1 (1.32) | 0 | 0 (0.00) | 1 | 1 (2.63) | 0 | 0 (0.00) |
| **Age Strata3** | **66** | **46 (60.53)** | **40** | **26 (68.42)** | **26** | **20 (52.63)** | **21** | **11 (57.89)** |
| **Gastrointestinal disorders** | 12 | 12 (15.79) | 6 | 6 (15.79) | 6 | 6 (15.79) | 6 | 6 (31.58) |
| Aphthous ulcer | 1 | 1 (1.32) | 0 | 0 (0.00) | 1 | 1 (2.63) | 0 | 0 (0.00) |
| Gastroenteritis | 11 | 11 (14.47) | 6 | 6 (15.79) | 5 | 5 (13.16) | 5 | 5 (26.32) |
| Toothache | 0 | 0 (0.00) | 0 | 0 (0.00) | 0 | 0 (0.00) | 1 | 1 (5.26) |
| **General disorders and administration site conditions** | 0 | 0 (0.00) | 0 | 0 (0.00) | 0 | 0 (0.00) | 1 | 1 (5.26) |
| Pyrexia | 0 | 0 (0.00) | 0 | 0 (0.00) | 0 | 0 (0.00) | 1 | 1 (5.26) |
| **Infections and infestations** | 33 | 28 (36.84) | 21 | 17 (44.74) | 12 | 11 (28.95) | 11 | 8 (42.11) |
| Carbuncle | 0 | 0 (0.00) | 0 | 0 (0.00) | 0 | 0 (0.00) | 1 | 1 (5.26) |
| Conjunctivitis viral | 2 | 2 (2.63) | 1 | 1 (2.63) | 1 | 1 (2.63) | 0 | 0 (0.00) |
| Herpangina | 2 | 2 (2.63) | 1 | 1 (2.63) | 1 | 1 (2.63) | 0 | 0 (0.00) |
| Impetigo | 2 | 2 (2.63) | 1 | 1 (2.63) | 1 | 1 (2.63) | 1 | 1 (5.26) |
| Nasopharyngitis | 1 | 1 (1.32) | 0 | 0 (0.00) | 1 | 1 (2.63) | 0 | 0 (0.00) |
| Otitis media | 1 | 1 (1.32) | 1 | 1 (2.63) | 0 | 0 (0.00) | 0 | 0 (0.00) |
| Pneumonia | 0 | 0 (0.00) | 0 | 0 (0.00) | 0 | 0 (0.00) | 1 | 1 (5.26) |
| Rhinitis | 7 | 7 (9.21) | 5 | 5 (13.16) | 2 | 2 (5.26) | 2 | 2 (10.53) |
| Upper respiratory tract infection | 16 | 15 (19.74) | 10 | 9 (23.68) | 6 | 6 (15.79) | 2 | 1 (5.26) |
| Varicella | 0 | 0 (0.00) | 0 | 0 (0.00) | 0 | 0 (0.00) | 1 | 1 (5.26) |
| Viral infection | 1 | 1 (1.32) | 1 | 1 (2.63) | 0 | 0 (0.00) | 3 | 3 (15.79) |
| Viral rash | 1 | 1 (1.32) | 1 | 1 (2.63) | 0 | 0 (0.00) | 0 | 0 (0.00) |
| **Injury, poisoning and procedural complications** | 5 | 5 (6.58) | 4 | 4 (10.53) | 1 | 1 (2.63) | 1 | 1 (5.26) |
| Animal bite | 2 | 2 (2.63) | 2 | 2 (5.26) | 0 | 0 (0.00) | 0 | 0 (0.00) |
| Animal scratch | 1 | 1 (1.32) | 1 | 1 (2.63) | 0 | 0 (0.00) | 0 | 0 (0.00) |
| Joint injury | 1 | 1 (1.32) | 1 | 1 (2.63) | 0 | 0 (0.00) | 1 | 1 (5.26) |
| Wound | 1 | 1 (1.32) | 0 | 0 (0.00) | 1 | 1 (2.63) | 0 | 0 (0.00) |
| **Respiratory, thoracic and mediastinal disorders** | 11 | 10 (13.16) | 6 | 5 (13.16) | 5 | 5 (13.16) | 1 | 1 (5.26) |
| Bronchitis | 6 | 6 (7.89) | 3 | 3 (7.89) | 3 | 3 (7.89) | 0 | 0 (0.00) |
| Cough | 1 | 1 (1.32) | 1 | 1 (2.63) | 0 | 0 (0.00) | 0 | 0 (0.00) |
| Nasopharyngitis | 3 | 3 (3.95) | 2 | 2 (5.26) | 1 | 1 (2.63) | 0 | 0 (0.00) |
| Pharyngotonsillitis | 1 | 1 (1.32) | 0 | 0 (0.00) | 1 | 1 (2.63) | 0 | 0 (0.00) |
| Upper respiratory tract infection | 0 | 0 (0.00) | 0 | 0 (0.00) | 0 | 0 (0.00) | 1 | 1 (5.26) |
| **Skin and subcutaneous tissue disorders** | 5 | 5 (6.58) | 3 | 3 (7.89) | 2 | 2 (5.26) | 1 | 1 (5.26) |
| Dermatitis infected | 1 | 1 (1.32) | 1 | 1 (2.63) | 0 | 0 (0.00) | 0 | 0 (0.00) |
| Folliculitis | 2 | 2 (2.63) | 0 | 0 (0.00) | 2 | 2 (5.26) | 0 | 0 (0.00) |
| Miliaria | 2 | 2 (2.63) | 2 | 2 (5.26) | 0 | 0 (0.00) | 1 | 1 (5.26) |
|  | **Vi-DT Group**  **(N=220)** | |  |  |  |  | **Comparator Group (N=55)** | |
|  |  |  | **Single dose**  **(N=112)** | | **two-dose**  **(N=108)** | |  |  |
| **Within 4 weeks after second dose** | **# of AEs** | **Number of participants (%)** | **# of AEs** | **Number of participants (%)** | **# of AEs** | **Number of participants (%)** | **# of AEs** | **Number of participants (%)** |
| **All ages** | **90** | **72 (32.73)** | **50** | **39 (34.82)** | **40** | **33 (30.56)** | **24** | **15 (27.27)** |
| **Gastrointestinal disorders** | 12 | 12 (5.45) | 6 | 6 (5.36) | 6 | 6 (5.56) | 4 | 4 (7.27) |
| Gastroenteritis | 10 | 10 (4.55) | 5 | 5 (4.46) | 5 | 5 (4.63) | 3 | 3 (5.45) |
| Stomatitis | 2 | 2 (0.91) | 1 | 1 (0.89) | 1 | 1 (0.93) | 1 | 1 (1.82) |
| **General disorders and administration site conditions** | 2 | 2 (0.91) | 2 | 2 (1.79) | 0 | 0 (0.00) | 0 | 0 (0.00) |
| Pyrexia | 2 | 2 (0.91) | 2 | 2 (1.79) | 0 | 0 (0.00) | 0 | 0 (0.00) |
| **Infections and infestations** | 48 | 43 (19.55) | 25 | 22 (19.64) | 23 | 21 (19.44) | 14 | 10 (18.18) |
| Amoebiasis | 1 | 1 (0.45) | 1 | 1 (0.89) | 0 | 0 (0.00) | 0 | 0 (0.00) |
| Carbuncle | 0 | 0 (0.00) | 0 | 0 (0.00) | 0 | 0 (0.00) | 1 | 1 (1.82) |
| Exanthema subitum | 1 | 1 (0.45) | 1 | 1 (0.89) | 0 | 0 (0.00) | 0 | 0 (0.00) |
| Impetigo | 0 | 0 (0.00) | 0 | 0 (0.00) | 0 | 0 (0.00) | 1 | 1 (1.82) |
| Nasopharyngitis | 1 | 1 (0.45) | 1 | 1 (0.89) | 0 | 0 (0.00) | 0 | 0 (0.00) |
| Otitis media | 0 | 0 (0.00) | 0 | 0 (0.00) | 0 | 0 (0.00) | 1 | 1 (1.82) |
| Otitis media acute | 2 | 2 (0.91) | 0 | 0 (0.00) | 2 | 2 (1.85) | 0 | 0 (0.00) |
| Parasitic gastroenteritis | 2 | 2 (0.91) | 2 | 2 (1.79) | 0 | 0 (0.00) | 0 | 0 (0.00) |
| Pneumonia | 4 | 4 (1.82) | 1 | 1 (0.89) | 3 | 3 (2.78) | 0 | 0 (0.00) |
| Rhinitis | 1 | 1 (0.45) | 0 | 0 (0.00) | 1 | 1 (0.93) | 1 | 1 (1.82) |
| Upper respiratory tract infection | 28 | 27 (12.27) | 15 | 14 (12.50) | 13 | 13 (12.04) | 7 | 6 (10.91) |
| Urinary tract infection | 5 | 5 (2.27) | 3 | 3 (2.68) | 2 | 2 (1.85) | 1 | 1 (1.82) |
| Viral infection | 3 | 3 (1.36) | 1 | 1 (0.89) | 2 | 2 (1.85) | 2 | 2 (3.64) |
| **Nervous system disorders** | 0 | 0 (0.00) | 0 | 0 (0.00) | 0 | 0 (0.00) | 1 | 1 (1.82) |
| Febrile convulsion | 0 | 0 (0.00) | 0 | 0 (0.00) | 0 | 0 (0.00) | 1 | 1 (1.82) |
| **Respiratory, thoracic and mediastinal disorders** | 25 | 25 (11.36) | 15 | 15 (13.39) | 10 | 10 (9.26) | 4 | 4 (7.27) |
| Bronchitis | 16 | 16 (7.27) | 9 | 9 (8.04) | 7 | 7 (6.48) | 2 | 2 (3.64) |
| Nasopharyngitis | 8 | 8 (3.64) | 5 | 5 (4.46) | 3 | 3 (2.78) | 2 | 2 (3.64) |
| Pharyngotonsillitis | 1 | 1 (0.45) | 1 | 1 (0.89) | 0 | 0 (0.00) | 0 | 0 (0.00) |
| **Skin and subcutaneous tissue disorders** | 3 | 3 (1.36) | 2 | 2 (1.79) | 1 | 1 (0.93) | 1 | 1 (1.82) |
| Dermatitis diaper | 0 | 0 (0.00) | 0 | 0 (0.00) | 0 | 0 (0.00) | 1 | 1 (1.82) |
| Folliculitis | 1 | 1 (0.45) | 1 | 1 (0.89) | 0 | 0 (0.00) | 0 | 0 (0.00) |
| Intertrigo | 1 | 1 (0.45) | 1 | 1 (0.89) | 0 | 0 (0.00) | 0 | 0 (0.00) |
| Miliaria | 1 | 1 (0.45) | 0 | 0 (0.00) | 1 | 1 (0.93) | 0 | 0 (0.00) |
| **Age Strata1** | **31** | **26 (34.67)** | **13** | **11 (28.95)** | **18** | **15 (40.54)** | **9** | **6 (35.29)** |
| **Gastrointestinal disorders** | 4 | 4 (5.33) | 1 | 1 (2.63) | 3 | 3 (8.11) | 2 | 2 (11.76) |
| Gastroenteritis | 4 | 4 (5.33) | 1 | 1 (2.63) | 3 | 3 (8.11) | 1 | 1 (5.88) |
| Stomatitis | 0 | 0 (0.00) | 0 | 0 (0.00) | 0 | 0 (0.00) | 1 | 1 (5.88) |
| **Infections and infestations** | 17 | 16 (21.33) | 7 | 7 (18.42) | 10 | 9 (24.32) | 4 | 3 (17.65) |
| Exanthema subitum | 1 | 1 (1.33) | 1 | 1 (2.63) | 0 | 0 (0.00) | 0 | 0 (0.00) |
| Parasitic gastroenteritis | 1 | 1 (1.33) | 1 | 1 (2.63) | 0 | 0 (0.00) | 0 | 0 (0.00) |
| Pneumonia | 2 | 2 (2.67) | 0 | 0 (0.00) | 2 | 2 (5.41) | 0 | 0 (0.00) |
| Rhinitis | 0 | 0 (0.00) | 0 | 0 (0.00) | 0 | 0 (0.00) | 1 | 1 (5.88) |
| Upper respiratory tract infection | 12 | 12 (16.00) | 5 | 5 (13.16) | 7 | 7 (18.92) | 1 | 1 (5.88) |
| Urinary tract infection | 1 | 1 (1.33) | 0 | 0 (0.00) | 1 | 1 (2.70) | 1 | 1 (5.88) |
| Viral infection | 0 | 0 (0.00) | 0 | 0 (0.00) | 0 | 0 (0.00) | 1 | 1 (5.88) |
| **Respiratory, thoracic and mediastinal disorders** | 10 | 10 (13.33) | 5 | 5 (13.16) | 5 | 5 (13.51) | 2 | 2 (11.76) |
| Bronchitis | 8 | 8 (10.67) | 4 | 4 (10.53) | 4 | 4 (10.81) | 2 | 2 (11.76) |
| Nasopharyngitis | 2 | 2 (2.67) | 1 | 1 (2.63) | 1 | 1 (2.70) | 0 | 0 (0.00) |
| **Skin and subcutaneous tissue disorders** | 0 | 0 (0.00) | 0 | 0 (0.00) | 0 | 0 (0.00) | 1 | 1 (5.88) |
| Dermatitis diaper | 0 | 0 (0.00) | 0 | 0 (0.00) | 0 | 0 (0.00) | 1 | 1 (5.88) |
| **Age Strata2** | **21** | **16 (23.19)** | **11** | **9 (25.00)** | **10** | **7 (21.21)** | **5** | **3 (15.79)** |
| **Gastrointestinal disorders** | 3 | 3 (4.35) | 1 | 1 (2.78) | 2 | 2 (6.06) | 1 | 1 (5.26) |
| Gastroenteritis | 2 | 2 (2.90) | 1 | 1 (2.78) | 1 | 1 (3.03) | 1 | 1 (5.26) |
| Stomatitis | 1 | 1 (1.45) | 0 | 0 (0.00) | 1 | 1 (3.03) | 0 | 0 (0.00) |
| **General disorders and administration site conditions** | 1 | 1 (1.45) | 1 | 1 (2.78) | 0 | 0 (0.00) | 0 | 0 (0.00) |
| Pyrexia | 1 | 1 (1.45) | 1 | 1 (2.78) | 0 | 0 (0.00) | 0 | 0 (0.00) |
| **Infections and infestations** | 11 | 10 (14.49) | 6 | 5 (13.89) | 5 | 5 (15.15) | 2 | 1 (5.26) |
| Amoebiasis | 1 | 1 (1.45) | 1 | 1 (2.78) | 0 | 0 (0.00) | 0 | 0 (0.00) |
| Carbuncle | 0 | 0 (0.00) | 0 | 0 (0.00) | 0 | 0 (0.00) | 1 | 1 (5.26) |
| Nasopharyngitis | 1 | 1 (1.45) | 1 | 1 (2.78) | 0 | 0 (0.00) | 0 | 0 (0.00) |
| Otitis media acute | 1 | 1 (1.45) | 0 | 0 (0.00) | 1 | 1 (3.03) | 0 | 0 (0.00) |
| Pneumonia | 2 | 2 (2.90) | 1 | 1 (2.78) | 1 | 1 (3.03) | 0 | 0 (0.00) |
| Upper respiratory tract infection | 3 | 2 (2.90) | 2 | 1 (2.78) | 1 | 1 (3.03) | 1 | 1 (5.26) |
| Urinary tract infection | 2 | 2 (2.90) | 1 | 1 (2.78) | 1 | 1 (3.03) | 0 | 0 (0.00) |
| Viral infection | 1 | 1 (1.45) | 0 | 0 (0.00) | 1 | 1 (3.03) | 0 | 0 (0.00) |
| **Nervous system disorders** | 0 | 0 (0.00) | 0 | 0 (0.00) | 0 | 0 (0.00) | 1 | 1 (5.26) |
| Febrile convulsion | 0 | 0 (0.00) | 0 | 0 (0.00) | 0 | 0 (0.00) | 1 | 1 (5.26) |
| **Respiratory, thoracic and mediastinal disorders** | 5 | 5 (7.25) | 3 | 3 (8.33) | 2 | 2 (6.06) | 1 | 1 (5.26) |
| Bronchitis | 2 | 2 (2.90) | 1 | 1 (2.78) | 1 | 1 (3.03) | 0 | 0 (0.00) |
| Nasopharyngitis | 3 | 3 (4.35) | 2 | 2 (5.56) | 1 | 1 (3.03) | 1 | 1 (5.26) |
| **Skin and subcutaneous tissue disorders** | 1 | 1 (1.45) | 0 | 0 (0.00) | 1 | 1 (3.03) | 0 | 0 (0.00) |
| Miliaria | 1 | 1 (1.45) | 0 | 0 (0.00) | 1 | 1 (3.03) | 0 | 0 (0.00) |
| **Age Strata3** | **38** | **30 (39.47)** | **26** | **19 (50.00)** | **12** | **11 (28.95)** | **10** | **6 (31.58)** |
| **Gastrointestinal disorders** | 5 | 5 (6.58) | 4 | 4 (10.53) | 1 | 1 (2.63) | 1 | 1 (5.26) |
| Gastroenteritis | 4 | 4 (5.26) | 3 | 3 (7.89) | 1 | 1 (2.63) | 1 | 1 (5.26) |
| Stomatitis | 1 | 1 (1.32) | 1 | 1 (2.63) | 0 | 0 (0.00) | 0 | 0 (0.00) |
| **General disorders and administration site conditions** | 1 | 1 (1.32) | 1 | 1 (2.63) | 0 | 0 (0.00) | 0 | 0 (0.00) |
| Pyrexia | 1 | 1 (1.32) | 1 | 1 (2.63) | 0 | 0 (0.00) | 0 | 0 (0.00) |
| **Infections and infestations** | 20 | 17 (22.37) | 12 | 10 (26.32) | 8 | 7 (18.42) | 8 | 6 (31.58) |
| Impetigo | 0 | 0 (0.00) | 0 | 0 (0.00) | 0 | 0 (0.00) | 1 | 1 (5.26) |
| Otitis media | 0 | 0 (0.00) | 0 | 0 (0.00) | 0 | 0 (0.00) | 1 | 1 (5.26) |
| Otitis media acute | 1 | 1 (1.32) | 0 | 0 (0.00) | 1 | 1 (2.63) | 0 | 0 (0.00) |
| Parasitic gastroenteritis | 1 | 1 (1.32) | 1 | 1 (2.63) | 0 | 0 (0.00) | 0 | 0 (0.00) |
| Rhinitis | 1 | 1 (1.32) | 0 | 0 (0.00) | 1 | 1 (2.63) | 0 | 0 (0.00) |
| Upper respiratory tract infection | 13 | 13 (17.11) | 8 | 8 (21.05) | 5 | 5 (13.16) | 5 | 4 (21.05) |
| Urinary tract infection | 2 | 2 (2.63) | 2 | 2 (5.26) | 0 | 0 (0.00) | 0 | 0 (0.00) |
| Viral infection | 2 | 2 (2.63) | 1 | 1 (2.63) | 1 | 1 (2.63) | 1 | 1 (5.26) |
| **Respiratory, thoracic and mediastinal disorders** | 10 | 10 (13.16) | 7 | 7 (18.42) | 3 | 3 (7.89) | 1 | 1 (5.26) |
| Bronchitis | 6 | 6 (7.89) | 4 | 4 (10.53) | 2 | 2 (5.26) | 0 | 0 (0.00) |
| Nasopharyngitis | 3 | 3 (3.95) | 2 | 2 (5.26) | 1 | 1 (2.63) | 1 | 1 (5.26) |
| Pharyngotonsillitis | 1 | 1 (1.32) | 1 | 1 (2.63) | 0 | 0 (0.00) | 0 | 0 (0.00) |
| **Skin and subcutaneous tissue disorders** | 2 | 2 (2.63) | 2 | 2 (5.26) | 0 | 0 (0.00) | 0 | 0 (0.00) |
| Folliculitis | 1 | 1 (1.32) | 1 | 1 (2.63) | 0 | 0 (0.00) | 0 | 0 (0.00) |
| Intertrigo | 1 | 1 (1.32) | 1 | 1 (2.63) | 0 | 0 (0.00) | 0 | 0 (0.00) |
|  | **Vi-DT Group**  **(N=228)** | |  |  |  |  | **Comparator Group (N=57)** | |
|  |  |  | **Single dose**  **(N=114)** | | **two-dose**  **(N=114)** | |  |  |
| **Within 4 weeks after any dose** | **# of AEs** | **Number of participants (%)** | **# of AEs** | **Number of participants (%)** | **# of AEs** | **Number of participants (%)** | **# of AEs** | **Number of participants (%)** |
| **All ages** | **301** | **162(71.05)** | **165** | **85 (74.56)** | **136** | **77 (67.54)** | **98** | **42 (73.68)** |
| **Gastrointestinal disorders** | 48 | 43 (18.86) | 25 | 22 (19.30) | 23 | 21 (18.42) | 23 | 19 (33.33) |
| Aphthous ulcer | 2 | 2 (0.88) | 1 | 1 (0.88) | 1 | 1 (0.88) | 0 | 0 (0.00) |
| Epigastric discomfort | 1 | 1 (0.44) | 0 | 0 (0.00) | 1 | 1 (0.88) | 0 | 0 (0.00) |
| Gastroenteritis | 42 | 39 (17.11) | 23 | 21 (18.42) | 19 | 18 (15.79) | 20 | 17 (29.82) |
| Stomatitis | 2 | 2 (0.88) | 1 | 1 (0.88) | 1 | 1 (0.88) | 1 | 1 (1.75) |
| Toothache | 1 | 1 (0.44) | 0 | 0 (0.00) | 1 | 1 (0.88) | 2 | 2 (3.51) |
| **General disorders and administration site conditions** | 8 | 8 (3.51) | 5 | 5 (4.39) | 3 | 3 (2.63) | 3 | 3 (5.26) |
| Crying | 0 | 0 (0.00) | 0 | 0 (0.00) | 0 | 0 (0.00) | 1 | 1 (1.75) |
| Inflammation | 0 | 0 (0.00) | 0 | 0 (0.00) | 0 | 0 (0.00) | 1 | 1 (1.75) |
| Pyrexia | 8 | 8 (3.51) | 5 | 5 (4.39) | 3 | 3 (2.63) | 1 | 1 (1.75) |
| **Immune system disorders** | 1 | 1 (0.44) | 0 | 0 (0.00) | 1 | 1 (0.88) | 0 | 0 (0.00) |
| Hypersensitivity | 1 | 1 (0.44) | 0 | 0 (0.00) | 1 | 1 (0.88) | 0 | 0 (0.00) |
| **Infections and infestation** | 159 | 116 (50.88) | 86 | 63 (55.26) | 73 | 53 (46.49) | 48 | 29 (50.88) |
| Amoebiasis | 1 | 1 (0.44) | 1 | 1 (0.88) | 0 | 0 (0.00) | 0 | 0 (0.00) |
| Carbuncle | 2 | 1 (0.44) | 2 | 1 (0.88) | 0 | 0 (0.00) | 3 | 3 (5.26) |
| Conjunctivitis viral | 3 | 3 (1.32) | 2 | 2 (1.75) | 1 | 1 (0.88) | 0 | 0 (0.00) |
| Exanthema subitum | 2 | 2 (0.88) | 2 | 2 (1.75) | 0 | 0 (0.00) | 0 | 0 (0.00) |
| Herpangina | 2 | 2 (0.88) | 1 | 1 (0.88) | 1 | 1 (0.88) | 0 | 0 (0.00) |
| Impetigo | 4 | 4 (1.75) | 1 | 1 (0.88) | 3 | 3 (2.63) | 2 | 1 (1.75) |
| Nasopharyngitis | 2 | 2 (0.88) | 1 | 1 (0.88) | 1 | 1 (0.88) | 0 | 0 (0.00) |
| Otitis media | 1 | 1 (0.44) | 1 | 1 (0.88) | 0 | 0 (0.00) | 1 | 1 (1.75) |
| Otitis media acute | 3 | 3 (1.32) | 0 | 0 (0.00) | 3 | 3 (2.63) | 0 | 0 (0.00) |
| Parasitic gastroenteritis | 2 | 2 (0.88) | 2 | 2 (1.75) | 0 | 0 (0.00) | 0 | 0 (0.00) |
| Perichondritis | 0 | 0 (0.00) | 0 | 0 (0.00) | 0 | 0 (0.00) | 1 | 1 (1.75) |
| Pneumonia | 6 | 6 (2.63) | 3 | 3 (2.63) | 3 | 3 (2.63) | 3 | 3 (5.26) |
| Rhinitis | 19 | 19 (8.33) | 11 | 11 (9.65) | 8 | 8 (7.02) | 6 | 6 (10.53) |
| Upper respiratory tract infection | 94 | 82 (35.96) | 49 | 44 (38.60) | 45 | 38 (33.33) | 22 | 19 (33.33) |
| Urinary tract infection | 9 | 9 (3.95) | 5 | 5 (4.39) | 4 | 4 (3.51) | 3 | 3 (5.26) |
| Varicella | 0 | 0 (0.00) | 0 | 0 (0.00) | 0 | 0 (0.00) | 1 | 1 (1.75) |
| Viral infection | 7 | 7 (3.07) | 3 | 3 (2.63) | 4 | 4 (3.51) | 6 | 5 (8.77) |
| Viral rash | 2 | 2 (0.88) | 2 | 2 (1.75) | 0 | 0 (0.00) | 0 | 0 (0.00) |
| **Injury, poisoning and procedural complications** | 6 | 6 (2.63) | 5 | 5 (4.39) | 1 | 1 (0.88) | 1 | 1 (1.75) |
| Animal bite | 2 | 2 (0.88) | 2 | 2 (1.75) | 0 | 0 (0.00) | 0 | 0 (0.00) |
| Animal scratch | 1 | 1 (0.44) | 1 | 1 (0.88) | 0 | 0 (0.00) | 0 | 0 (0.00) |
| Joint injury | 1 | 1 (0.44) | 1 | 1 (0.88) | 0 | 0 (0.00) | 1 | 1 (1.75) |
| Traumatic haematoma | 1 | 1 (0.44) | 1 | 1 (0.88) | 0 | 0 (0.00) | 0 | 0 (0.00) |
| Wound | 1 | 1 (0.44) | 0 | 0 (0.00) | 1 | 1 (0.88) | 0 | 0 (0.00) |
| **Metabolism and nutrition disorders** | 0 | 0 (0.00) | 0 | 0 (0.00) | 0 | 0 (0.00) | 1 | 1 (1.75) |
| Food intolerance | 0 | 0 (0.00) | 0 | 0 (0.00) | 0 | 0 (0.00) | 1 | 1 (1.75) |
| **Nervous system disorders** | 1 | 1 (0.44) | 1 | 1 (0.88) | 0 | 0 (0.00) | 1 | 1 (1.75) |
| Febrile convulsion | 1 | 1 (0.44) | 1 | 1 (0.88) | 0 | 0 (0.00) | 1 | 1 (1.75) |
| **Psychiatric disorders** | 0 | 0 (0.00) | 0 | 0 (0.00) | 0 | 0 (0.00) | 1 | 1 (1.75) |
| Irritability | 0 | 0 (0.00) | 0 | 0 (0.00) | 0 | 0 (0.00) | 1 | 1 (1.75) |
| **Respiratory, thoracic and mediastinal disorders** | 61 | 51 (22.37) | 36 | 30 (26.32) | 25 | 21 (18.42) | 15 | 15 (26.32) |
| Bronchiolitis | 0 | 0 (0.00) | 0 | 0 (0.00) | 0 | 0 (0.00) | 1 | 1 (1.75) |
| Bronchitis | 33 | 31 (13.60) | 20 | 19 (16.67) | 13 | 12 (10.53) | 6 | 6 (10.53) |
| Cough | 1 | 1 (0.44) | 1 | 1 (0.88) | 0 | 0 (0.00) | 0 | 0 (0.00) |
| Nasopharyngitis | 25 | 23 (10.09) | 14 | 14 (12.28) | 11 | 9 (7.89) | 7 | 7 (12.28) |
| Pharyngotonsillitis | 2 | 2 (0.88) | 1 | 1 (0.88) | 1 | 1 (0.88) | 0 | 0 (0.00) |
| Upper respiratory tract infection | 0 | 0 (0.00) | 0 | 0 (0.00) | 0 | 0 (0.00) | 1 | 1 (1.75) |
| **Skin and subcutaneous tissue disorders** | 17 | 17 (7.46) | 7 | 7 (6.14) | 10 | 10 (8.77) | 5 | 4 (7.02) |
| Dermatitis | 0 | 0 (0.00) | 0 | 0 (0.00) | 0 | 0 (0.00) | 1 | 1 (1.75) |
| Dermatitis contact | 1 | 1 (0.44) | 0 | 0 (0.00) | 1 | 1 (0.88) | 1 | 1 (1.75) |
| Dermatitis diaper | 4 | 4 (1.75) | 2 | 2 (1.75) | 2 | 2 (1.75) | 2 | 1 (1.75) |
| Dermatitis infected | 1 | 1 (0.44) | 1 | 1 (0.88) | 0 | 0 (0.00) | 0 | 0 (0.00) |
| Folliculitis | 6 | 6 (2.63) | 1 | 1 (0.88) | 5 | 5 (4.39) | 0 | 0 (0.00) |
| Intertrigo | 1 | 1 (0.44) | 1 | 1 (0.88) | 0 | 0 (0.00) | 0 | 0 (0.00) |
| Miliaria | 4 | 4 (1.75) | 2 | 2 (1.75) | 2 | 2 (1.75) | 1 | 1 (1.75) |
| **AgeStrata1** | **99** | **54 (71.05)** | **46** | **28 (73.68)** | **53** | **26 (68.42)** | **38** | **16 (84.21)** |
| **Gastrointestinal disorders** | 15 | 14 (18.42) | 6 | 6 (15.79) | 9 | 8 (21.05) | 10 | 8 (42.11) |
| Epigastric discomfort | 1 | 1 (1.32) | 0 | 0 (0.00) | 1 | 1 (2.63) | 0 | 0 (0.00) |
| Gastroenteritis | 14 | 14 (18.42) | 6 | 6 (15.79) | 8 | 8 (21.05) | 9 | 8 (42.11) |
| Stomatitis | 0 | 0 (0.00) | 0 | 0 (0.00) | 0 | 0 (0.00) | 1 | 1 (5.26) |
| **General disorders and administration site conditions** | 2 | 2 (2.63) | 0 | 0 (0.00) | 2 | 2 (5.26) | 2 | 2 (10.53) |
| Crying | 0 | 0 (0.00) | 0 | 0 (0.00) | 0 | 0 (0.00) | 1 | 1 (5.26) |
| Inflammation | 0 | 0 (0.00) | 0 | 0 (0.00) | 0 | 0 (0.00) | 1 | 1 (5.26) |
| Pyrexia | 2 | 2 (2.63) | 0 | 0 (0.00) | 2 | 2 (5.26) | 0 | 0 (0.00) |
| **Infections and infestation** | 56 | 42 (55.26) | 27 | 22 (57.89) | 29 | 20 (52.63) | 16 | 10 (52.63) |
| Conjunctivitis viral | 1 | 1 (1.32) | 1 | 1 (2.63) | 0 | 0 (0.00) | 0 | 0 (0.00) |
| Exanthema subitum | 1 | 1 (1.32) | 1 | 1 (2.63) | 0 | 0 (0.00) | 0 | 0 (0.00) |
| Impetigo | 1 | 1 (1.32) | 0 | 0 (0.00) | 1 | 1 (2.63) | 0 | 0 (0.00) |
| Otitis media acute | 1 | 1 (1.32) | 0 | 0 (0.00) | 1 | 1 (2.63) | 0 | 0 (0.00) |
| Parasitic gastroenteritis | 1 | 1 (1.32) | 1 | 1 (2.63) | 0 | 0 (0.00) | 0 | 0 (0.00) |
| Perichondritis | 0 | 0 (0.00) | 0 | 0 (0.00) | 0 | 0 (0.00) | 1 | 1 (5.26) |
| Pneumonia | 3 | 3 (3.95) | 1 | 1 (2.63) | 2 | 2 (5.26) | 0 | 0 (0.00) |
| Rhinitis | 8 | 8 (10.53) | 3 | 3 (7.89) | 5 | 5 (13.16) | 3 | 3 (15.79) |
| Upper respiratory tract infection | 35 | 28 (36.84) | 18 | 16 (42.11) | 17 | 12 (31.58) | 8 | 7 (36.84) |
| Urinary tract infection | 3 | 3 (3.95) | 1 | 1 (2.63) | 2 | 2 (5.26) | 2 | 2 (10.53) |
| Viral infection | 1 | 1 (1.32) | 0 | 0 (0.00) | 1 | 1 (2.63) | 2 | 2 (10.53) |
| Viral rash | 1 | 1 (1.32) | 1 | 1 (2.63) | 0 | 0 (0.00) | 0 | 0 (0.00) |
| **Nervous system disorders** | 1 | 1 (1.32) | 1 | 1 (2.63) | 0 | 0 (0.00) | 0 | 0 (0.00) |
| Febrile convulsion | 1 | 1 (1.32) | 1 | 1 (2.63) | 0 | 0 (0.00) | 0 | 0 (0.00) |
| **Psychiatric disorders** | 0 | 0 (0.00) | 0 | 0 (0.00) | 0 | 0 (0.00) | 1 | 1 (5.26) |
| Irritability | 0 | 0 (0.00) | 0 | 0 (0.00) | 0 | 0 (0.00) | 1 | 1 (5.26) |
| **Respiratory, thoracic and mediastinal disorders** | 23 | 18 (23.68) | 12 | 10 (26.32) | 11 | 8 (21.05) | 6 | 6 (31.58) |
| Bronchitis | 15 | 13 (17.11) | 8 | 7 (18.42) | 7 | 6 (15.79) | 3 | 3 (15.79) |
| Nasopharyngitis | 8 | 7 (9.21) | 4 | 4 (10.53) | 4 | 3 (7.89) | 3 | 3 (15.79) |
| **Skin and subcutaneous tissue disorders** | 2 | 2 (2.63) | 0 | 0 (0.00) | 2 | 2 (5.26) | 3 | 2 (10.53) |
| Dermatitis | 0 | 0 (0.00) | 0 | 0 (0.00) | 0 | 0 (0.00) | 1 | 1 (5.26) |
| Dermatitis diaper | 2 | 2 (2.63) | 0 | 0 (0.00) | 2 | 2 (5.26) | 2 | 1 (5.26) |
| **AgeStrata2** | **98** | **53 (69.74)** | **53** | **27 (71.05)** | **45** | **26 (68.42)** | **29** | **14 (73.68)** |
| **Gastrointestinal disorders** | 16 | 13 (17.11) | 9 | 6 (15.79) | 7 | 7 (18.42) | 6 | 4 (21.05) |
| Aphthous ulcer | 1 | 1 (1.32) | 1 | 1 (2.63) | 0 | 0 (0.00) | 0 | 0 (0.00) |
| Gastroenteritis | 13 | 11 (14.47) | 8 | 6 (15.79) | 5 | 5 (13.16) | 5 | 3 (15.79) |
| Stomatitis | 1 | 1 (1.32) | 0 | 0 (0.00) | 1 | 1 (2.63) | 0 | 0 (0.00) |
| Toothache | 1 | 1 (1.32) | 0 | 0 (0.00) | 1 | 1 (2.63) | 1 | 1 (5.26) |
| **General disorders and administration site conditions** | 5 | 5 (6.58) | 4 | 4 (10.53) | 1 | 1 (2.63) | 0 | 0 (0.00) |
| Pyrexia | 5 | 5 (6.58) | 4 | 4 (10.53) | 1 | 1 (2.63) | 0 | 0 (0.00) |
| **Immune system disorders** | 1 | 1 (1.32) | 0 | 0 (0.00) | 1 | 1 (2.63) | 0 | 0 (0.00) |
| Hypersensitivity | 1 | 1 (1.32) | 0 | 0 (0.00) | 1 | 1 (2.63) | 0 | 0 (0.00) |
| **Infections and infestation** | 50 | 35 (46.05) | 26 | 18 (47.37) | 24 | 17 (44.74) | 13 | 9 (47.37) |
| Amoebiasis | 1 | 1 (1.32) | 1 | 1 (2.63) | 0 | 0 (0.00) | 0 | 0 (0.00) |
| Carbuncle | 2 | 1 (1.32) | 2 | 1 (2.63) | 0 | 0 (0.00) | 2 | 2 (10.53) |
| Exanthema subitum | 1 | 1 (1.32) | 1 | 1 (2.63) | 0 | 0 (0.00) | 0 | 0 (0.00) |
| Impetigo | 1 | 1 (1.32) | 0 | 0 (0.00) | 1 | 1 (2.63) | 0 | 0 (0.00) |
| Nasopharyngitis | 1 | 1 (1.32) | 1 | 1 (2.63) | 0 | 0 (0.00) | 0 | 0 (0.00) |
| Otitis media acute | 1 | 1 (1.32) | 0 | 0 (0.00) | 1 | 1 (2.63) | 0 | 0 (0.00) |
| Pneumonia | 3 | 3 (3.95) | 2 | 2 (5.26) | 1 | 1 (2.63) | 2 | 2 (10.53) |
| Rhinitis | 3 | 3 (3.95) | 3 | 3 (7.89) | 0 | 0 (0.00) | 1 | 1 (5.26) |
| Upper respiratory tract infection | 30 | 26 (34.21) | 13 | 11 (28.95) | 17 | 15 (39.47) | 7 | 7 (36.84) |
| Urinary tract infection | 4 | 4 (5.26) | 2 | 2 (5.26) | 2 | 2 (5.26) | 1 | 1 (5.26) |
| Viral infection | 3 | 3 (3.95) | 1 | 1 (2.63) | 2 | 2 (5.26) | 0 | 0 (0.00) |
| **Injury, poisoning and procedural complications** | 1 | 1 (1.32) | 1 | 1 (2.63) | 0 | 0 (0.00) | 0 | 0 (0.00) |
| Traumatic haematoma | 1 | 1 (1.32) | 1 | 1 (2.63) | 0 | 0 (0.00) | 0 | 0 (0.00) |
| **Metabolism and nutrition disorders** | 0 | 0 (0.00) | 0 | 0 (0.00) | 0 | 0 (0.00) | 1 | 1 (5.26) |
| Food intolerance | 0 | 0 (0.00) | 0 | 0 (0.00) | 0 | 0 (0.00) | 1 | 1 (5.26) |
| **Nervous system disorders** | 0 | 0 (0.00) | 0 | 0 (0.00) | 0 | 0 (0.00) | 1 | 1 (5.26) |
| Febrile convulsion | 0 | 0 (0.00) | 0 | 0 (0.00) | 0 | 0 (0.00) | 1 | 1 (5.26) |
| **Respiratory, thoracic and mediastinal disorders** | 17 | 15 (19.74) | 11 | 10 (26.32) | 6 | 5 (13.16) | 7 | 7 (36.84) |
| Bronchiolitis | 0 | 0 (0.00) | 0 | 0 (0.00) | 0 | 0 (0.00) | 1 | 1 (5.26) |
| Bronchitis | 6 | 6 (7.89) | 5 | 5 (13.16) | 1 | 1 (2.63) | 3 | 3 (15.79) |
| Nasopharyngitis | 11 | 10 (13.16) | 6 | 6 (15.79) | 5 | 4 (10.53) | 3 | 3 (15.79) |
| **Skin and subcutaneous tissue disorders** | 8 | 8 (10.53) | 2 | 2 (5.26) | 6 | 6 (15.79) | 1 | 1 (5.26) |
| Dermatitis contact | 1 | 1 (1.32) | 0 | 0 (0.00) | 1 | 1 (2.63) | 1 | 1 (5.26) |
| Dermatitis diaper | 2 | 2 (2.63) | 2 | 2 (5.26) | 0 | 0 (0.00) | 0 | 0 (0.00) |
| Folliculitis | 3 | 3 (3.95) | 0 | 0 (0.00) | 3 | 3 (7.89) | 0 | 0 (0.00) |
| Miliaria | 2 | 2 (2.63) | 0 | 0 (0.00) | 2 | 2 (5.26) | 0 | 0 (0.00) |
| **AgeStrata3** | **104** | **55 (72.37)** | **66** | **30 (78.95)** | **38** | **25 (65.79)** | **31** | **12 (63.16)** |
| **Gastrointestinal disorders** | 17 | 16 (21.05) | 10 | 10 (26.32) | 7 | 6 (15.79) | 7 | 7 (36.84) |
| Aphthous ulcer | 1 | 1 (1.32) | 0 | 0 (0.00) | 1 | 1 (2.63) | 0 | 0 (0.00) |
| Gastroenteritis | 15 | 14 (18.42) | 9 | 9 (23.68) | 6 | 5 (13.16) | 6 | 6 (31.58) |
| Stomatitis | 1 | 1 (1.32) | 1 | 1 (2.63) | 0 | 0 (0.00) | 0 | 0 (0.00) |
| Toothache | 0 | 0 (0.00) | 0 | 0 (0.00) | 0 | 0 (0.00) | 1 | 1 (5.26) |
| **General disorders and administration site conditions** | 1 | 1 (1.32) | 1 | 1 (2.63) | 0 | 0 (0.00) | 1 | 1 (5.26) |
| Pyrexia | 1 | 1 (1.32) | 1 | 1 (2.63) | 0 | 0 (0.00) | 1 | 1 (5.26) |
| **Infections and infestation** | 53 | 39 (51.32) | 33 | 23 (60.53) | 20 | 16 (42.11) | 19 | 10 (52.63) |
| Carbuncle | 0 | 0 (0.00) | 0 | 0 (0.00) | 0 | 0 (0.00) | 1 | 1 (5.26) |
| Conjunctivitis viral | 2 | 2 (2.63) | 1 | 1 (2.63) | 1 | 1 (2.63) | 0 | 0 (0.00) |
| Herpangina | 2 | 2 (2.63) | 1 | 1 (2.63) | 1 | 1 (2.63) | 0 | 0 (0.00) |
| Impetigo | 2 | 2 (2.63) | 1 | 1 (2.63) | 1 | 1 (2.63) | 2 | 1 (5.26) |
| Nasopharyngitis | 1 | 1 (1.32) | 0 | 0 (0.00) | 1 | 1 (2.63) | 0 | 0 (0.00) |
| Otitis media | 1 | 1 (1.32) | 1 | 1 (2.63) | 0 | 0 (0.00) | 1 | 1 (5.26) |
| Otitis media acute | 1 | 1 (1.32) | 0 | 0 (0.00) | 1 | 1 (2.63) | 0 | 0 (0.00) |
| Parasitic gastroenteritis | 1 | 1 (1.32) | 1 | 1 (2.63) | 0 | 0 (0.00) | 0 | 0 (0.00) |
| Pneumonia | 0 | 0 (0.00) | 0 | 0 (0.00) | 0 | 0 (0.00) | 1 | 1 (5.26) |
| Rhinitis | 8 | 8 (10.53) | 5 | 5 (13.16) | 3 | 3 (7.89) | 2 | 2 (10.53) |
| Upper respiratory tract infection | 29 | 28 (36.84) | 18 | 17 (44.74) | 11 | 11 (28.95) | 7 | 5 (26.32) |
| Urinary tract infection | 2 | 2 (2.63) | 2 | 2 (5.26) | 0 | 0 (0.00) | 0 | 0 (0.00) |
| Varicella | 0 | 0 (0.00) | 0 | 0 (0.00) | 0 | 0 (0.00) | 1 | 1 (5.26) |
| Viral infection | 3 | 3 (3.95) | 2 | 2 (5.26) | 1 | 1 (2.63) | 4 | 3 (15.79) |
| Viral rash | 1 | 1 (1.32) | 1 | 1 (2.63) | 0 | 0 (0.00) | 0 | 0 (0.00) |
| **Injury, poisoning and procedural complications** | 5 | 5 (6.58) | 4 | 4 (10.53) | 1 | 1 (2.63) | 1 | 1 (5.26) |
| Animal bite | 2 | 2 (2.63) | 2 | 2 (5.26) | 0 | 0 (0.00) | 0 | 0 (0.00) |
| Animal scratch | 1 | 1 (1.32) | 1 | 1 (2.63) | 0 | 0 (0.00) | 0 | 0 (0.00) |
| Joint injury | 1 | 1 (1.32) | 1 | 1 (2.63) | 0 | 0 (0.00) | 1 | 1 (5.26) |
| Wound | 1 | 1 (1.32) | 0 | 0 (0.00) | 1 | 1 (2.63) | 0 | 0 (0.00) |
| **Respiratory, thoracic and mediastinal disorders** | 21 | 18 (23.68) | 13 | 10 (26.32) | 8 | 8 (21.05) | 2 | 2 (10.53) |
| Bronchitis | 12 | 12 (15.79) | 7 | 7 (18.42) | 5 | 5 (13.16) | 0 | 0 (0.00) |
| Cough | 1 | 1 (1.32) | 1 | 1 (2.63) | 0 | 0 (0.00) | 0 | 0 (0.00) |
| Nasopharyngitis | 6 | 6 (7.89) | 4 | 4 (10.53) | 2 | 2 (5.26) | 1 | 1 (5.26) |
| Pharyngotonsillitis | 2 | 2 (2.63) | 1 | 1 (2.63) | 1 | 1 (2.63) | 0 | 0 (0.00) |
| Upper respiratory tract infection | 0 | 0 (0.00) | 0 | 0 (0.00) | 0 | 0 (0.00) | 1 | 1 (5.26) |
| **Skin and subcutaneous tissue disorders** | 7 | 7 (9.21) | 5 | 5 (13.16) | 2 | 2 (5.26) | 1 | 1 (5.26) |
| Dermatitis infected | 1 | 1 (1.32) | 1 | 1 (2.63) | 0 | 0 (0.00) | 0 | 0 (0.00) |
| Folliculitis | 3 | 3 (3.95) | 1 | 1 (2.63) | 2 | 2 (5.26) | 0 | 0 (0.00) |
| Intertrigo | 1 | 1 (1.32) | 1 | 1 (2.63) | 0 | 0 (0.00) | 0 | 0 (0.00) |
| Miliaria | 2 | 2 (2.63) | 2 | 2 (5.26) | 0 | 0 (0.00) | 1 | 1 (5.26) |

Table S 12 Proportion of subjects with unsolicited AE within 4 weeks after each vaccination (Safety Analysis Set)

|  | **Vi-DT Group** | | | | | | | | | | **Group-C:**  **Comparator Group** | | |  |
| --- | --- | --- | --- | --- | --- | --- | --- | --- | --- | --- | --- | --- | --- | --- |
|  | **Total** | | | **Group A: Single dose** | | | | **Group-B: Two-dose** | | |  |  |  |  |
| **Within 4 weeks after first dose** | **N** | **Number of participants (%)** | **95% CI** | **N** | **Number of participants (%)** | **95% CI** | **N** | | **Number of participants (%)** | **95% CI** | **N** | **Number of participants (%)** | **95% CI** | **P-value**† |
| All ages | 228 | 140 (61·40) | (54·95, 67·48) | 114 | 74 (64·91) | (55·80, 73·06) | 114 | | 66 (57·89) | (48·72, 66·56) | 57 | 39 (68·42) | (55·52, 79·00) | 0·3422 |
| AgeStrata1 | 76 | 44 (57·89) | (46·68, 68·35) | 38 | 23 (60·53) | (44·72, 74·40) | 38 | | 21 (55·26) | (39·71, 69·85) | 19 | 16 (84·21) | (62·43, 94·48) | -- |
| AgeStrata2 | 76 | 50 (65·79) | (54·60, 75·46) | 38 | 25 (65·79) | (49·89, 78·79) | 38 | | 25 (65·79) | (49·89, 78·79) | 19 | 12 (63·16) | (41·04, 80·85) | -- |
| AgeStrata3 | 76 | 46 (60·53) | (49·29, 70·75) | 38 | 26 (68·42) | (52·54, 80·92) | 38 | | 20 (52·63) | (37·26, 67·52) | 19 | 11 (57·89) | (36·28, 76·86) | -- |
| **Within 4 weeks after second dose** | **N** | **Number of participants (%)** | **95% CI** | **N** | **Number of participants (%)** | **95% CI** | **N** | | **Number of participants (%)** | **95% CI** | **N** | **Number of participants (%)** | **95% CI** | **P-value**† |
| All ages | 220 | 72 (32·73) | (26·87, 39·18) | 112 | 39 (34·82) | (26·63, 44·01) | 108 | | 33 (30·56) | (22·66, 39·79) | 55 | 15 (27·27) | (17·28, 40·23) | 0·5057 |
| AgeStrata1 | 75 | 26 (34·67) | (24·88, 45·95) | 38 | 11 (28·95) | (17·00, 44·76) | 37 | | 15 (40·54) | (26·35, 56·51) | 17 | 6 (35·29) | (17·31, 58·70) | -- |
| AgeStrata2 | 69 | 16 (23·19) | (14·81, 34·40) | 36 | 9 (25·00) | (13·75, 41·07) | 33 | | 7 (21·21) | (10·68, 37·75) | 19 | 3 (15·79) | (5·52, 37·57) | -- |
| AgeStrata3 | 76 | 30 (39·47) | (29·25, 50·71) | 38 | 19 (50·00) | (34·85, 65·15) | 38 | | 11 (28·95) | (17·00, 44·76) | 19 | 6 (31·58) | (15·36, 53·99) | -- |
| **Within 4 weeks after any dose** | **N** | **Number of participants (%)** | **95% CI** | **N** | **Number of participants (%)** | **95% CI** | **N** | | **Number of participants (%)** | **95% CI** | **N** | **Number of participants (%)** | **95% CI** | **P-value**† |
| All ages | 228 | 162 (71·05) | (64·86, 76·55) | 114 | 85 (74·56) | (65·86, 81·66) | 114 | | 77 (67·54) | (58·50, 75·44) | 57 | 42 (73·68) | (61·02, 83·35) | 0·4675 |
| AgeStrata1 | 76 | 54 (71·05) | (60·04, 80·04) | 38 | 28 (73·68) | (57·99, 85·03) | 38 | | 26 (68·42) | (52·54, 80·92) | 19 | 16 (84·21) | (62·43, 94·48) | -- |
| AgeStrata2 | 76 | 53 (69·74) | (58·67, 78·91) | 38 | 27 (71·05) | (55·24, 83·00) | 38 | | 26 (68·42) | (52·54, 80·92) | 19 | 14 (73·68) | (51·21, 88·19) | -- |
| AgeStrata3 | 76 | 55 (72·37) | (61·42, 81·16) | 38 | 30 (78·95) | (63·65, 88·93) | 38 | | 25 (65·79) | (49·89, 78·79) | 19 | 12 (63·16) | (41·04, 80·85) | -- |

**Table S13** Summary of proportion of subject with medically significant unsolicited AE (except 4 weeks after each vaccination)

|  | **Vi-DT Group** | | |  | | |  | | | **Comparator Group** | | |  |
| --- | --- | --- | --- | --- | --- | --- | --- | --- | --- | --- | --- | --- | --- |
|  |  |  |  | **Single dose** | | | **two-dose** | | |  |  |  |  |
| **Entire study period** | **N** | **Number of participants (%)** | **95% CI** | **N** | **Number of participants (%)** | **95% CI** | **N** | **Number of participants (%)** | **95% CI** | **N** | **Number of participants (%)** | **95% CI** | **P-value**† |
| All ages | 228 | 52 (22·81) | (17·84, 28·68) | 114 | 27 (23·68) | (16·82, 32·27) | 114 | 25 (21·93) | (15·32, 30·37) | 57 | 22 (38·60) | (27·06, 51·57) | 0·0510 |
| AgeStrata1 | 76 | 19 (25·00) | (16·63, 35·78) | 38 | 11 (28·95) | (17·00, 44·76) | 38 | 8 (21·05) | (11·07, 36·35) | 19 | 4 (21·05) | (8·51, 43·33) | -- |
| AgeStrata2 | 76 | 18 (23·68) | (15·54, 34·36) | 38 | 11 (28·95) | (17·00, 44·76) | 38 | 7 (18·42) | (9·22, 33·42) | 19 | 9 (47·37) | (27·33, 68·29) | -- |
| AgeStrata3 | 76 | 15 (19·74) | (12·34, 30·04) | 38 | 5 (13·16) | (5·75, 27·33) | 38 | 10(26·32) | (14·97, 42·01) | 19 | 9 (47·37) | (27·33, 68·29) | -- |

† P-values for all ages have been derived using stratified Chi-square (Cochran-Mantel-Haenszel) test stratified by age· (Vi-DT single vs· two-dose Group vs· Comparator Group)

Source: TFL Table 10·5·8

**Table S14** Distribution of medically significant unsolicited AE (except 4 weeks after each vaccination)

| **SOC\PT** | **Vi-DT Group**  **(N=228)** | |  |  |  |  | **Comparator Group (N=57)** | |
| --- | --- | --- | --- | --- | --- | --- | --- | --- |
|  |  |  | **Single dose**  **(N=114)** | | **two-dose**  **(N=114)** | |  |  |
| **Entire study period** | **# of AEs** | **Number of participants (%)** | **# of AEs** | **Number of participants (%)** | **# of AEs** | **Number of participants (%)** | **# of AEs** | **Number of participants (%)** |
| **All ages** | **75** | **52 (22·81)** | **42** | **27 (23·68)** | **33** | **25 (21·93)** | **34** | **22 (38·60)** |
| **Blood and lymphatic system disorder** | 3 | 3 (1·32) | 1 | 1 (0·88) | 2 | 2 (1·75) | 0 | 0 (0·00) |
| Anaemia | 3 | 3 (1·32) | 1 | 1 (0·88) | 2 | 2 (1·75) | 0 | 0 (0·00) |
| **Gastrointestinal disorder** | 12 | 11 (4·82) | 7 | 6 (5·26) | 5 | 5 (4·39) | 3 | 3 (5·26) |
| Ascariasis | 1 | 1 (0·44) | 0 | 0 (0·00) | 1 | 1 (0·88) | 0 | 0 (0·00) |
| Gastroenteritis | 10 | 9 (3·95) | 7 | 6 (5·26) | 3 | 3 (2·63) | 3 | 3 (5·26) |
| Inguinal hernia | 1 | 1 (0·44) | 0 | 0 (0·00) | 1 | 1 (0·88) | 0 | 0 (0·00) |
| **General disorders and administration site condition** | 2 | 2 (0·88) | 1 | 1 (0·88) | 1 | 1 (0·88) | 0 | 0 (0·00) |
| Pyrexia | 2 | 2 (0·88) | 1 | 1 (0·88) | 1 | 1 (0·88) | 0 | 0 (0·00) |
| **Immune system disorder** | 1 | 1 (0·44) | 0 | 0 (0·00) | 1 | 1 (0·88) | 0 | 0 (0·00) |
| Hypersensitivity | 1 | 1 (0·44) | 0 | 0 (0·00) | 1 | 1 (0·88) | 0 | 0 (0·00) |
| **Infections and infestation** | 48 | 38 (16·67) | 27 | 21 (18·42) | 21 | 17 (14·91) | 29 | 21 (36·84) |
| Amoebiasis | 0 | 0 (0·00) | 0 | 0 (0·00) | 0 | 0 (0·00) | 1 | 1 (1·75) |
| Amoebic dysentery | 1 | 1 (0·44) | 0 | 0 (0·00) | 1 | 1 (0·88) | 0 | 0 (0·00) |
| Bronchitis | 13 | 13 (5·70) | 9 | 9 (7·89) | 4 | 4 (3·51) | 8 | 8 (14·04) |
| arbuncle | 1 | 1 (0·44) | 1 | 1 (0·88) | 0 | 0 (0·00) | 1 | 1 (1·75) |
| Conjunctivitis viral | 0 | 0 (0·00) | 0 | 0 (0·00) | 0 | 0 (0·00) | 1 | 1 (1·75) |
| Exanthema subitum | 1 | 1 (0·44) | 1 | 1 (0·88) | 0 | 0 (0·00) | 0 | 0 (0·00) |
| Hand-foot-and-mouth disease | 0 | 0 (0·00) | 0 | 0 (0·00) | 0 | 0 (0·00) | 1 | 1 (1·75) |
| Herpangina | 0 | 0 (0·00) | 0 | 0 (0·00) | 0 | 0 (0·00) | 1 | 1 (1·75) |
| Hordeolum | 0 | 0 (0·00) | 0 | 0 (0·00) | 0 | 0 (0·00) | 1 | 1 (1·75) |
| Impetigo | 3 | 3 (1·32) | 1 | 1 (0·88) | 2 | 2 (1·75) | 1 | 1 (1·75) |
| Infections NEC | 1 | 1 (0·44) | 0 | 0 (0·00) | 1 | 1 (0·88) | 0 | 0 (0·00) |
| Oral candidiasis | 1 | 1 (0·44) | 1 | 1 (0·88) | 0 | 0 (0·00) | 1 | 1 (1·75) |
| Otitis media acute | 1 | 1 (0·44) | 0 | 0 (0·00) | 1 | 1 (0·88) | 0 | 0 (0·00) |
| Parasitic gastroenteritis | 1 | 1 (0·44) | 0 | 0 (0·00) | 1 | 1 (0·88) | 0 | 0 (0·00) |
| Pneumonia | 9 | 9 (3·95) | 7 | 7 (6·14) | 2 | 2 (1·75) | 4 | 4 (7·02) |
| Upper respiratory tract infection | 13 | 11 (4·82) | 7 | 5 (4·39) | 6 | 6 (5·26) | 3 | 3 (5·26) |
| Urinary tract infection | 2 | 2 (0·88) | 0 | 0 (0·00) | 2 | 2 (1·75) | 4 | 4 (7·02) |
| Viral infection | 1 | 1 (0·44) | 0 | 0 (0·00) | 1 | 1 (0·88) | 2 | 2 (3·51) |
| **Injury, poisoning and procedural complication** | 1 | 1 (0·44) | 1 | 1 (0·88) | 0 | 0 (0·00) | 1 | 1 (1·75) |
| Animal bite | 1 | 1 (0·44) | 1 | 1 (0·88) | 0 | 0 (0·00) | 1 | 1 (1·75) |
| **Musculoskeletal and connective tissue disorder** | 1 | 1 (0·44) | 1 | 1 (0·88) | 0 | 0 (0·00) | 0 | 0 (0·00) |
| Abscess neck | 1 | 1 (0·44) | 1 | 1 (0·88) | 0 | 0 (0·00) | 0 | 0 (0·00) |
| **Nervous system disorder** | 2 | 2 (0·88) | 0 | 0 (0·00) | 2 | 2 (1·75) | 0 | 0 (0·00) |
| Febrile convulsion | 2 | 2 (0·88) | 0 | 0 (0·00) | 2 | 2 (1·75) | 0 | 0 (0·00) |
| **Respiratory, thoracic and mediastinal disorder** | 3 | 3 (1·32) | 2 | 2 (1·75) | 1 | 1 (0·88) | 0 | 0 (0·00) |
| Bronchial hyperreactivity | 1 | 1 (0·44) | 1 | 1 (0·88) | 0 | 0 (0·00) | 0 | 0 (0·00) |
| Bronchiolitis | 1 | 1 (0·44) | 0 | 0 (0·00) | 1 | 1 (0·88) | 0 | 0 (0·00) |
| Pharyngotonsillitis | 1 | 1 (0·44) | 1 | 1 (0·88) | 0 | 0 (0·00) | 0 | 0 (0·00) |
| **Skin and subcutaneous tissue disorder** | 2 | 2 (0·88) | 2 | 2 (1·75) | 0 | 0 (0·00) | 1 | 1 (1·75) |
| Dermatitis diaper | 2 | 2 (0·88) | 2 | 2 (1·75) | 0 | 0 (0·00) | 0 | 0 (0·00) |
| Folliculitis | 0 | 0 (0·00) | 0 | 0 (0·00) | 0 | 0 (0·00) | 1 | 1 (1·75) |
| **Age Strata1** | **26** | **19 (25·00)** | **17** | **11 (28·95)** | **9** | **8 (21·05)** | **5** | **4 (21·05)** |
| **Blood and lymphatic system disorder** | 1 | 1 (1·32) | 0 | 0 (0·00) | 1 | 1 (2·63) | 0 | 0 (0·00) |
| Anaemia | 1 | 1 (1·32) | 0 | 0 (0·00) | 1 | 1 (2·63) | 0 | 0 (0·00) |
| **Gastrointestinal disorder** | 4 | 3 (3·95) | 2 | 1 (2·63) | 2 | 2 (5·26) | 0 | 0 (0·00) |
| Gastroenteritis | 4 | 3 (3·95) | 2 | 1 (2·63) | 2 | 2 (5·26) | 0 | 0 (0·00) |
| **General disorders and administration site condition** | 2 | 2 (2·63) | 1 | 1 (2·63) | 1 | 1 (2·63) | 0 | 0 (0·00) |
| Pyrexia | 2 | 2 (2·63) | 1 | 1 (2·63) | 1 | 1 (2·63) | 0 | 0 (0·00) |
| **Infections and infestation** | 17 | 14 (18·42) | 13 | 10 (26·32) | 4 | 4 (10·53) | 5 | 4 (21·05) |
| Bronchitis | 5 | 5 (6·58) | 5 | 5 (13·16) | 0 | 0 (0·00) | 3 | 3 (15·79) |
| Carbuncle | 1 | 1 (1·32) | 1 | 1 (2·63) | 0 | 0 (0·00) | 0 | 0 (0·00) |
| Exanthema subitum | 1 | 1 (1·32) | 1 | 1 (2·63) | 0 | 0 (0·00) | 0 | 0 (0·00) |
| Infections NEC | 1 | 1 (1·32) | 0 | 0 (0·00) | 1 | 1 (2·63) | 0 | 0 (0·00) |
| Oral candidiasis | 1 | 1 (1·32) | 1 | 1 (2·63) | 0 | 0 (0·00) | 0 | 0 (0·00) |
| Pneumonia | 7 | 7 (9·21) | 5 | 5 (13·16) | 2 | 2 (5·26) | 0 | 0 (0·00) |
| Upper respiratory tract infection | 1 | 1 (1·32) | 0 | 0 (0·00) | 1 | 1 (2·63) | 0 | 0 (0·00) |
| Urinary tract infection | 0 | 0 (0·00) | 0 | 0 (0·00) | 0 | 0 (0·00) | 1 | 1 (5·26) |
| Viral infection | 0 | 0 (0·00) | 0 | 0 (0·00) | 0 | 0 (0·00) | 1 | 1 (5·26) |
| **Nervous system disorder** | 1 | 1 (1·32) | 0 | 0 (0·00) | 1 | 1 (2·63) | 0 | 0 (0·00) |
| Febrile convulsion | 1 | 1 (1·32) | 0 | 0 (0·00) | 1 | 1 (2·63) | 0 | 0 (0·00) |
| **Skin and subcutaneous tissue disorder** | 1 | 1 (1·32) | 1 | 1 (2·63) | 0 | 0 (0·00) | 0 | 0 (0·00) |
| Dermatitis diaper | 1 | 1 (1·32) | 1 | 1 (2·63) | 0 | 0 (0·00) | 0 | 0 (0·00) |
| **Age Strata2** | **25** | **18 (23·68)** | **18** | **11 (28·95)** | **7** | **7 (18·42)** | **13** | **9 (47·37)** |
| **Blood and lymphatic system disorder** | 2 | 2 (2·63) | 1 | 1 (2·63) | 1 | 1 (2·63) | 0 | 0 (0·00) |
| Anaemia | 2 | 2 (2·63) | 1 | 1 (2·63) | 1 | 1 (2·63) | 0 | 0 (0·00) |
| **Gastrointestinal disorder** | 5 | 5 (6·58) | 4 | 4 (10·53) | 1 | 1 (2·63) | 1 | 1 (5·26) |
| Gastroenteritis | 4 | 4 (5·26) | 4 | 4 (10·53) | 0 | 0 (0·00) | 1 | 1 (5·26) |
| Inguinal hernia | 1 | 1 (1·32) | 0 | 0 (0·00) | 1 | 1 (2·63) | 0 | 0 (0·00) |
| **Infections and infestation** | 14 | 12 (15·79) | 9 | 7 (18·42) | 5 | 5 (13·16) | 12 | 9 (47·37) |
| Amoebiasis | 0 | 0 (0·00) | 0 | 0 (0·00) | 0 | 0 (0·00) | 1 | 1 (5·26) |
| Bronchitis | 3 | 3 (3·95) | 2 | 2 (5·26) | 1 | 1 (2·63) | 3 | 3 (15·79) |
| Conjunctivitis viral | 0 | 0 (0·00) | 0 | 0 (0·00) | 0 | 0 (0·00) | 1 | 1 (5·26) |
| Herpangina | 0 | 0 (0·00) | 0 | 0 (0·00) | 0 | 0 (0·00) | 1 | 1 (5·26) |
| Hordeolum | 0 | 0 (0·00) | 0 | 0 (0·00) | 0 | 0 (0·00) | 1 | 1 (5·26) |
| Pneumonia | 1 | 1 (1·32) | 1 | 1 (2·63) | 0 | 0 (0·00) | 1 | 1 (5·26) |
| Upper respiratory tract infection | 9 | 7 (9·21) | 6 | 4 (10·53) | 3 | 3 (7·89) | 2 | 2 (10·53) |
| Urinary tract infection | 0 | 0 (0·00) | 0 | 0 (0·00) | 0 | 0 (0·00) | 1 | 1 (5·26) |
| Viral infection | 1 | 1 (1·32) | 0 | 0 (0·00) | 1 | 1 (2·63) | 1 | 1 (5·26) |
| **Injury, poisoning and procedural complication** | 1 | 1 (1·32) | 1 | 1 (2·63) | 0 | 0 (0·00) | 0 | 0 (0·00) |
| Animal bite | 1 | 1 (1·32) | 1 | 1 (2·63) | 0 | 0 (0·00) | 0 | 0 (0·00) |
| **Respiratory, thoracic and mediastinal disorder** | 2 | 2 (2·63) | 2 | 2 (5·26) | 0 | 0 (0·00) | 0 | 0 (0·00) |
| Bronchial hyperreactivity | 1 | 1 (1·32) | 1 | 1 (2·63) | 0 | 0 (0·00) | 0 | 0 (0·00) |
| Pharyngotonsillitis | 1 | 1 (1·32) | 1 | 1 (2·63) | 0 | 0 (0·00) | 0 | 0 (0·00) |
| **Skin and subcutaneous tissue disorder** | 1 | 1 (1·32) | 1 | 1 (2·63) | 0 | 0 (0·00) | 0 | 0 (0·00) |
| Dermatitis diaper | 1 | 1 (1·32) | 1 | 1 (2·63) | 0 | 0 (0·00) | 0 | 0 (0·00) |
| **AgeStrata3** | **24** | **15 (19·74)** | **7** | **5 (13·16)** | **17** | **10 (26·32)** | **16** | **9 (47·37)** |
| **Gastrointestinal disorder** | 3 | 3 (3·95) | 1 | 1 (2·63) | 2 | 2 (5·26) | 2 | 2 (10·53) |
| Ascariasis | 1 | 1 (1·32) | 0 | 0 (0·00) | 1 | 1 (2·63) | 0 | 0 (0·00) |
| Gastroenteritis | 2 | 2 (2·63) | 1 | 1 (2·63) | 1 | 1 (2·63) | 2 | 2 (10·53) |
| **Immune system disorder** | 1 | 1 (1·32) | 0 | 0 (0·00) | 1 | 1 (2·63) | 0 | 0 (0·00) |
| Hypersensitivity | 1 | 1 (1·32) | 0 | 0 (0·00) | 1 | 1 (2·63) | 0 | 0 (0·00) |
| **Infections and infestation** | 17 | 12 (15·79) | 5 | 4 (10·53) | 12 | 8 (21·05) | 12 | 8 (42·11) |
| Amoebic dysentery | 1 | 1 (1·32) | 0 | 0 (0·00) | 1 | 1 (2·63) | 0 | 0 (0·00) |
| Bronchitis | 5 | 5 (6·58) | 2 | 2 (5·26) | 3 | 3 (7·89) | 2 | 2 (10·53) |
| Carbuncle | 0 | 0 (0·00) | 0 | 0 (0·00) | 0 | 0 (0·00) | 1 | 1 (5·26) |
| Hand-foot-and-mouth disease | 0 | 0 (0·00) | 0 | 0 (0·00) | 0 | 0 (0·00) | 1 | 1 (5·26) |
| Impetigo | 3 | 3 (3·95) | 1 | 1 (2·63) | 2 | 2 (5·26) | 1 | 1 (5·26) |
| Oral candidiasis | 0 | 0 (0·00) | 0 | 0 (0·00) | 0 | 0 (0·00) | 1 | 1 (5·26) |
| Otitis media acute | 1 | 1 (1·32) | 0 | 0 (0·00) | 1 | 1 (2·63) | 0 | 0 (0·00) |
| Parasitic gastroenteritis | 1 | 1 (1·32) | 0 | 0 (0·00) | 1 | 1 (2·63) | 0 | 0 (0·00) |
| Pneumonia | 1 | 1 (1·32) | 1 | 1 (2·63) | 0 | 0 (0·00) | 3 | 3 (15·79) |
| Upper respiratory tract infection | 3 | 3 (3·95) | 1 | 1 (2·63) | 2 | 2 (5·26) | 1 | 1 (5·26) |
| Urinary tract infection | 2 | 2 (2·63) | 0 | 0 (0·00) | 2 | 2 (5·26) | 2 | 2 (10·53) |
| **Injury, poisoning and procedural complication** | 0 | 0 (0·00) | 0 | 0 (0·00) | 0 | 0 (0·00) | 1 | 1 (5·26) |
| Animal bite | 0 | 0 (0·00) | 0 | 0 (0·00) | 0 | 0 (0·00) | 1 | 1 (5·26) |
| **Musculoskeletal and connective tissue disorder** | 1 | 1 (1·32) | 1 | 1 (2·63) | 0 | 0 (0·00) | 0 | 0 (0·00) |
| Abscess neck | 1 | 1 (1·32) | 1 | 1 (2·63) | 0 | 0 (0·00) | 0 | 0 (0·00) |
| **Nervous system disorder** | 1 | 1 (1·32) | 0 | 0 (0·00) | 1 | 1 (2·63) | 0 | 0 (0·00) |
| Febrile convulsion | 1 | 1 (1·32) | 0 | 0 (0·00) | 1 | 1 (2·63) | 0 | 0 (0·00) |
| **Respiratory, thoracic and mediastinal disorder** | 1 | 1 (1·32) | 0 | 0 (0·00) | 1 | 1 (2·63) | 0 | 0 (0·00) |
| Bronchiolitis | 1 | 1 (1·32) | 0 | 0 (0·00) | 1 | 1 (2·63) | 0 | 0 (0·00) |
| **Skin and subcutaneous tissue disorder** | 0 | 0 (0·00) | 0 | 0 (0·00) | 0 | 0 (0·00) | 1 | 1 (5·26) |
| Folliculitis | 0 | 0 (0·00) | 0 | 0 (0·00) | 0 | 0 (0·00) | 1 | 1 (5·26) |

Table S15 Seroconversion of Anti-Vi IgG Response for all ages – immunogenicity set

| **Vi-DT Dose** | **Time point** | **Vi-DT Group** | | | | | | **Comparator Group** | | **P-value**† |
| --- | --- | --- | --- | --- | --- | --- | --- | --- | --- | --- |
|  |  | **Any dose** | | **Single dose** | | **two-dose** | |  |  |  |
|  |  | N | SCR (95%CI) | N | SCR (95%CI) | N | SCR (95%CI) | N | SCR (95%CI) |  |
| First Dose | Day 0 | 228 | -- | 114 | -- | 114 | -- | 57 | -- | -- |
|  | Week 4 | 228 | 100.0 (98.34, 100.0) | 114 | 100.0(96.74, 100.0) | 114 | 100.0(96.74, 100.0) | 57 | 7.02 (2.76, 16.70) | <0.0001^[1]^ |
| Second Dose | Week 24 | 222 | 98.20 (95.46, 99.30) | 112 | 99.11 (95.12, 99.84) | 110 | 97.27 (92.29, 99.07) | 55 | 21.82 (12.95, 34.37) | -- |
|  | Week 28 | 221 | 98.64 (96.09, 99.54) | 112 | 99.11 (95.12, 99.84) | 109 | 98.17 (93.56, 99.50) | 55 | 21.82 (12.95, 34.37) | <0.0001^[2]^  0.5508^[3]^ |

N – Number tested; SCN – Seroconversion rate

[1] Primary, Seroconversion of Anti-Vi IgG Response at Week 4 (Combined Vaccine group vs· Comparator)

[2] Secondary, Seroconversion of Anti-Vi IgG Response at Week 28 (Two-dose Vaccine group vs· Comparator)

Following comparision is tested without multiple testing adjustment, no decision-making criteria

[3] Seroconversion of Anti-Vi IgG Response at Week 28 (One-dose vs· Two-dose)

* Seroconversion of Anti-Vi IgG Response at Week 4 of single-dose group vs· Week 28 of two-dose group, p-value 0·1458

Table S16 Seroconversion of Anti-Vi IgG Response as per age strata – Immunogenicity set

| **Vi-DT Dose** | **Time point** | | **Vi-DT Group** | | | | | | | | | | | | | | | | | | | | | | | **Comparator Group** | | | | | | |
| --- | --- | --- | --- | --- | --- | --- | --- | --- | --- | --- | --- | --- | --- | --- | --- | --- | --- | --- | --- | --- | --- | --- | --- | --- | --- | --- | --- | --- | --- | --- | --- | --- |
|  |  |  | **Any dose** | | | | | | | | | | **Single dose** | | | | | | **two-dose** | | | | | | |  |  |  |  |  |  |  |
|  |  |  | N | | | | | | | SCN (95% CI) | | | N | | | | | SCN (95% CI) | N | | | | | SCN (95% CI) | | N | | | SCN (95% CI) | | | |
| **Age Strata1: 6 to less than 9 months** | | | | | | | | | | | | | | | | | | | | | | | | | | | | | | | | |
| First Dose | Day 0 | | 76 | | | | | | | -- | | | 38 | | | | | -- | 38 | | | | | -- | | 19 | | | -- | | | |
|  | Week 4 | | 76 | | | | | | | 100.0 (95.19, 100.0) | | | 38 | | | | | 100.0 (90.82, 100.0) | 38 | | | | | 100.0 (90.82, 100.0) | | 19 | | | 10.53 (2.94, 31.39) | | | |
| Second Dose | Week 24 | | 76 | | | | | | | 97.37 (90.90, 99.28) | | | 38 | | | | | 100.0 (90.82, 100.0) | 38 | | | | | 94.74 (82.71, 98.54) | | 17 | | | 17.65 (6.19, 41.03) | | | |
|  | Week 28 | | 75 | | | | | | | 100.0 (95.13, 100.0) | | | 38 | | | | | 100.0 (90.82, 100.0) | 37 | | | | | 100.0 (90.59, 100.0) | | 17 | | | 23.53 (9.56, 47.26) | | | |
| **Age Strata2: 9 to 12 m** | | | | | |  | | | | |  | | | | | | | | | | |  | | | | | | | | |  | |
| First Dose | | Day 0 | | 76 | | | | -- | | | | | | 38 | | -- | | | | 38 | | | | -- | 19 | | | -- | | | | |
|  |  | Week 4 | | 76 | | | | 100.0 (95.19, 100.0) | | | | | | 38 | | 100.0 (90.82, 100.0) | | | | 38 | | | | 100.0 (90.82, 100.0) | 19 | | | 5.26 (0.94, 24.64) | | | | |
| Second Dose | | Week 24 | | 70 | | | | 100.0 (94.80, 100.0) | | | | | | 36 | | 100.0 (90.36, 100.0) | | | | 34 | | | | 100.0 (89.85, 100.0) | 19 | | | 10.53 (2.94, 31.39) | | | | |
|  |  | Week 28 | | 70 | | | | 98.57 (92.34, 99.75) | | | | | | 36 | | 100.0 (90.36, 100.0) | | | | 34 | | | | 97.06 (85.08, 99.48) | 19 | | | 21.05 (8.51, 43.33) | | | | |
| **Age Strata3: 13 to 23 m** | | | | | | |  | | | | |  | | | | | | | | | | |  | | | | | | | | |  |
| First Dose | | Day 0 | | | 76 | | | | -- | | | | | | 38 | | -- | | | | 38 | | | -- | | | 19 | | | -- | | |
|  |  | Week 4 | | | 76 | | | | 100.0 (95.19, 100.0) | | | | | | 38 | | 100.0 (90.82, 100.0) | | | | 38 | | | 100.0 (90.82, 100.0) | | | 19 | | | 5.26 (0.94, 24.64) | | |
| Second Dose | | Week 24 | | | 76 | | | | 97.37 (90.90, 99.28) | | | | | | 38 | | 97.37 (86.51, 99.53) | | | | 38 | | | 97.37 (86.51, 99.53) | | | 19 | | | 36.84 (19.15, 58.96) | | |
|  |  | Week 28 | | | 76 | | | | 97.37 (90.90, 99.28) | | | | | | 38 | | 97.37 (86.51, 99.53) | | | | 38 | | | 97.37 (86.51, 99.53) | | | 19 | | | 21.05 (8.51, 43.33) | | |

N – Number tested; SCN – Seroconversion rate

Table S17 Seroconversion of Anti-Vi IgG Response for all ages – per protocol set

| **Vi-DT Dose** | **Time point** | **Vi-DT Group** | | | | | | **Comparator Group** | | **P-value**† |
| --- | --- | --- | --- | --- | --- | --- | --- | --- | --- | --- |
|  |  | **Any dose** | | **Single dose** | | **two-dose** | |  |  |  |
|  |  | N | SCR (95%CI) | N | SCR (95%CI) | N | SCR (95%CI) | N | SCR (95%CI) |  |
| First Dose | Day 0 | 216 | -- | 110 | -- | 106 | -- | 54 | -- | -- |
|  | Week 4 | 216 | 100.0 (98.25, 100.0) | 110 | 100.0 (96.63, 100.0) | 106 | 100.0 (96.50, 100.0) | 54 | 5.56 (1.91, 15.11) | <0.0001^[1]^ |
| Second Dose | Week 24 | 216 | 98.15 (95.34, 99.28) | 110 | 99.09 (95.03, 99.84) | 106 | 97.17 (92.01, 99.03) | 54 | 22.22 (13.20, 34.94) | -- |
|  | Week 28 | 216 | 98.61 (96.00, 99.53) | 110 | 99.09 (95.03, 99.84) | 106 | 98.11 (93.38, 99.48) | 54 | 20.37 (11.77, 32.90) | <0.0001^[2]^  0.5383^[3]^  0.5671^[4]^ |

N – Number tested; SCN – Seroconversion rate

[1] Primary, Seroconversion of Anti-Vi IgG Response at Week 4 (Combined Vaccine group vs· Comparator)

[2] Secondary, Seroconversion of Anti-Vi IgG Response at Week 28 (Two-dose Vaccine group vs· Comparator)

Following comparision is tested without multiple testing adjustment, no decision-making criteria

[3] Seroconversion of Anti-Vi IgG Response at Week 28 (One-dose vs· Two-dose)

* Seroconversion of Anti-Vi IgG Response at Week 4 of single-dose group vs· Week 28 of two-dose group, p-value 0·1458

[4] Seroconversion of Anti-Vi IgG ELISA Response at Week 24 vs. Week 28 of two-dose regimen

Table S18 Seroconversion of Anti-Vi IgG Response by age strata – per protocol set

| **Vi-DT Dose** | **Time point** | | | **Vi-DT Group** | | | | | | | | | | | | | | | | | | | | | | | **Comparator Group** | | | | | | |  |
| --- | --- | --- | --- | --- | --- | --- | --- | --- | --- | --- | --- | --- | --- | --- | --- | --- | --- | --- | --- | --- | --- | --- | --- | --- | --- | --- | --- | --- | --- | --- | --- | --- | --- | --- |
|  |  |  |  | **Any dose** | | | | | | | | | | **Single dose** | | | | | | **two-dose** | | | | | | |  |  |  |  |  |  |  |  |
|  |  |  |  | N | | | | | | | SCN (95% CI) | | | N | | | | | SCN (95% CI) | N | | | | | SCN (95% CI) | | N | | | SCN (95% CI) | | | |  |
| **Age Strata1: 6 to less than 9 months** | | | | | | | | | | | | | | | | | | | | | | | | | | | | | | | | | |  |
| First Dose | Day 0 | | | 74 | | | | | | | -- | | | 37 | | | | | -- | 37 | | | | | -- | | 17 | | | -- | | | |  |
|  | Week 4 | | | 74 | | | | | | | 100.0 (95.07, 100.0) | | | 37 | | | | | 100.0 (90.59, 100.0) | 37 | | | | | 100.0 (90.59, 100.0) | | 17 | | | 11.76 (3.29, 34.34) | | | |  |
| Second Dose | Week 24 | | | 74 | | | | | | | 97.30 (90.67, 99.26) | | | 37 | | | | | 100.0 (90.59, 100.0) | 37 | | | | | 94.59 (82.30, 98.50) | | 17 | | | 17.65 (6.19, 41.03) | | | |  |
|  | Week 28 | | | 74 | | | | | | | 100.0 (95.07, 100.0) | | | 37 | | | | | 100.0 (90.59, 100.0) | 37 | | | | | 100.0 (90.59, 100.0) | | 17 | | | 23.53 (9.56, 47.26) | | | |  |
| **Age Strata2: 9 to 12 m** | | | | | | |  | | | | |  | | | | | | | | | | |  | | | | | | | | |  | |  |
| First Dose | | Day 0 | | | 66 | | | | -- | | | | | | 35 | | -- | | | | 41 | | | | -- | 18 | | | -- | | | | |  |
|  |  | Week 4 | | | 66 | | | | 100.0 (94.50, 100.0) | | | | | | 35 | | 100.0 (90.11, 100.0) | | | | 31 | | | | 100.0 (88.97, 100.0) | 18 | | | 0.00 (-) | | | | |  |
| Second Dose | | Week 24 | | | 66 | | | | 100.0 (94.50, 100.0) | | | | | | 35 | | 100.0 (90.11, 100.0) | | | | 31 | | | | 100.0 (88.97, 100.0) | 18 | | | 11.11 (3.10, 32.80) | | | | |  |
|  |  | Week 28 | | | 66 | | | | 98.48 (91.90, 99.73) | | | | | | 35 | | 100.0 (90.11, 100.0) | | | | 31 | | | | 96.77 (83.81, 99.43) | 18 | | | 16.67 (5.84, 39.22) | | | | |  |
| **Age Strata3: 13 to 23 m** | | | | | | | |  | | | | |  | | | | | | | | | | |  | | | | | | | | |  | |
| First Dose | | | Day 0 | | | 76 | | | | -- | | | | | | 38 | | -- | | | | 38 | | | -- | | | 19 | | | -- | | | |
|  |  |  | Week 4 | | | 76 | | | | 100.0 (95.19, 100.0) | | | | | | 38 | | 100.0 (90.82, 100.0) | | | | 38 | | | 100.0 (90.82, 100.0) | | | 19 | | | 5.26 (0.94, 24.64) | | | |
| Second Dose | | | Week 24 | | | 76 | | | | 97.37 (90.90, 99.28) | | | | | | 38 | | 97.37 (86.51, 99.53) | | | | 38 | | | 97.37 (86.51, 99.53) | | | 19 | | | 36.84 (19.15, 58.96) | | | |
|  |  |  | Week 28 | | | 76 | | | | 97.37 (90.90, 99.28) | | | | | | 38 | | 97.37 (86.51, 99.53) | | | | 38 | | | 97.37 (86.51, 99.53) | | | 19 | | | 21.05 (8.51, 43.33) | | | |

N – Number tested; SCN – Seroconversion rate


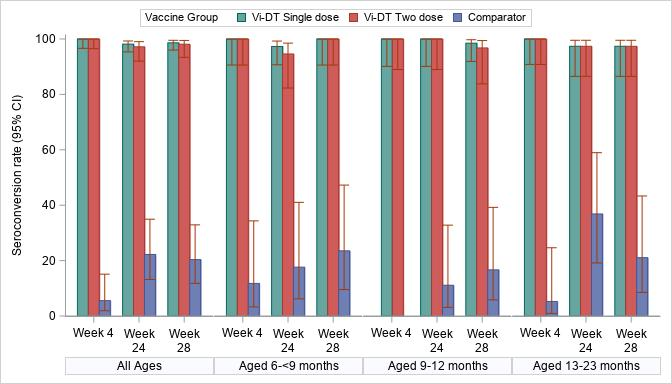


Figure S6 Seroconversion of Anti-Vi IgG ELISA Response – Per Protocol set

The bars represent the percent serconversion, defined as 4-fold rise in the anti-Vi IgG titer at week 4 as compared to baseline value. The error bars represent the standard deviation of the proportion seroconversion.

Table S19 GMT and GMF rise of Anti-Vi IgG Response by age strata- Immunogenicity set

| **Response** | **Vi-DT dose** | **Time point** | **Vi-DT Group** | | | | | | | **Comparator Group** | |
| --- | --- | --- | --- | --- | --- | --- | --- | --- | --- | --- | --- |
|  |  |  | **Any dose** | | **Single dose** | | **two-dose** | | |  |  |
|  |  |  | N | IU/mL (95% CI) | N | IU/mL (95% CI) | N | IU/mL (95% CI) | | N | IU/mL (95% CI) |
| **Age Strata1: 6 to less than 9 m** | | | | | | | | | | | |
| **GMT**^a^ | First Dose | Day 0 | 76 | 0·31 (0·23, 0·41) | 38 | 0·27 (0·18, 0·40) | 38 | 0·35 (0·23, 0·52) | | 19 | 0·53 (0·30, 0·93) |
|  |  | Week 4 | 76 | 308·80 (268·28, 355·43) | 38 | 259·73 (214·04, 315·17) | 38 | 367·13 (302·55, 445·50) | | 19 | 0·39 (0·30, 0·51) |
|  | Second Dose | Week 24 | 76 | 29·55 (24·80, 35·22) | 38 | 24·31 (19·07, 30·99) | 38 | 35·93 (28·19, 45·80) | | 17 | 0·50 (0·35, 0·71) |
|  |  | Week 28 | 75 | 62·69 (44·71, 87·89) | 38 | 19·48 (14·08, 26·95) | 37 | 208·17 (149·84, 289·20) | | 17 | 0·75 (0·46, 1·22) |
| **GMFrise^b^** | First Dose | Day 0 | 76 |  | 38 |  | 38 |  | | 19 |  |
|  |  | Week 4 | 76 | 1006·84 (753·02, 1346·21) | 38 | 964·40 (638·17, 1457·39) | 38 | 1051·14 (695·57, 1588·47) | | 19 | 0·74 (0·41, 1·32) |
|  | Second Dose | Week 24 | 76 | 96·36 (71·21, 130·40) | 38 | 90·27 (58·73, 138·73) | 38 | 102·87 (66·94, 158·10) | | 17 | 0·94 (0·49, 1·78) |
|  |  | Week 28 | 75 | 202·26 (138·38, 295·63) | 38 | 72·34 (46·65, 112·18) | 37 | 581·46 (372·74, 907·06) | | 17 | 1·42 (0·74, 2·73) |
| **Age Strata2: 9 to 12 m** | | |  | |  | | | |  | | |
| **GMT^a^** | First Dose | Day 0 | 76 | 0·31 (0·24, 0·40) | 38 | 0·29 (0·20, 0·41) | 38 | 0·33 (0·23, 0·48) | | 19 | 0·46 (0·28, 0·76) |
|  |  | Week 4 | 76 | 533·50 (437·10, 651·16) | 38 | 502·67 (378·81, 667·02) | 38 | 566·22 (426·70, 751·34) | | 19 | 0·51 (0·34, 0·75) |
|  | Second Dose | Week 24 | 70 | 40·34 (32·79, 49·63) | 36 | 34·26 (25·73, 45·62) | 34 | 47·95 (35·71, 64·37) | | 19 | 0·40 (0·27, 0·60) |
|  |  | Week 28 | 70 | 64·52 (43·73, 95·17) | 36 | 25·62 (16·09, 40·81) | 34 | 171·51 (106·23, 276·92) | | 19 | 0·69 (0·36, 1·31) |
| **GMFrise^b^** | First Dose | Day 0 | 76 | -- | 38 | -- | 38 | -- | | 19 | -- |
|  |  | Week 4 | 76 | 1730·45 (1320·94, 2266·91) | 38 | 1757·55 (1197·14, 2580·30) | 38 | 1703·77 (1160·50, 2501·34) | | 19 | 1·10 (0·64, 1·89) |
|  | Second Dose | Week 24 | 70 | 137·45 (100·13, 188·67) | 36 | 120·75 (77·57, 187·97) | 34 | 157·65 (99·98, 248·59) | | 19 | 0·87 (0·48, 1·61) |
|  |  | Week 28 | 70 | 219·83 (138·64, 348·55) | 36 | 90·30 (50·26, 162·21) | 34 | 563·94 (308·64, 1030·43) | | 19 | 1·50 (0·67, 3·35) |
| **Age Strata3: 13 to 23 m** | | |  | |  | | | |  | | |
| **GMT^a^** | First Dose | Day 0 | 76 | 0·45 (0·33, 0·62) | 38 | 0·44 (0·28, 0·69) | 38 | 0·46 (0·29, 0·72) | | 19 | 0·32 (0·17, 0·60) |
|  |  | Week 4 | 76 | 532·69 (444·34, 638·60) | 38 | 567·62 (438·89, 734·11) | 38 | 499·90 (386·53, 646·53) | | 19 | 0·36 (0·25, 0·51) |
|  | Second Dose | Week 24 | 76 | 60·39 (49·22, 74·09) | 38 | 59·93 (44·81, 80·16) | 38 | 60·85 (45·49, 81·39) | | 19 | 0·75 (0·50, 1·13) |
|  |  | Week 28 | 76 | 103·67 (81·56, 131·78) | 38 | 47·27 (36·74, 60·82) | 38 | 227·37 (176·73, 292·54) | | 19 | 0·46 (0·32, 0·66) |
| **GMFrise^b^** | First Dose | Day 0 | 76 | -- | 38 | -- | 38 | -- | | 19 | -- |
|  |  | Week 4 | 76 | 1182·89 (872·07, 1604·49) | 38 | 1287·07 (834·90, 1984·13) | 38 | 1087·14 (705·21, 1675·93) | | 19 | 1·12 (0·61, 2·06) |
|  | Second Dose | Week 24 | 76 | 134·10 (95·96, 187·39) | 38 | 135·89 (84·43, 218·71) | 38 | 132·33 (82·22, 212·97) | | 19 | 2·34 (1·19, 4·58) |
|  |  | Week 28 | 76 | 230·22 (158·57, 334·24) | 38 | 107·19 (66·28, 173·33) | 38 | 494·47 (305·78, 799·60) | | 19 | 1·43 (0·73, 2·83) |

^a^ Geometric Mean Titers (unit: IU/ml)

^b^ Geometric Mean Fold rise from baseline (Day 0) to post dose

95% CI – 95% Confidence Interval

Table S20 GMT of Anti-Vi IgG Response, all ages – Per Protocol Set

| **Response** | **Vi-DT dose** | **Time point** | **Vi-DT Group** | | | | | | **Comparator Group** | | **P-value**† |
| --- | --- | --- | --- | --- | --- | --- | --- | --- | --- | --- | --- |
|  |  |  | **Any dose** | | **Single dose** | | **two-dose** | |  |  |  |
|  |  |  | N | IU/mL (95% CI) | N | IU/mL (95% CI) | N | IU/mL (95% CI) | N | IU/mL (95% CI) |  |
| **GMT^a^** | First Dose | Day 0 | 216 | 0·34 (0·29, 0·40) | 110 | 0·32 (0·25, 0·40) | 106 | 0·36 (0·28, 0·46) | 54 | 0·43 (0·31, 0·60) | -- |
|  |  | Week 4 | 216 | 448·75 (406·51, 495·38) | 110 | 423·38 (368·71, 486·15) | 106 | 476·77 (414·06, 548·98) | 54 | 0·37 (0·30, 0·45) | -- |
|  | Second Dose | Week 24 | 216 | 41·89 (37·35, 46·98) | 110 | 36·61 (31·22, 42·93) | 106 | 48·19 (40·97, 56·69) | 54 | 0·54 (0·43, 0·67) | -- |
|  |  | Week 28 | 216 | 75·09 (62·30, 90·50) | 110 | 28·42 (23·25, 34·74) | 106 | 206·32 (168·10, 253·21) | 54 | 0·57 (0·43, 0·76) | <0·0001^[1]^ |
| **GMFrise^b^** | First Dose | Day 0 | 216 | -- | 110 | -- | 106 | -- | 54 | -- | -- |
|  |  | Week 4 | 216 | 1326·48 (1126·43, 1562·07) | 110 | 1322·72 (1051·75, 1663·51) | 106 | 1330·41 (1053·01, 1680·87) | 54 | 0·86 (0·62, 1·19) | -- |
|  | Second Dose | Week 24 | 216 | 123·82 (102·80, 149·12) | 110 | 114·37 (88·15, 148·39) | 106 | 134·47 (103·11, 175·39) | 54 | 1·24 (0·86, 1·80) | -- |
|  |  | Week 28 | 216 | 221·95 (176·19, 279·59) | 110 | 88·80 (66·95, 117·77) | 106 | 575·71 (431·63, 767·90) | 54 | 1·32 (0·88, 1·97) | -- |

^a^ Geometric Mean Titers (unit: IU/ml)

^b^ Geometric Mean Fold rise from baseline (Day 0) to post dose

[1] Secondary, GMT of Anti-Vi IgG Response at Week 28 (One-dose vs· Two-dose) Following comparision is tested without multiple testing adjustment, no decision-making criteria

† P-values for comparison of GMTs or GMF rise was adjusted for age strata in the model· * Ratio (95% CI) of GMT of Anti-Vi IgG Response at Week 4 of single-dose group vs· Week 28 of two-dose group is 2·03 (1·62, 2·56)

Figure S7 GMT of Anti-Vi IgG ELISA Response – Per Protocol Set


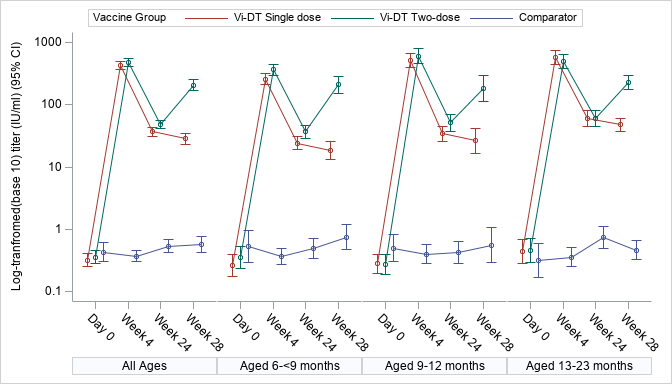


Table S21 GMT of Anti-Vi IgG Response by age strata – Per Protocol Set

| **Response** | **Vi-DT Dose** | **Time point** | **Vi-DT Group** | | | | | | **Comparator Group** | |
| --- | --- | --- | --- | --- | --- | --- | --- | --- | --- | --- |
|  |  |  | **Any dose** | | **Single dose** | | **two-dose** | |  |  |
| **Age Strata1: 6 to less than 9 m** | | | | | | | | | | |
| **GMT^b^ (95% CI)** | First Dose | Day 0 | 74 | 0·31 (0·23, 0·41) | 37 | 0·27 (0·18, 0·40) | 37 | 0·36 (0·24, 0·54) | 17 | 0·53 (0·29, 0·97) |
|  |  | Week 4 | 74 | 306·32 (265·61, 353·27) | 37 | 258·53 (212·44, 314·63) | 37 | 362·94 (298·23, 441·69) | 17 | 0·36 (0·27, 0·49) |
|  | Second Dose | Week 24 | 74 | 29·66 (24·79, 35·49) | 37 | 24·02 (18·76, 30·76) | 37 | 36·62 (28·60, 46·89) | 17 | 0·50 (0·35, 0·72) |
|  |  | Week 28 | 74 | 61·89 (43·99, 87·08) | 37 | 18·40 (13·33, 25·40) | 37 | 208·17 (150·83, 287·31) | 17 | 0·75 (0·47, 1·21) |
| **GMFrise^c^ (95% CI)** | First Dose | Day 0 | 74 | -- | 37 | -- | 37 | -- | 17 | -- |
|  |  | Week 4 | 74 | 994·09 (737·88, 1339·27) | 37 | 974·80 (637·98, 1489·43) | 37 | 1013·76 (663·48, 1548·97) | 17 | 0·69 (0·37, 1·29) |
|  | Second Dose | Week 24 | 74 | 96·26 (70·60, 131·23) | 37 | 90·58 (58·31, 140·71) | 37 | 102·29 (65·85, 158·91) | 17 | 0·94 (0·49, 1·80) |
|  |  | Week 28 | 74 | 200·86 (136·80, 294·92) | 37 | 69·39 (44·51, 108·16) | 37 | 581·46 (373·01, 906·40) | 17 | 1·42 (0·74, 2·73) |
| **Age Strata2: 9 to 12 m** | | | | | | | | | | |
| **GMT^b^ (95% CI)** | First Dose | Day 0 | 66 | 0·28 (0·21, 0·36) | 35 | 0·28 (0·20, 0·40) | 31 | 0·27 (0·19, 0·40) | 18 | 0·49 (0·30, 0·81) |
|  |  | Week 4 | 66 | 554·99 (459·46, 670·38) | 35 | 515·18 (397·26, 668·11) | 31 | 603·64 (457·96, 795·66) | 18 | 0·40 (0·28, 0·57) |
|  | Second Dose | Week 24 | 66 | 41·24 (33·20, 51·23) | 35 | 33·92 (25·31, 45·46) | 31 | 51·42 (37·67, 70·20) | 18 | 0·42 (0·28, 0·63) |
|  |  | Week 28 | 66 | 65·60 (44·34, 97·05) | 35 | 26·17 (16·64, 41·16) | 31 | 185·16 (114·44, 299·57) | 18 | 0·56 (0·30, 1·05) |
| **GMFrise^c^ (95% CI)** | First Dose | Day 0 | 66 | -- | 35 | -- | 31 | -- | 18 | -- |
|  |  | Week 4 | 66 | 2010·88 (1591·62, 2540·57) | 35 | 1845·82 (1337·75, 2546·85) | 31 | 2215·04 (1573·33, 3118·47) | 18 | 0·81 (0·52, 1·27) |
|  | Second Dose | Week 24 | 66 | 149·43 (108·39, 206·00) | 35 | 121·53 (78·38, 188·44) | 31 | 188·70 (118·40, 300·73) | 18 | 0·85 (0·46, 1·57) |
|  |  | Week 28 | 66 | 237·70 (149·81, 377·16) | 35 | 93·76 (53·40, 164·64) | 31 | 679·43 (373·54, 1235·81) | 18 | 1·13 (0·52, 2·49) |
| **Age Strata3: 13 to 23 m** | | | | | | | | | | |
| **GMT^b^ (95% CI)** | First Dose | Day 0 | 76 | 0·45 (0·33, 0·62) | 38 | 0·44 (0·28, 0·69) | 38 | 0·46 (0·29, 0·72) | 19 | 0·32 (0·17, 0·60) |
|  |  | Week 4 | 76 | 532·69 (444·34, 638·60) | 38 | 567·62 (438·89, 734·11) | 38 | 499·90 (386·53, 646·53) | 19 | 0·36 (0·25, 0·51) |
|  | Second Dose | Week 24 | 76 | 60·39 (49·22, 74·09) | 38 | 59·93 (44·81, 80·16) | 38 | 60·85 (45·49, 81·39) | 19 | 0·75 (0·50, 1·13) |
|  |  | Week 28 | 76 | 103·67 (81·56, 131·78) | 38 | 47·27 (36·74, 60·82) | 38 | 227·37 (176·73, 292·54) | 19 | 0·46 (0·32, 0·66) |
| **GMFrise^c^ (95% CI)** | First Dose | Day 0 | 76 | -- | 38 | -- | 38 | -- | 19 | -- |
|  |  | Week 4 | 76 | 1182·89 (872·07, 1604·49) | 38 | 1287·07 (834·90, 1984·13) | 38 | 1087·14 (705·21, 1675·93) | 19 | 1·12 (0·61, 2·06) |
|  | Second Dose | Week 24 | 76 | 134·10 (95·96, 187·39) | 38 | 135·89 (84·43, 218·71) | 38 | 132·33 (82·22, 212·97) | 19 | 2·34 (1·19, 4·58) |
|  |  | Week 28 | 76 | 230·22 (158·57, 334·24) | 38 | 107·19 (66·28, 173·33) | 38 | 494·47 (305·78, 799·60) | 19 | 1·43 (0·73, 2·83) |

GMT of Anti-Vi IgG response results from PP set analysis are in agreement with the results from Immunogenicity set analysis·

Table S22 Seroconversion among children for whom measles, mumps and rubella vaccines were co-administered.

| **Age Strata2: 9 to 12 m** | | |  | |
| --- | --- | --- | --- | --- |
| **Response** | | **Time point** | **Vi-DT Group** | **Comparator Group** |
| Number of participants | | Day 0 | 76 | 19 |
|  |  | Week 4 | 76 | 19 |
| Measles | Antibody titre > 12 unit/mL  (95% CI) | Week 4 | 94·74 (87·23, 97·93) | 100·0 (83·18, 100·0) |
| Mumps | Antibody titre > 12 unit/mL  (95% CI) | Week 4 | 86·84 (77·45, 92·69) | 89·47 (68·61, 97·06) |
| Rubella | Antibody titre > 1·1 antibody index (95% CI) | Week 4 | 98·68 (92·92, 99·77) | 94·74 (75·36, 99·06) |
